# Supplementary figures and images for: Evolutionary rescue of phosphomannomutase deficiency in yeast models of human disease (part 2 of 2)
Source: eLife. 2022 Oct 10;11:e79346. doi: 10.7554/eLife.79346 (PMC9578706; doi:10.7554/eLife.79346)

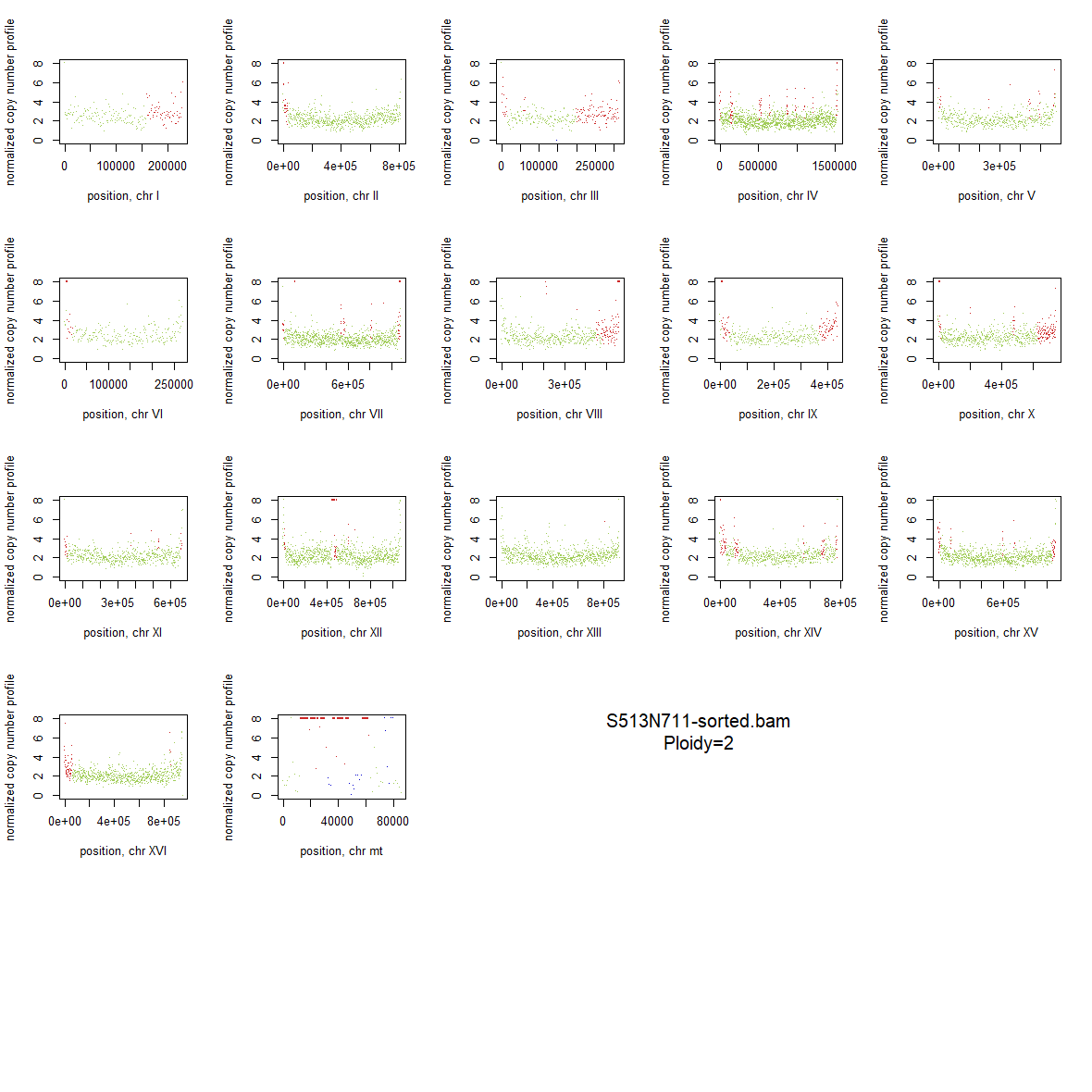

Supplement: Figure 2—source data 2. [file elife-79346-fig2-data2.zip › Figure2-source data 1/pACT1-SEC53-WT/2x_Wildtype_10.png]

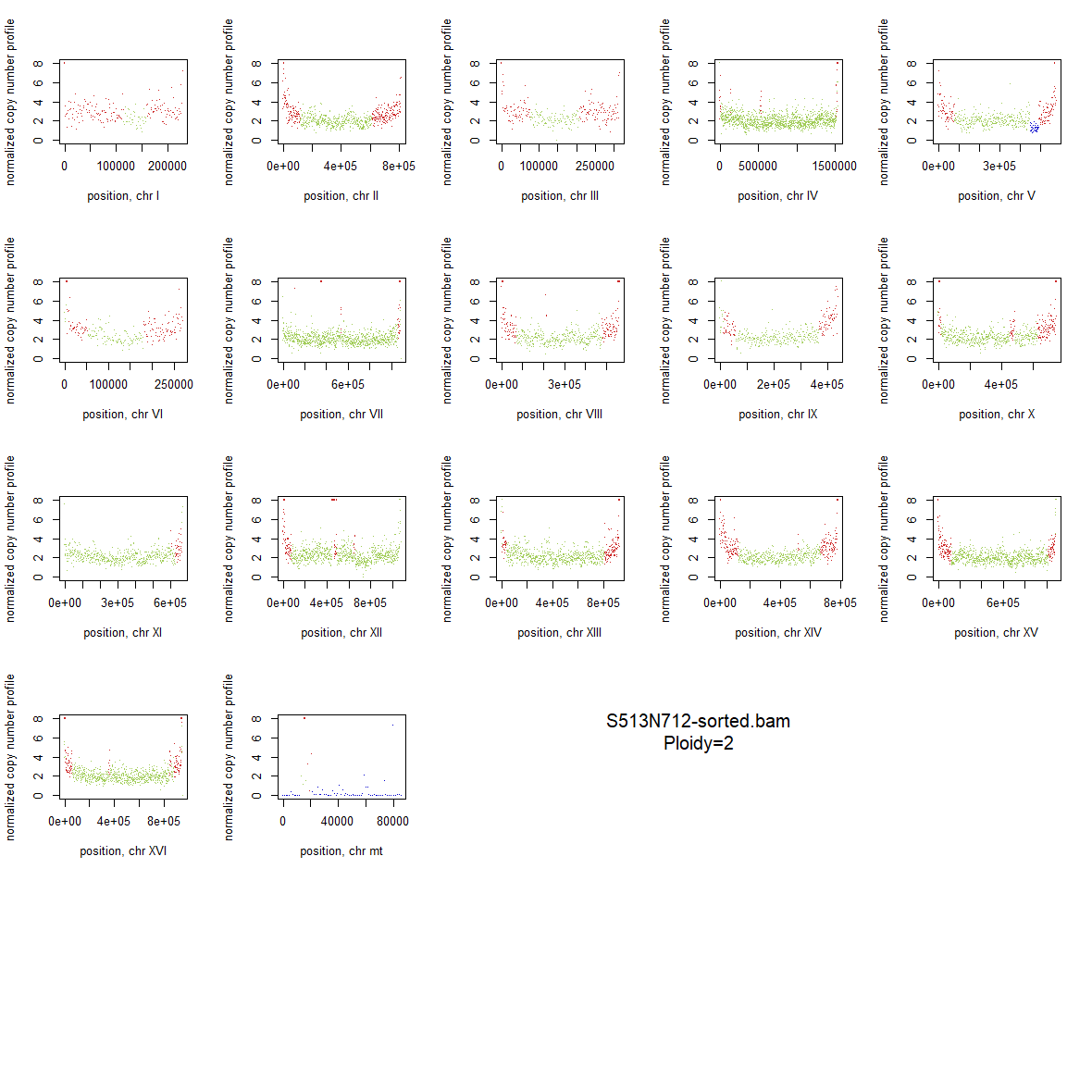

Supplement: Figure 2—source data 2. [file elife-79346-fig2-data2.zip › Figure2-source data 1/pACT1-SEC53-WT/2x_Wildtype_11.png]

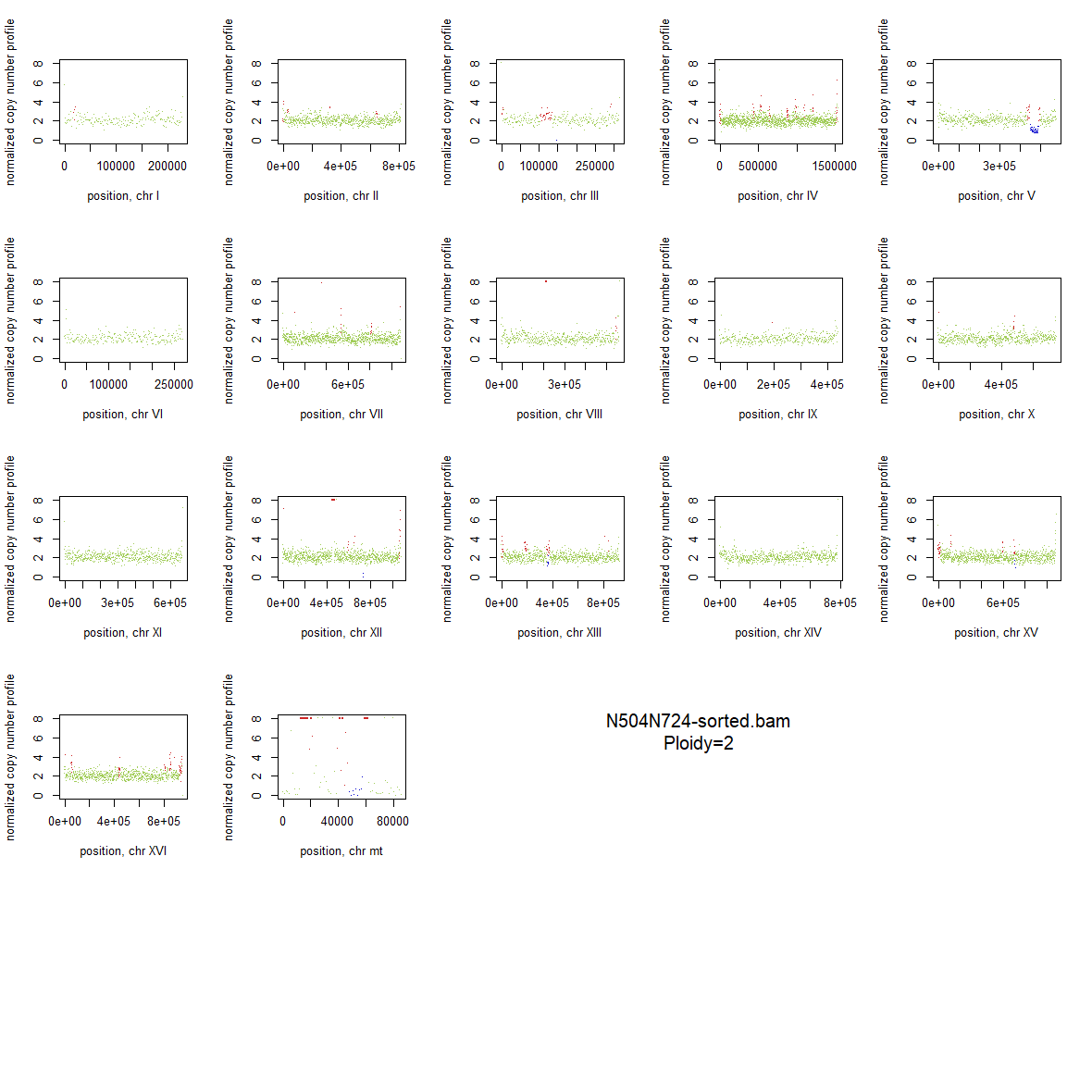

Supplement: Figure 2—source data 2. [file elife-79346-fig2-data2.zip › Figure2-source data 1/pACT-sec53-F126L/2x_F126L_01.png]

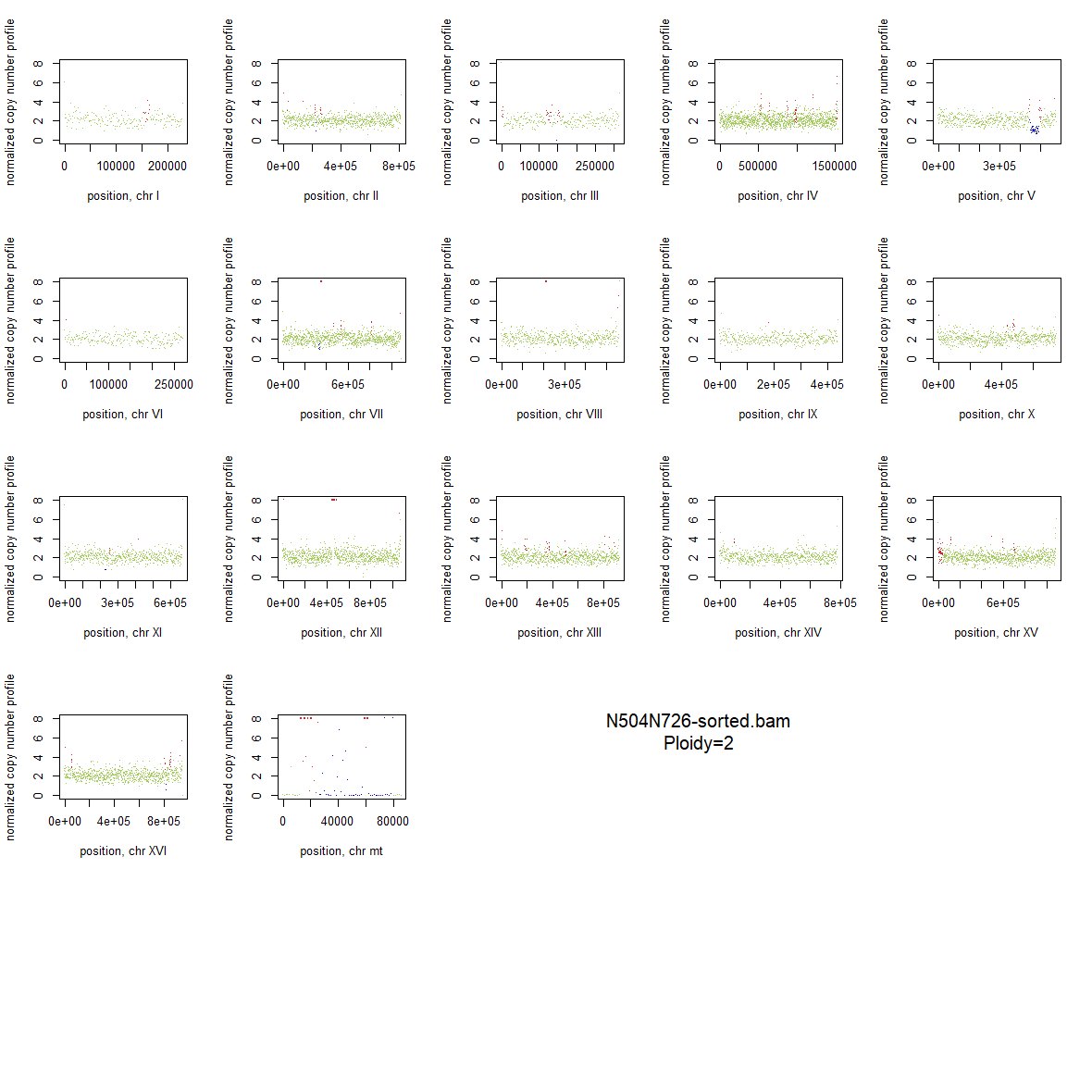

Supplement: Figure 2—source data 2. [file elife-79346-fig2-data2.zip › Figure2-source data 1/pACT-sec53-F126L/2x_F126L_02.png]

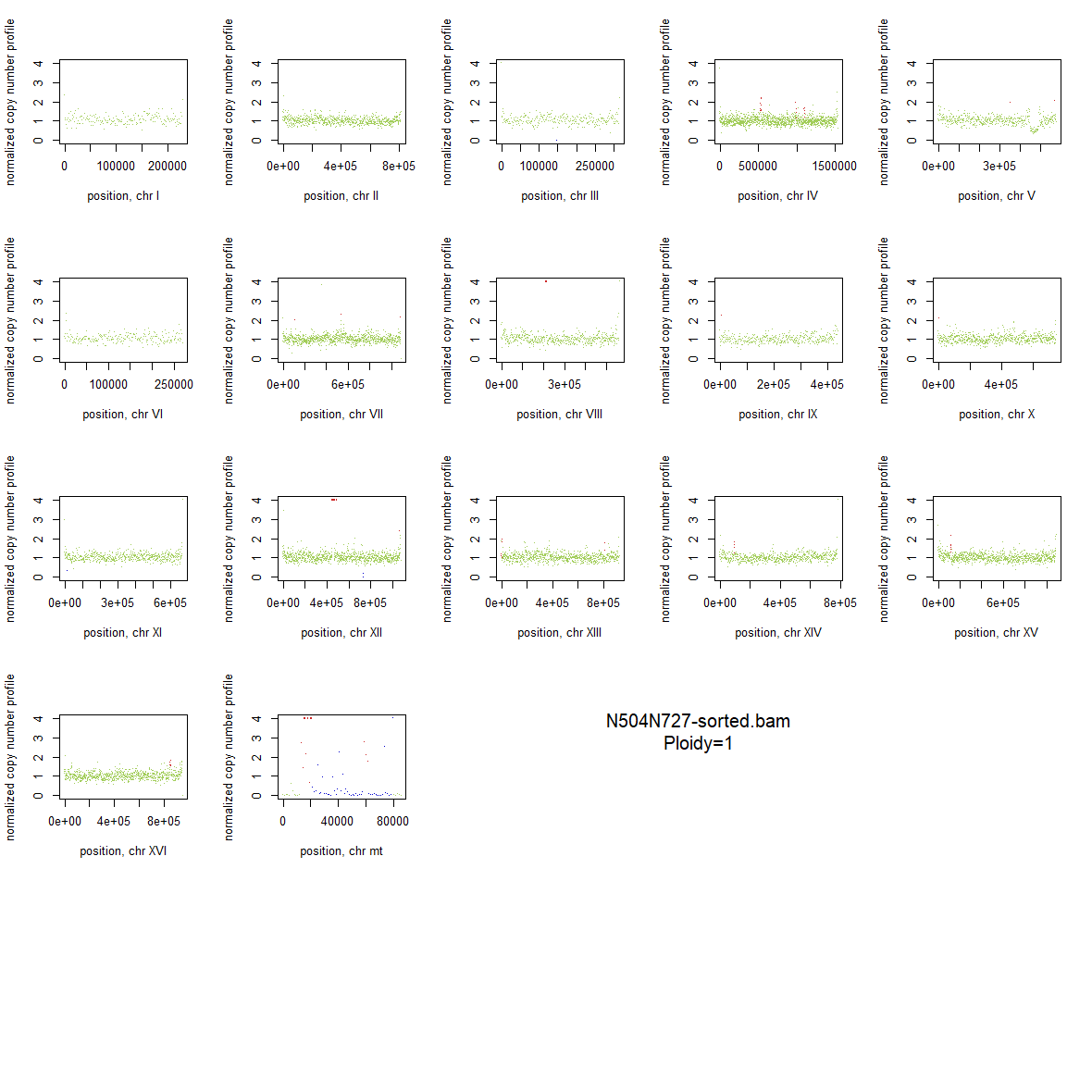

Supplement: Figure 2—source data 2. [file elife-79346-fig2-data2.zip › Figure2-source data 1/pACT-sec53-F126L/2x_F126L_03.png]

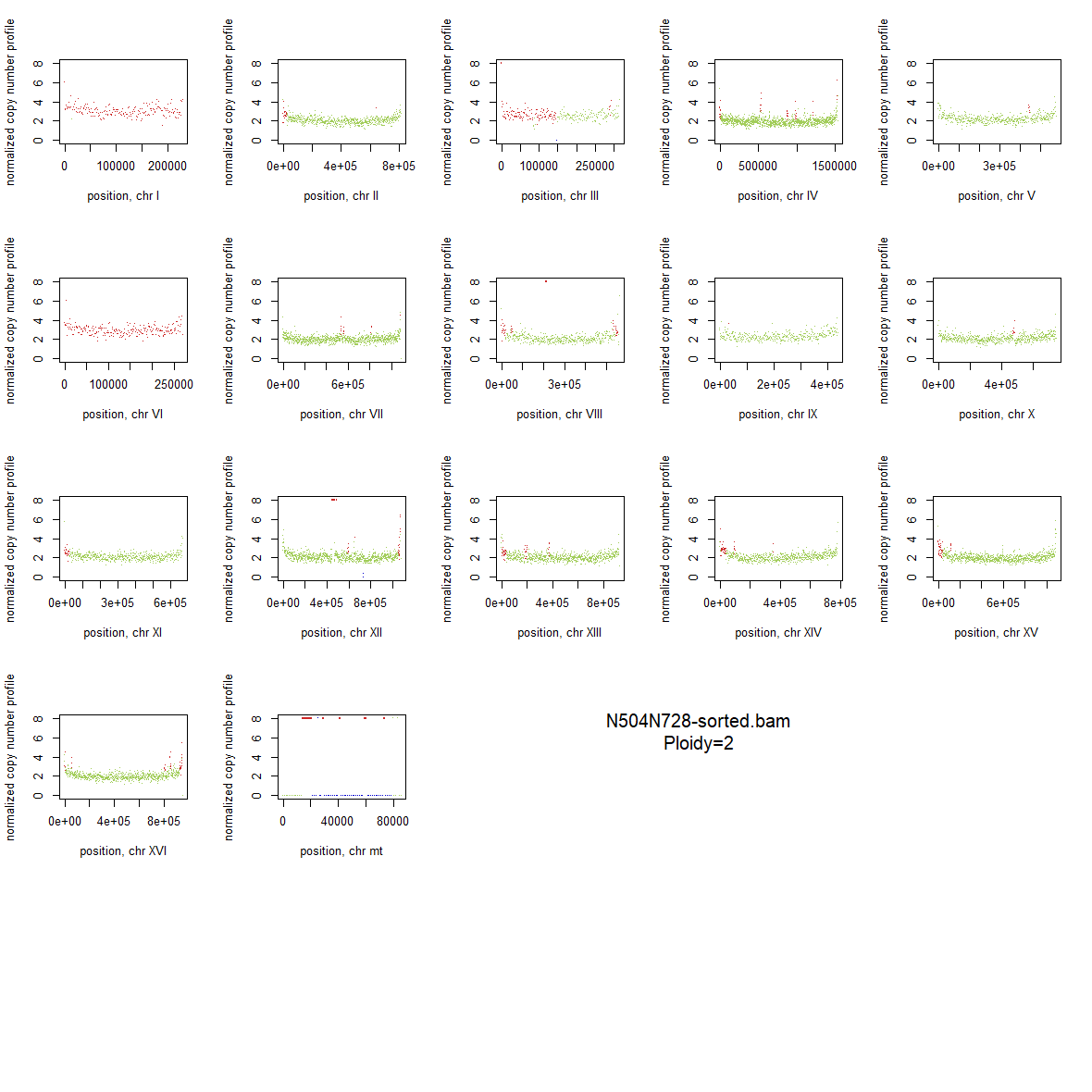

Supplement: Figure 2—source data 2. [file elife-79346-fig2-data2.zip › Figure2-source data 1/pACT-sec53-F126L/2x_F126L_04.png]

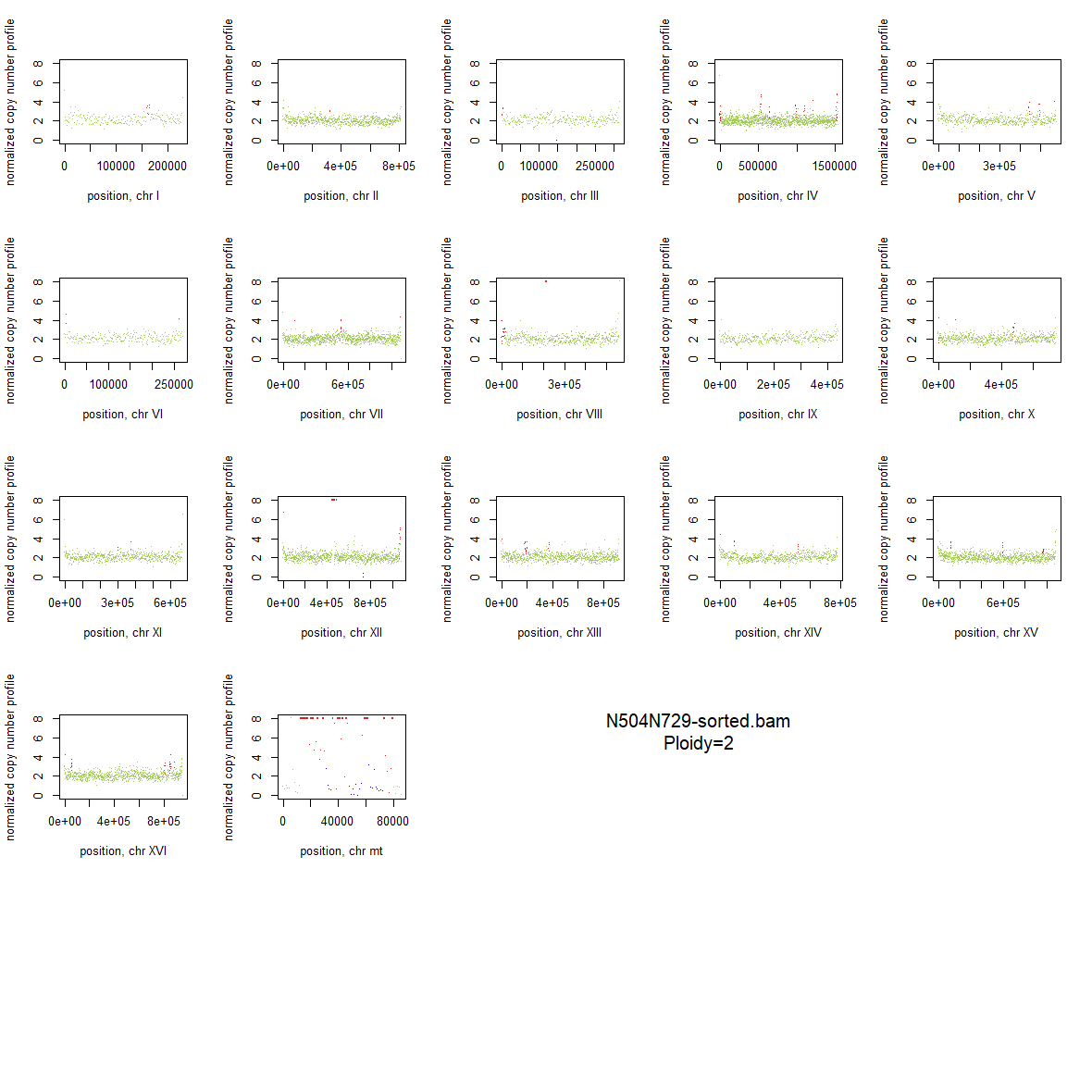

Supplement: Figure 2—source data 2. [file elife-79346-fig2-data2.zip › Figure2-source data 1/pACT-sec53-F126L/2x_F126L_05.png]

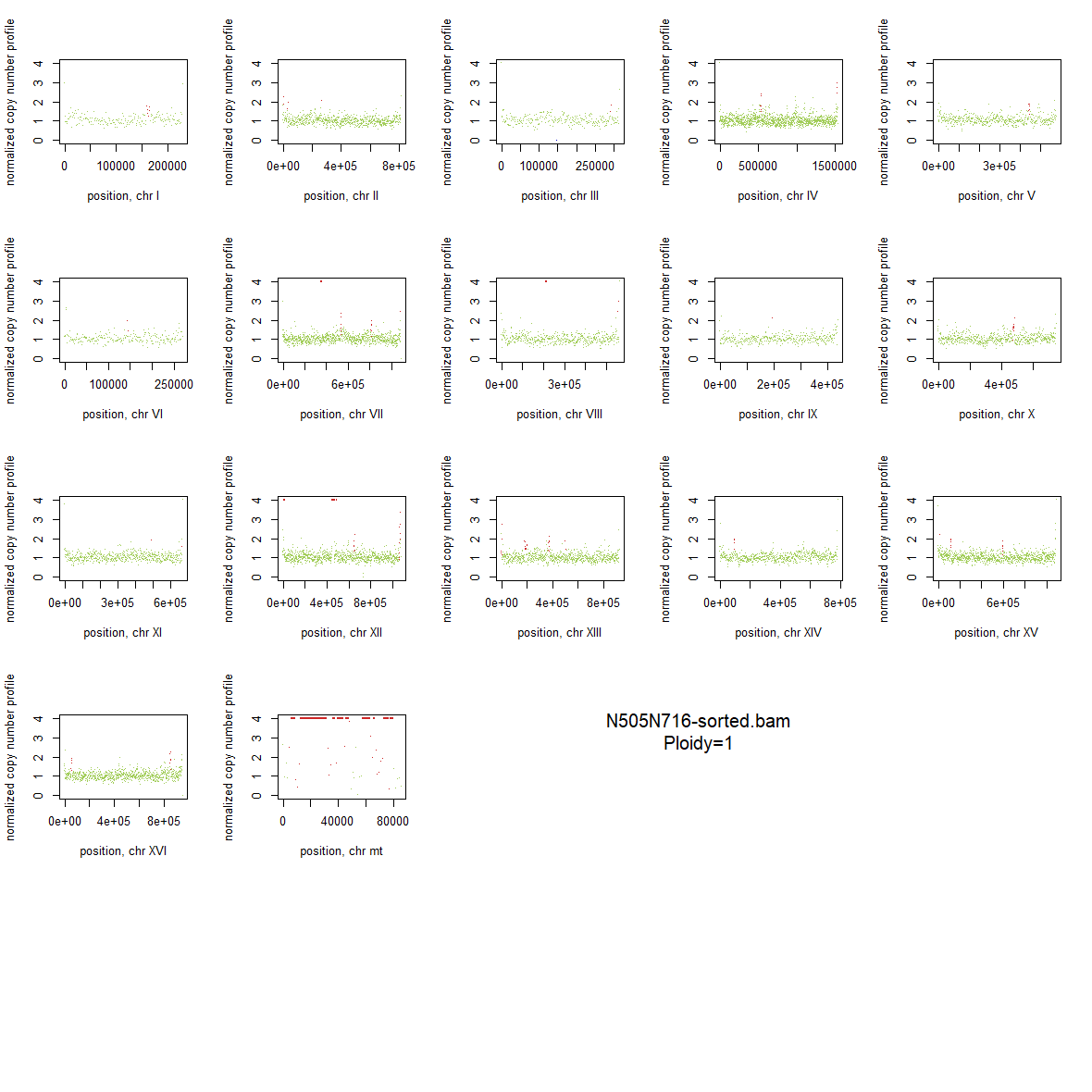

Supplement: Figure 2—source data 2. [file elife-79346-fig2-data2.zip › Figure2-source data 1/pACT-sec53-F126L/2x_F126L_06.png]

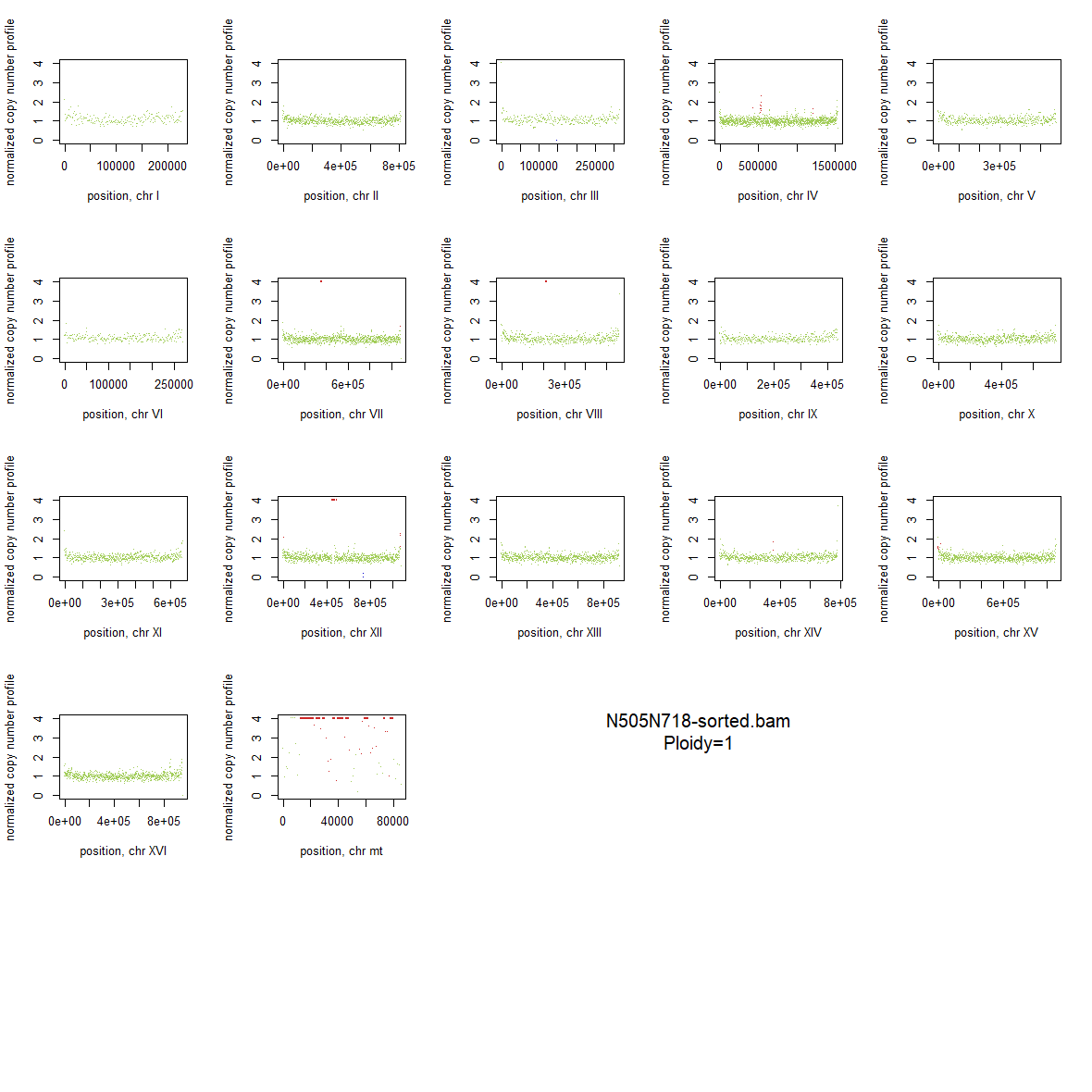

Supplement: Figure 2—source data 2. [file elife-79346-fig2-data2.zip › Figure2-source data 1/pACT-sec53-F126L/2x_F126L_07.png]

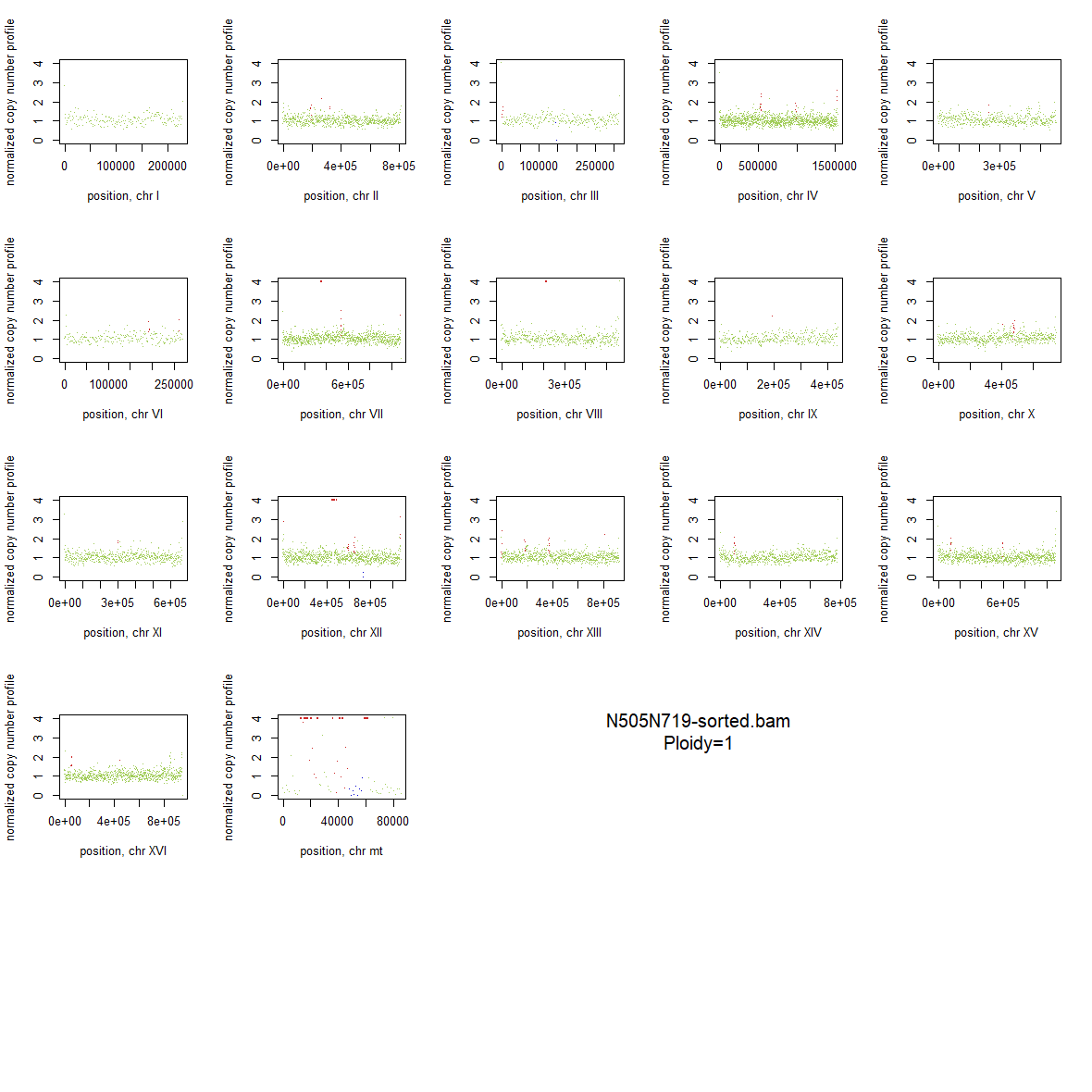

Supplement: Figure 2—source data 2. [file elife-79346-fig2-data2.zip › Figure2-source data 1/pACT-sec53-F126L/2x_F126L_08.png]

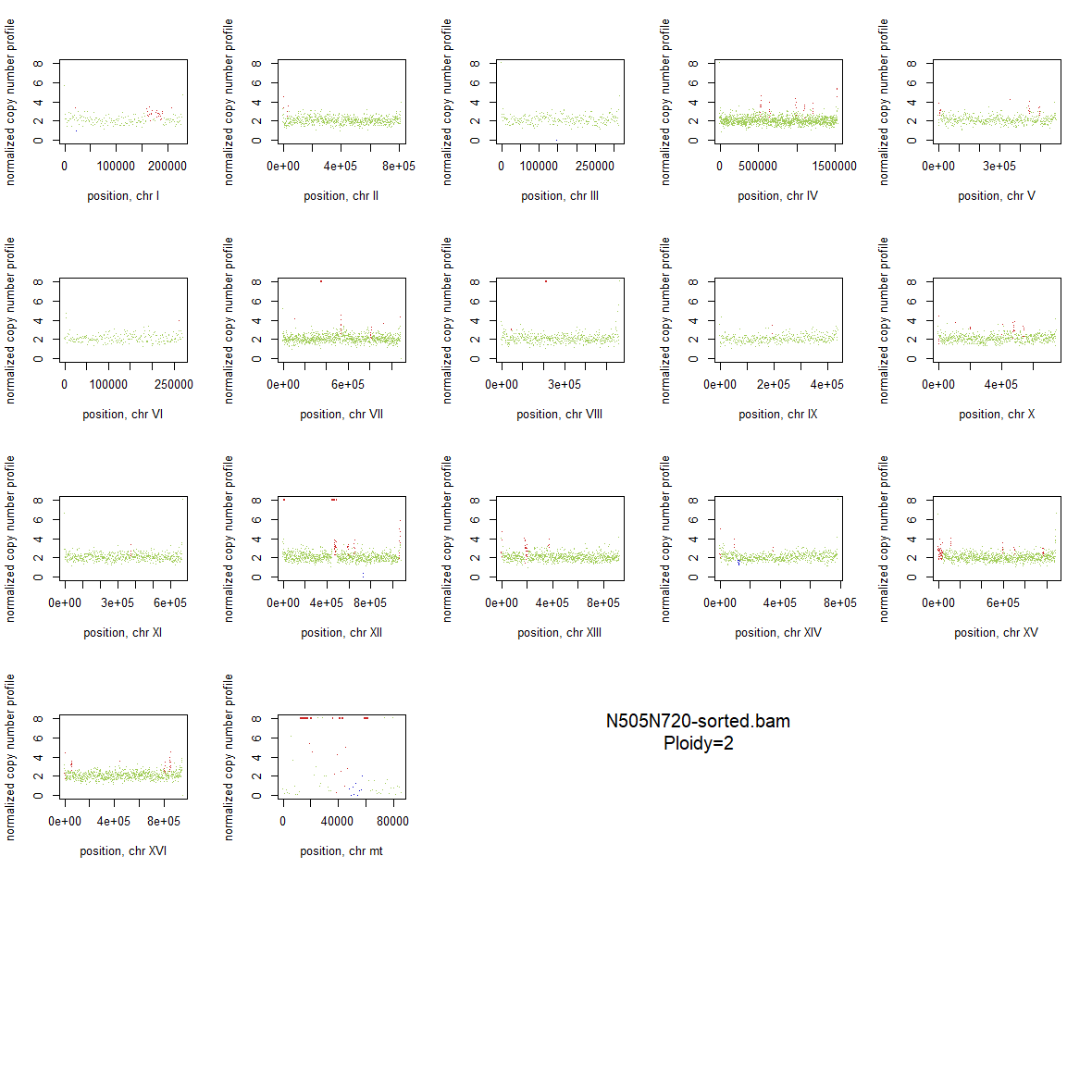

Supplement: Figure 2—source data 2. [file elife-79346-fig2-data2.zip › Figure2-source data 1/pACT-sec53-F126L/2x_F126L_09.png]

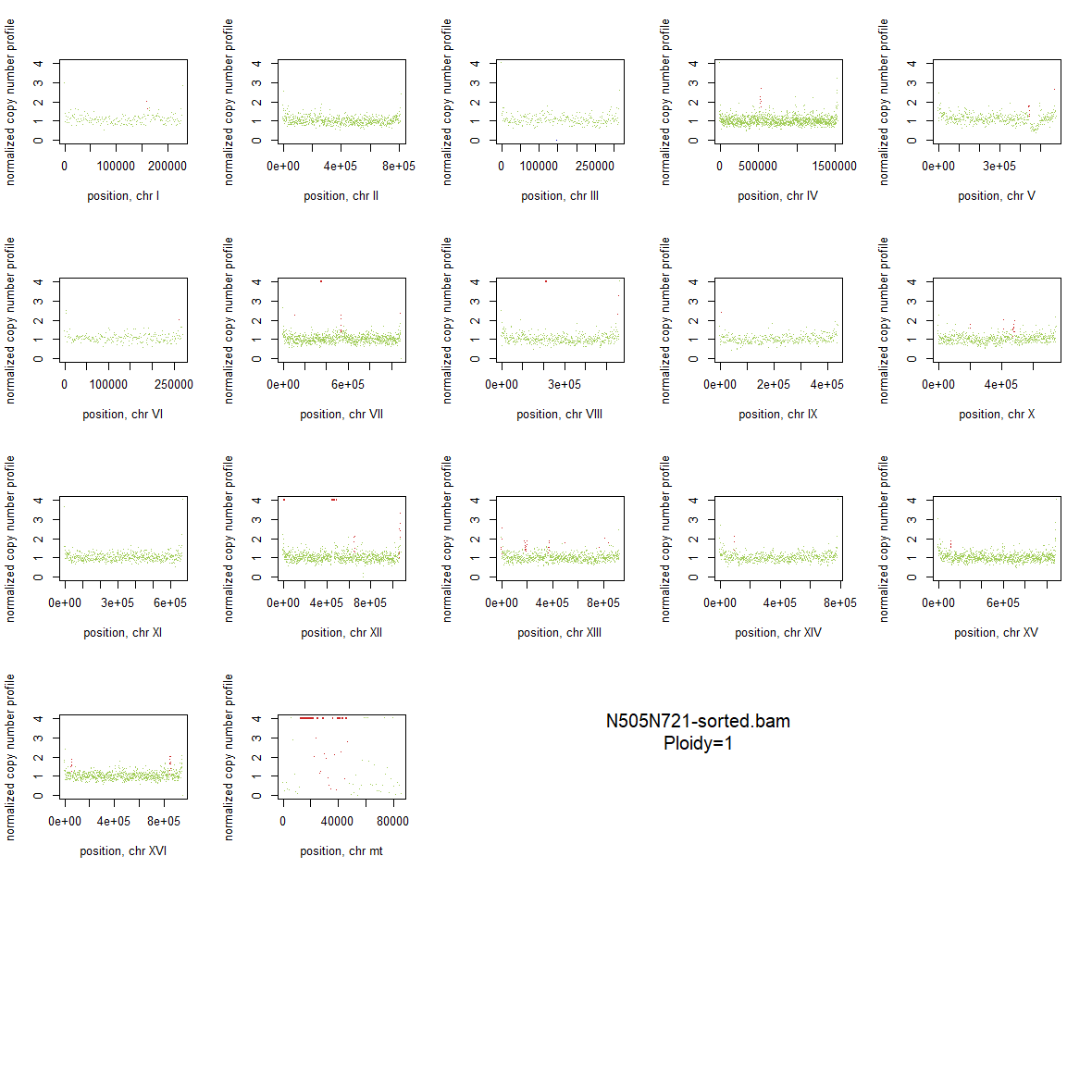

Supplement: Figure 2—source data 2. [file elife-79346-fig2-data2.zip › Figure2-source data 1/pACT-sec53-F126L/2x_F126L_10.png]

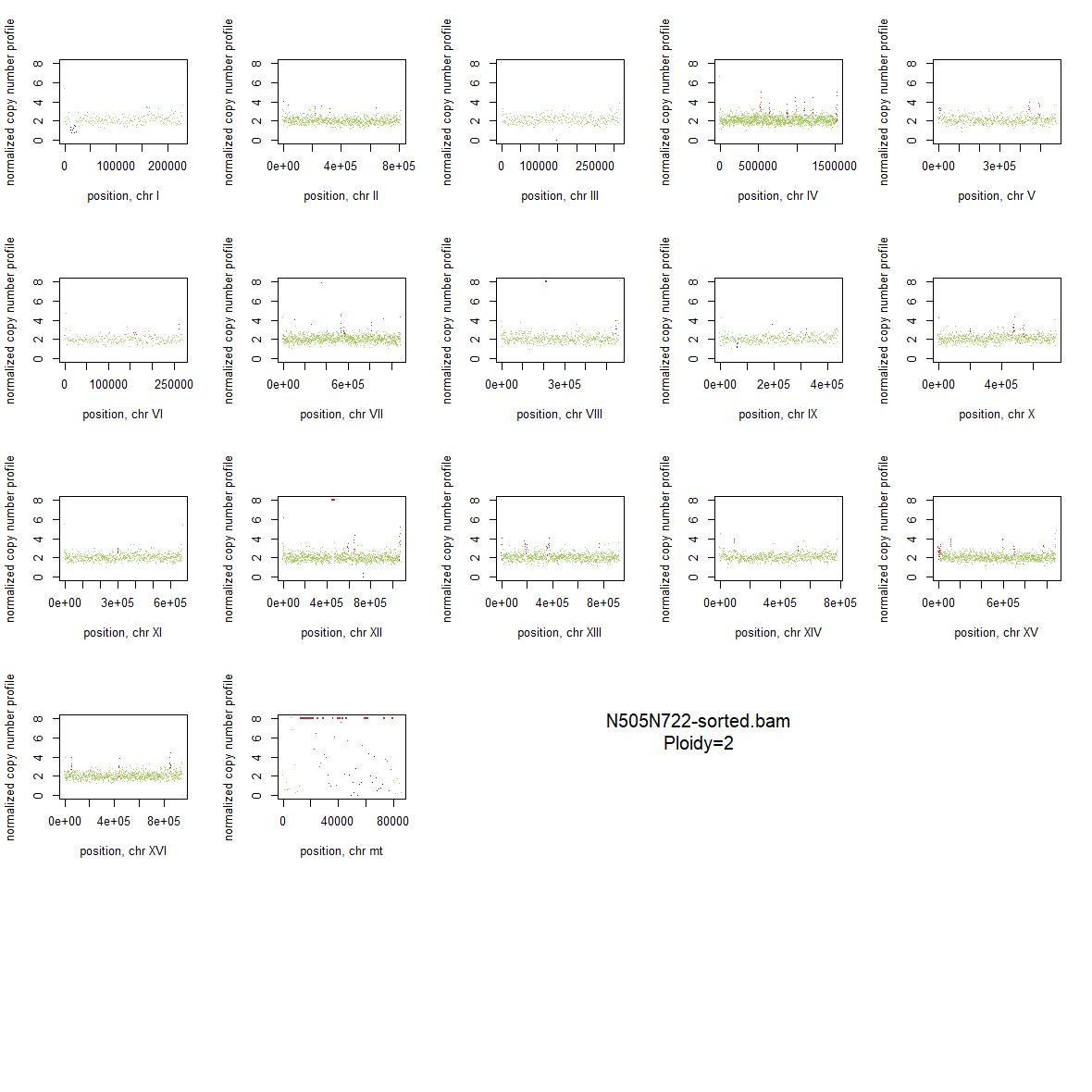

Supplement: Figure 2—source data 2. [file elife-79346-fig2-data2.zip › Figure2-source data 1/pACT-sec53-F126L/2x_F126L_11.png]

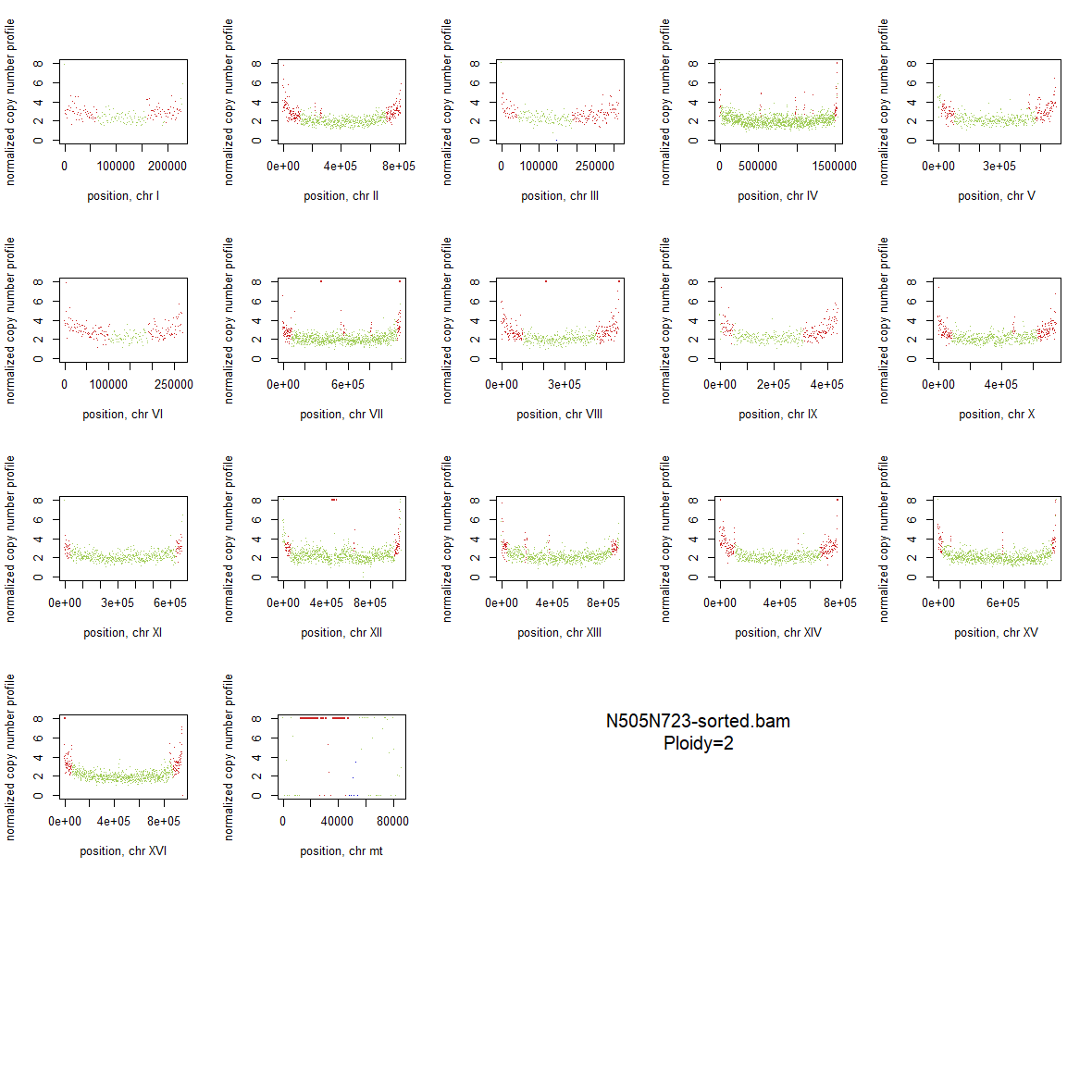

Supplement: Figure 2—source data 2. [file elife-79346-fig2-data2.zip › Figure2-source data 1/pACT-sec53-F126L/2x_F126L_12.png]

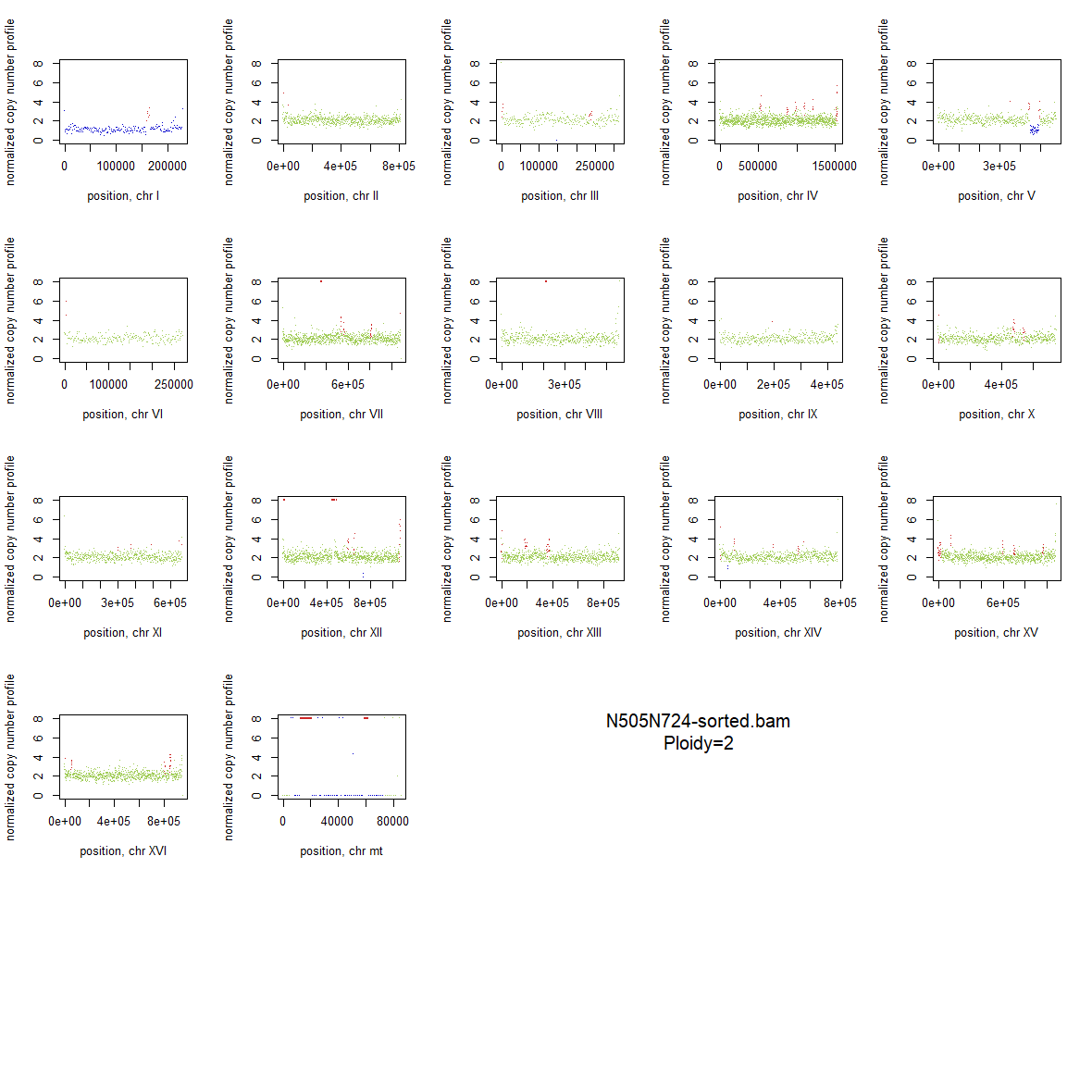

Supplement: Figure 2—source data 2. [file elife-79346-fig2-data2.zip › Figure2-source data 1/pACT-sec53-F126L/2x_F126L_13.png]

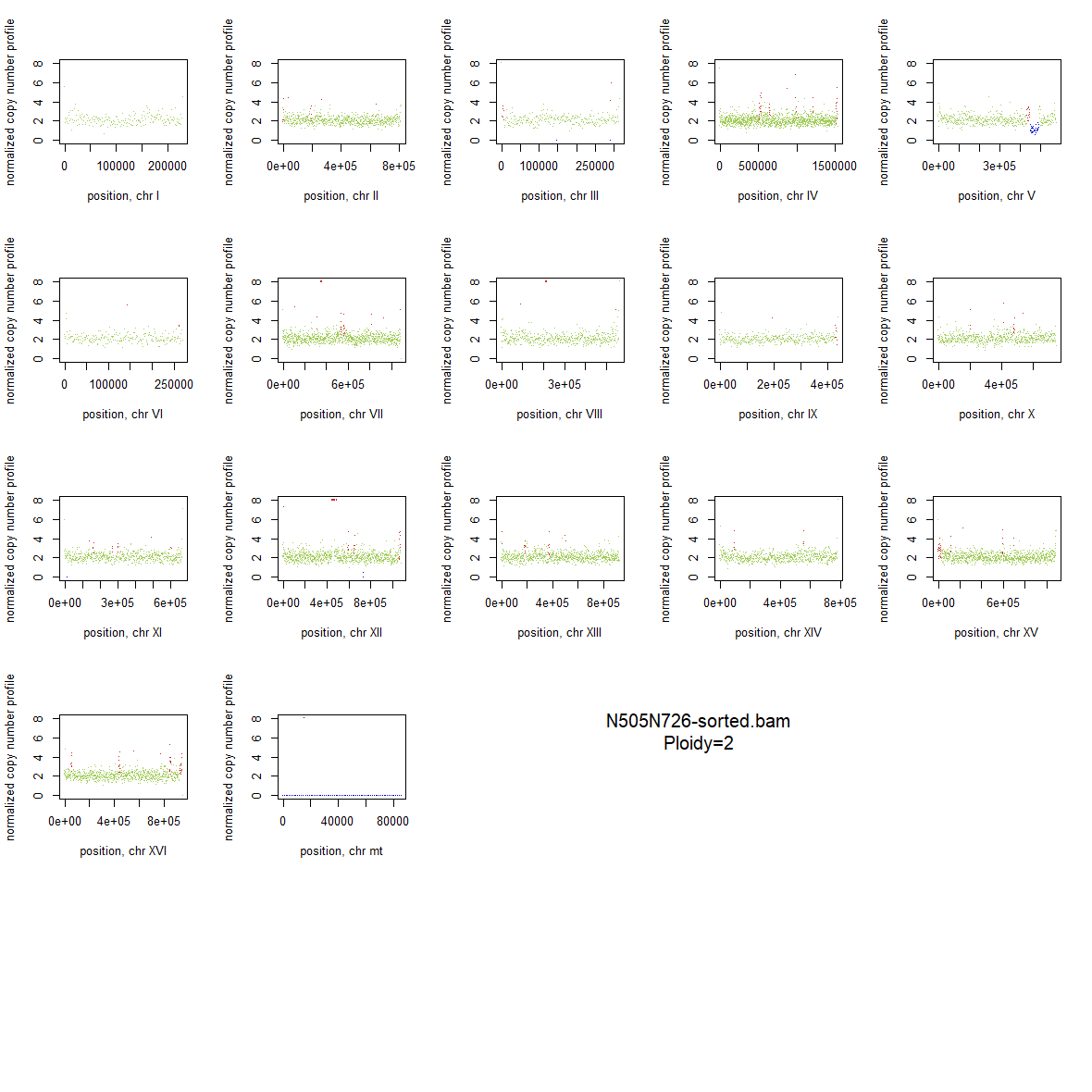

Supplement: Figure 2—source data 2. [file elife-79346-fig2-data2.zip › Figure2-source data 1/pACT-sec53-F126L/2x_F126L_14.png]

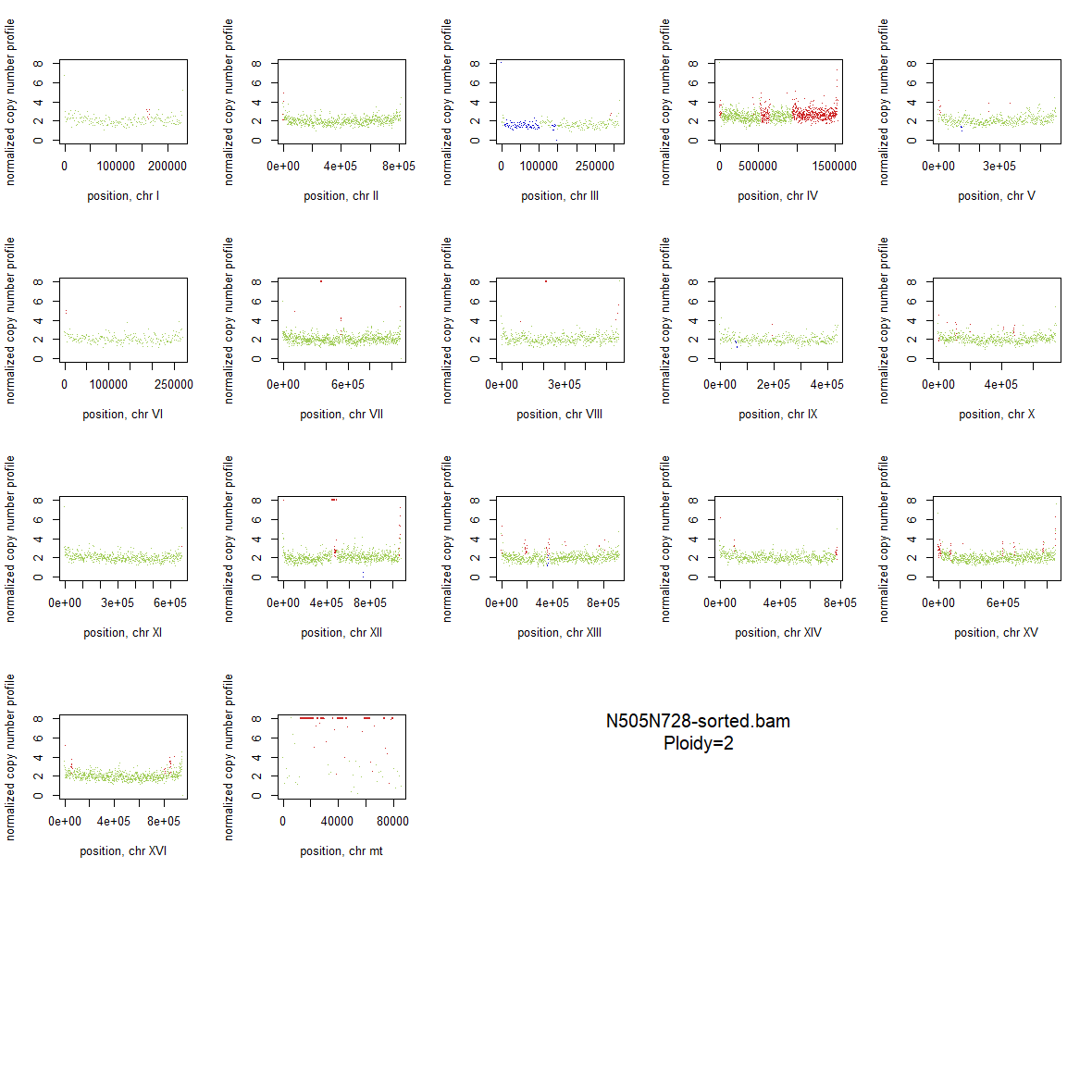

Supplement: Figure 2—source data 2. [file elife-79346-fig2-data2.zip › Figure2-source data 1/pACT-sec53-F126L/2x_F126L_15.png]

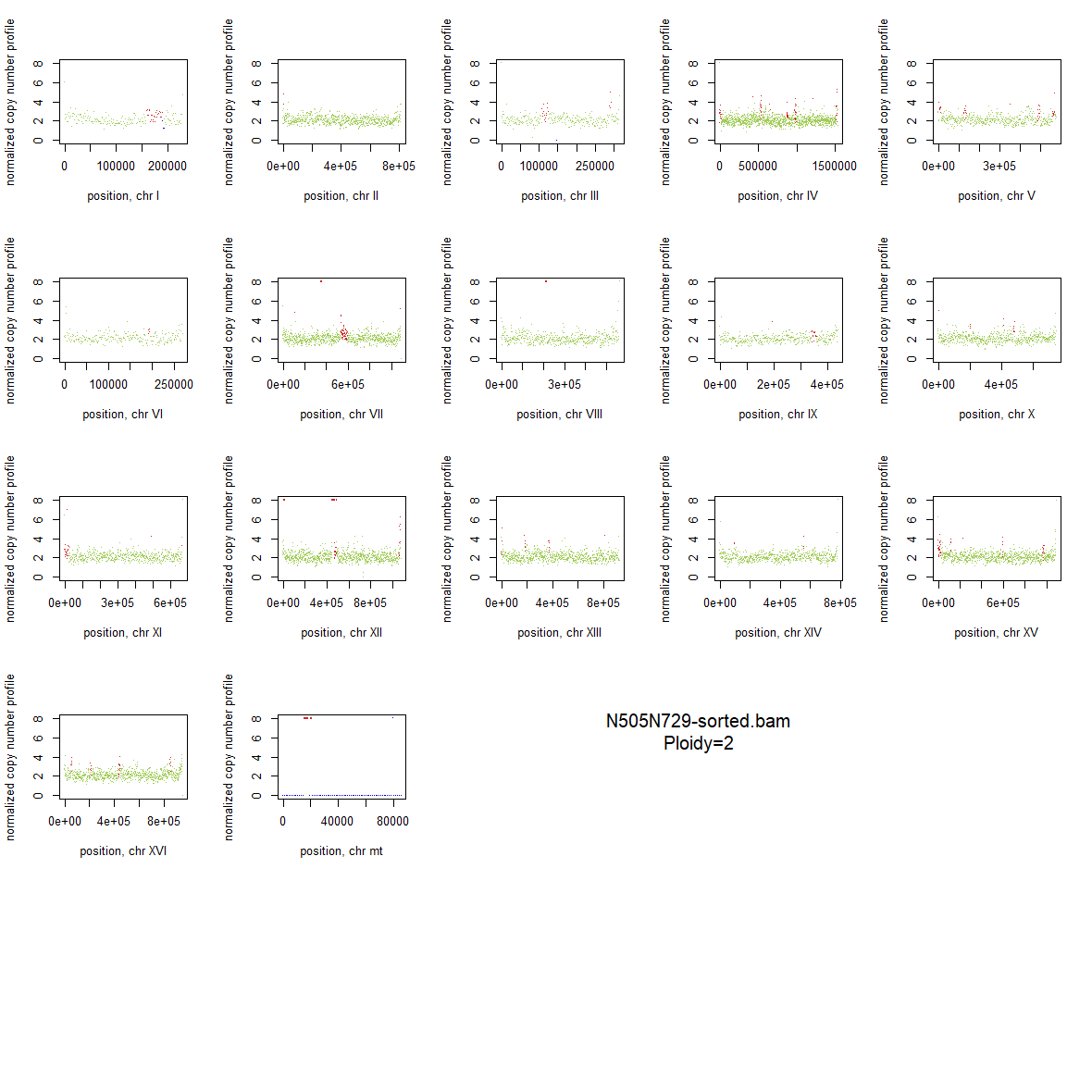

Supplement: Figure 2—source data 2. [file elife-79346-fig2-data2.zip › Figure2-source data 1/pACT-sec53-F126L/2x_F126L_16.png]

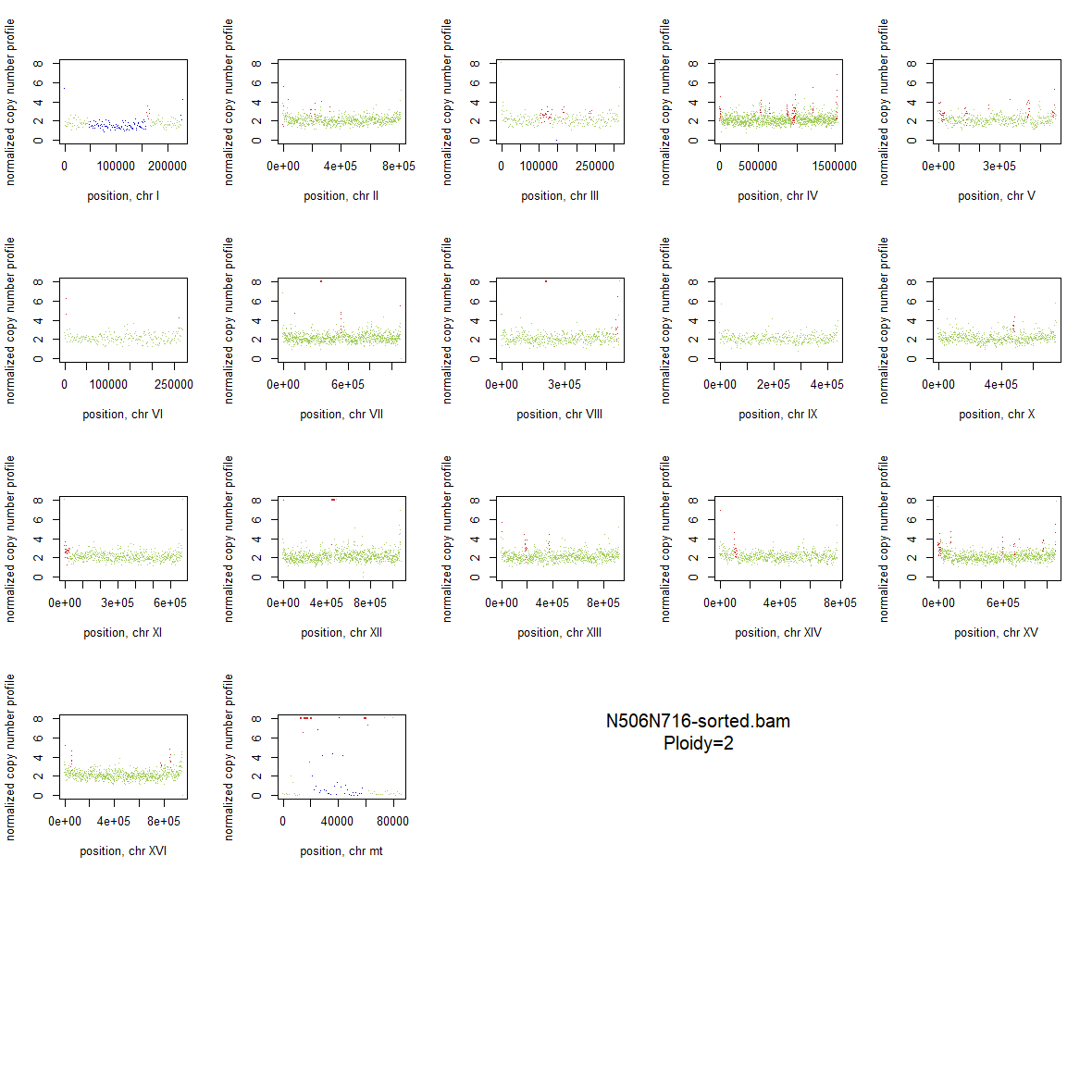

Supplement: Figure 2—source data 2. [file elife-79346-fig2-data2.zip › Figure2-source data 1/pACT-sec53-F126L/2x_F126L_17.png]

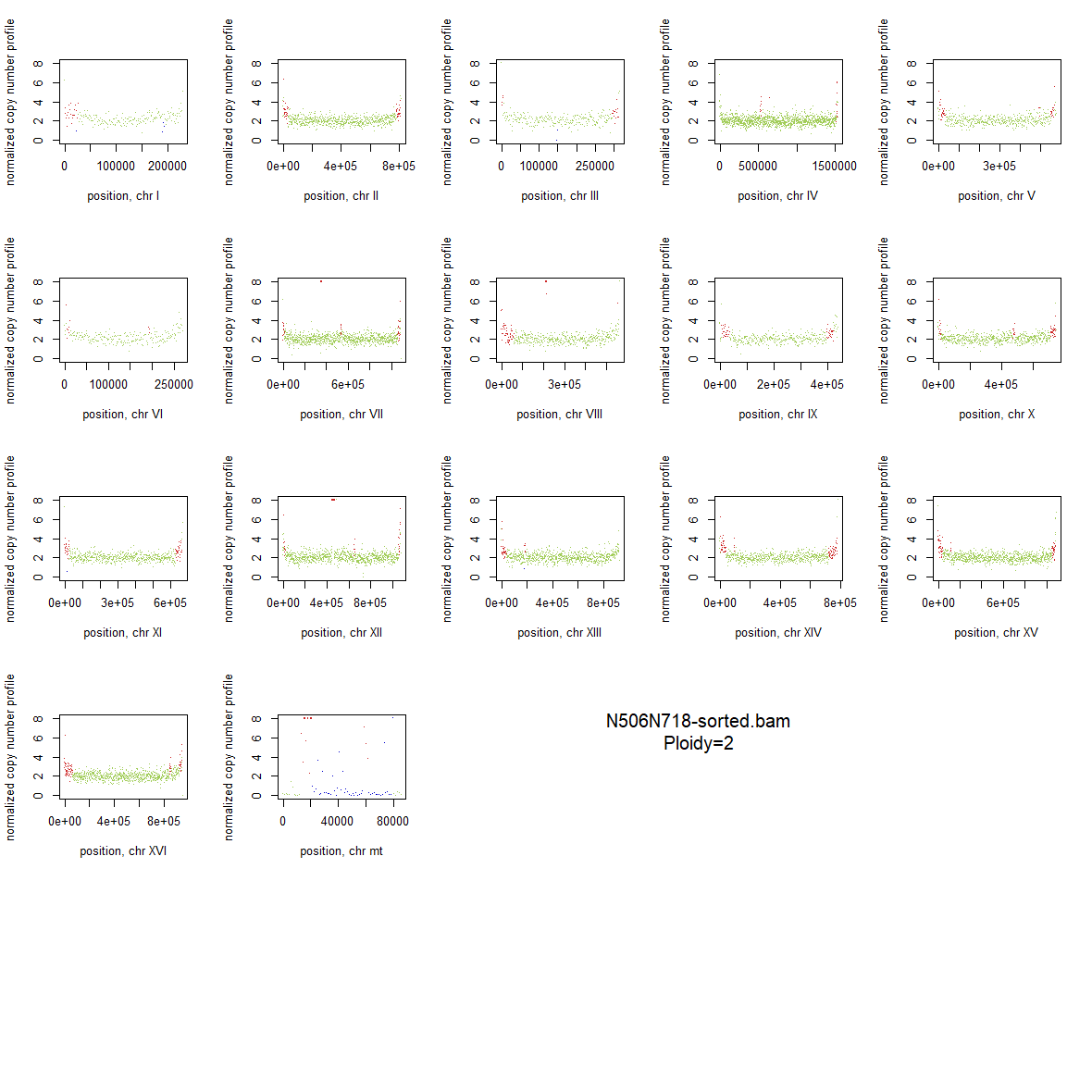

Supplement: Figure 2—source data 2. [file elife-79346-fig2-data2.zip › Figure2-source data 1/pACT-sec53-F126L/2x_F126L_18.png]

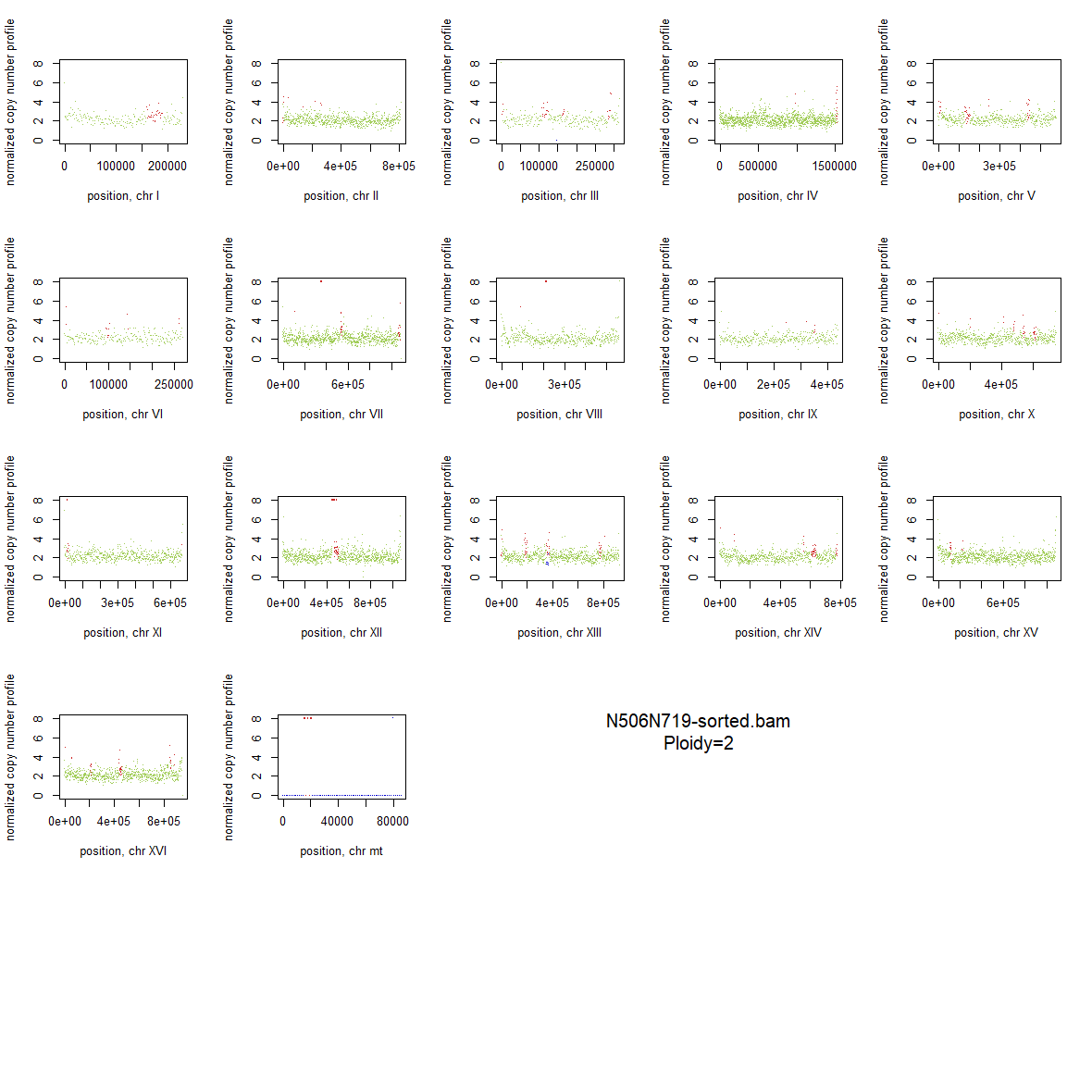

Supplement: Figure 2—source data 2. [file elife-79346-fig2-data2.zip › Figure2-source data 1/pACT-sec53-F126L/2x_F126L_19.png]

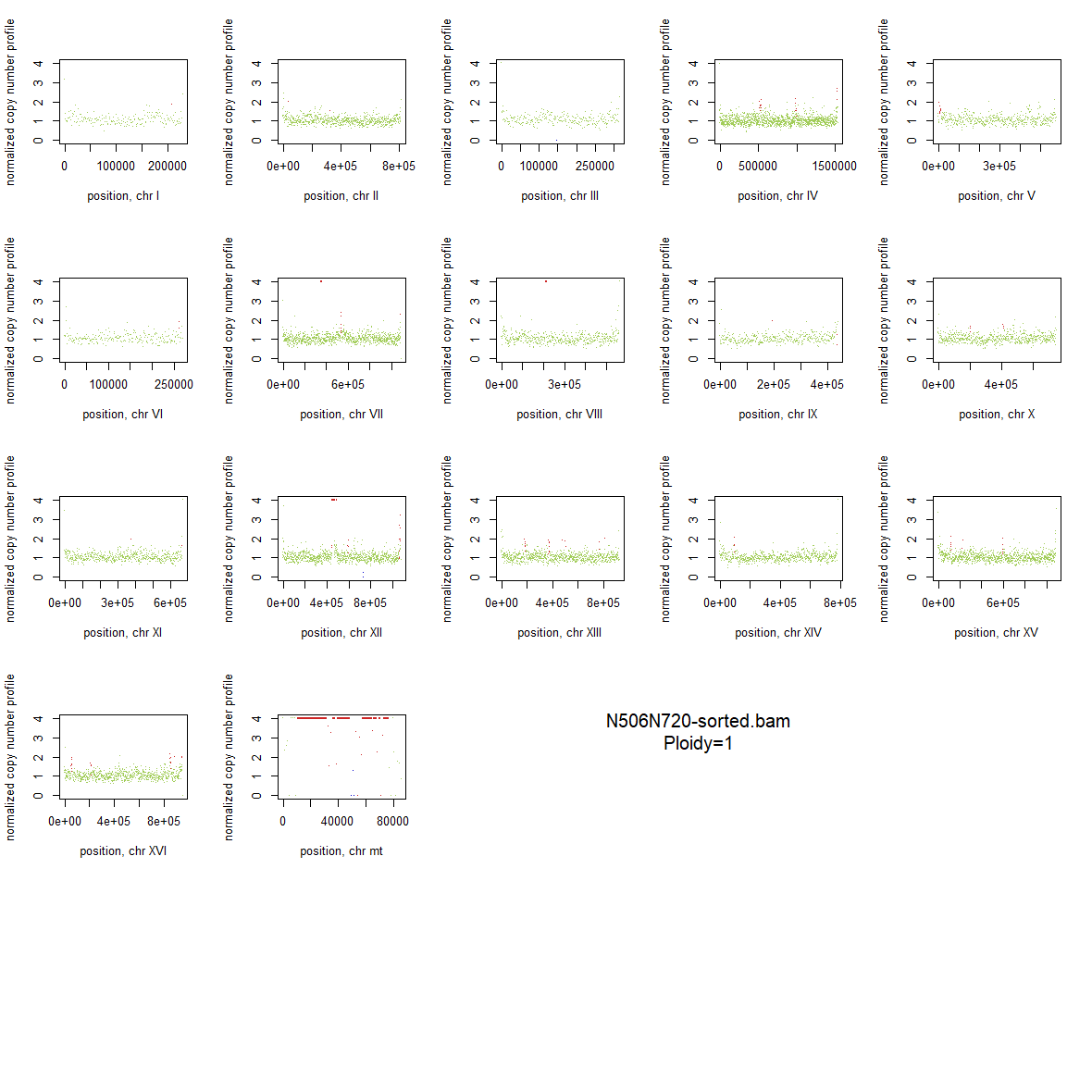

Supplement: Figure 2—source data 2. [file elife-79346-fig2-data2.zip › Figure2-source data 1/pACT-sec53-F126L/2x_F126L_20.png]

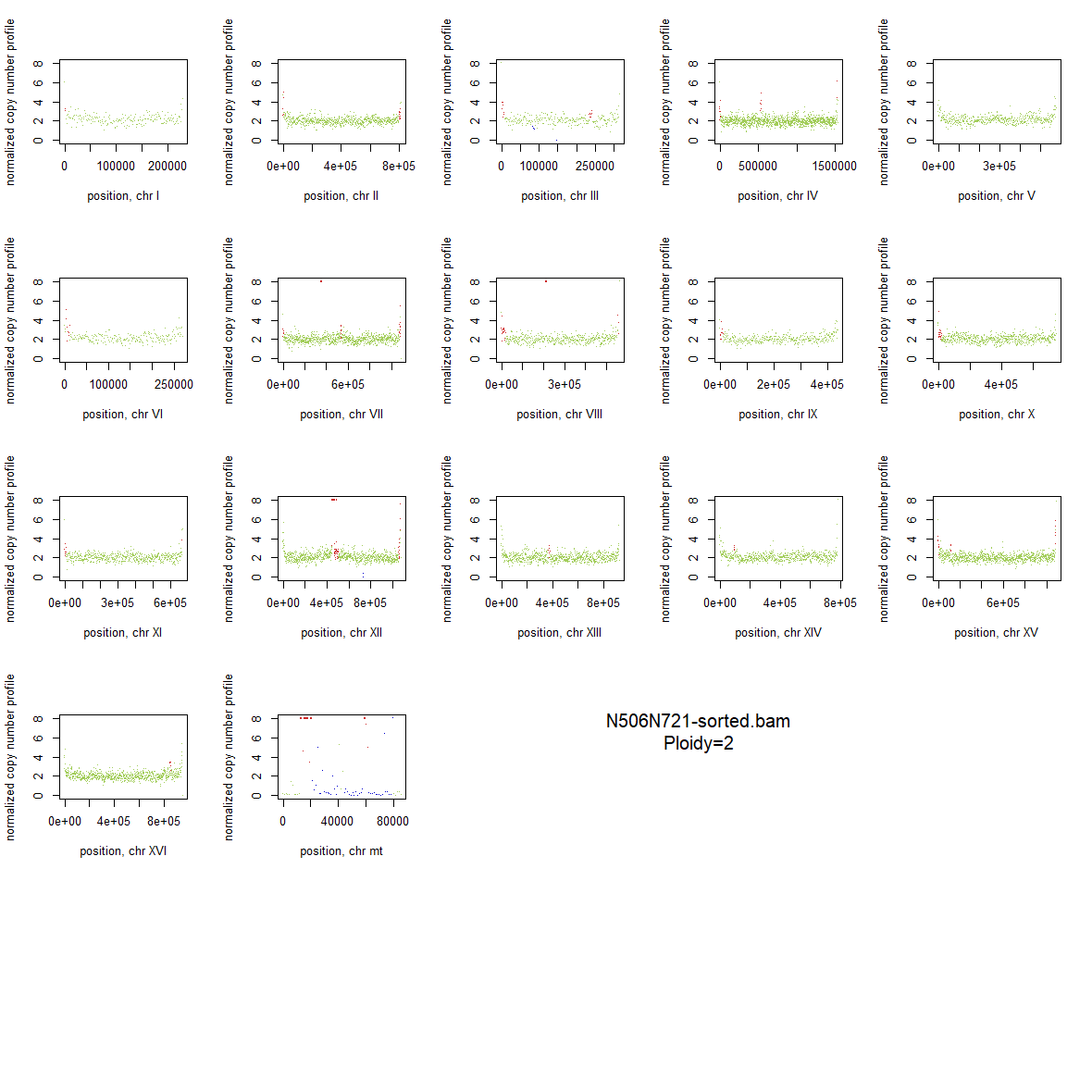

Supplement: Figure 2—source data 2. [file elife-79346-fig2-data2.zip › Figure2-source data 1/pACT-sec53-F126L/2x_F126L_21.png]

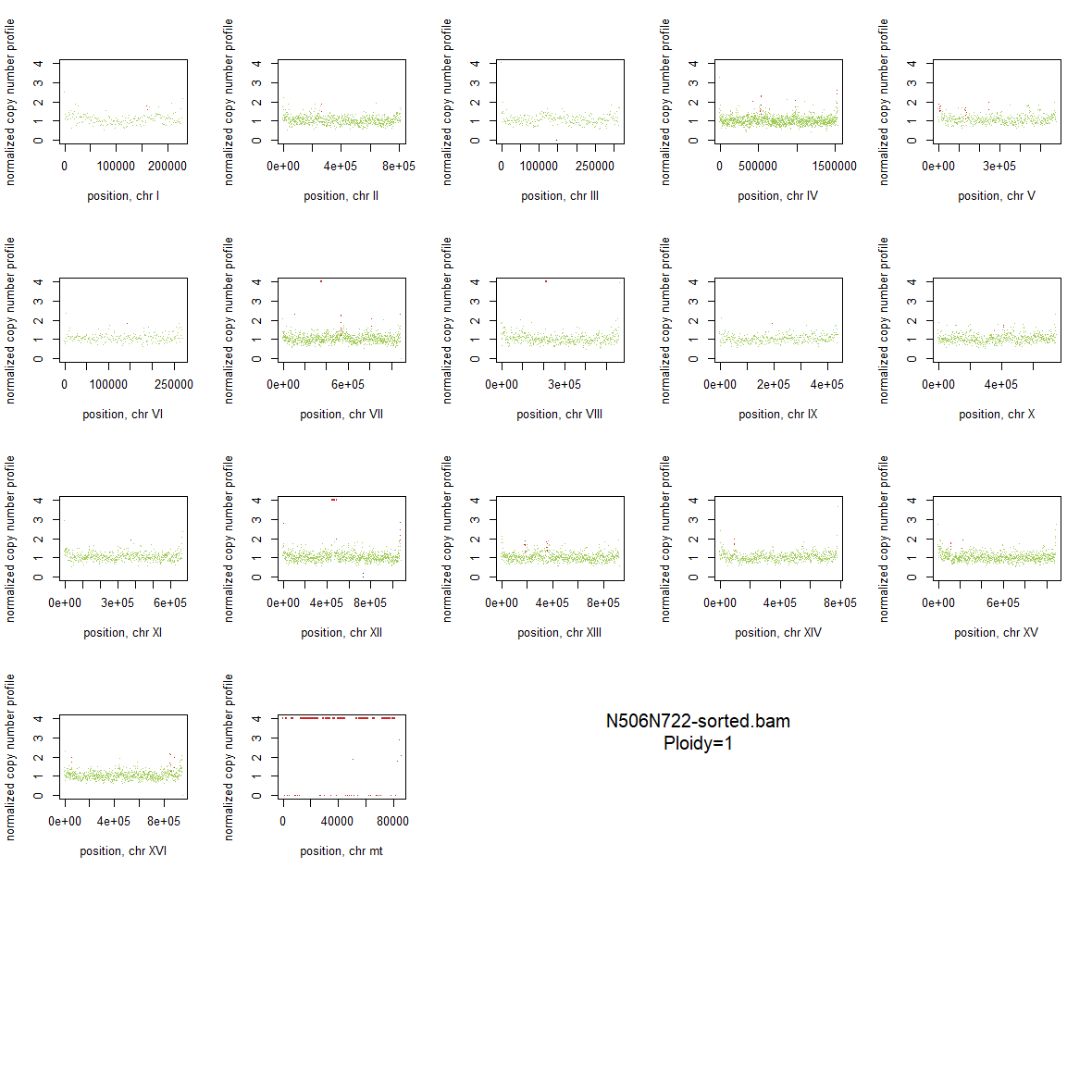

Supplement: Figure 2—source data 2. [file elife-79346-fig2-data2.zip › Figure2-source data 1/pACT-sec53-F126L/2x_F126L_22.png]

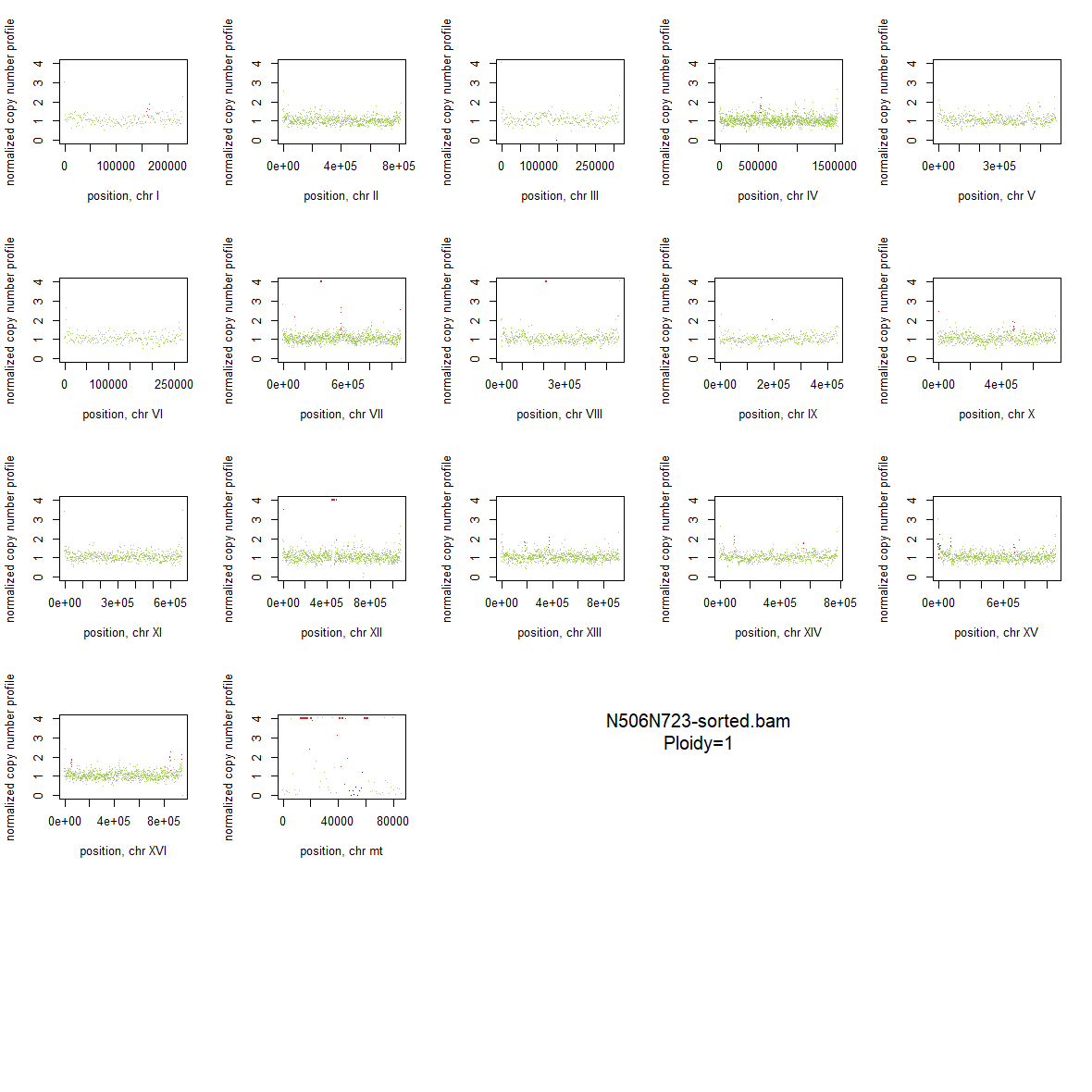

Supplement: Figure 2—source data 2. [file elife-79346-fig2-data2.zip › Figure2-source data 1/pACT-sec53-F126L/2x_F126L_23.png]

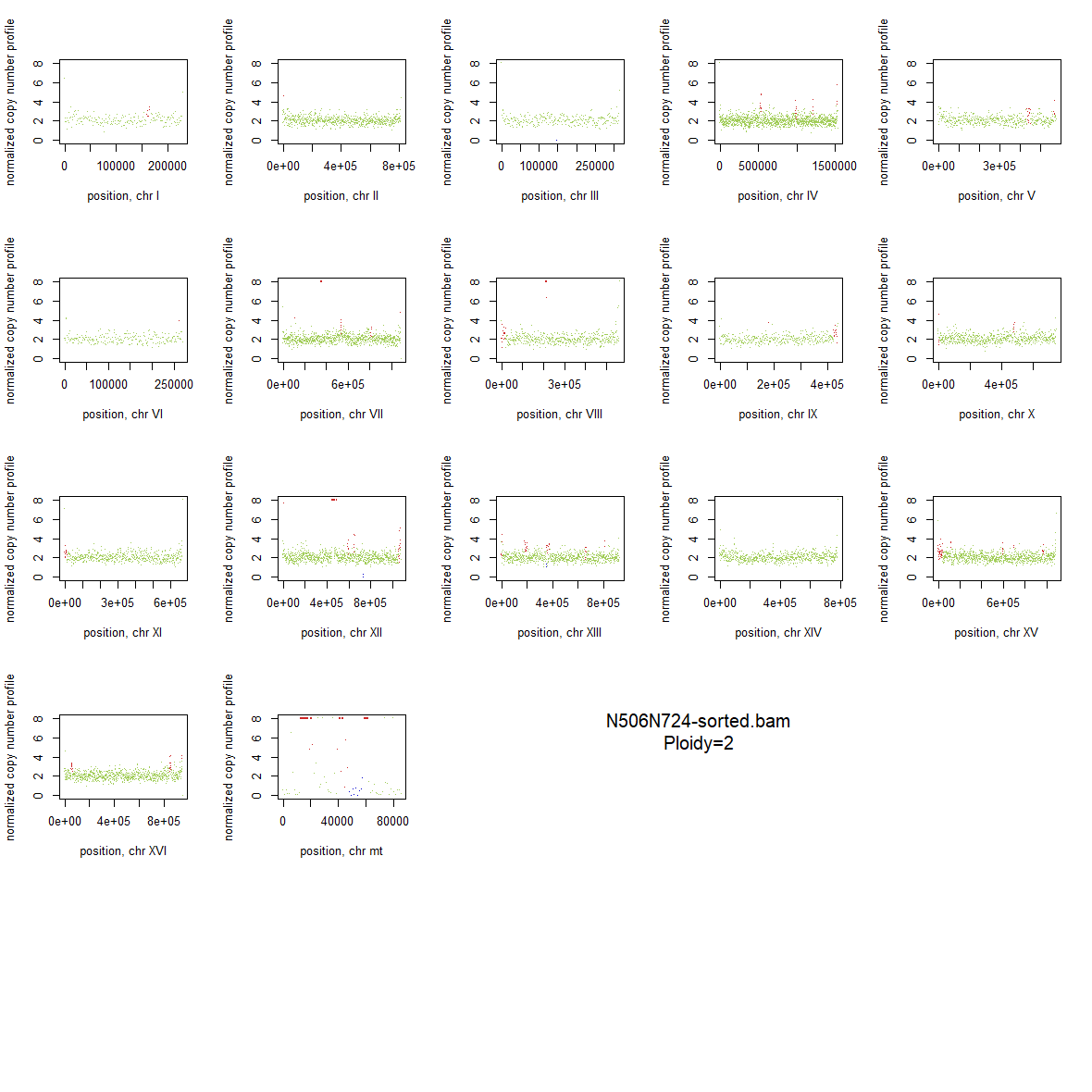

Supplement: Figure 2—source data 2. [file elife-79346-fig2-data2.zip › Figure2-source data 1/pACT-sec53-F126L/2x_F126L_24.png]

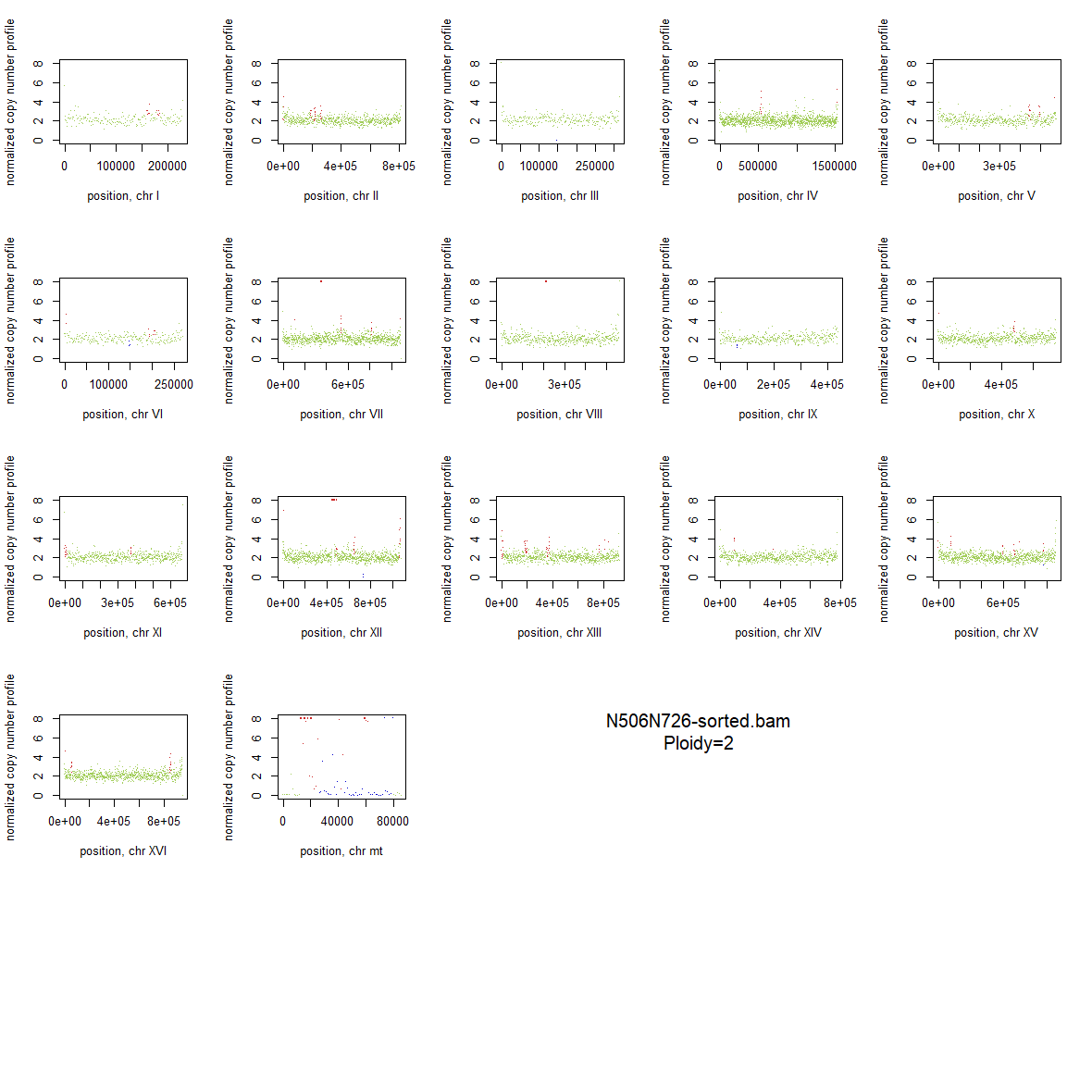

Supplement: Figure 2—source data 2. [file elife-79346-fig2-data2.zip › Figure2-source data 1/pACT-sec53-F126L/2x_F126L_25.png]

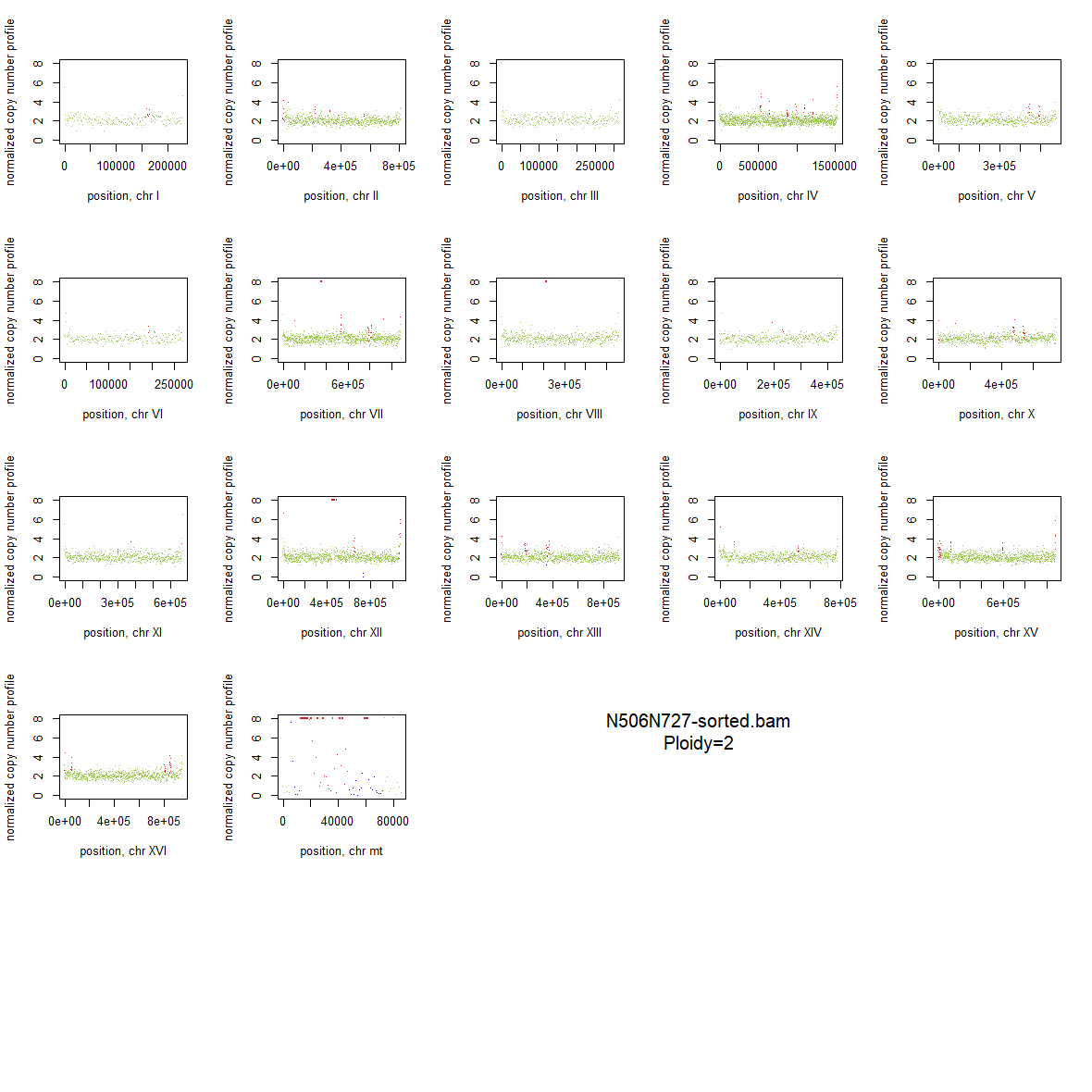

Supplement: Figure 2—source data 2. [file elife-79346-fig2-data2.zip › Figure2-source data 1/pACT-sec53-F126L/2x_F126L_26.png]

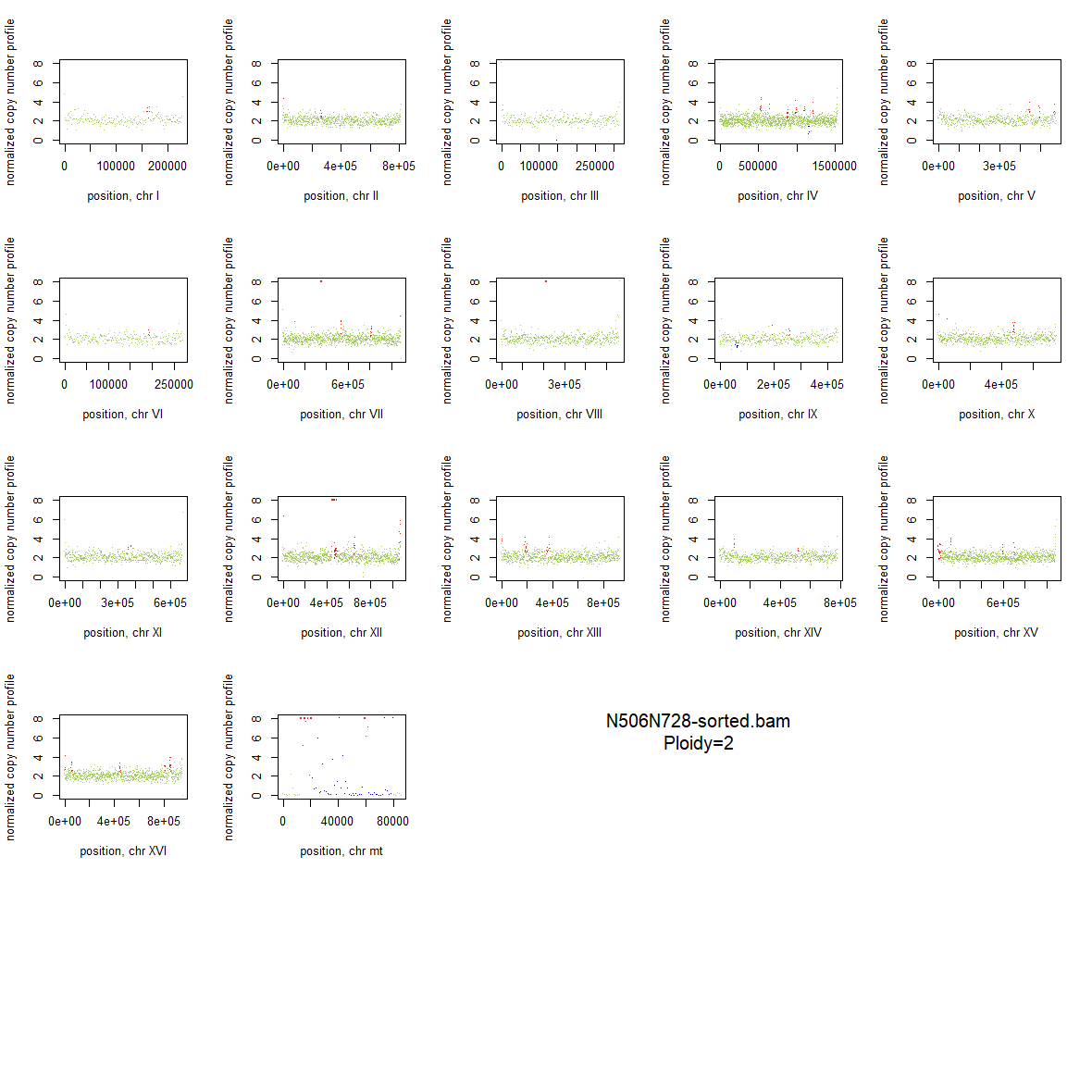

Supplement: Figure 2—source data 2. [file elife-79346-fig2-data2.zip › Figure2-source data 1/pACT-sec53-F126L/2x_F126L_27.png]

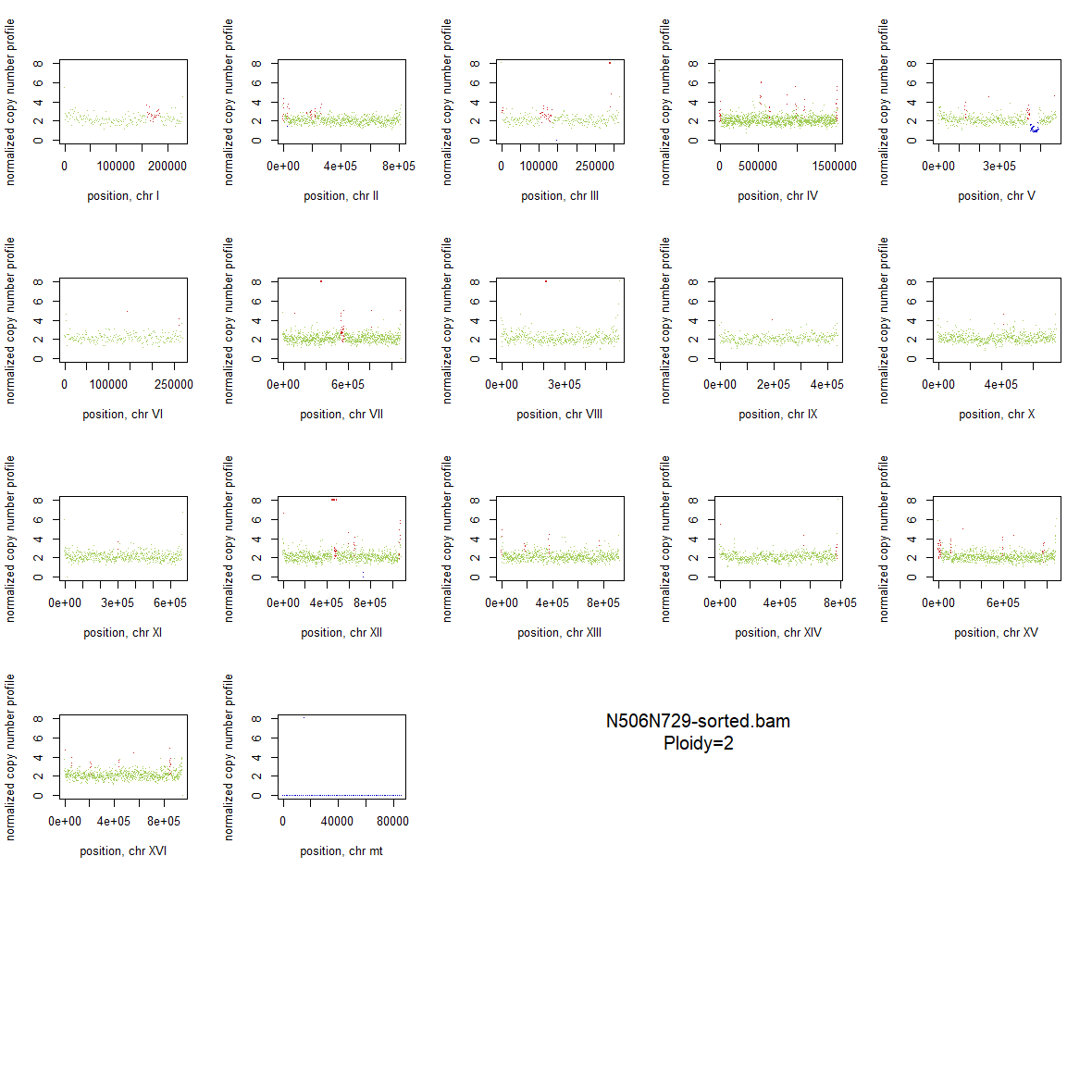

Supplement: Figure 2—source data 2. [file elife-79346-fig2-data2.zip › Figure2-source data 1/pACT-sec53-F126L/2x_F126L_28.png]

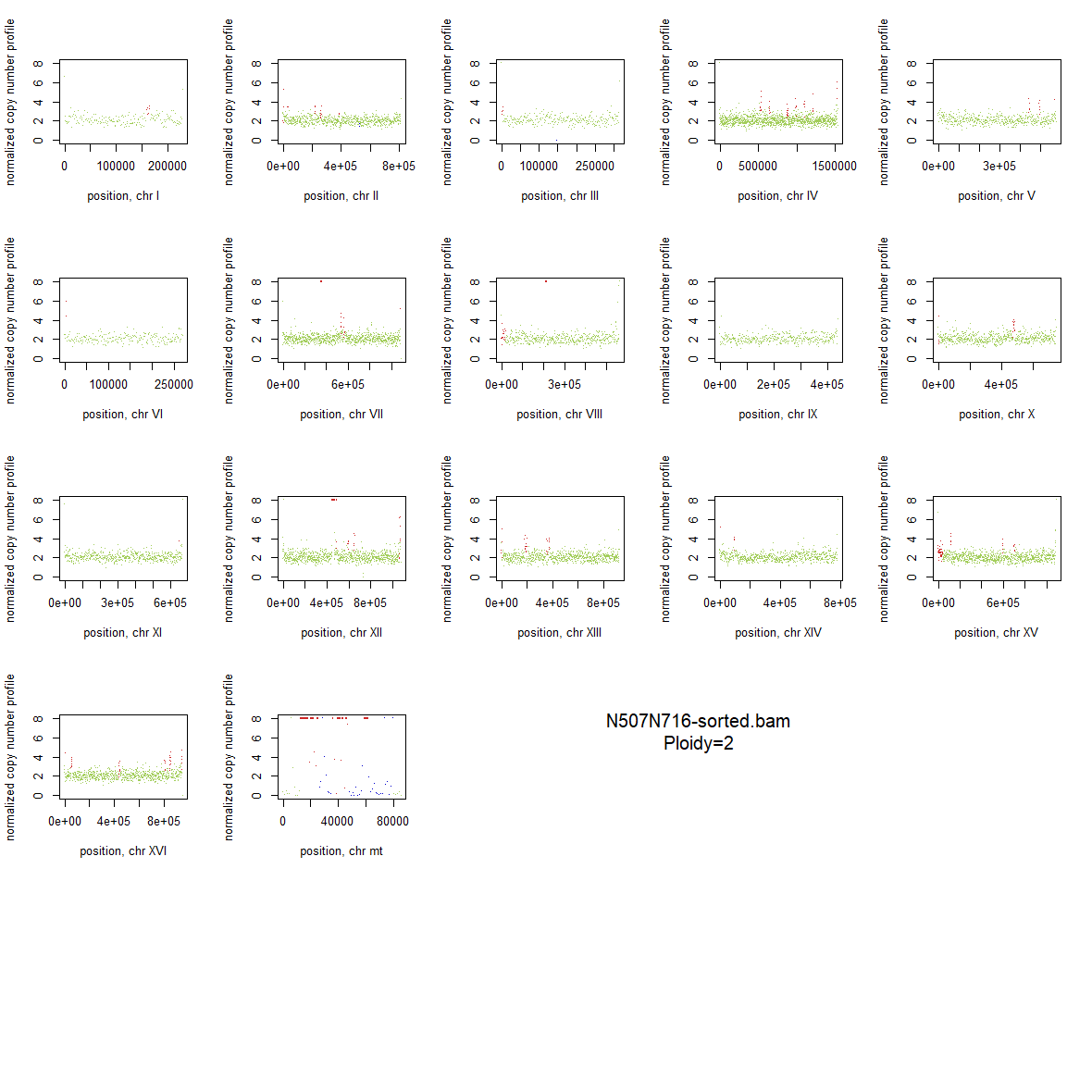

Supplement: Figure 2—source data 2. [file elife-79346-fig2-data2.zip › Figure2-source data 1/pACT-sec53-F126L/2x_F126L_29.png]

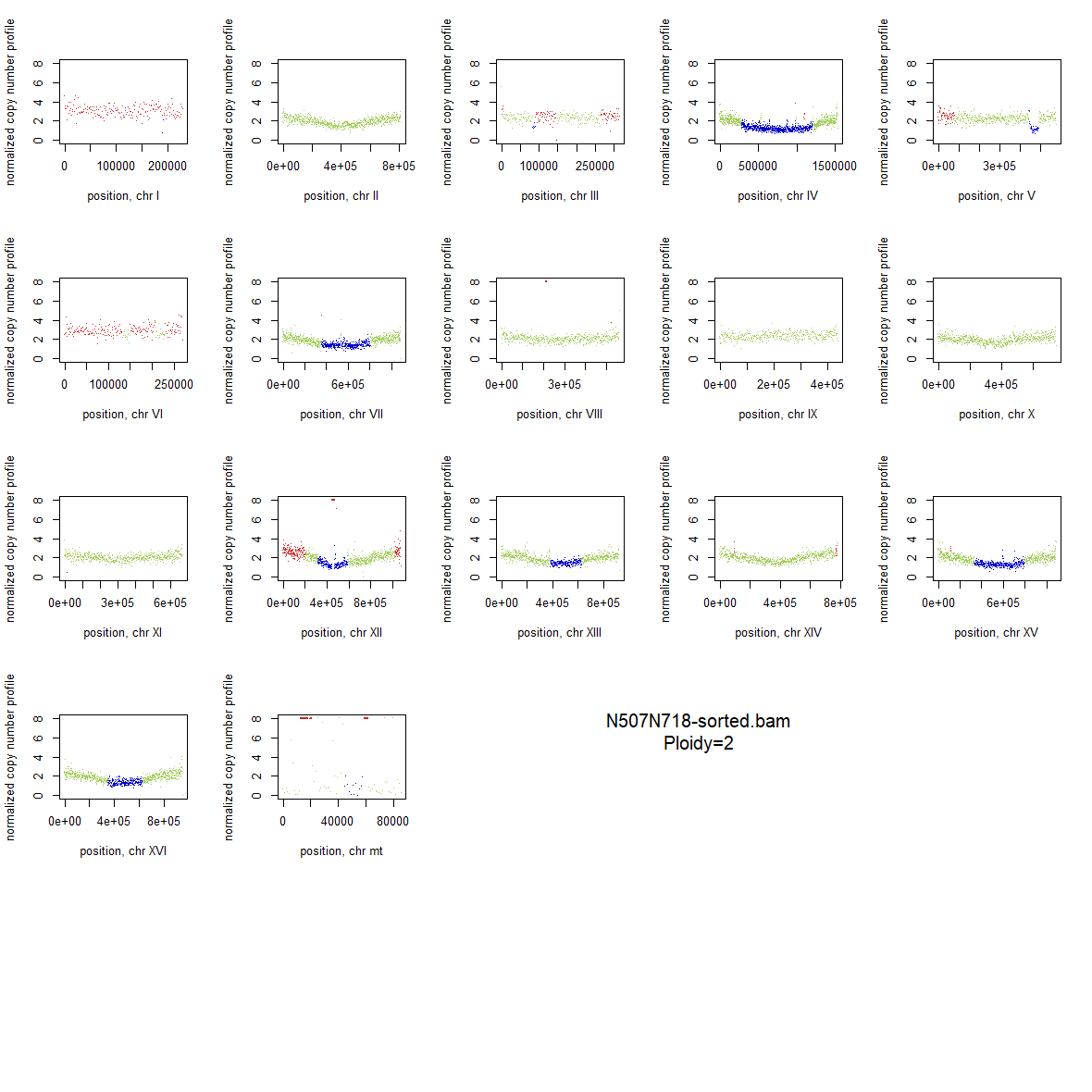

Supplement: Figure 2—source data 2. [file elife-79346-fig2-data2.zip › Figure2-source data 1/pACT-sec53-F126L/2x_F126L_30.png]

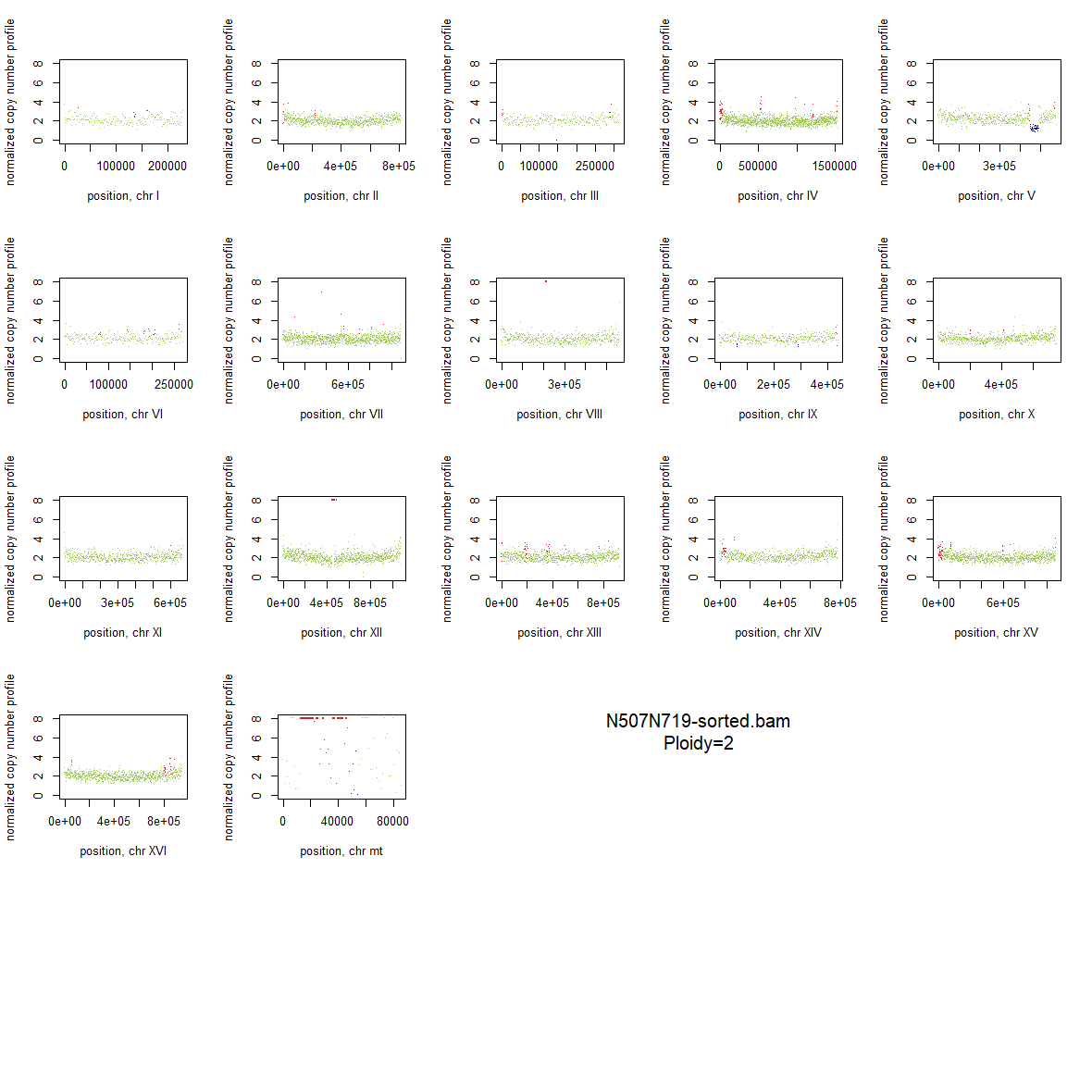

Supplement: Figure 2—source data 2. [file elife-79346-fig2-data2.zip › Figure2-source data 1/pACT-sec53-F126L/2x_F126L_31.png]

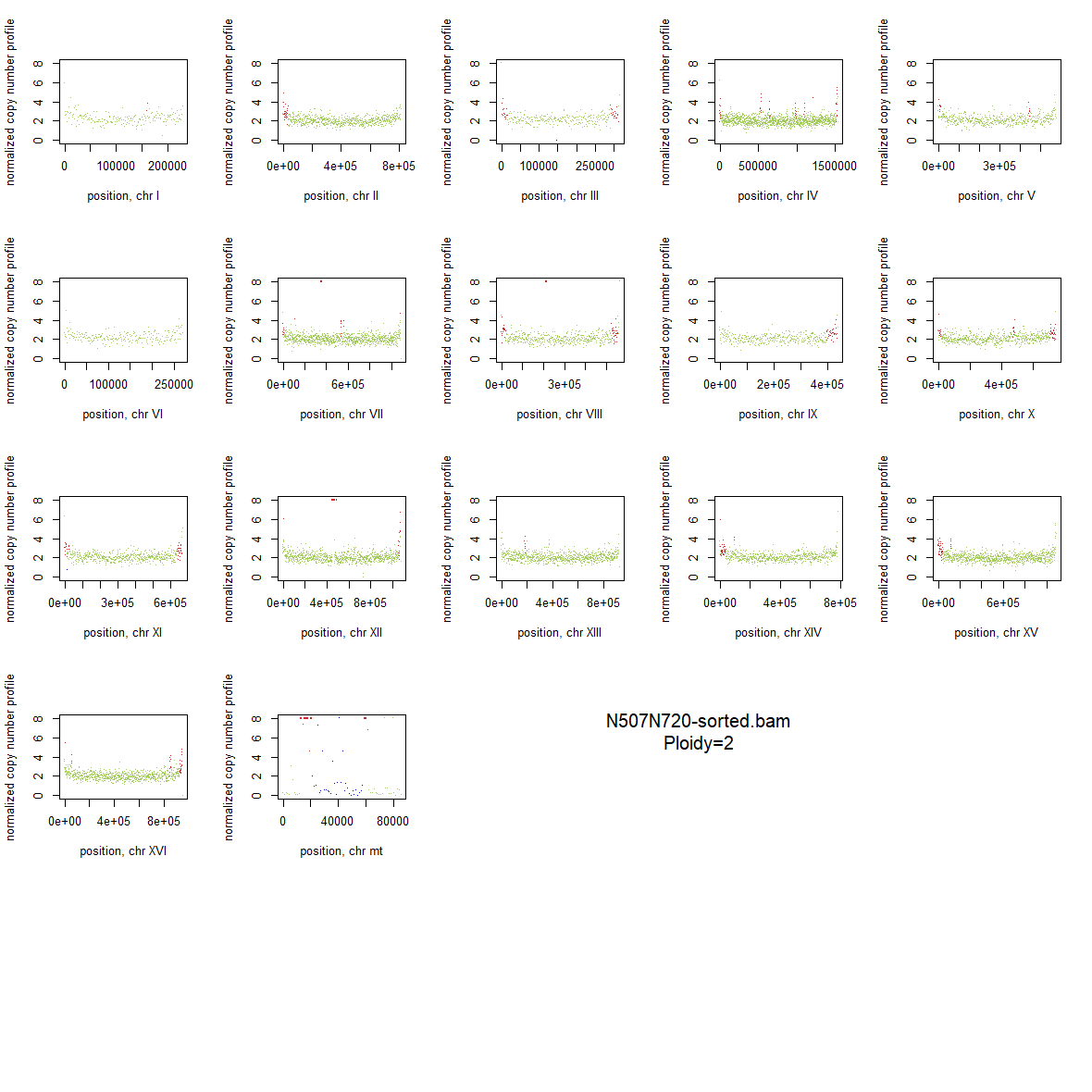

Supplement: Figure 2—source data 2. [file elife-79346-fig2-data2.zip › Figure2-source data 1/pACT-sec53-F126L/2x_F126L_32.png]

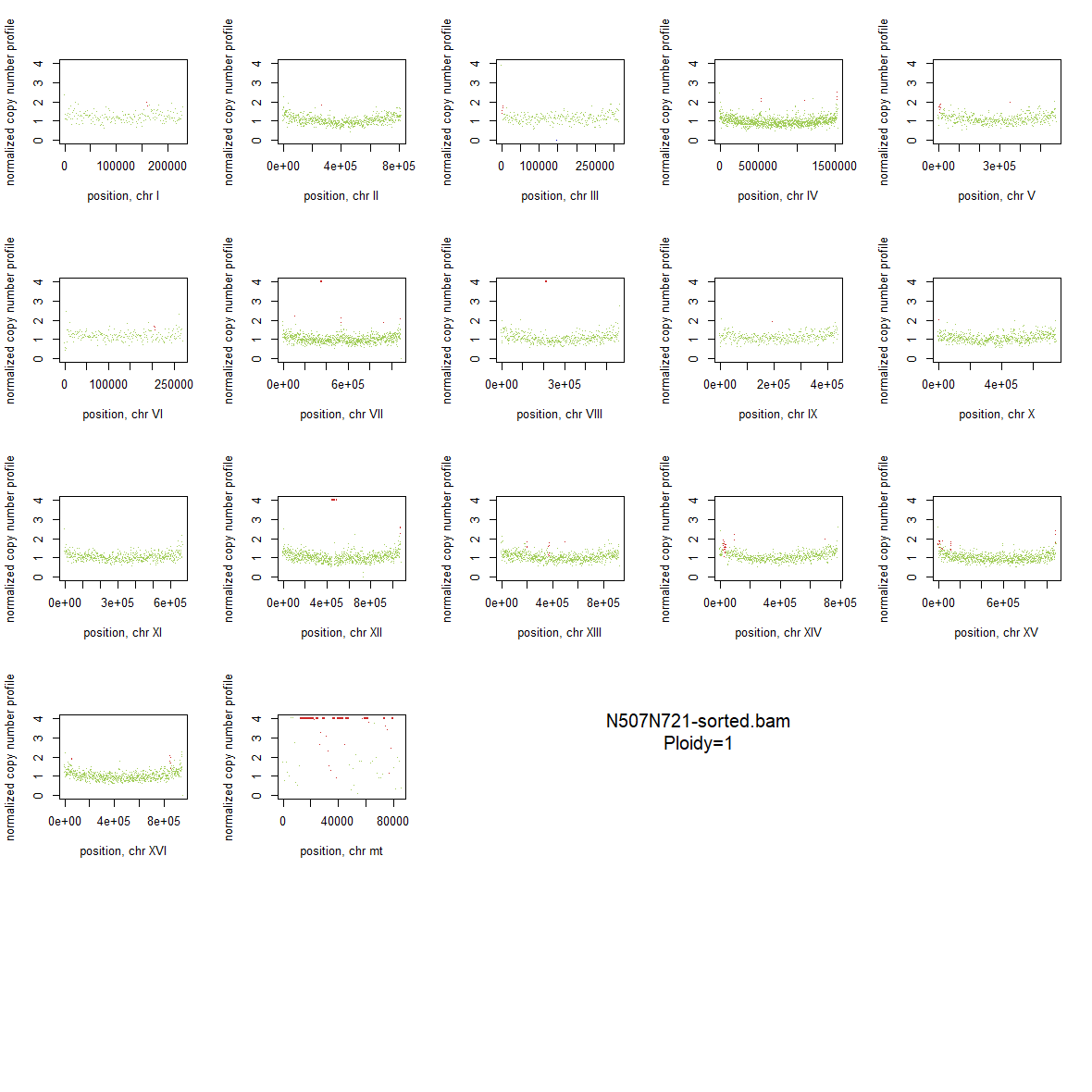

Supplement: Figure 2—source data 2. [file elife-79346-fig2-data2.zip › Figure2-source data 1/pACT-sec53-F126L/2x_F126L_33.png]

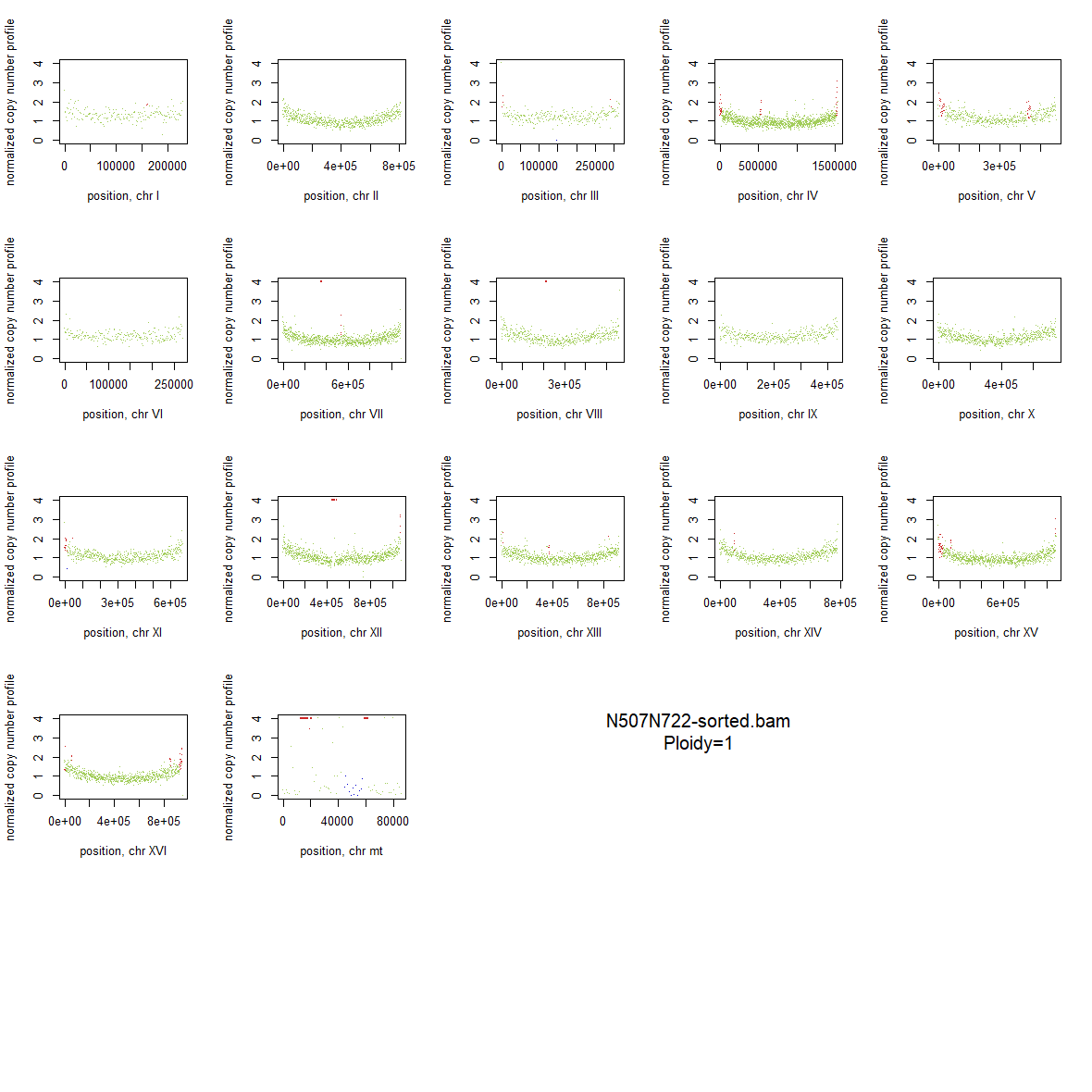

Supplement: Figure 2—source data 2. [file elife-79346-fig2-data2.zip › Figure2-source data 1/pACT-sec53-F126L/2x_F126L_34.png]

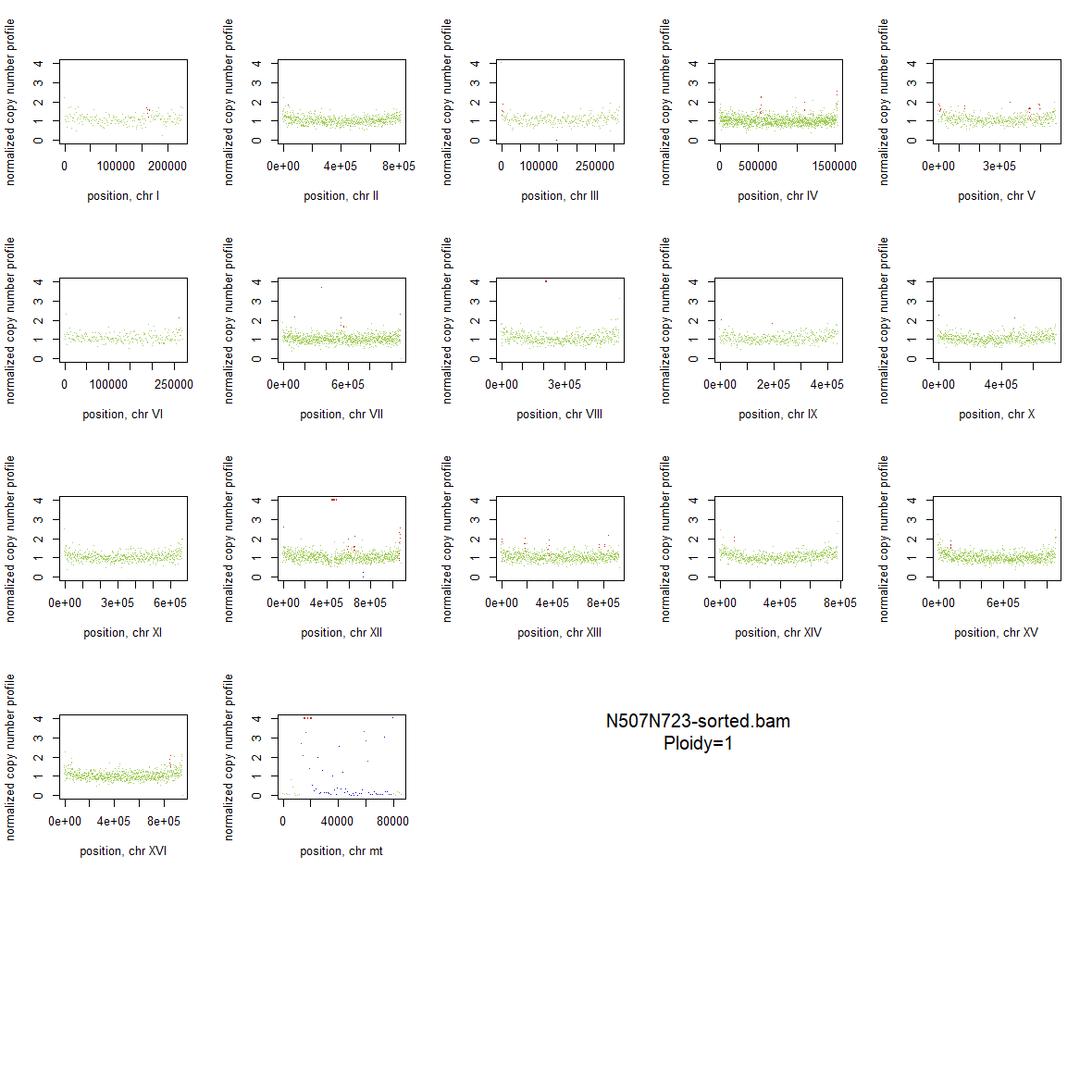

Supplement: Figure 2—source data 2. [file elife-79346-fig2-data2.zip › Figure2-source data 1/pACT-sec53-F126L/2x_F126L_35.png]

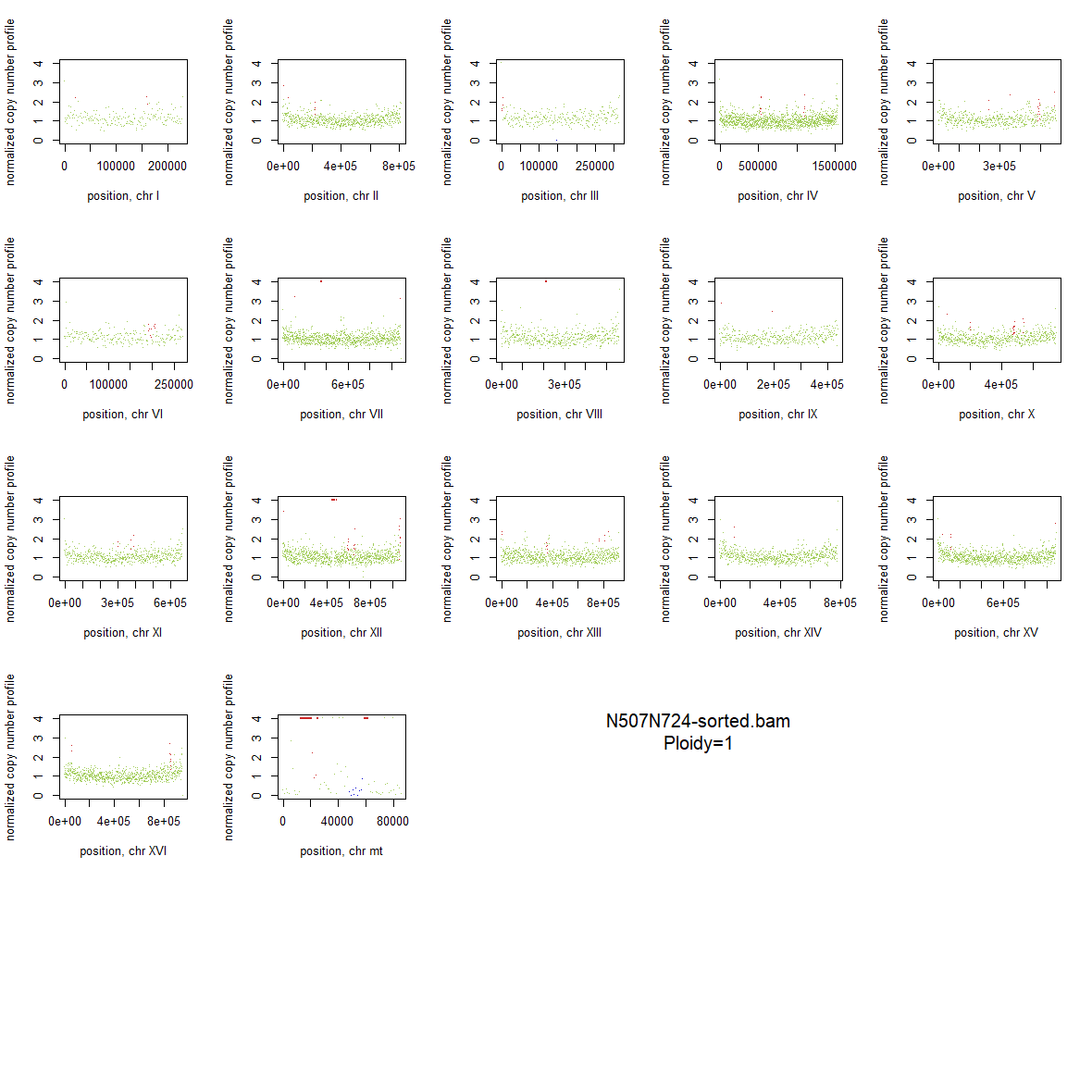

Supplement: Figure 2—source data 2. [file elife-79346-fig2-data2.zip › Figure2-source data 1/pACT-sec53-F126L/2x_F126L_36.png]

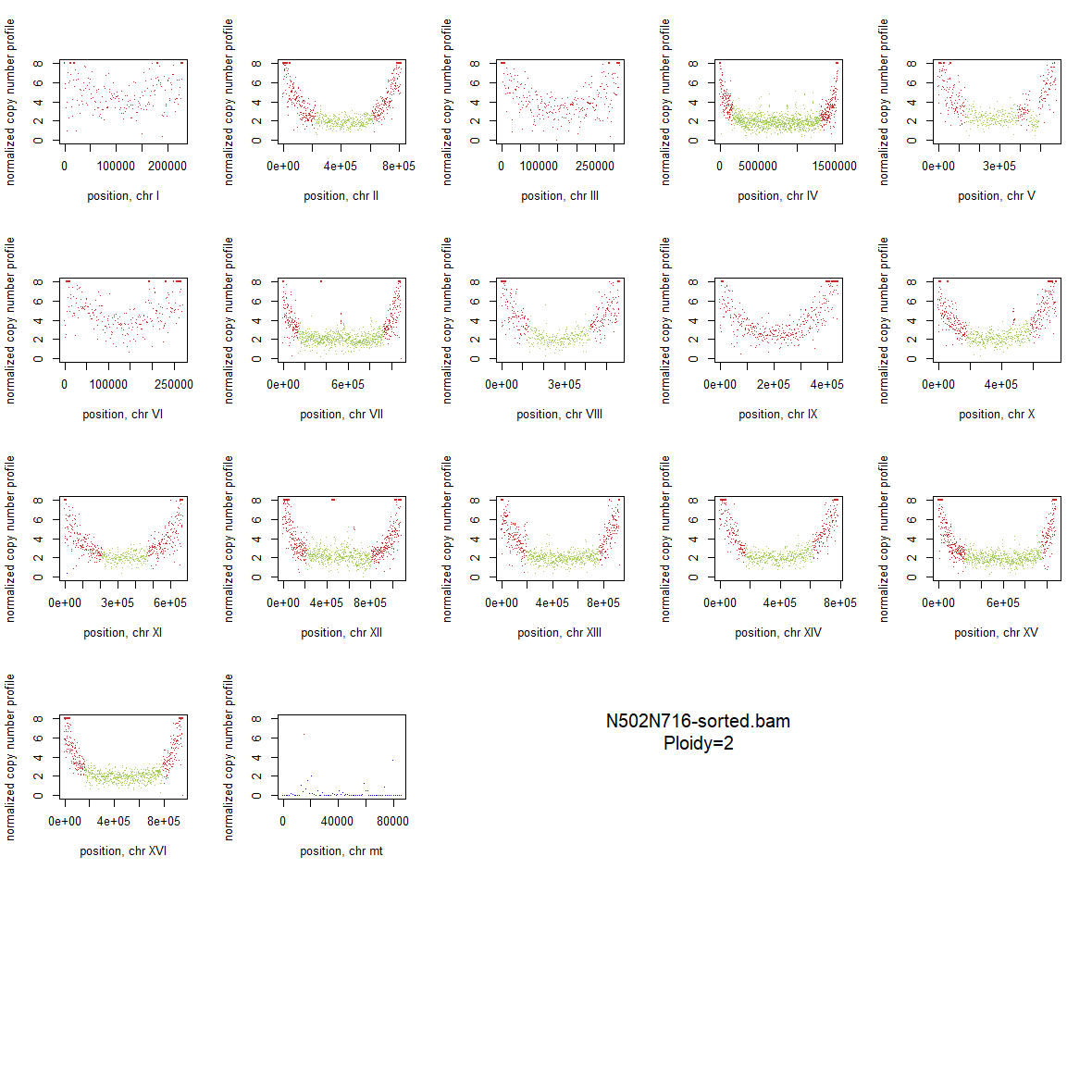

Supplement: Figure 2—source data 2. [file elife-79346-fig2-data2.zip › Figure2-source data 1/pSEC53-SEC53-WT/1x_Wildtype_01.png]

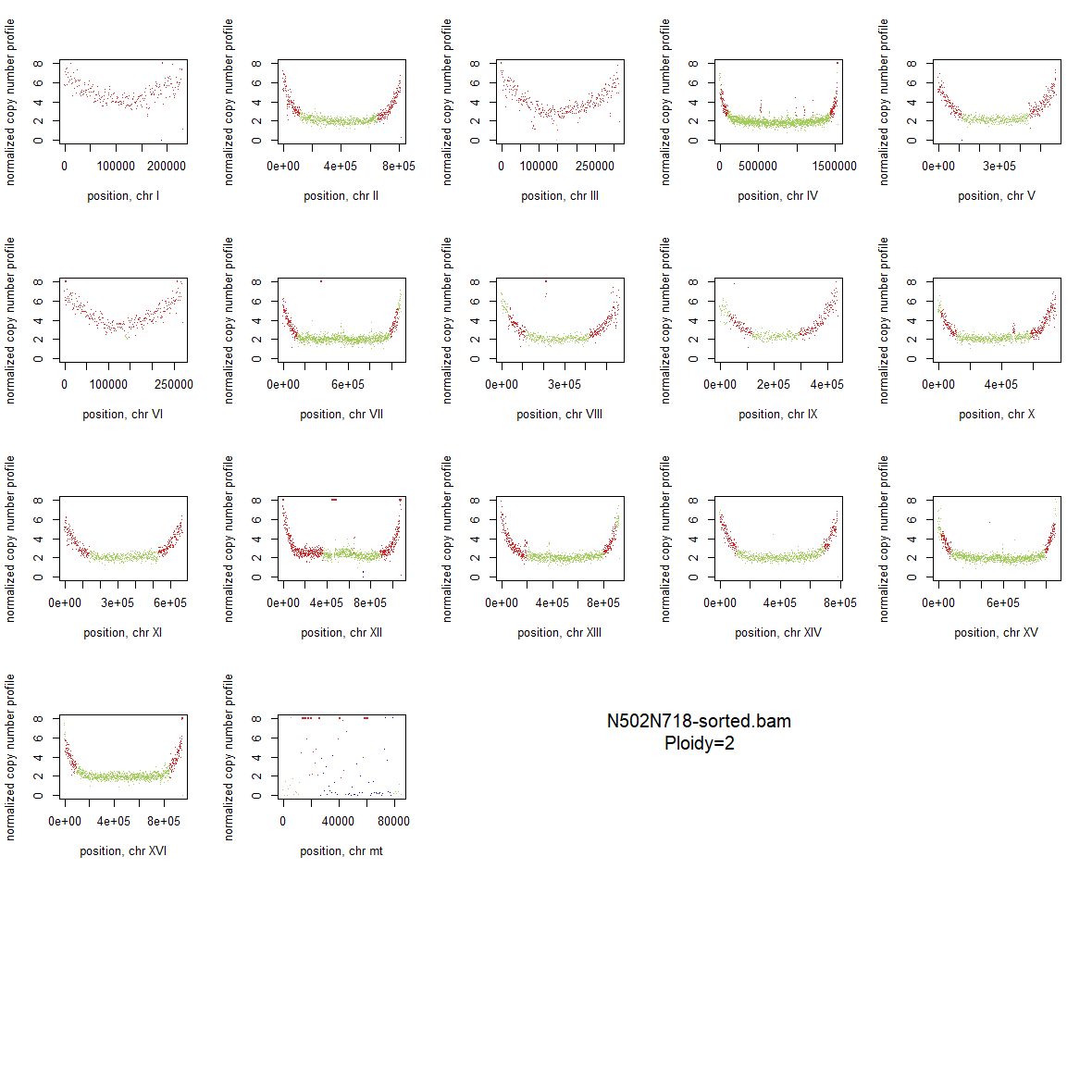

Supplement: Figure 2—source data 2. [file elife-79346-fig2-data2.zip › Figure2-source data 1/pSEC53-SEC53-WT/1x_Wildtype_02.png]

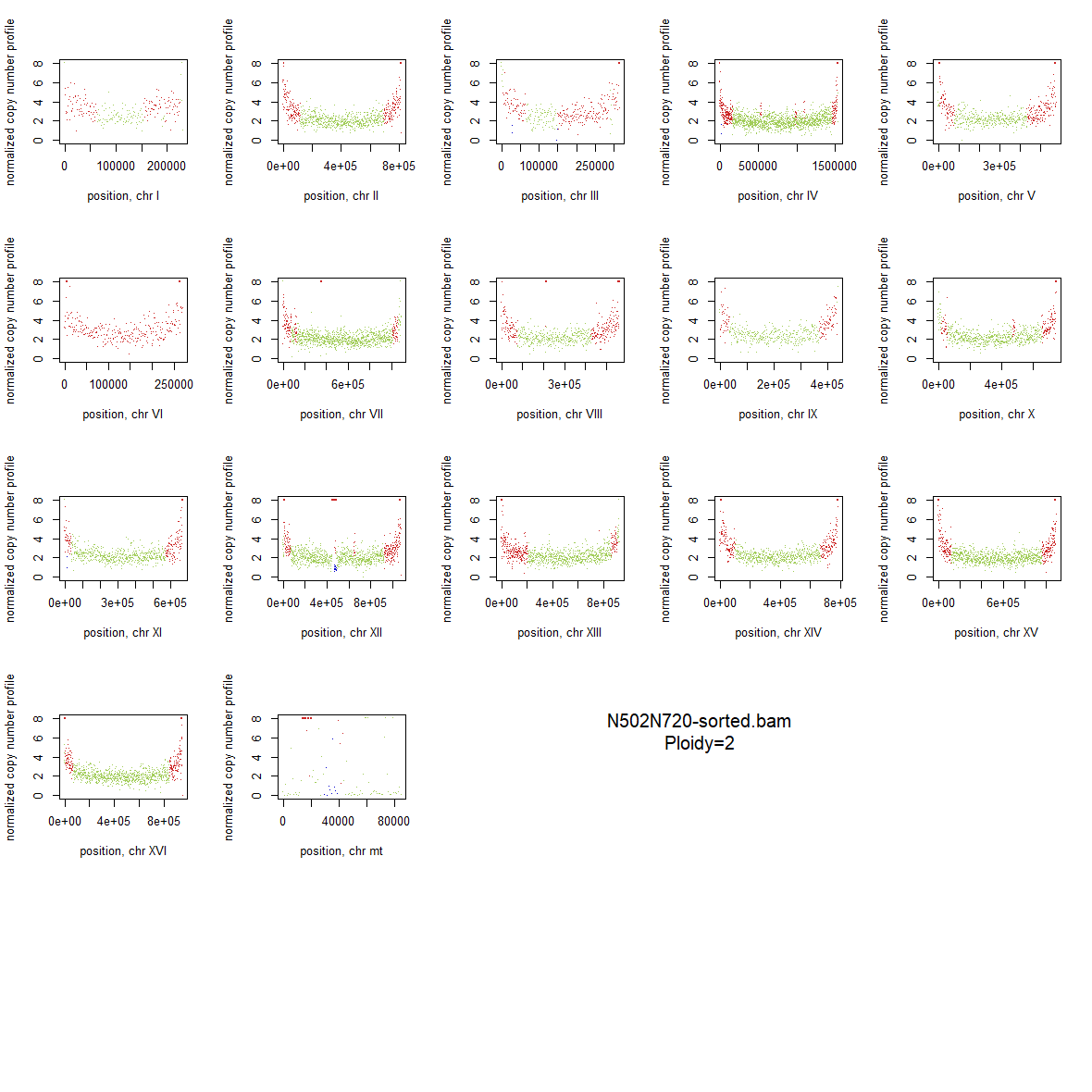

Supplement: Figure 2—source data 2. [file elife-79346-fig2-data2.zip › Figure2-source data 1/pSEC53-SEC53-WT/1x_Wildtype_03.png]

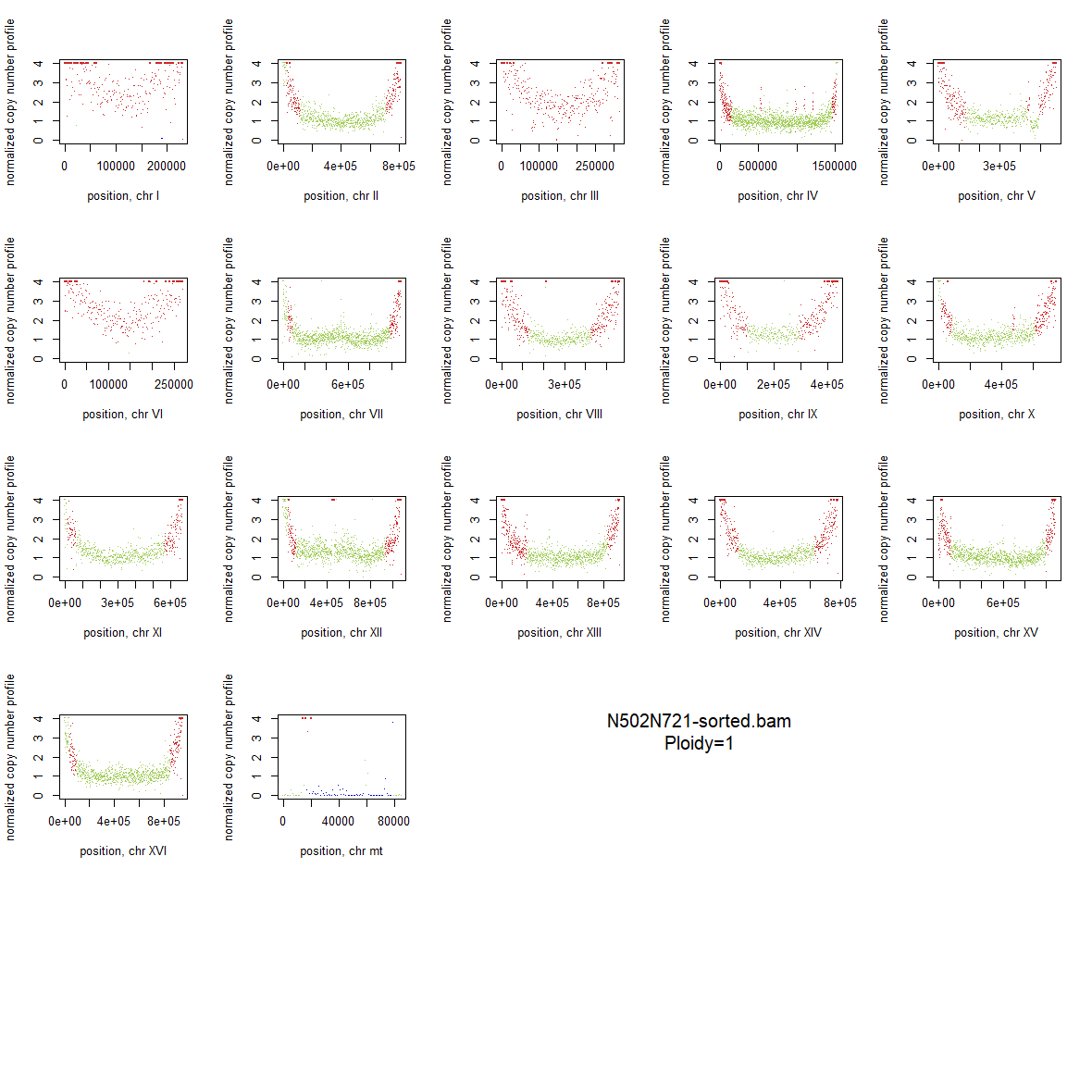

Supplement: Figure 2—source data 2. [file elife-79346-fig2-data2.zip › Figure2-source data 1/pSEC53-SEC53-WT/1x_Wildtype_04.png]

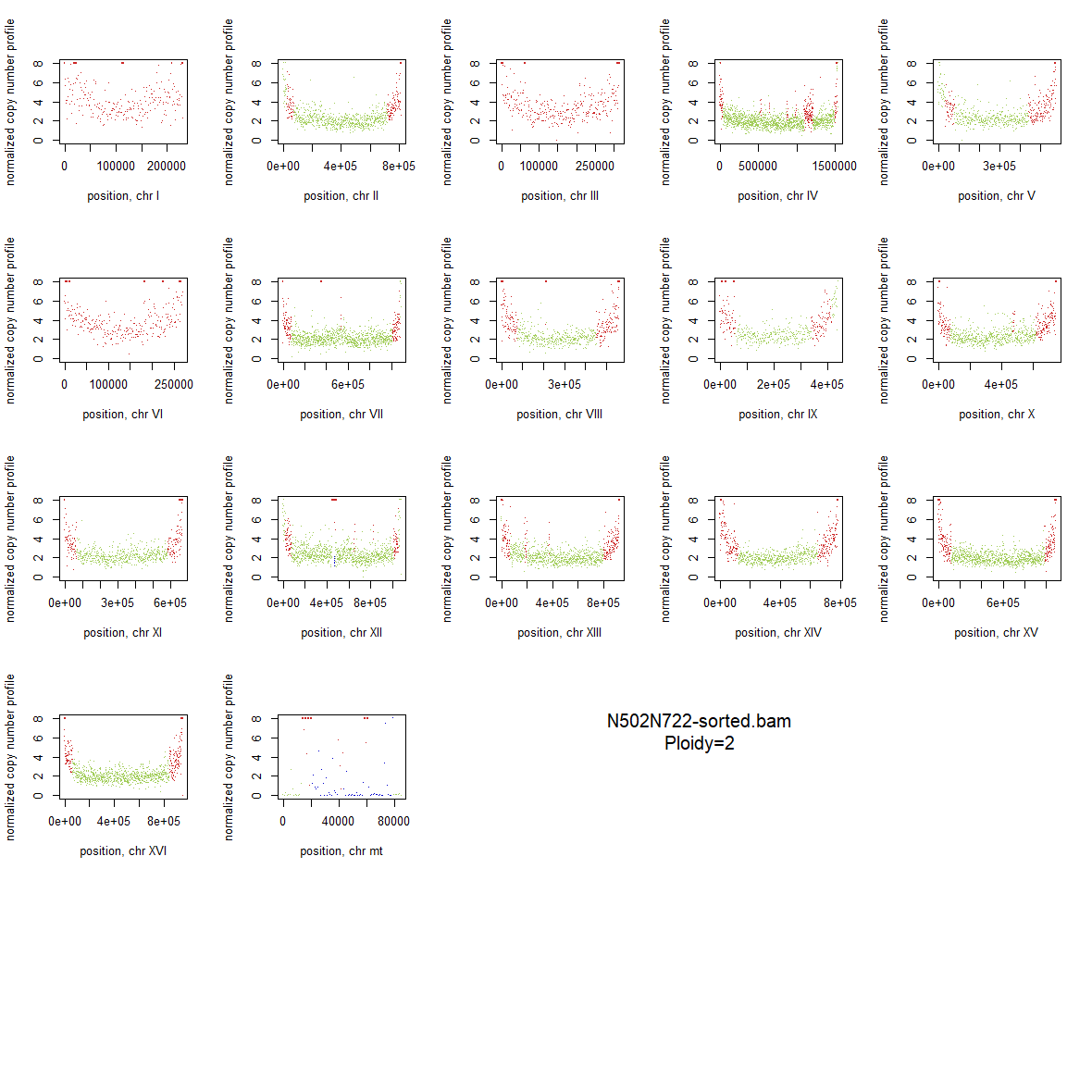

Supplement: Figure 2—source data 2. [file elife-79346-fig2-data2.zip › Figure2-source data 1/pSEC53-SEC53-WT/1x_Wildtype_05.png]

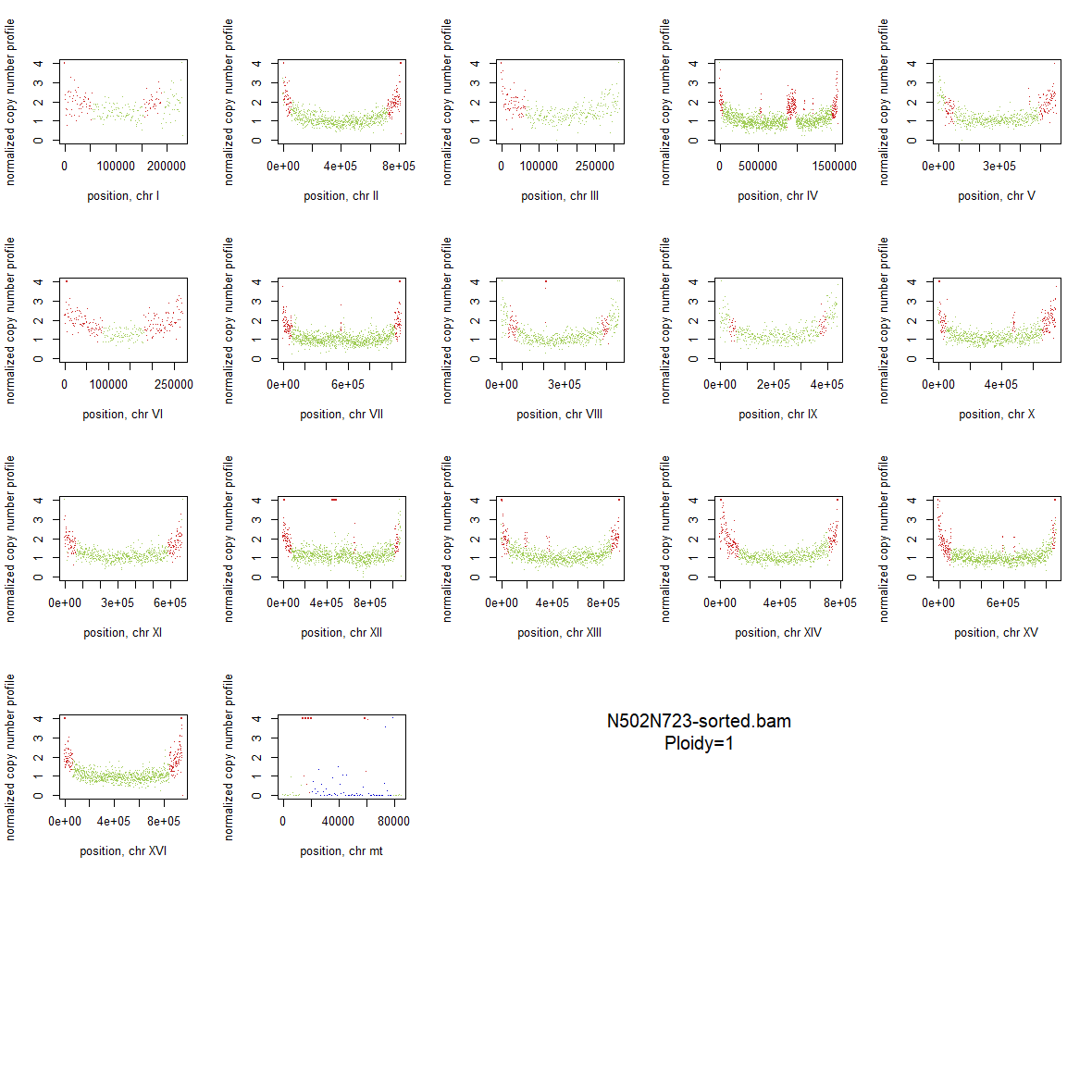

Supplement: Figure 2—source data 2. [file elife-79346-fig2-data2.zip › Figure2-source data 1/pSEC53-SEC53-WT/1x_Wildtype_06.png]

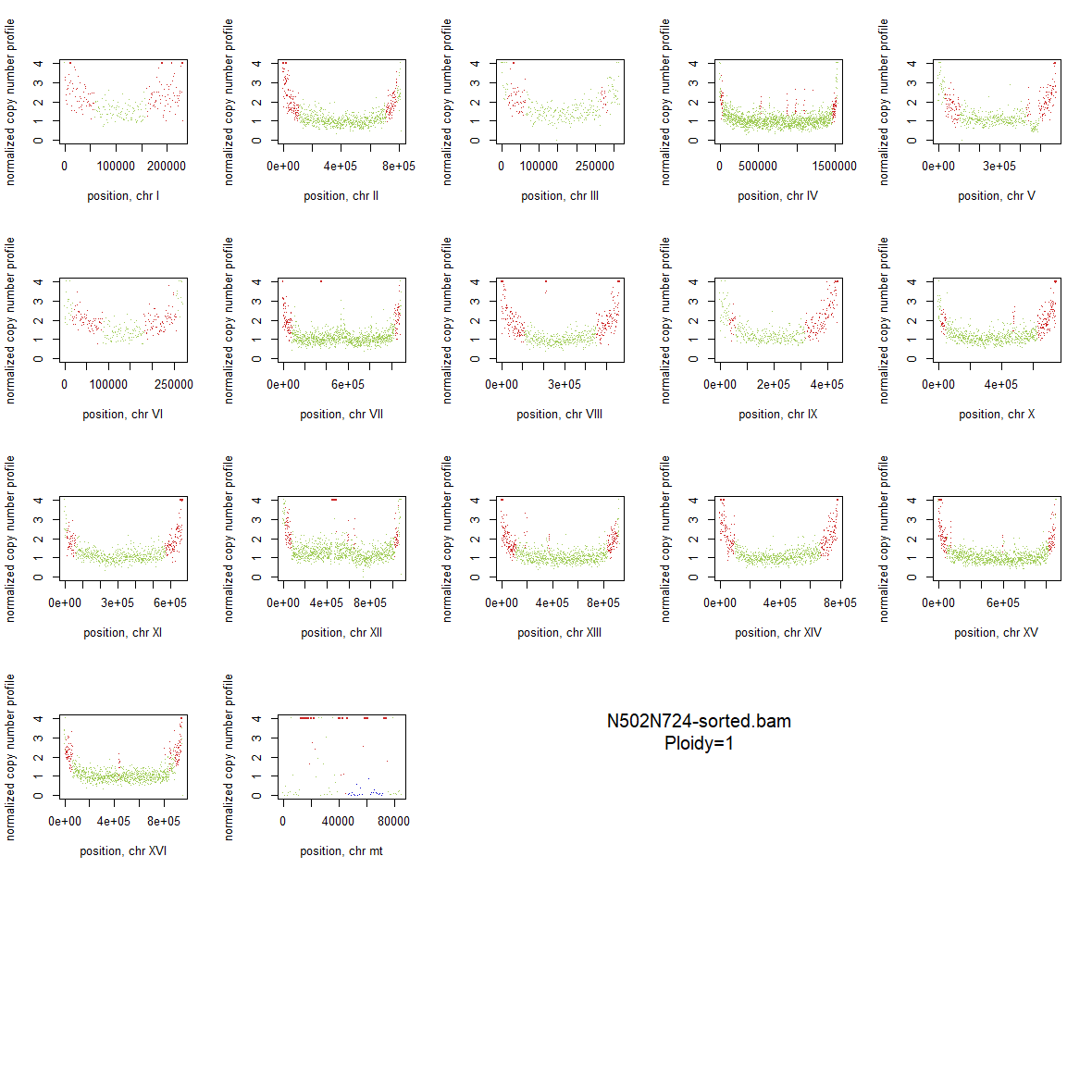

Supplement: Figure 2—source data 2. [file elife-79346-fig2-data2.zip › Figure2-source data 1/pSEC53-SEC53-WT/1x_Wildtype_07.png]

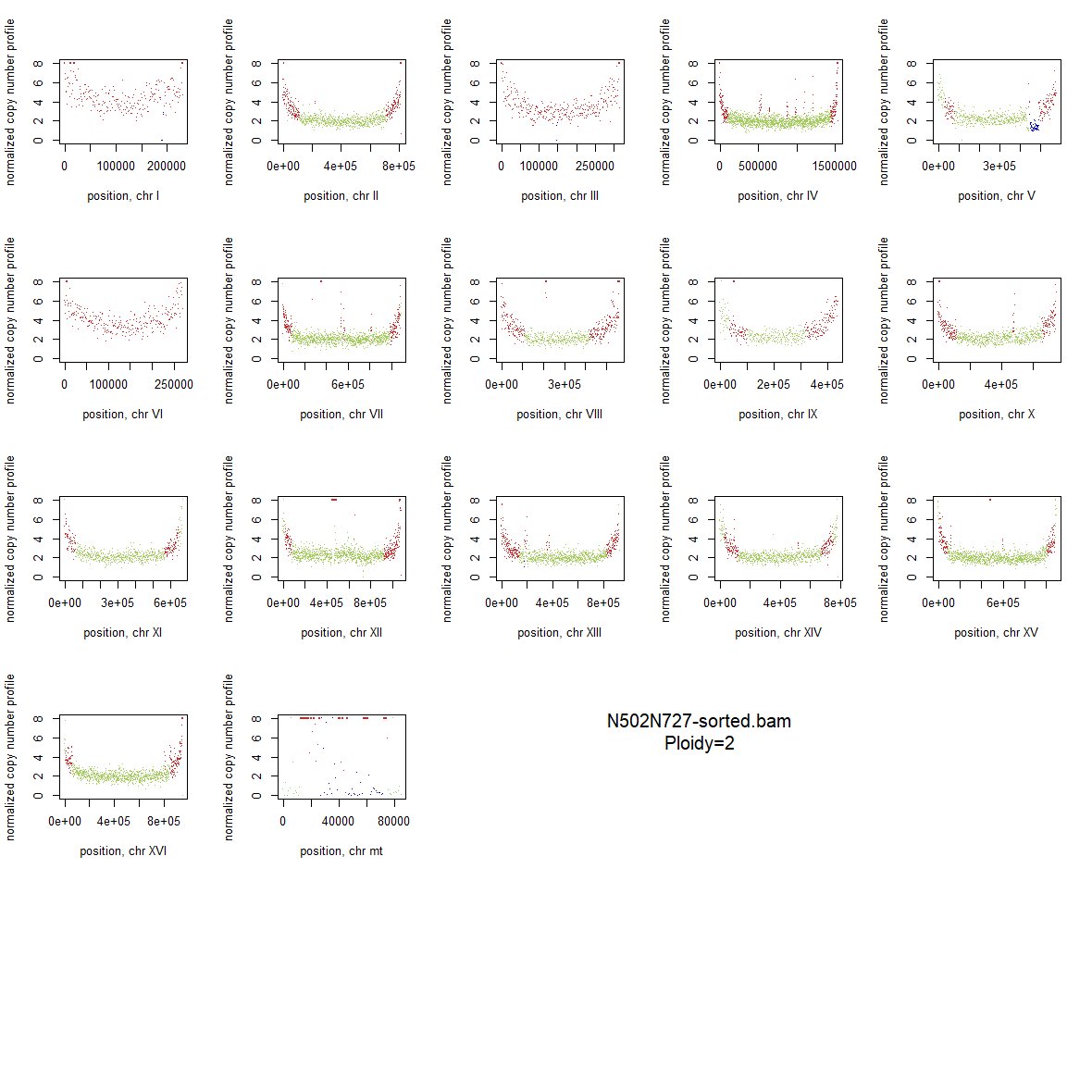

Supplement: Figure 2—source data 2. [file elife-79346-fig2-data2.zip › Figure2-source data 1/pSEC53-SEC53-WT/1x_Wildtype_08.png]

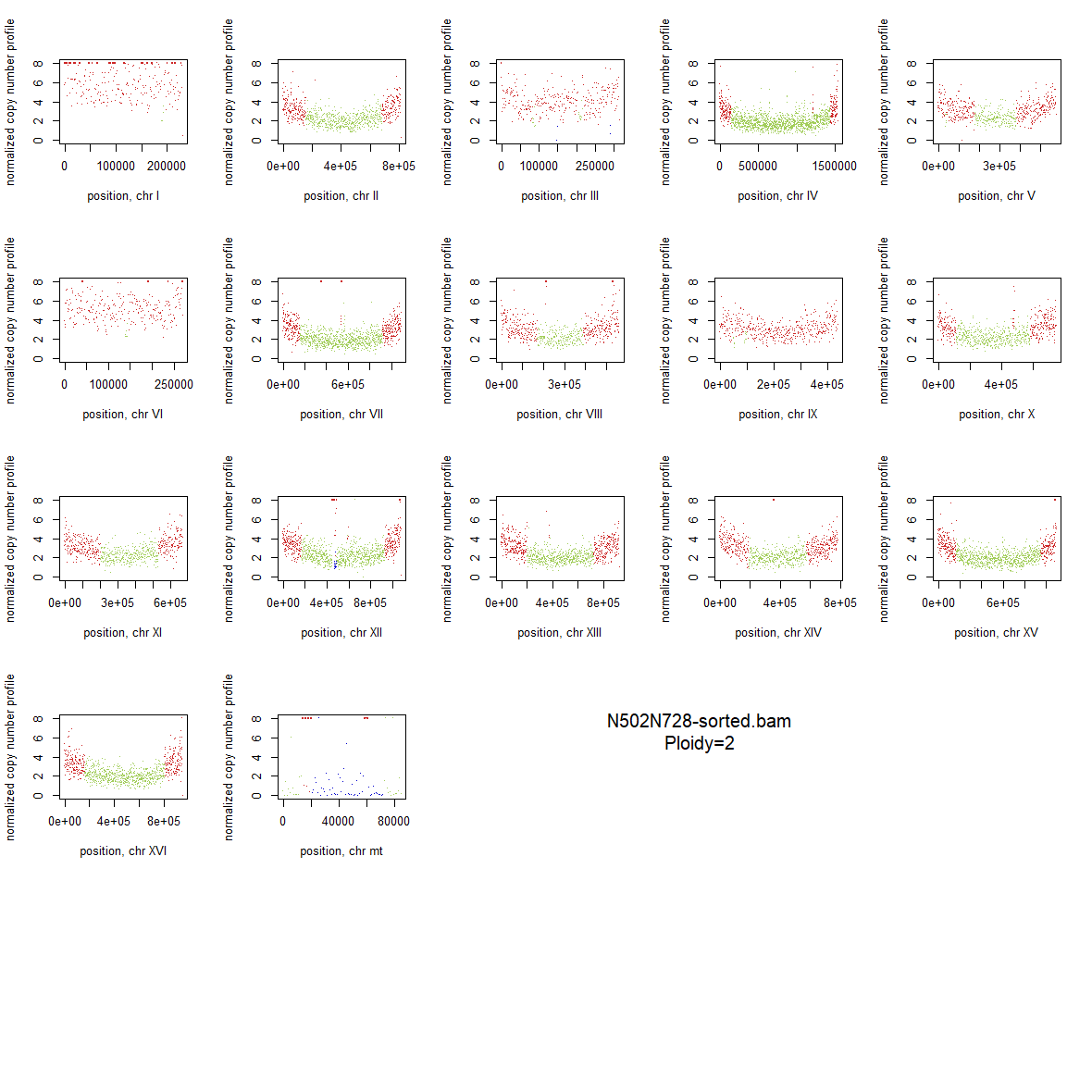

Supplement: Figure 2—source data 2. [file elife-79346-fig2-data2.zip › Figure2-source data 1/pSEC53-SEC53-WT/1x_Wildtype_09.png]

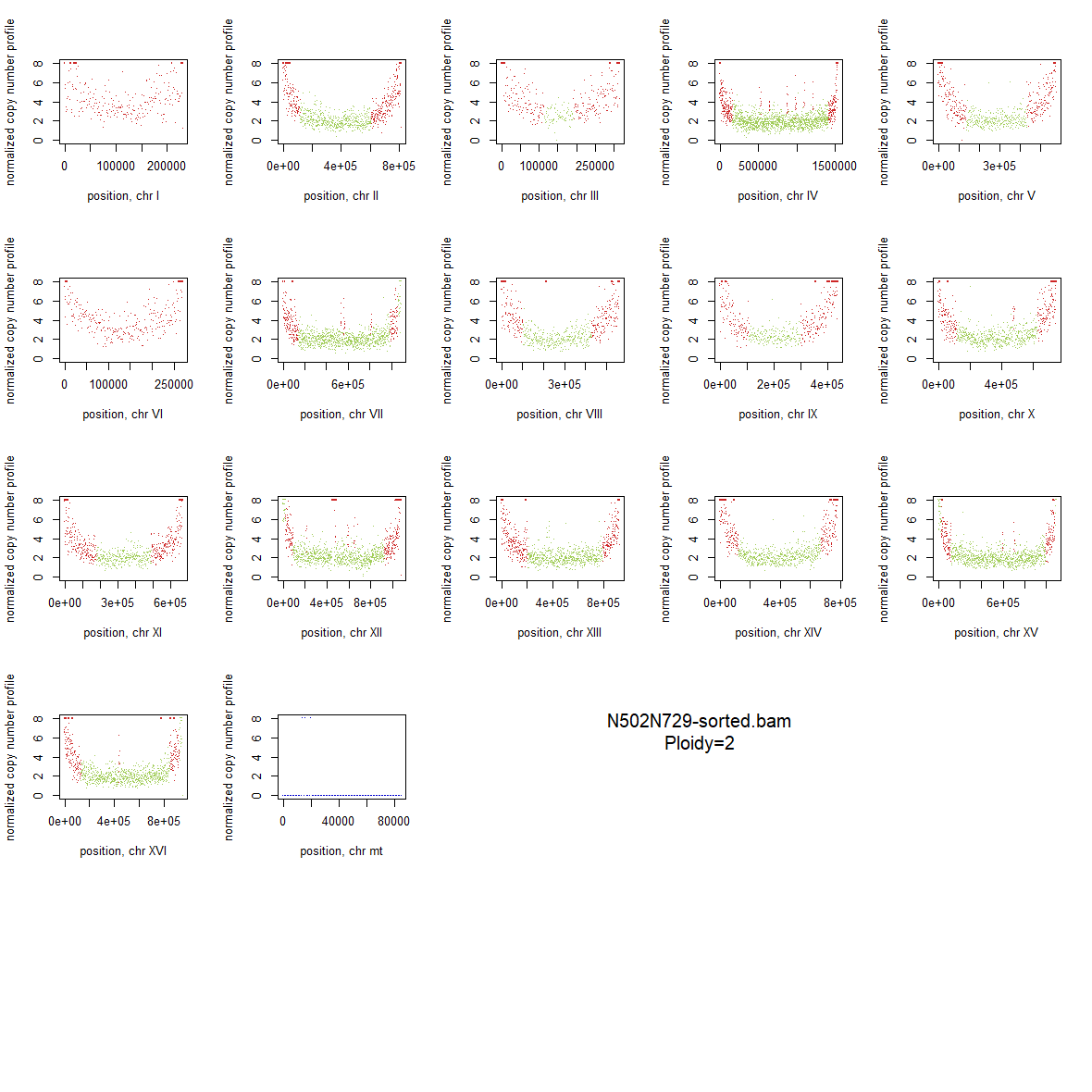

Supplement: Figure 2—source data 2. [file elife-79346-fig2-data2.zip › Figure2-source data 1/pSEC53-SEC53-WT/1x_Wildtype_10.png]

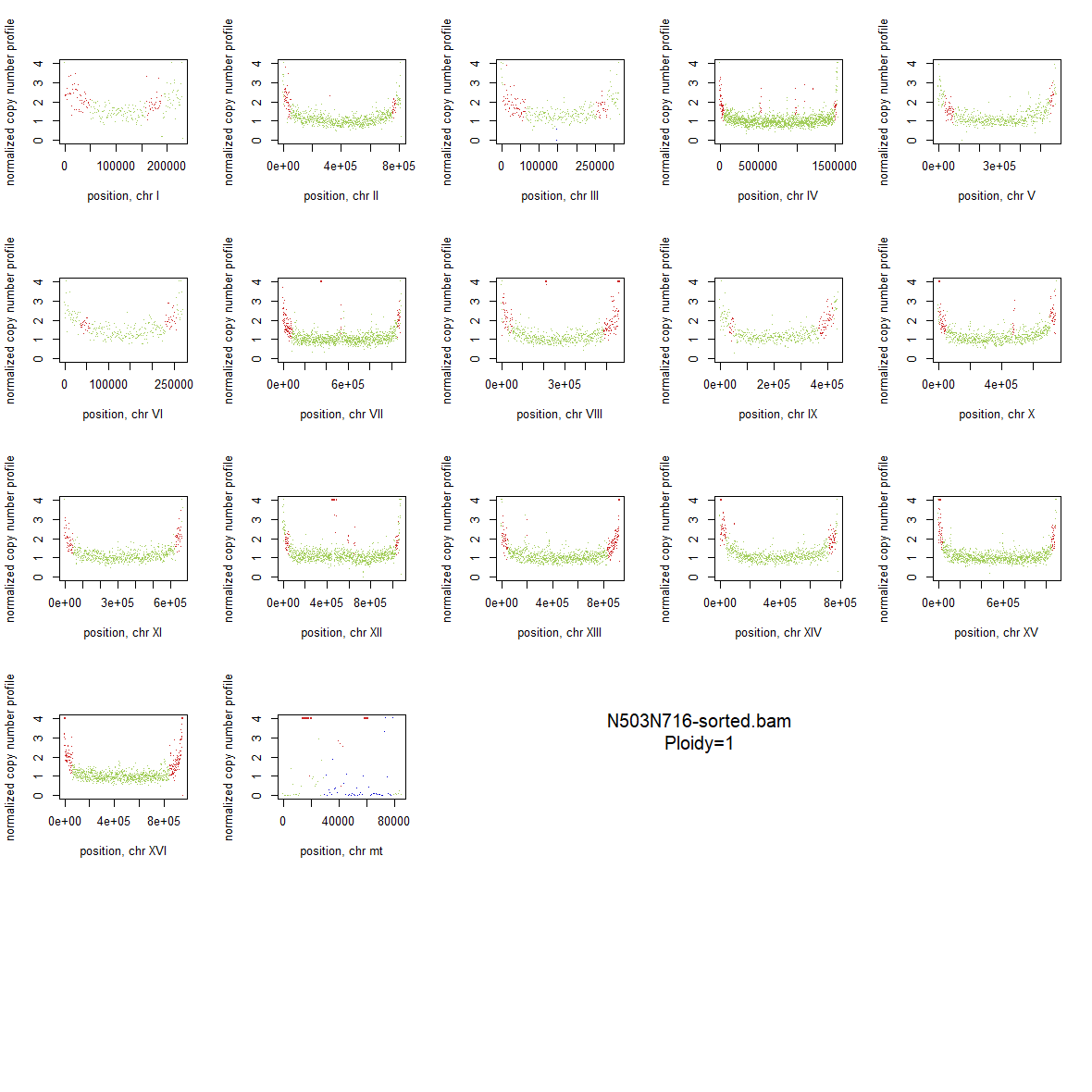

Supplement: Figure 2—source data 2. [file elife-79346-fig2-data2.zip › Figure2-source data 1/pSEC53-SEC53-WT/1x_Wildtype_11.png]

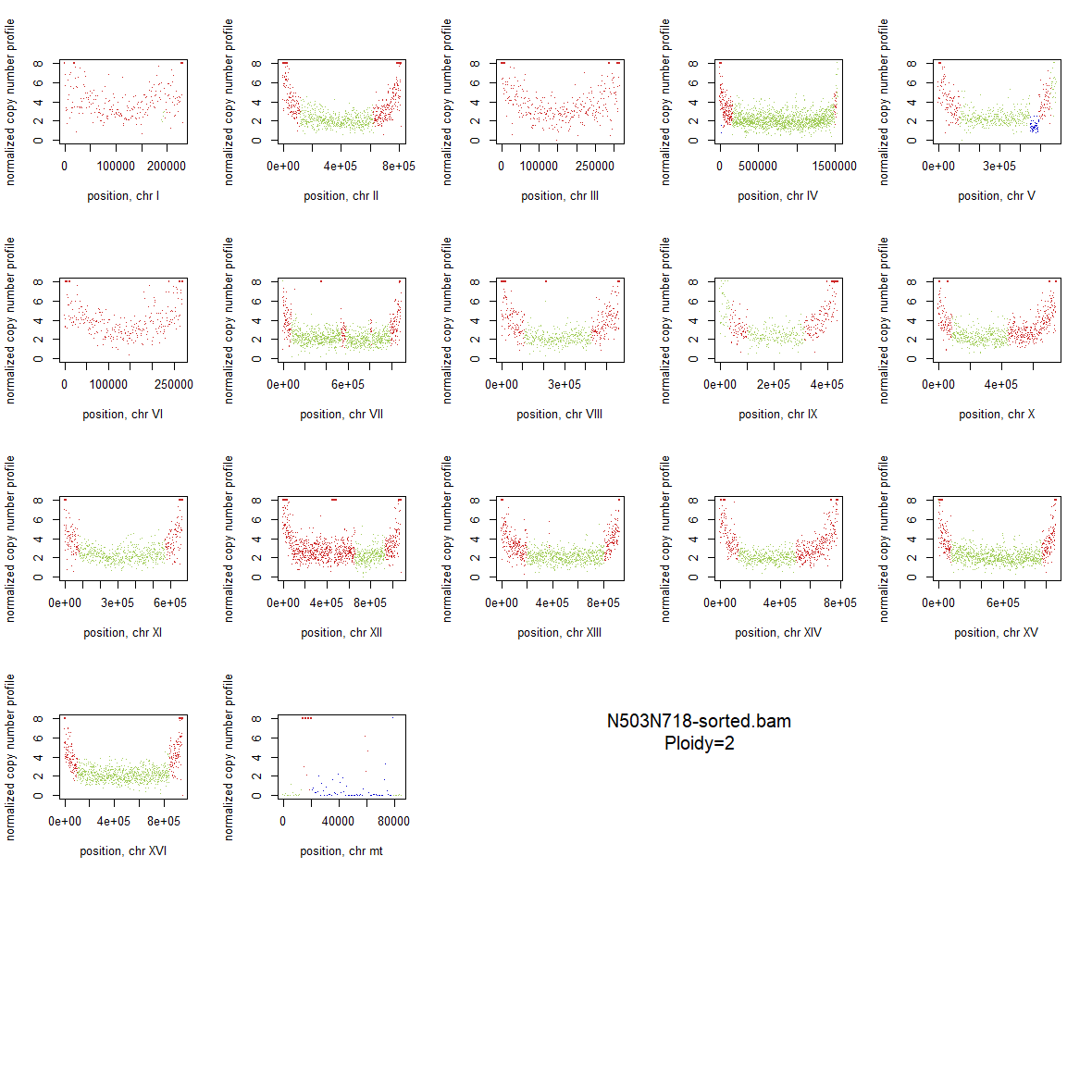

Supplement: Figure 2—source data 2. [file elife-79346-fig2-data2.zip › Figure2-source data 1/pSEC53-SEC53-WT/1x_Wildtype_12.png]

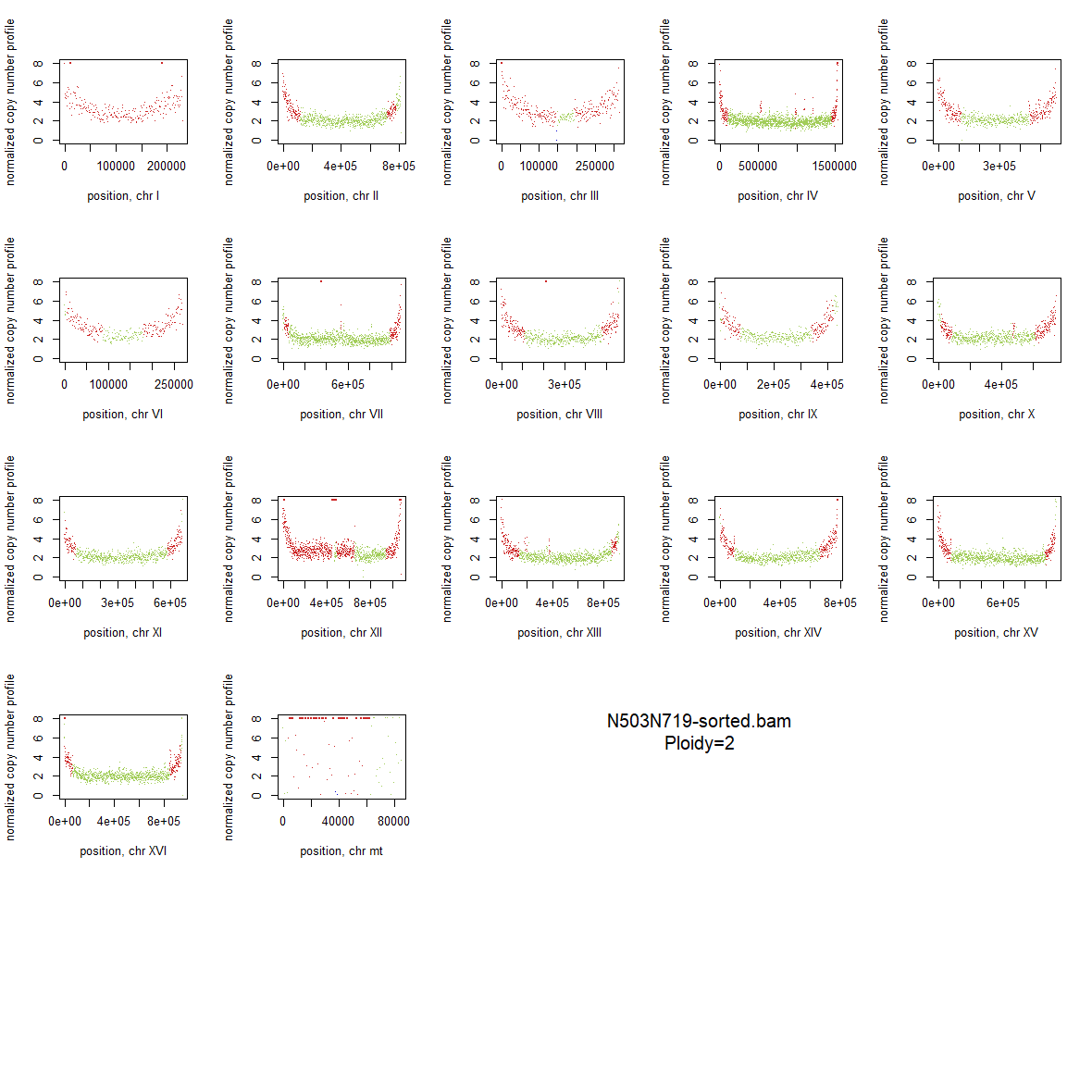

Supplement: Figure 2—source data 2. [file elife-79346-fig2-data2.zip › Figure2-source data 1/pSEC53-SEC53-WT/1x_Wildtype_13.png]

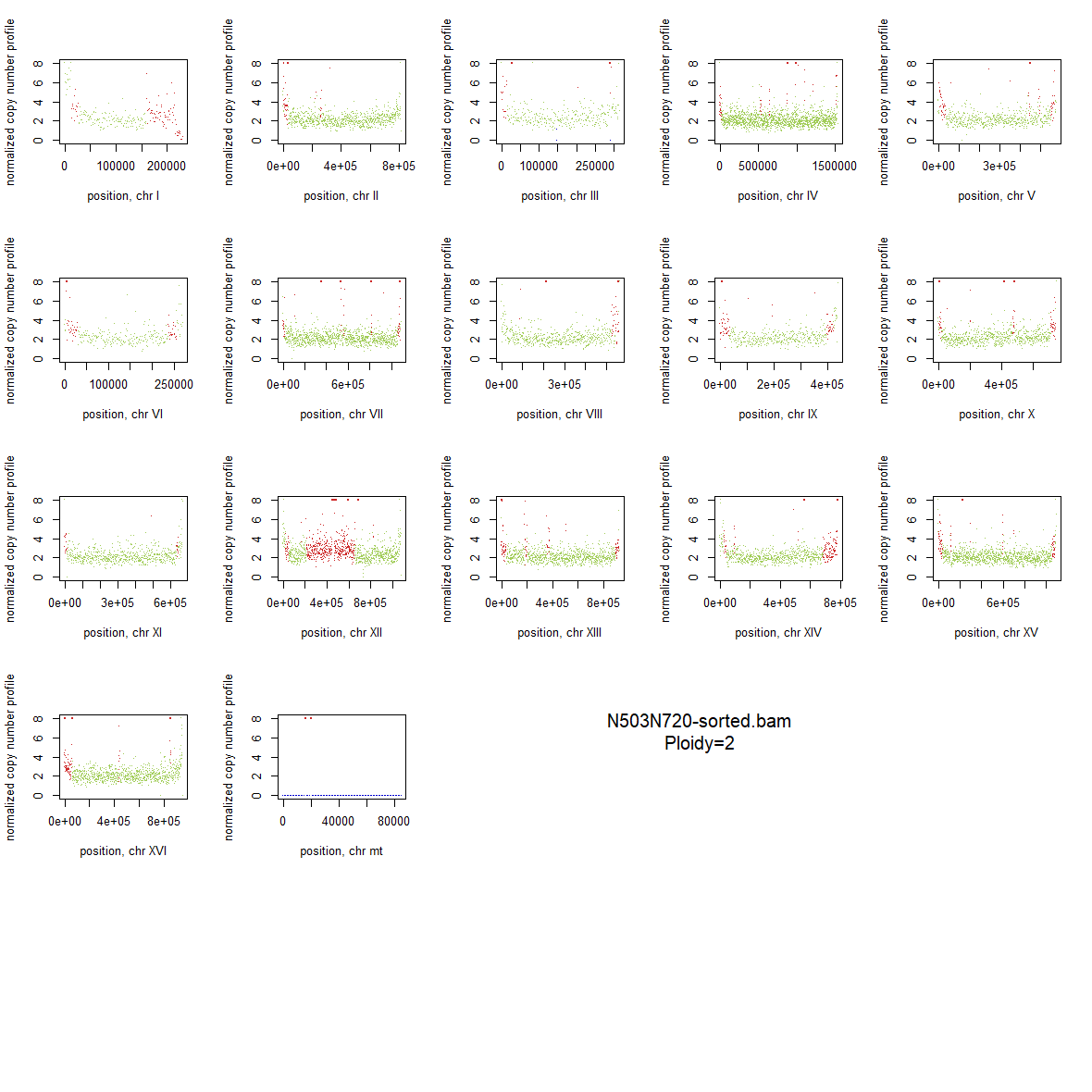

Supplement: Figure 2—source data 2. [file elife-79346-fig2-data2.zip › Figure2-source data 1/pSEC53-SEC53-WT/1x_Wildtype_14.png]

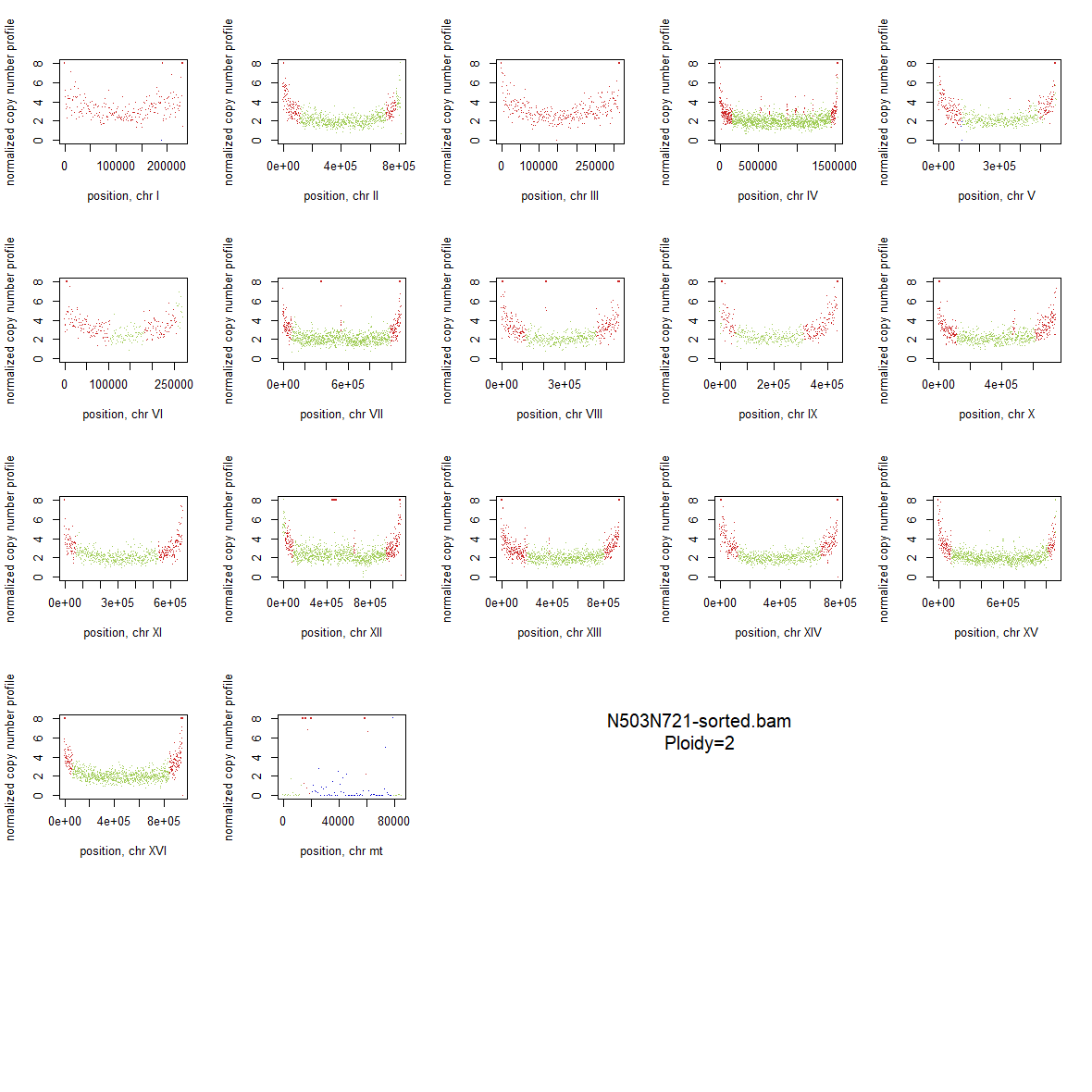

Supplement: Figure 2—source data 2. [file elife-79346-fig2-data2.zip › Figure2-source data 1/pSEC53-SEC53-WT/1x_Wildtype_15.png]

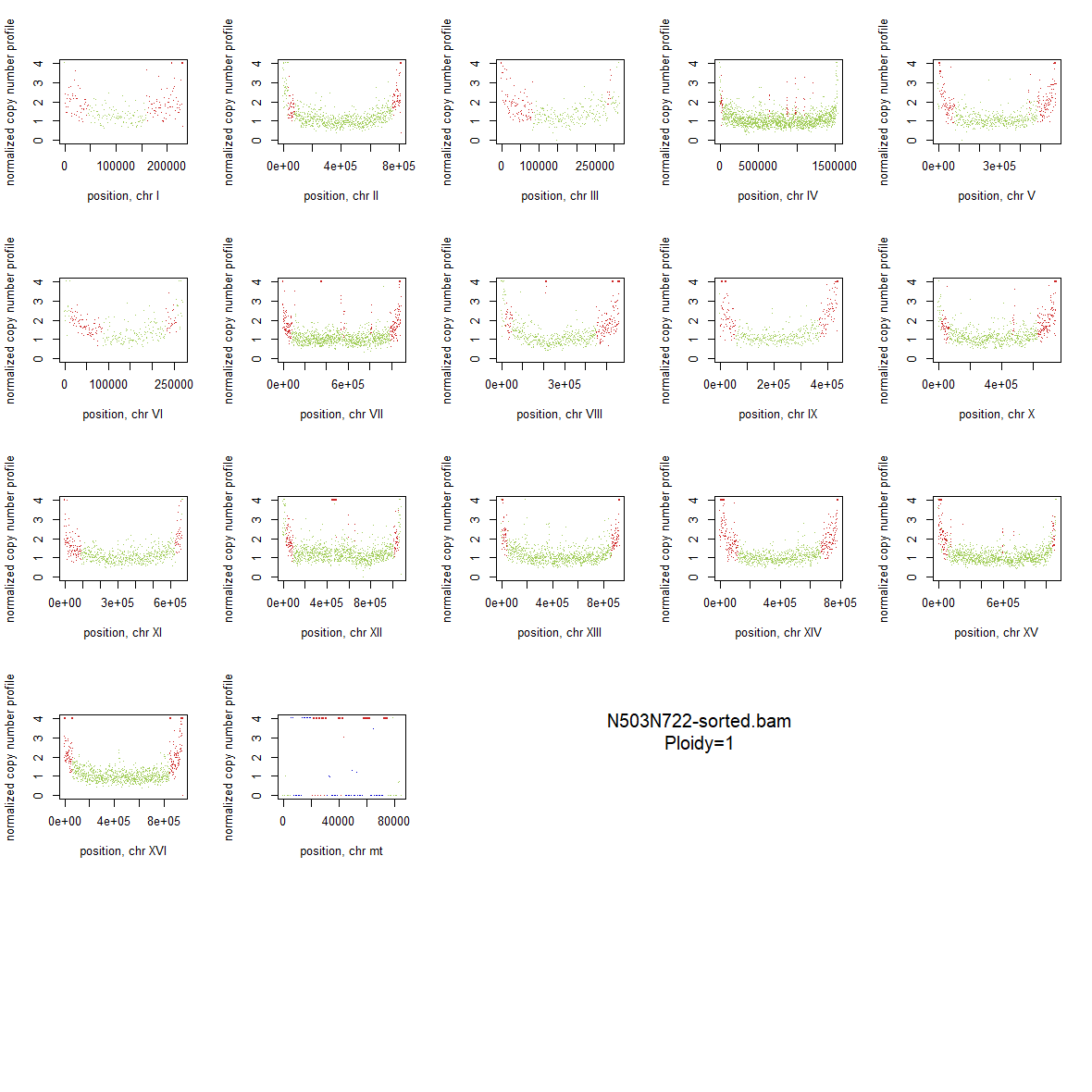

Supplement: Figure 2—source data 2. [file elife-79346-fig2-data2.zip › Figure2-source data 1/pSEC53-SEC53-WT/1x_Wildtype_16.png]

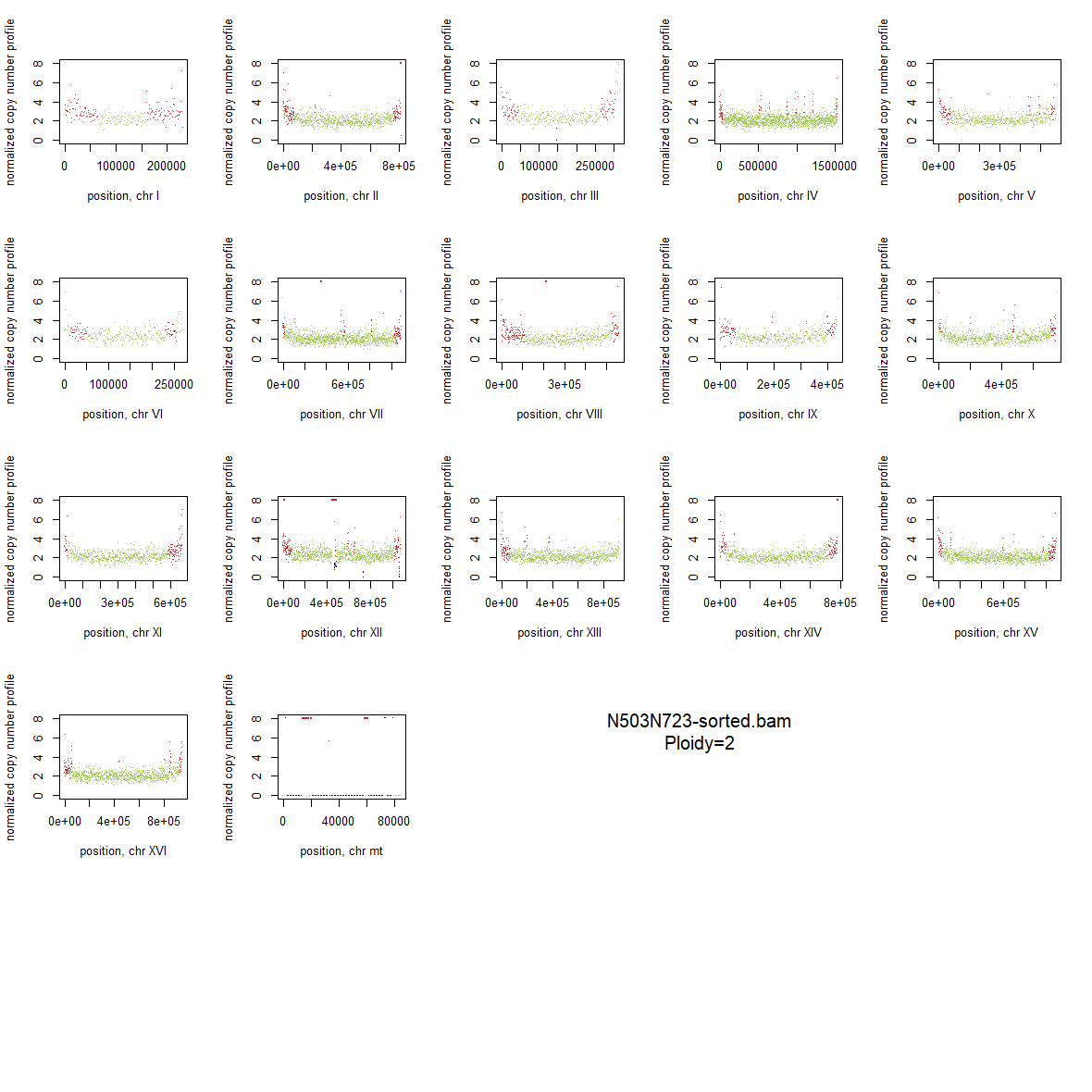

Supplement: Figure 2—source data 2. [file elife-79346-fig2-data2.zip › Figure2-source data 1/pSEC53-SEC53-WT/1x_Wildtype_17.png]

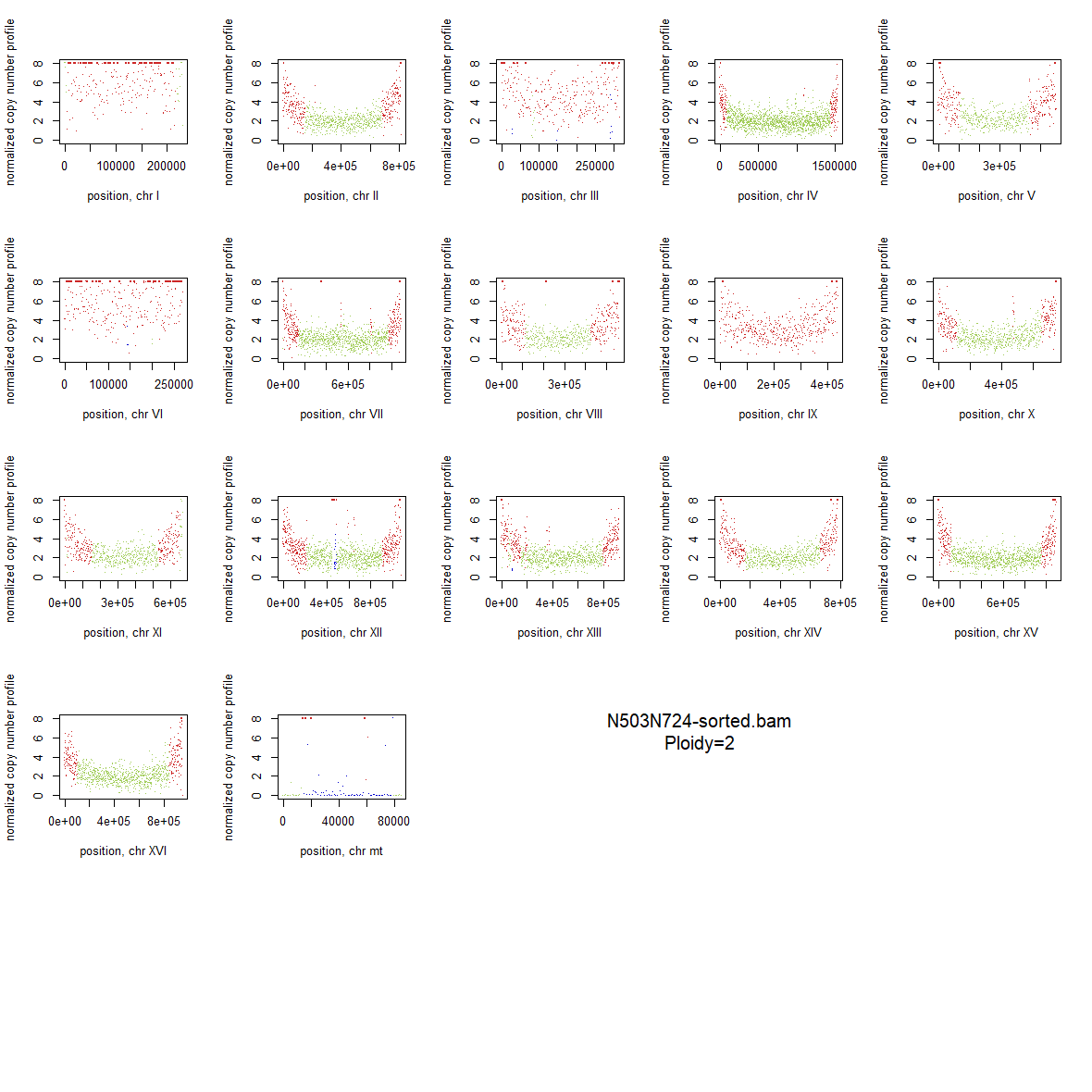

Supplement: Figure 2—source data 2. [file elife-79346-fig2-data2.zip › Figure2-source data 1/pSEC53-SEC53-WT/1x_Wildtype_18.png]

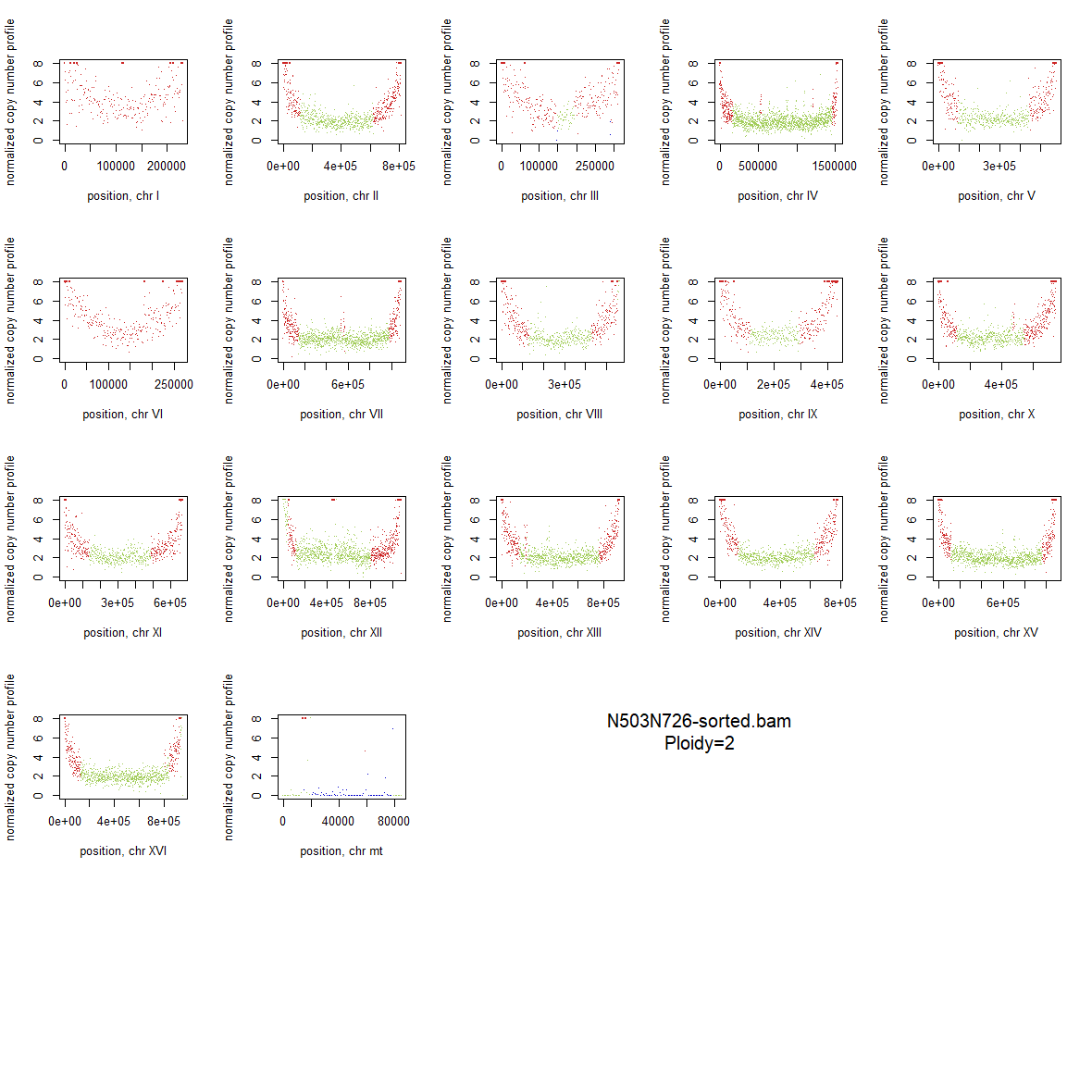

Supplement: Figure 2—source data 2. [file elife-79346-fig2-data2.zip › Figure2-source data 1/pSEC53-SEC53-WT/1x_Wildtype_19.png]

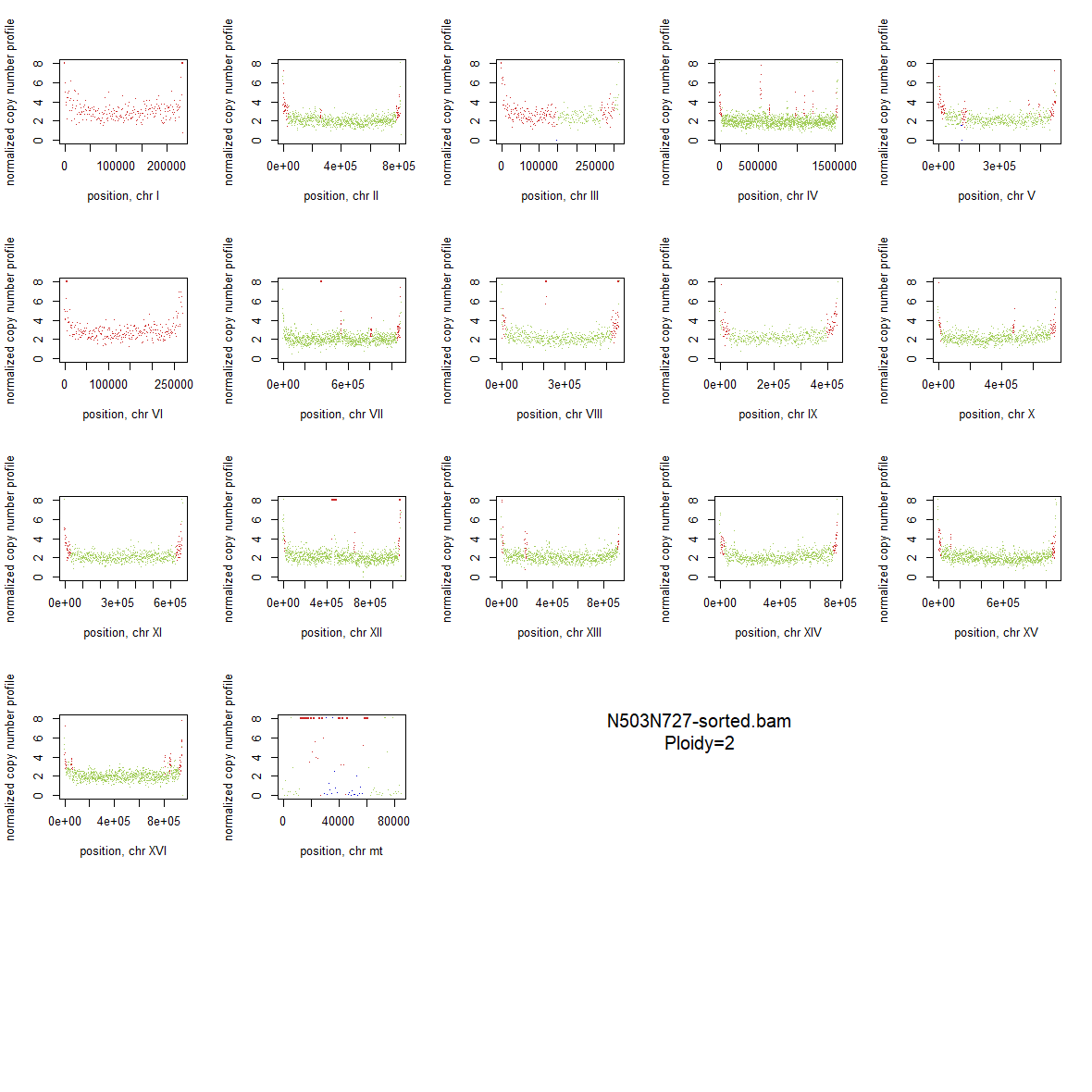

Supplement: Figure 2—source data 2. [file elife-79346-fig2-data2.zip › Figure2-source data 1/pSEC53-SEC53-WT/1x_Wildtype_20.png]

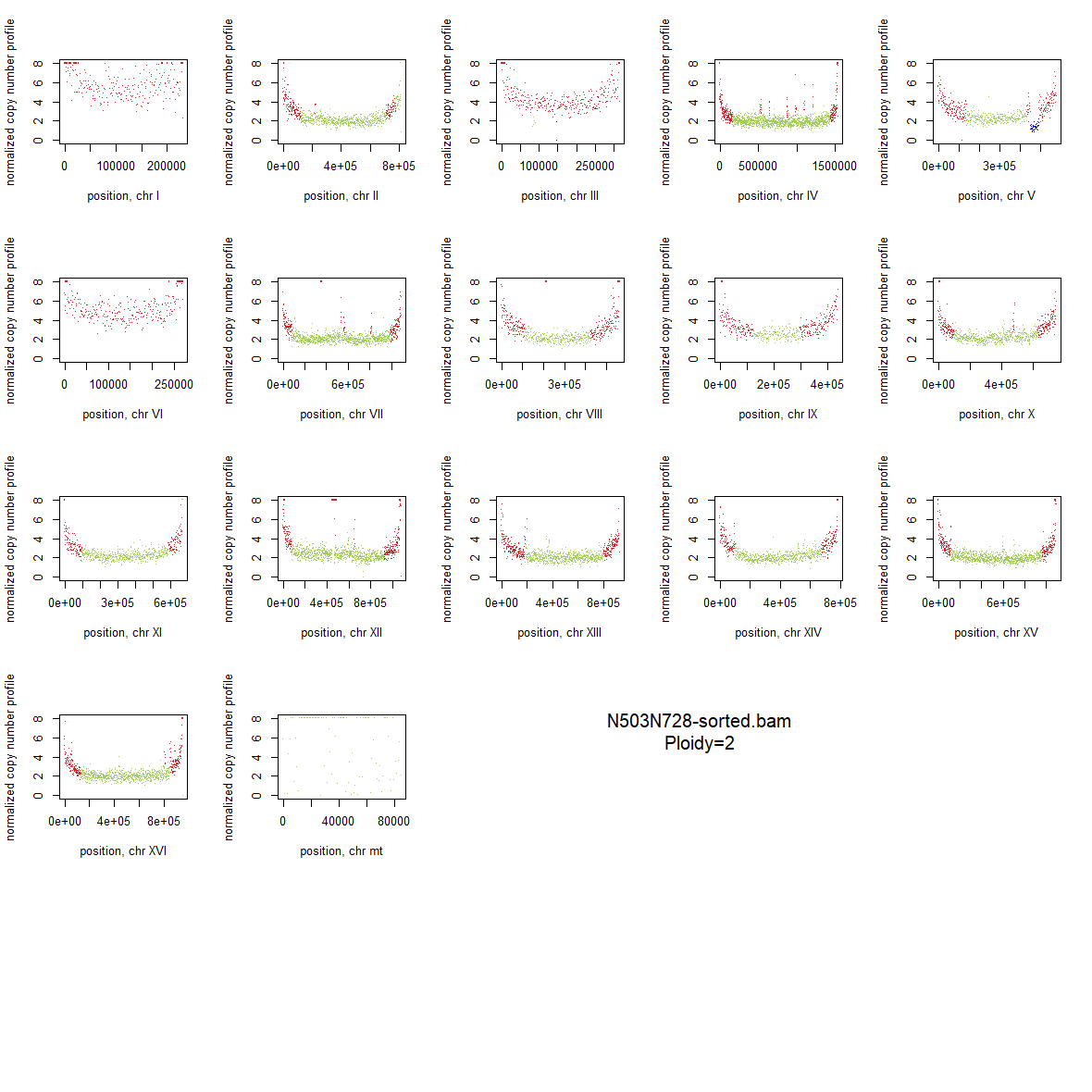

Supplement: Figure 2—source data 2. [file elife-79346-fig2-data2.zip › Figure2-source data 1/pSEC53-SEC53-WT/1x_Wildtype_21.png]

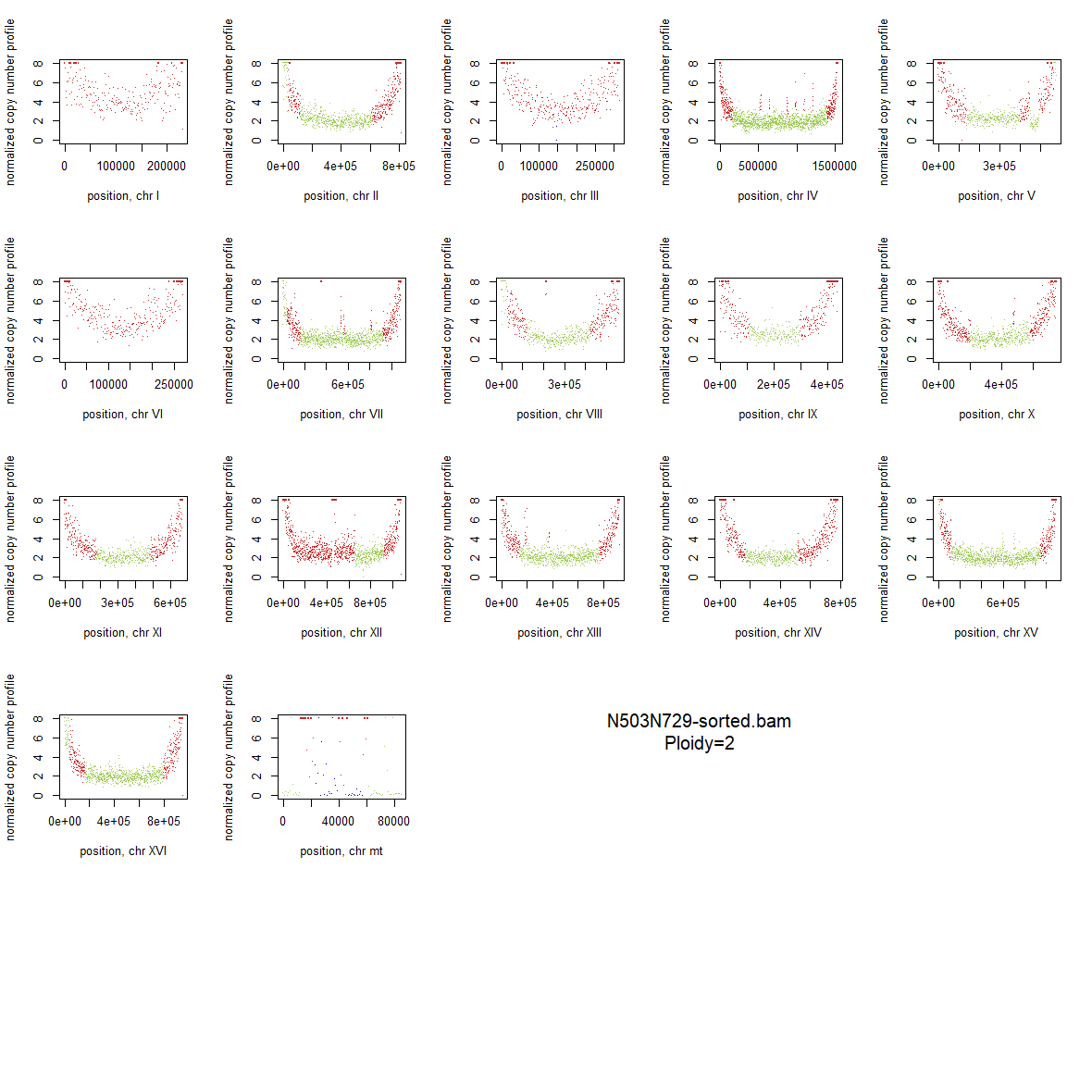

Supplement: Figure 2—source data 2. [file elife-79346-fig2-data2.zip › Figure2-source data 1/pSEC53-SEC53-WT/1x_Wildtype_22.png]

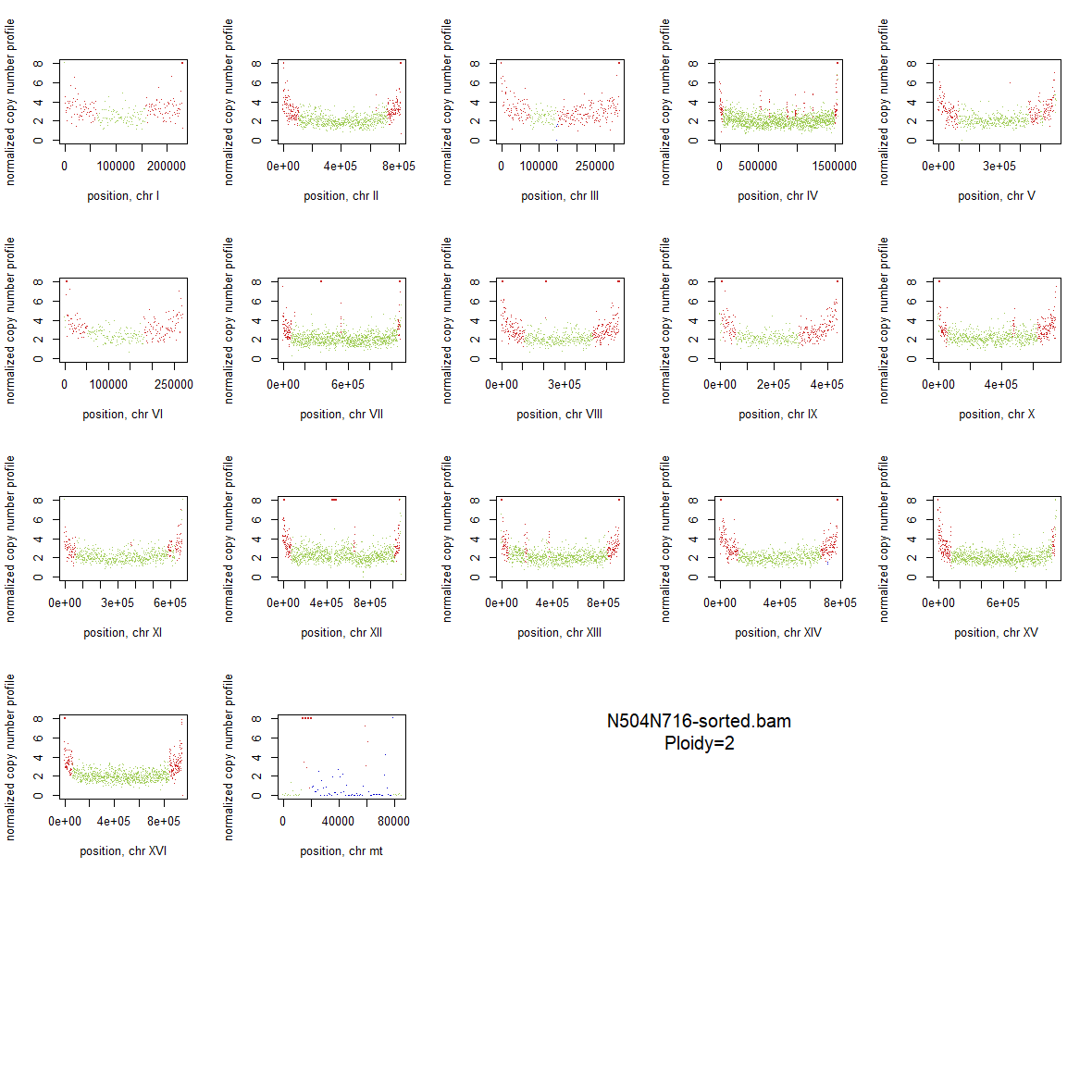

Supplement: Figure 2—source data 2. [file elife-79346-fig2-data2.zip › Figure2-source data 1/pSEC53-SEC53-WT/1x_Wildtype_23.png]

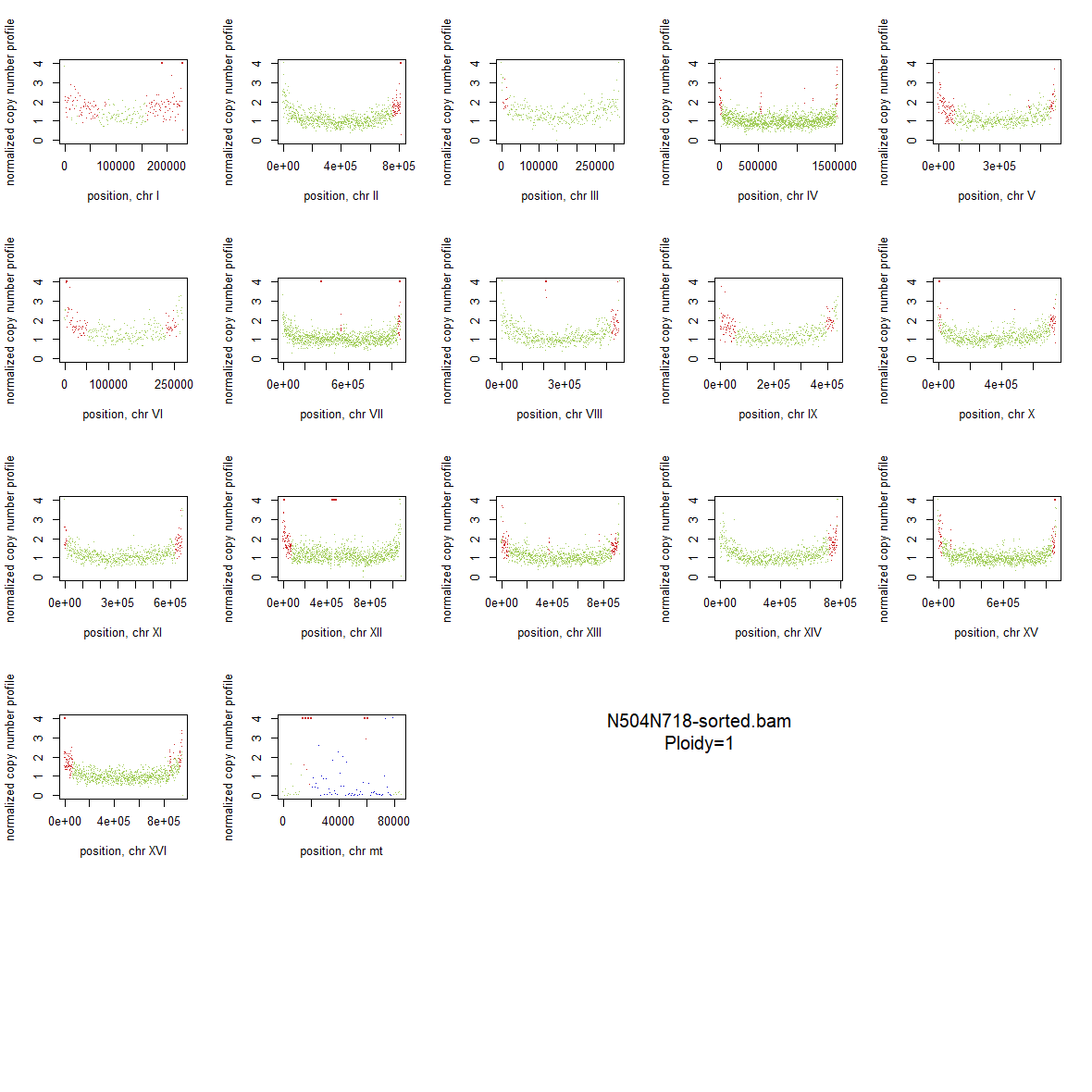

Supplement: Figure 2—source data 2. [file elife-79346-fig2-data2.zip › Figure2-source data 1/pSEC53-SEC53-WT/1x_Wildtype_24.png]

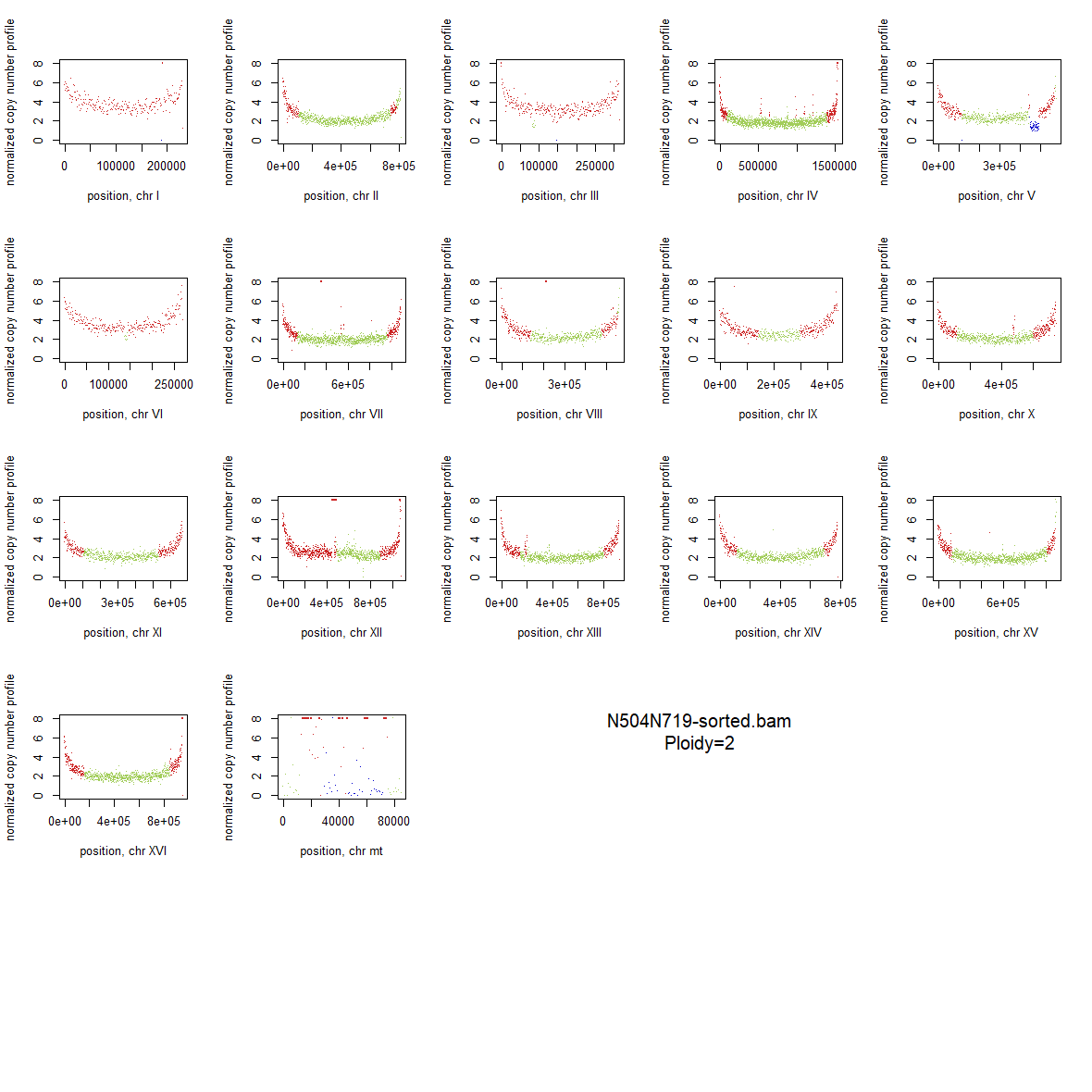

Supplement: Figure 2—source data 2. [file elife-79346-fig2-data2.zip › Figure2-source data 1/pSEC53-SEC53-WT/1x_Wildtype_25.png]

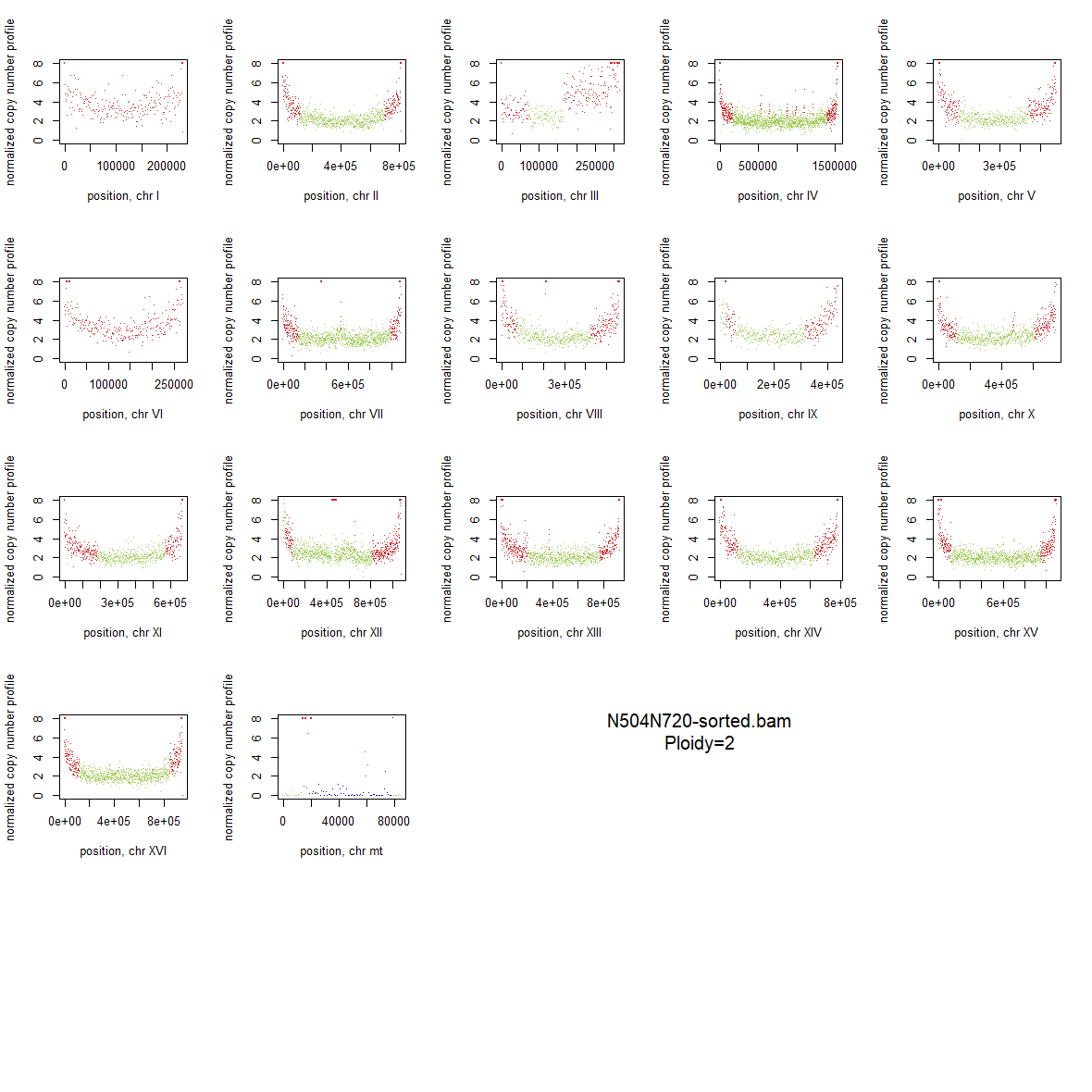

Supplement: Figure 2—source data 2. [file elife-79346-fig2-data2.zip › Figure2-source data 1/pSEC53-SEC53-WT/1x_Wildtype_26.png]

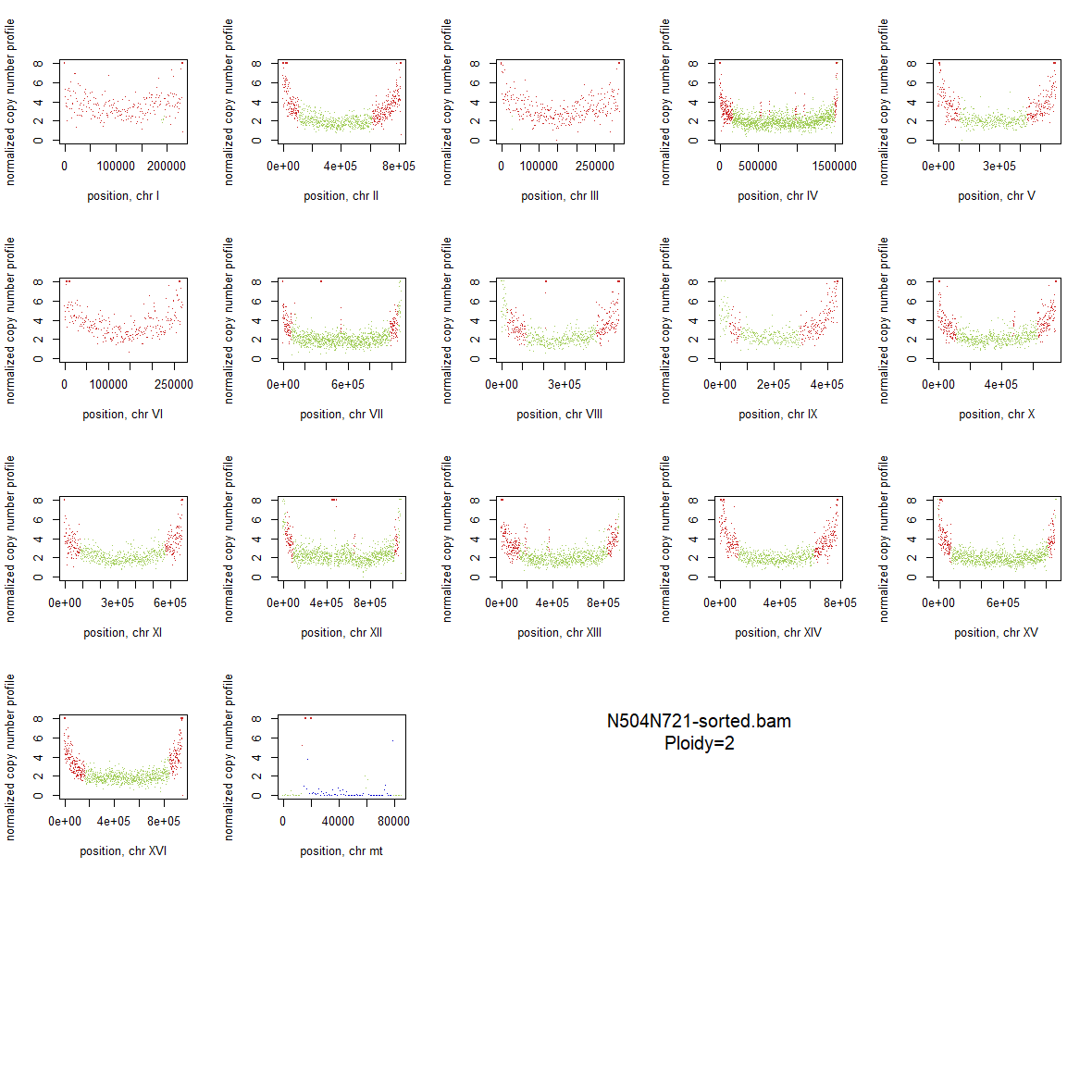

Supplement: Figure 2—source data 2. [file elife-79346-fig2-data2.zip › Figure2-source data 1/pSEC53-SEC53-WT/1x_Wildtype_27.png]

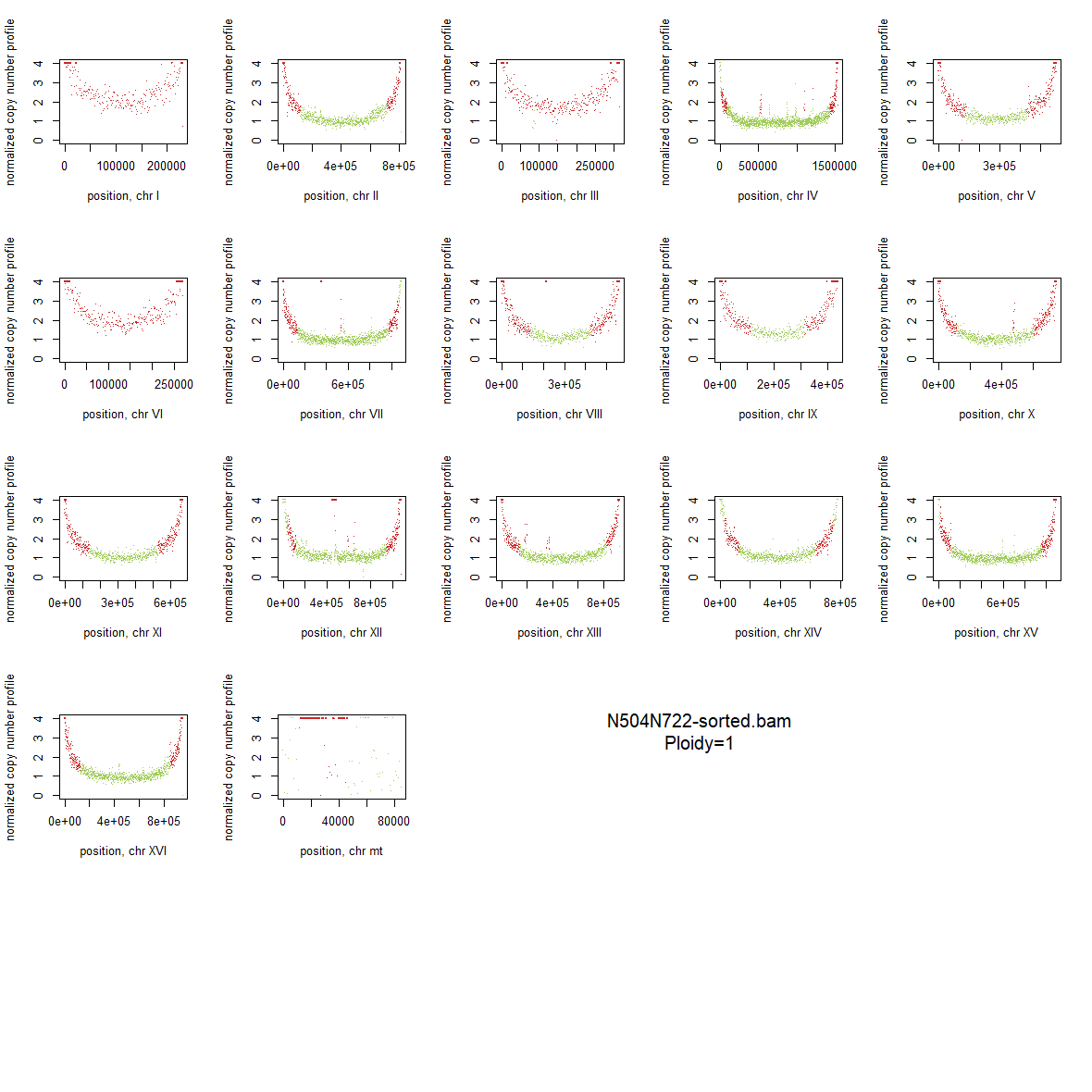

Supplement: Figure 2—source data 2. [file elife-79346-fig2-data2.zip › Figure2-source data 1/pSEC53-SEC53-WT/1x_Wildtype_28.png]

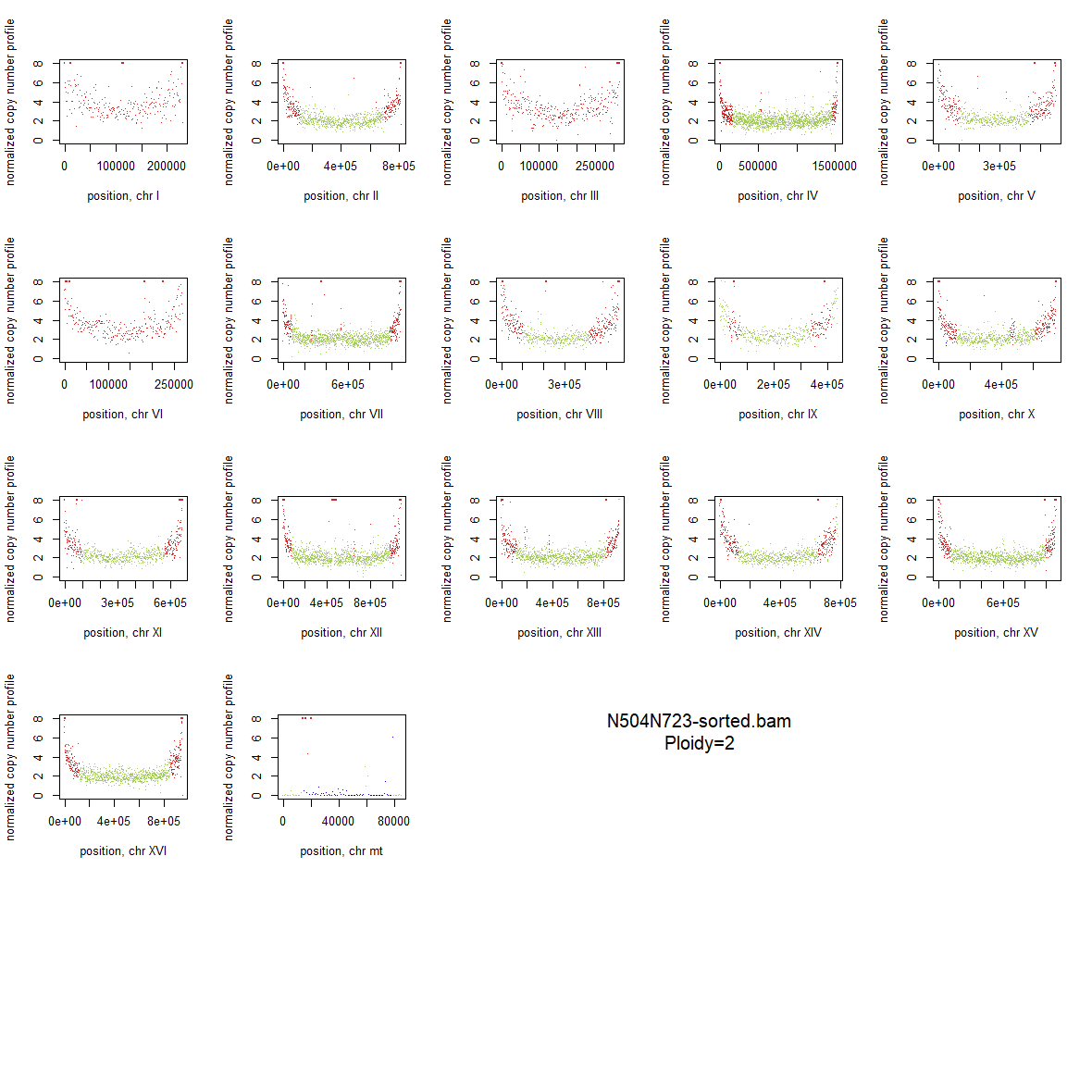

Supplement: Figure 2—source data 2. [file elife-79346-fig2-data2.zip › Figure2-source data 1/pSEC53-SEC53-WT/1x_Wildtype_29.png]

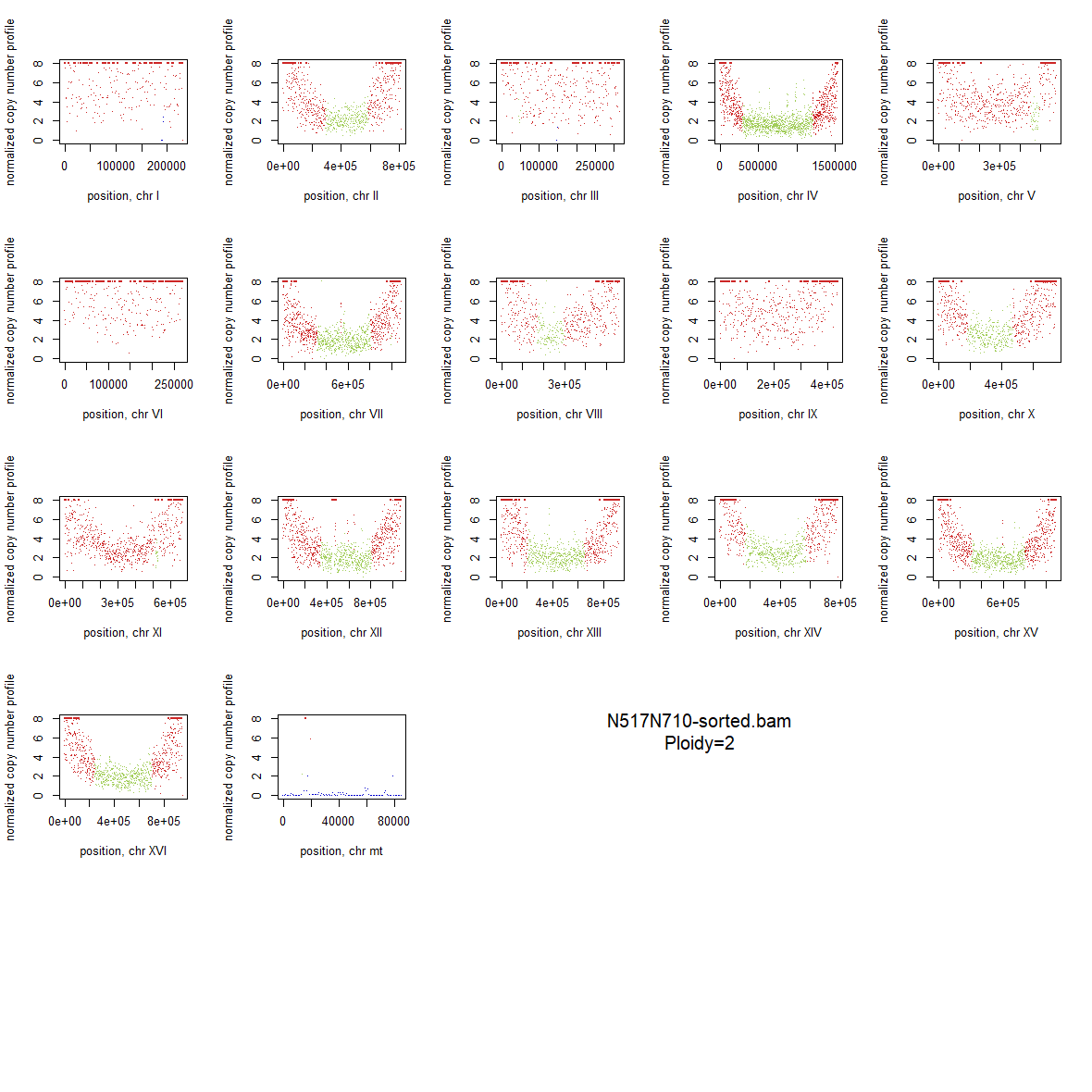

Supplement: Figure 2—source data 2. [file elife-79346-fig2-data2.zip › Figure2-source data 1/pSEC53-SEC53-WT/1x_Wildtype_30.png]

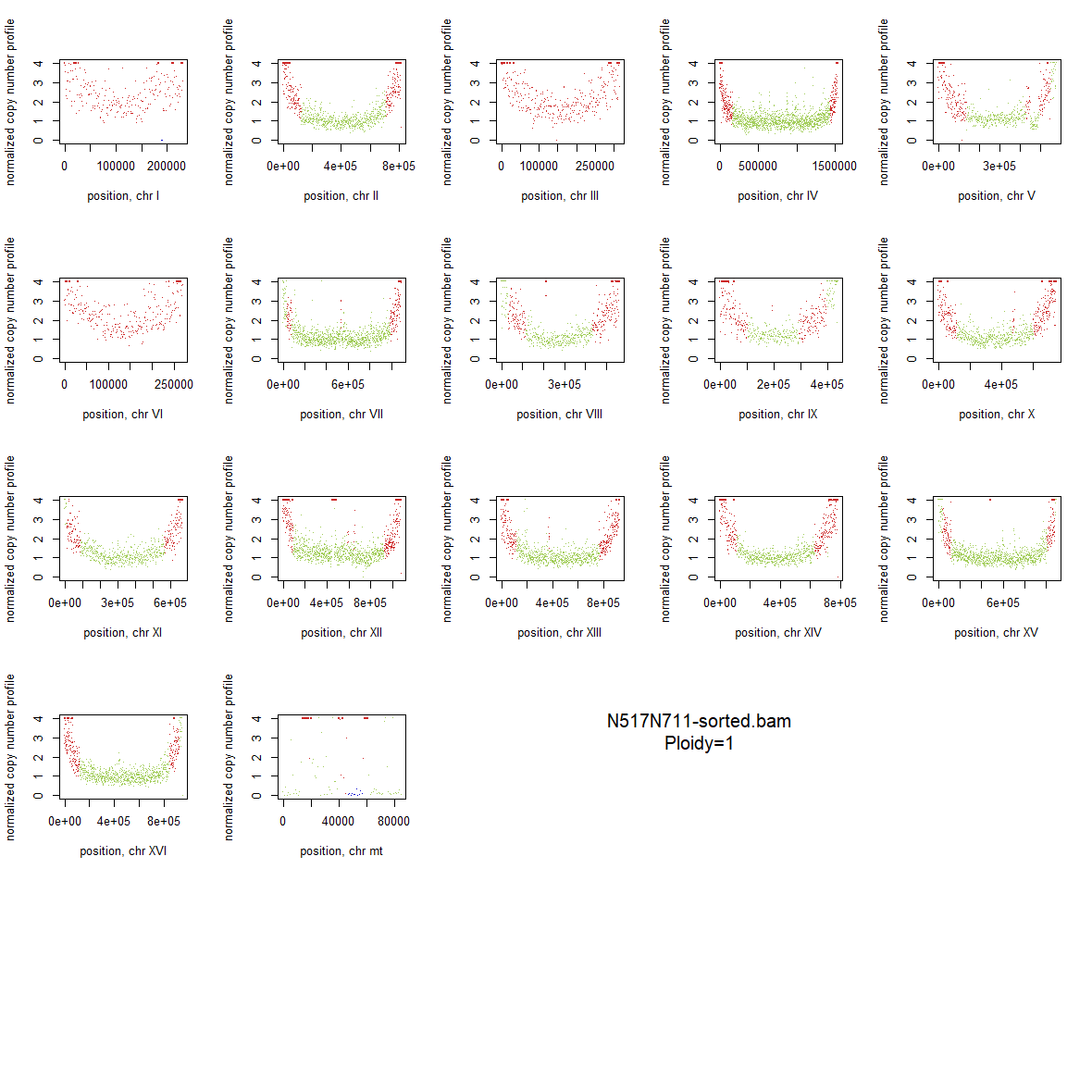

Supplement: Figure 2—source data 2. [file elife-79346-fig2-data2.zip › Figure2-source data 1/pSEC53-SEC53-WT/1x_Wildtype_31.png]

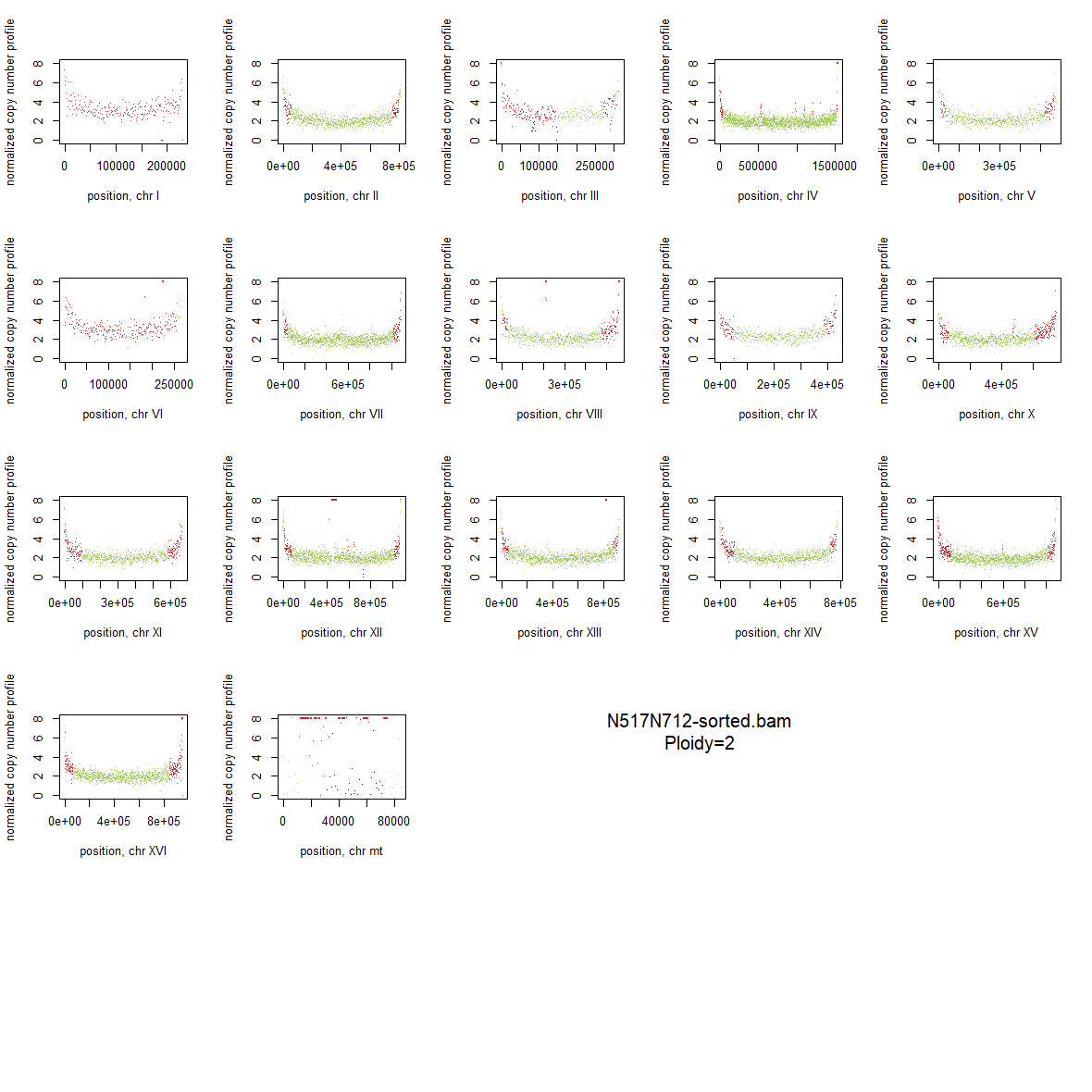

Supplement: Figure 2—source data 2. [file elife-79346-fig2-data2.zip › Figure2-source data 1/pSEC53-SEC53-WT/1x_Wildtype_32.png]

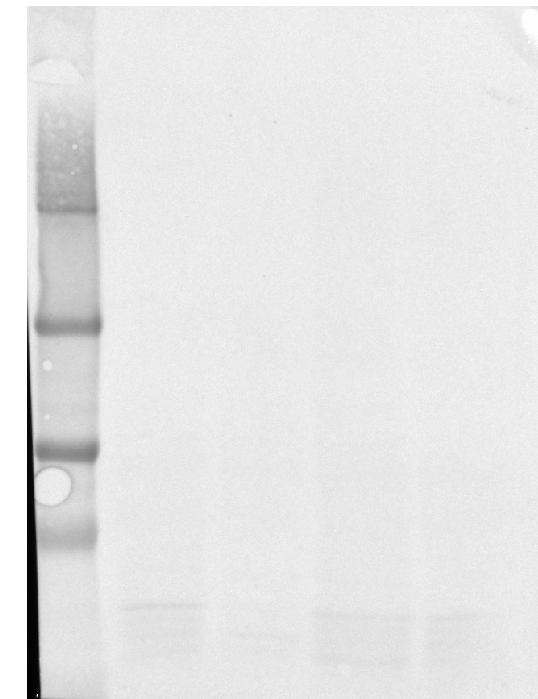

Supplement: Figure 3—source data 1. [file elife-79346-fig3-data1.zip › Figure3-source data 1/ponceau_V238M_invertase.tif]

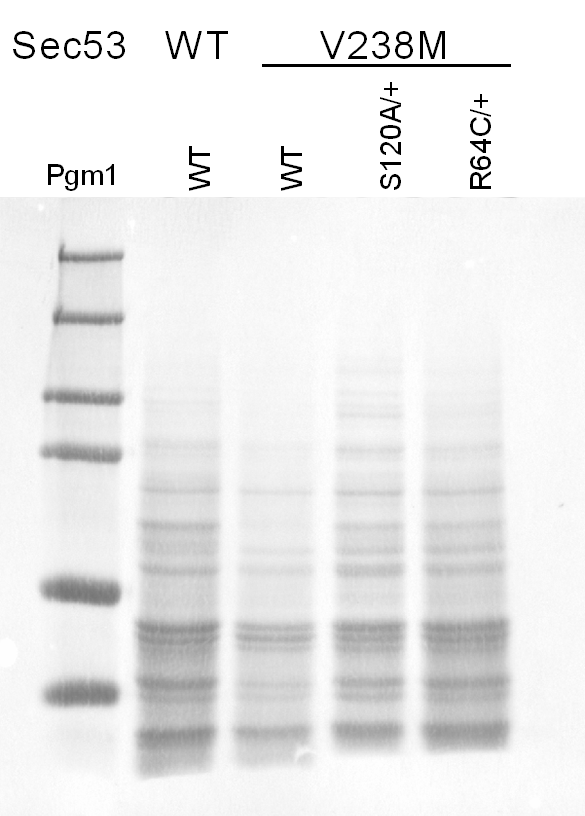

Supplement: Figure 3—source data 1. [file elife-79346-fig3-data1.zip › Figure3-source data 1/ponceau_V238M_CPY_labeled.tif]

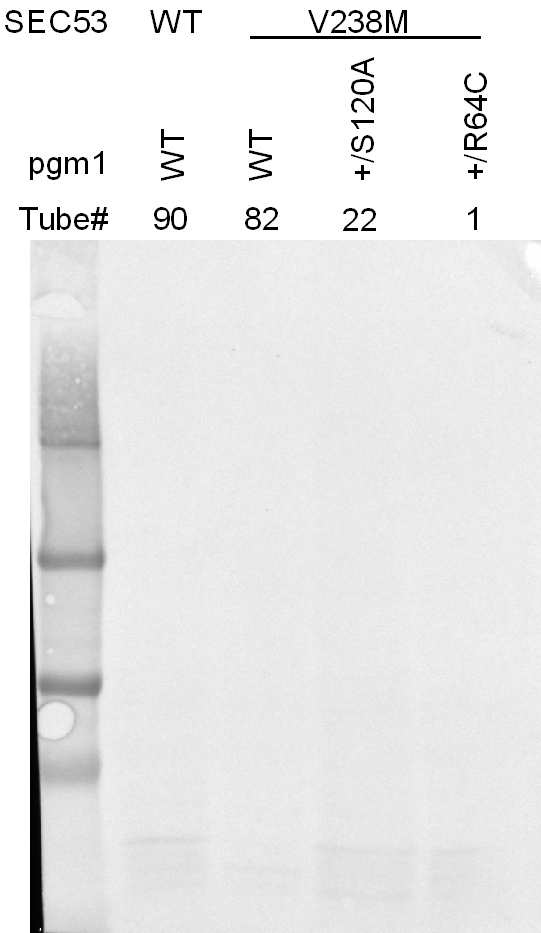

Supplement: Figure 3—source data 1. [file elife-79346-fig3-data1.zip › Figure3-source data 1/ponceau_V238M_invertase_labeled.tif]

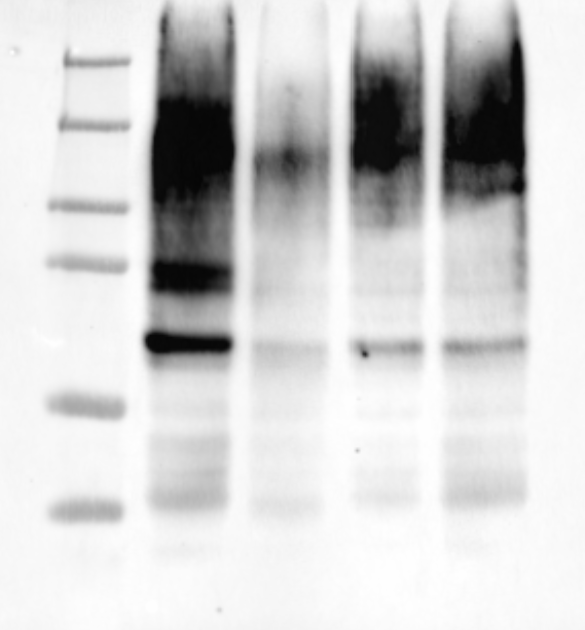

Supplement: Figure 3—source data 1. [file elife-79346-fig3-data1.zip › Figure3-source data 1/uncropped_blot_V238M_CPY.tiff]

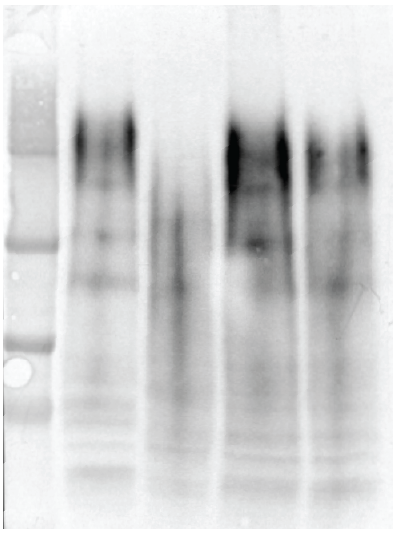

Supplement: Figure 3—source data 1. [file elife-79346-fig3-data1.zip › Figure3-source data 1/uncropped_blot_V238M_invertase.tiff]

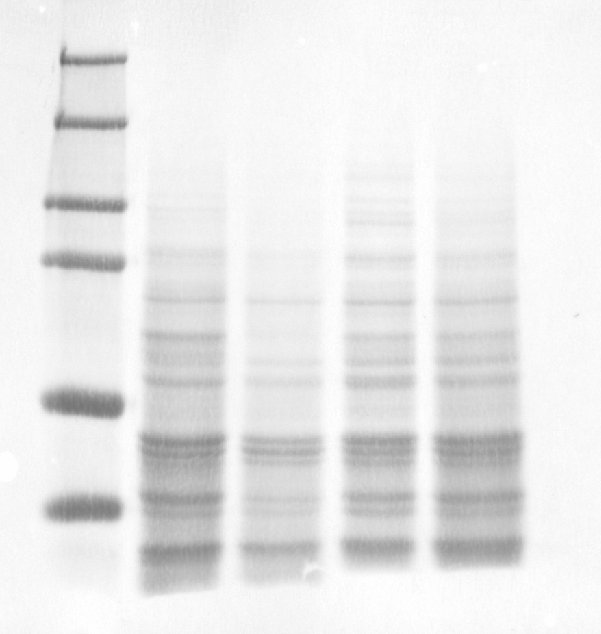

Supplement: Figure 3—source data 1. [file elife-79346-fig3-data1.zip › Figure3-source data 1/ponceau_V238M_CPY.tiff]

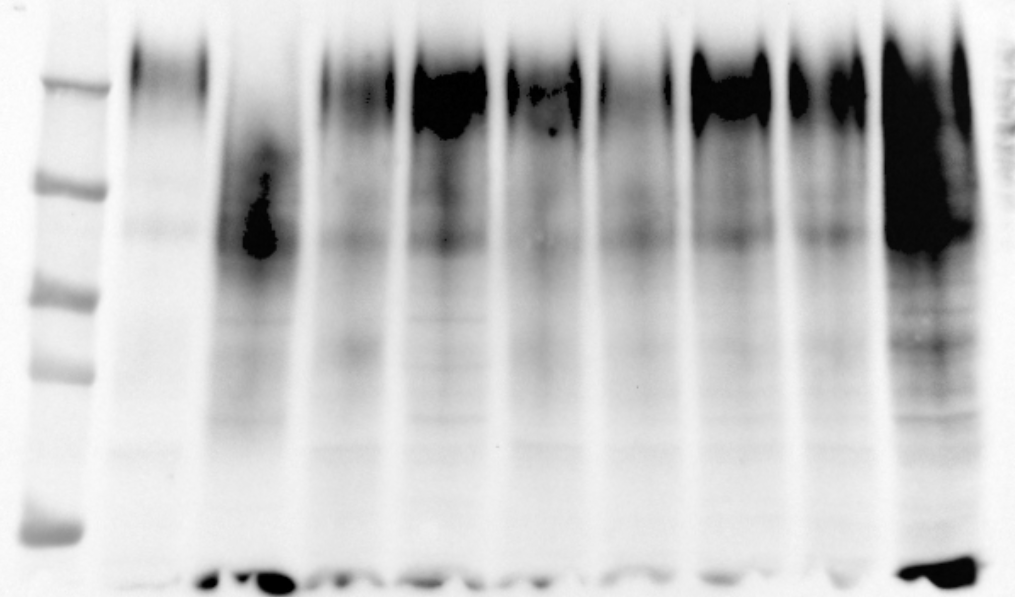

Supplement: Figure 3—figure supplement 4—source data 1. [file elife-79346-fig3-figsupp4-data1.zip › Figure3-figure supplement 4-source data 1/uncropped_blot_V238M_invertase.tif]

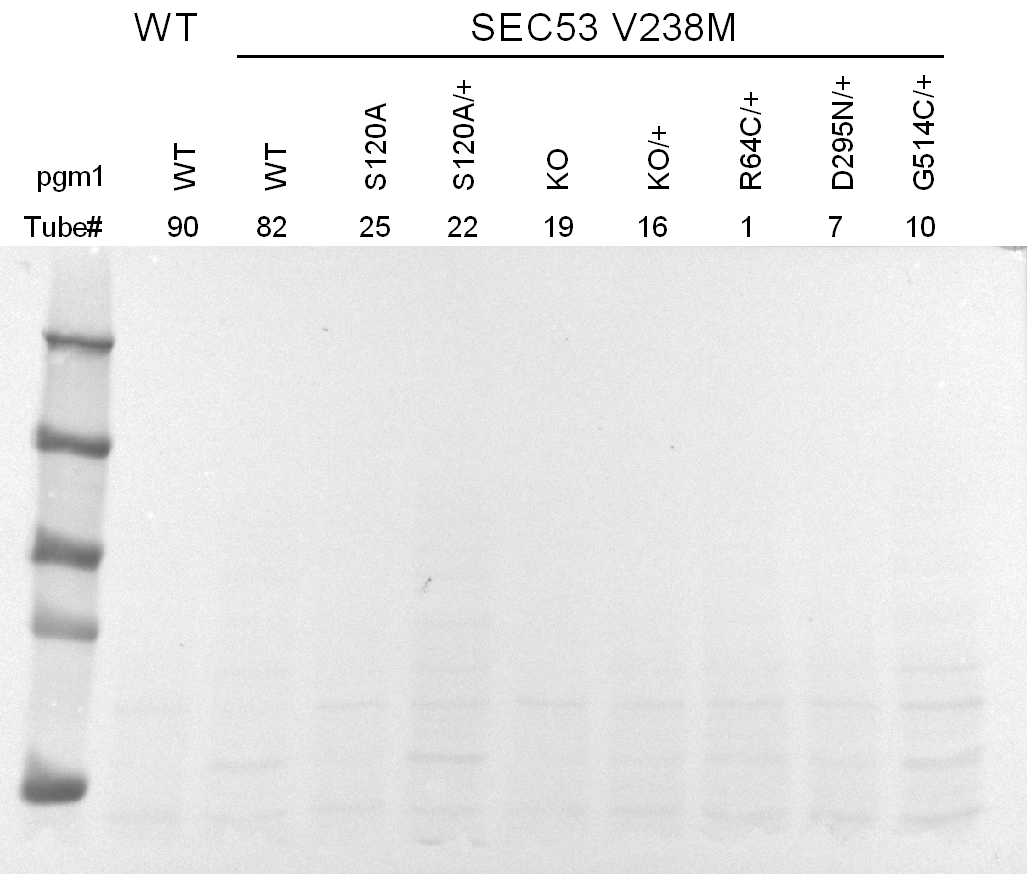

Supplement: Figure 3—figure supplement 4—source data 1. [file elife-79346-fig3-figsupp4-data1.zip › Figure3-figure supplement 4-source data 1/ponceauC_V238M_invertase_labeled.tif]

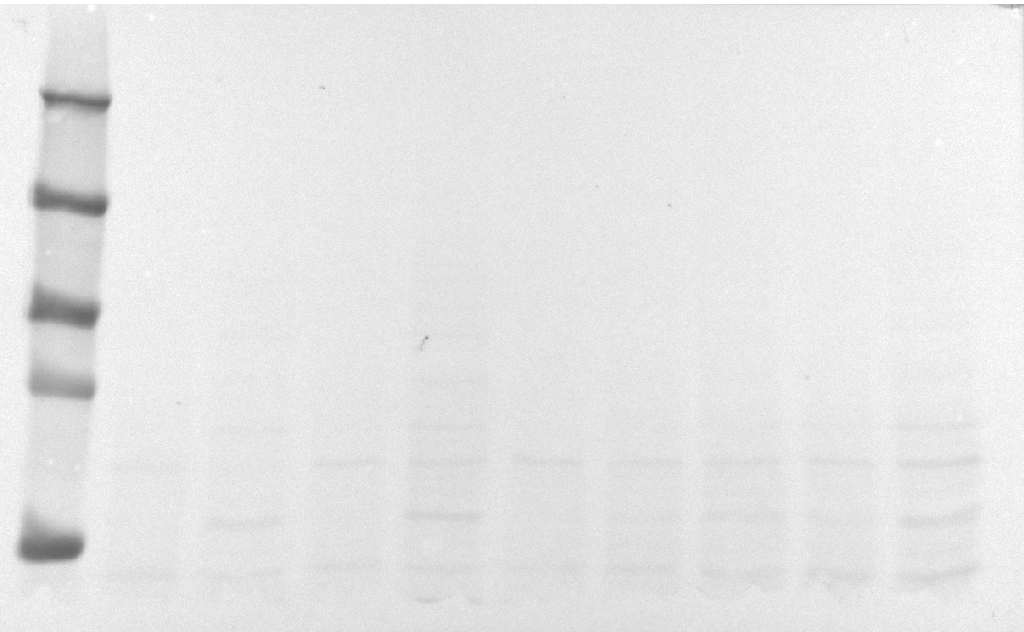

Supplement: Figure 3—figure supplement 4—source data 1. [file elife-79346-fig3-figsupp4-data1.zip › Figure3-figure supplement 4-source data 1/ponceauC_V238M_invertase.tif]

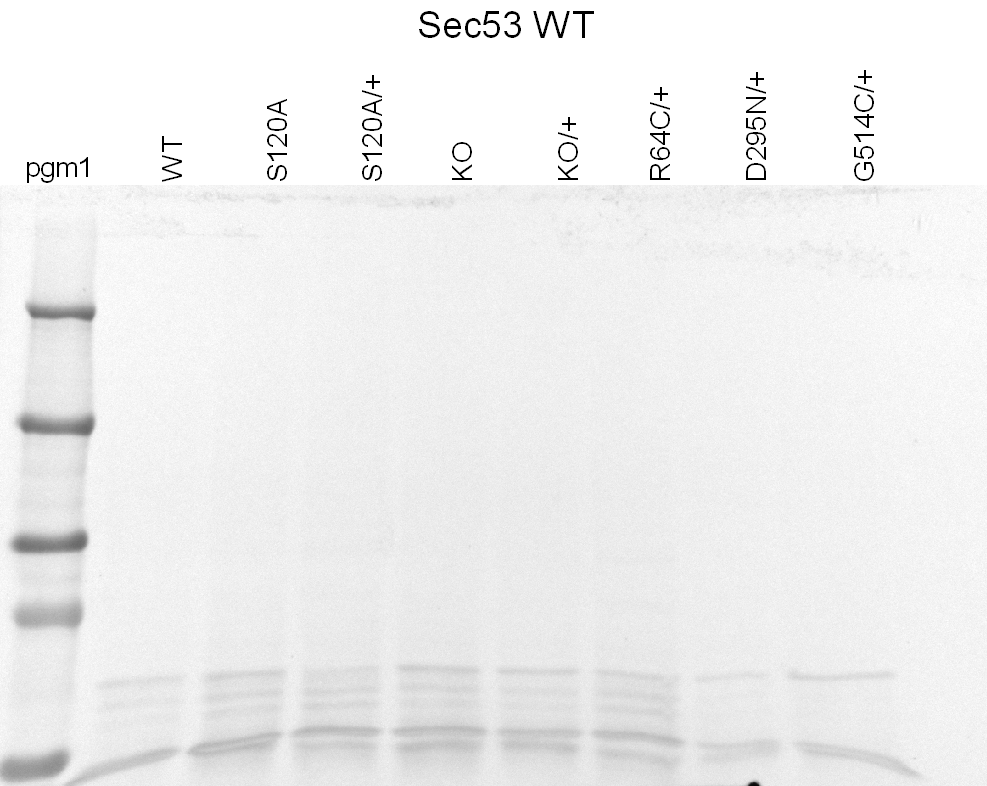

Supplement: Figure 3—figure supplement 5—source data 1. [file elife-79346-fig3-figsupp5-data1.zip › Figure3-figure supplement 5-source data 1/ponceau_WT_invertase_labeled.tif]

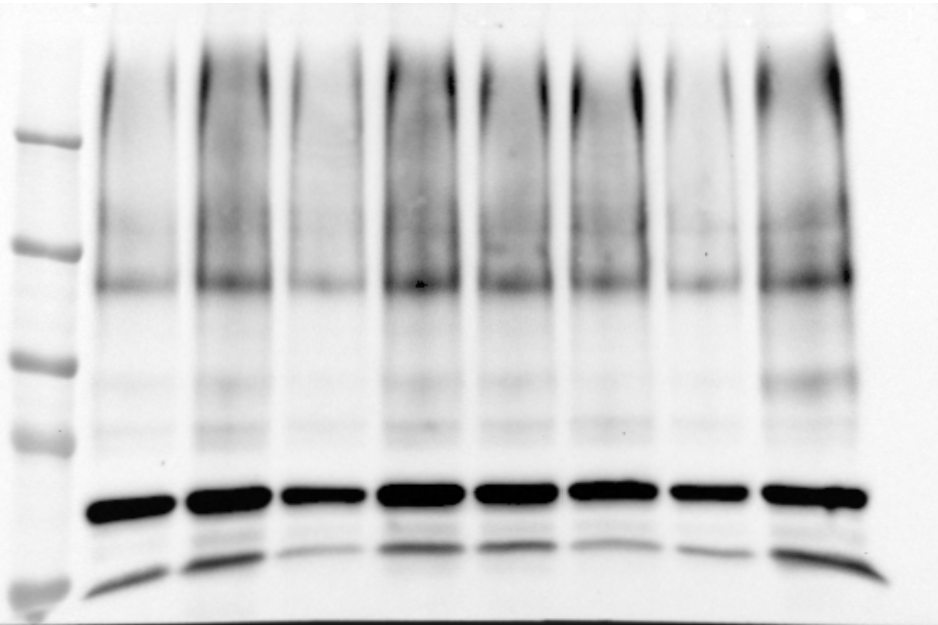

Supplement: Figure 3—figure supplement 5—source data 1. [file elife-79346-fig3-figsupp5-data1.zip › Figure3-figure supplement 5-source data 1/uncropped_blot_WT_CPY.tif]

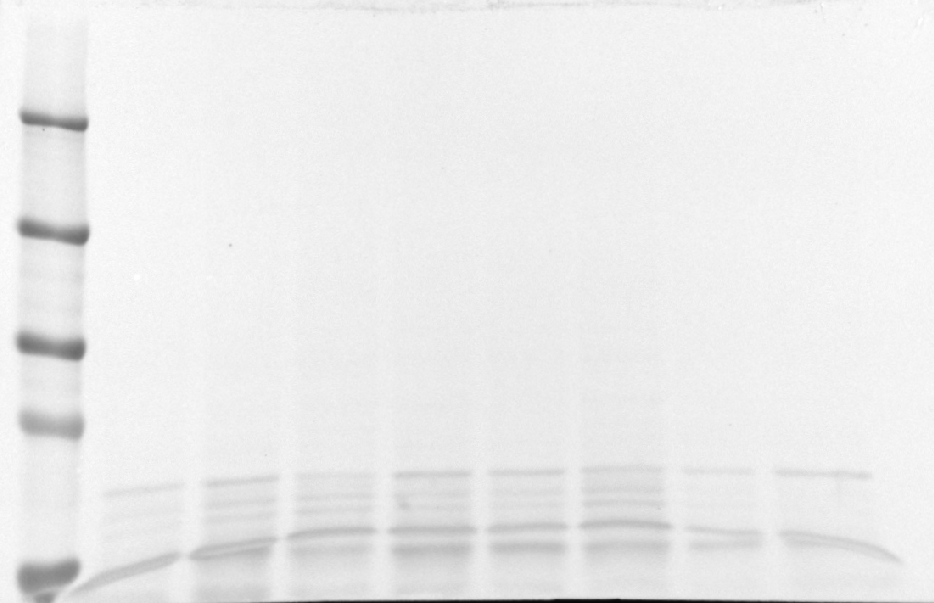

Supplement: Figure 3—figure supplement 5—source data 1. [file elife-79346-fig3-figsupp5-data1.zip › Figure3-figure supplement 5-source data 1/ponceau_WT_CPY.tif]

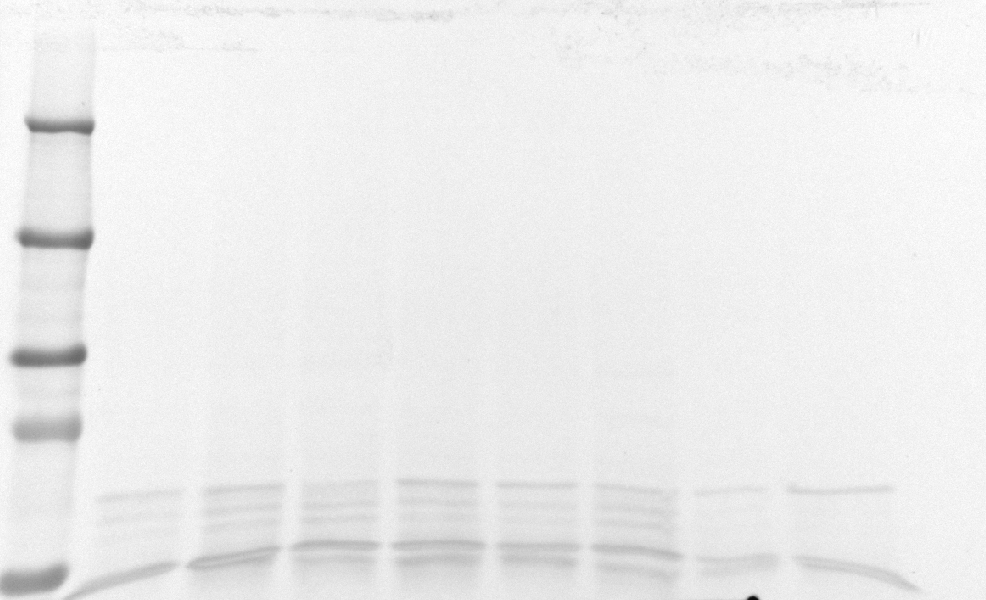

Supplement: Figure 3—figure supplement 5—source data 1. [file elife-79346-fig3-figsupp5-data1.zip › Figure3-figure supplement 5-source data 1/ponceau_WT_invertase.tif]

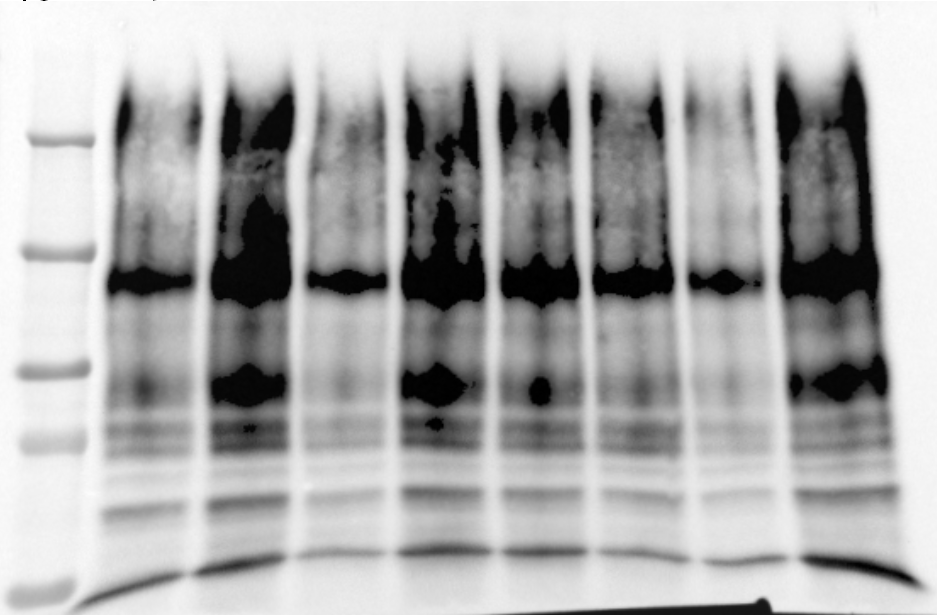

Supplement: Figure 3—figure supplement 5—source data 1. [file elife-79346-fig3-figsupp5-data1.zip › Figure3-figure supplement 5-source data 1/uncropped_blot_WT_invertase.tif]

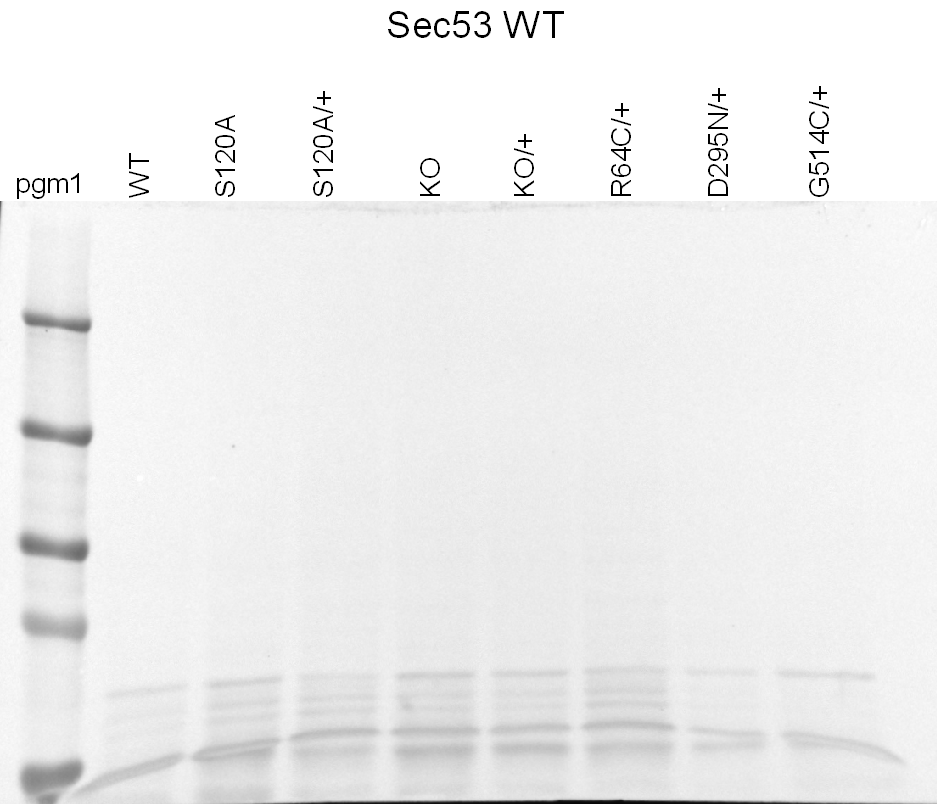

Supplement: Figure 3—figure supplement 5—source data 1. [file elife-79346-fig3-figsupp5-data1.zip › Figure3-figure supplement 5-source data 1/ponceau_WT_CPY_labeled.tif]

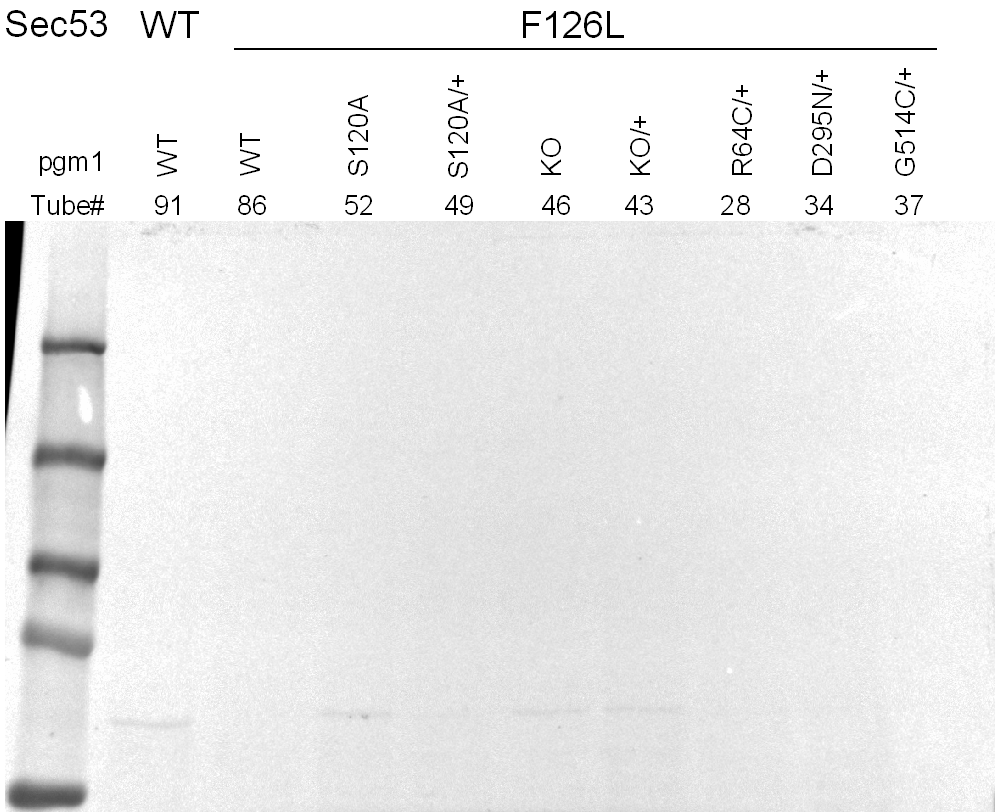

Supplement: Figure 3—figure supplement 6—source data 1. [file elife-79346-fig3-figsupp6-data1.zip › Figure3-figure supplement 6-source data 1/ponceau_F126L_invertase_labeled.tif]

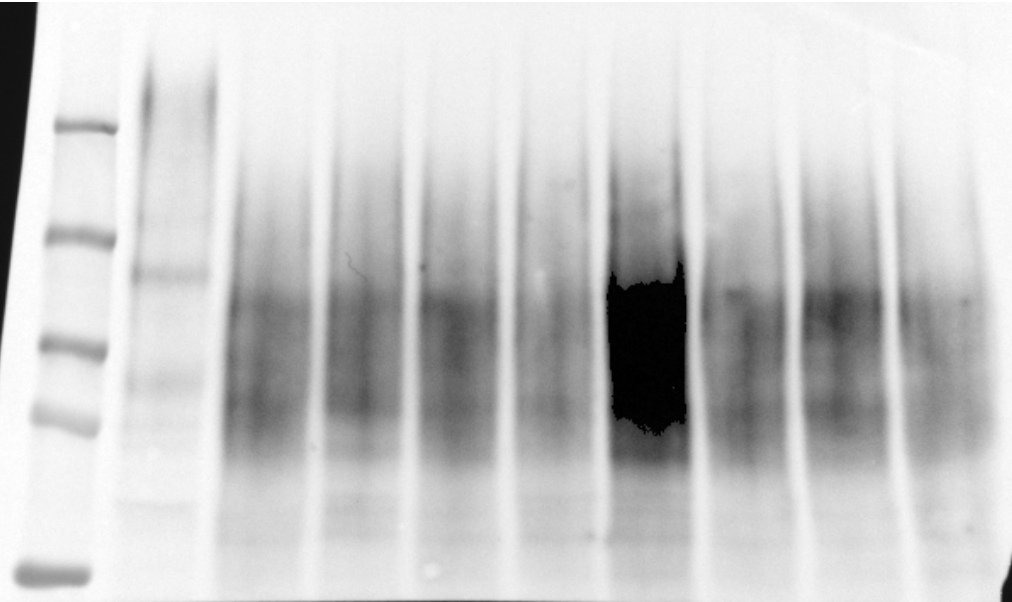

Supplement: Figure 3—figure supplement 6—source data 1. [file elife-79346-fig3-figsupp6-data1.zip › Figure3-figure supplement 6-source data 1/uncropped_blot_F126L_invertase.tif]

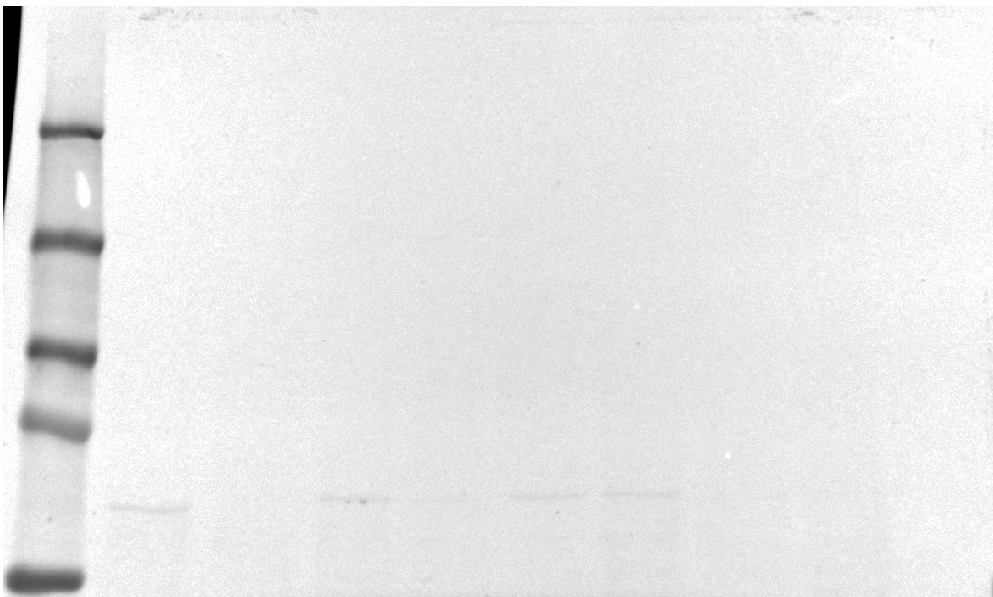

Supplement: Figure 3—figure supplement 6—source data 1. [file elife-79346-fig3-figsupp6-data1.zip › Figure3-figure supplement 6-source data 1/ponceau_F126L_invertase.tif]
