# Supplementary figures and images for: Evolutionary rescue of phosphomannomutase deficiency in yeast models of human disease (part 1 of 2)
Source: eLife. 2022 Oct 10;11:e79346. doi: 10.7554/eLife.79346 (PMC9578706; doi:10.7554/eLife.79346)

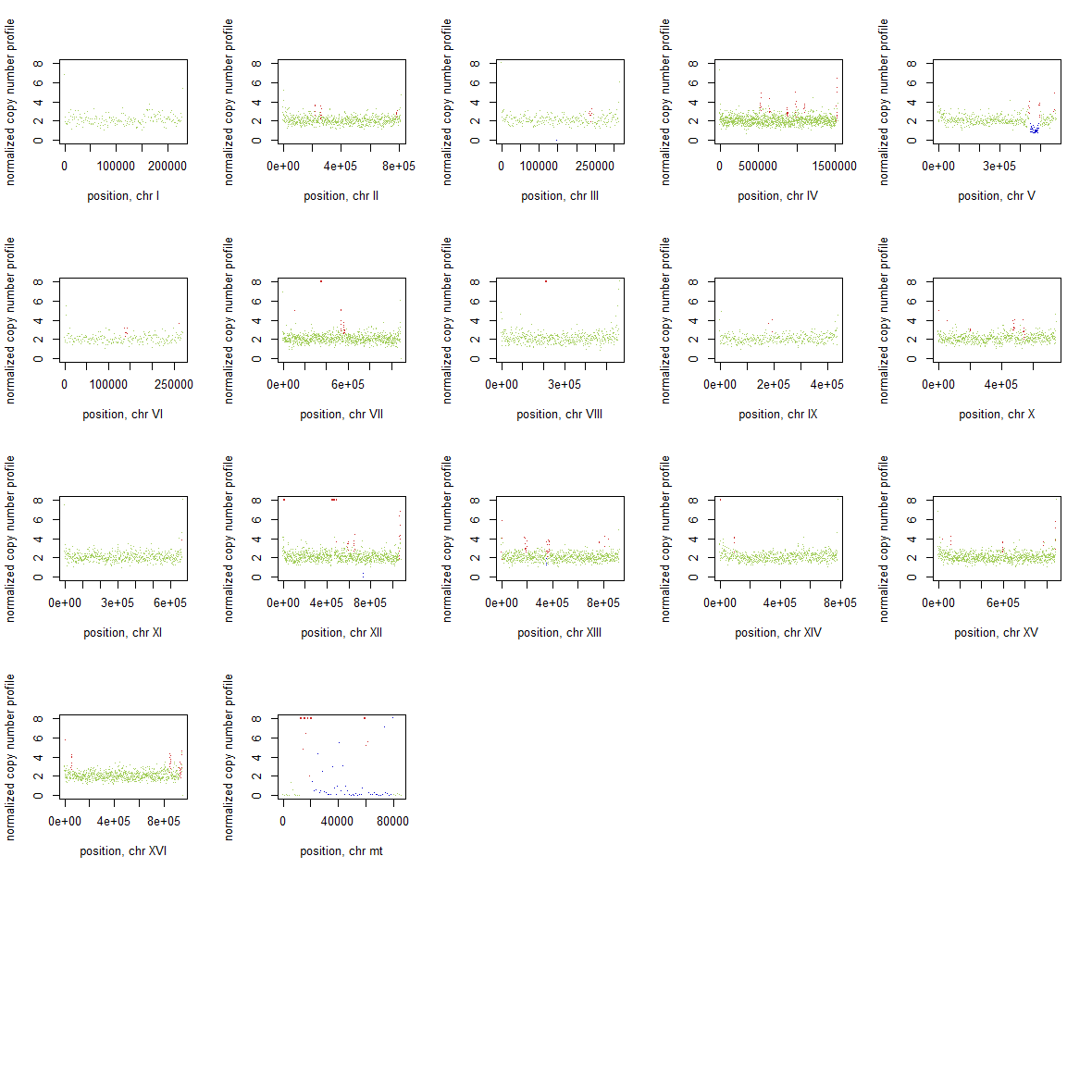

Supplement: Figure 2—source data 2. [file elife-79346-fig2-data2.zip › Figure2-source data 1/pACT1-sec53-V238M/2x_V238M_01.png]

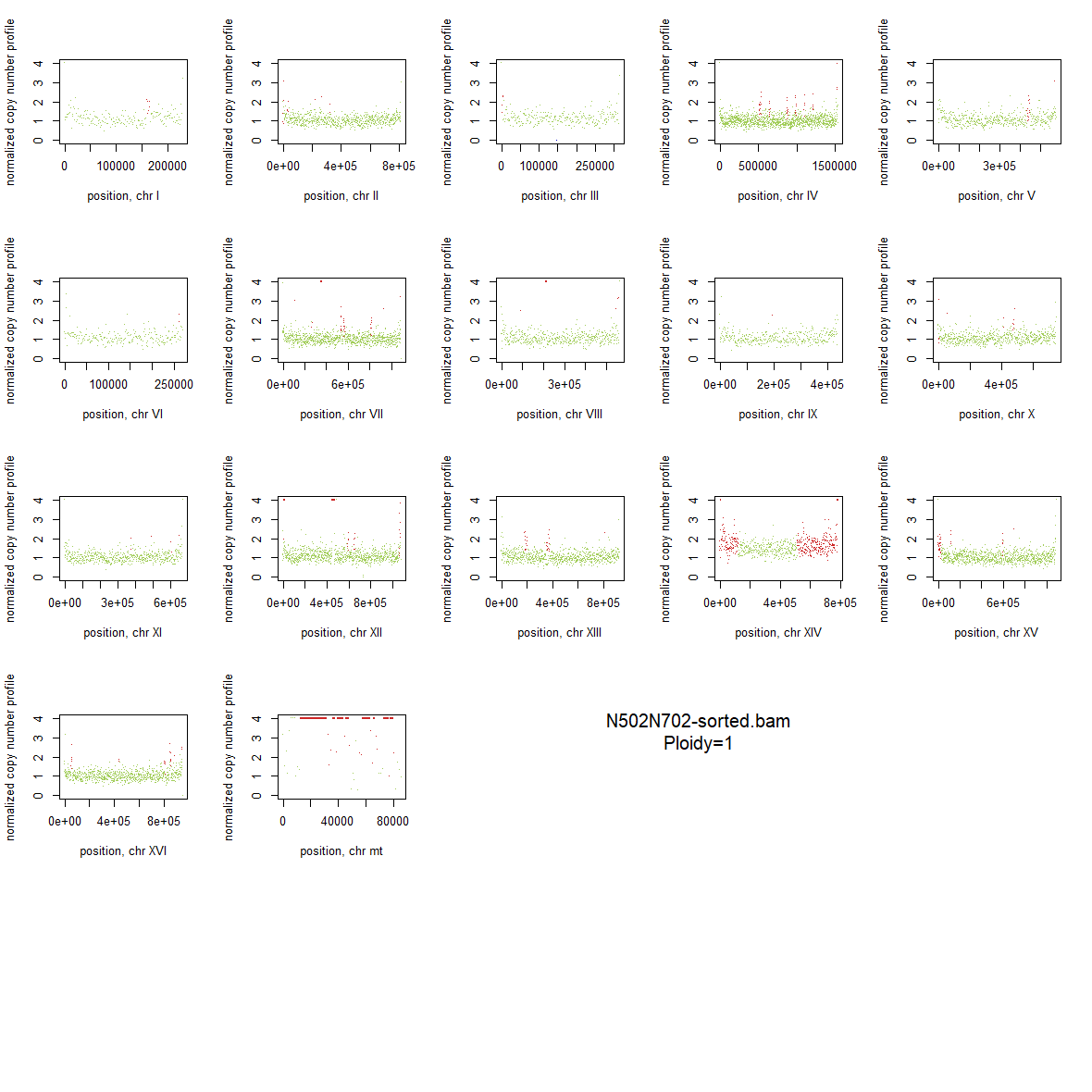

Supplement: Figure 2—source data 2. [file elife-79346-fig2-data2.zip › Figure2-source data 1/pACT1-sec53-V238M/2x_V238M_02.png]

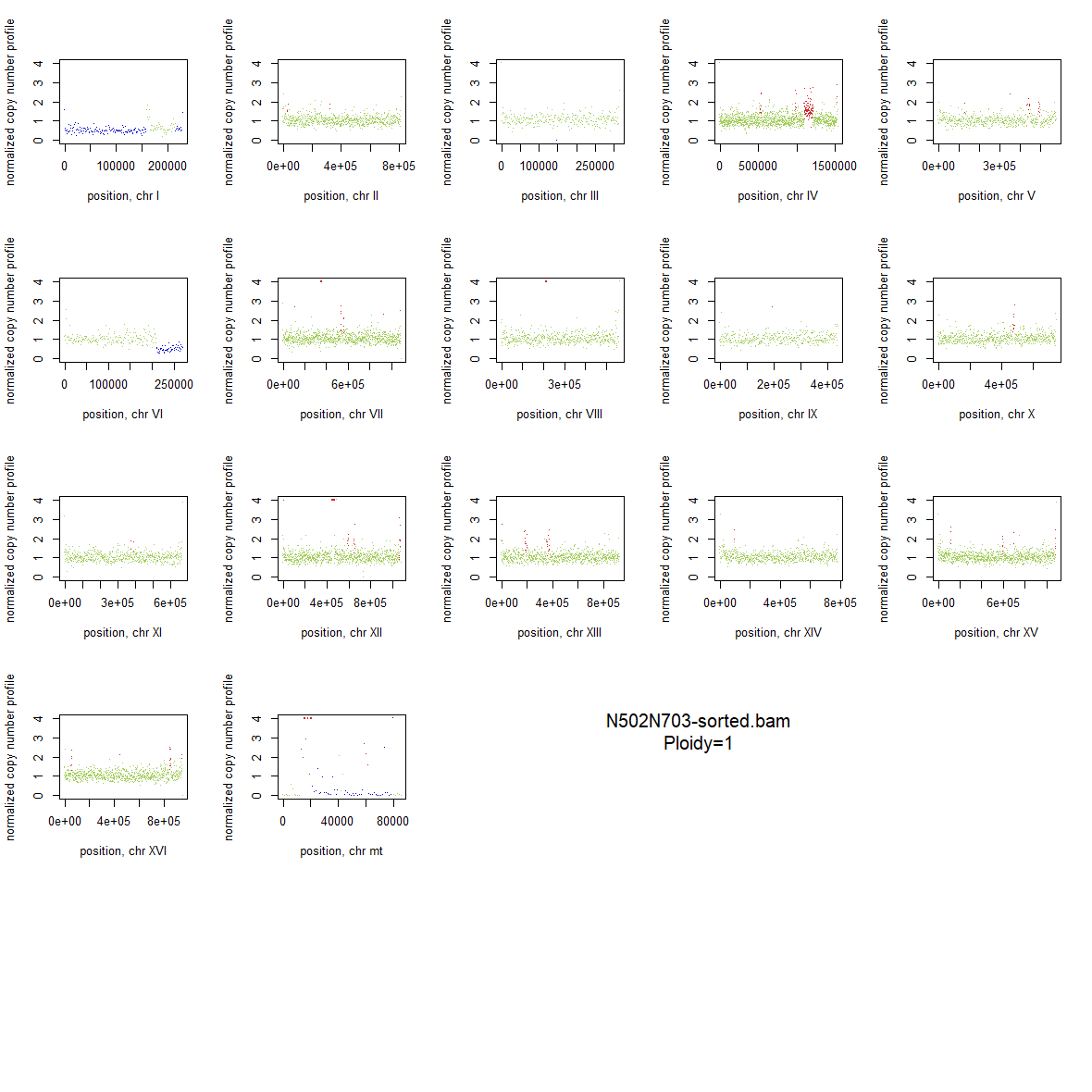

Supplement: Figure 2—source data 2. [file elife-79346-fig2-data2.zip › Figure2-source data 1/pACT1-sec53-V238M/2x_V238M_03.png]

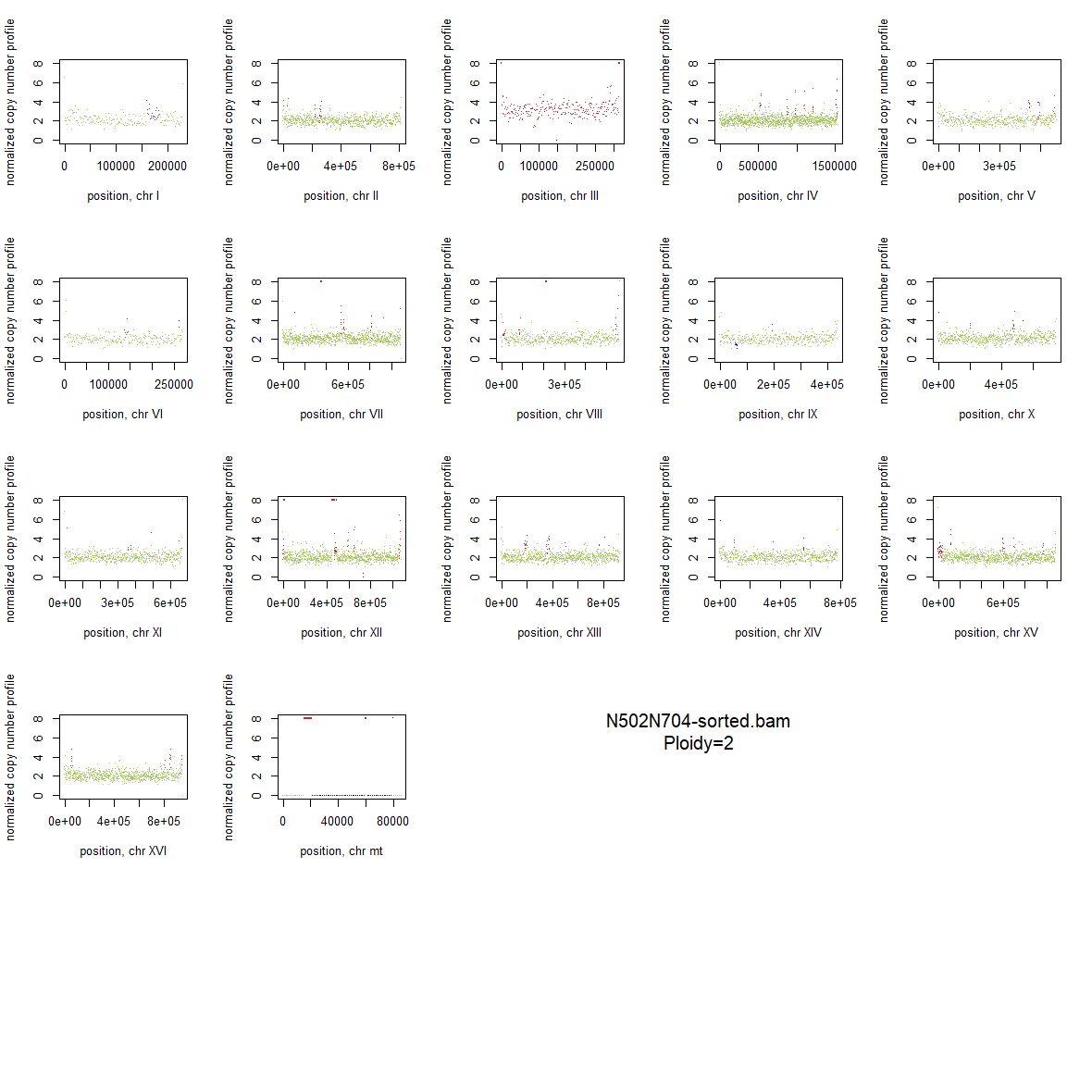

Supplement: Figure 2—source data 2. [file elife-79346-fig2-data2.zip › Figure2-source data 1/pACT1-sec53-V238M/2x_V238M_04.png]

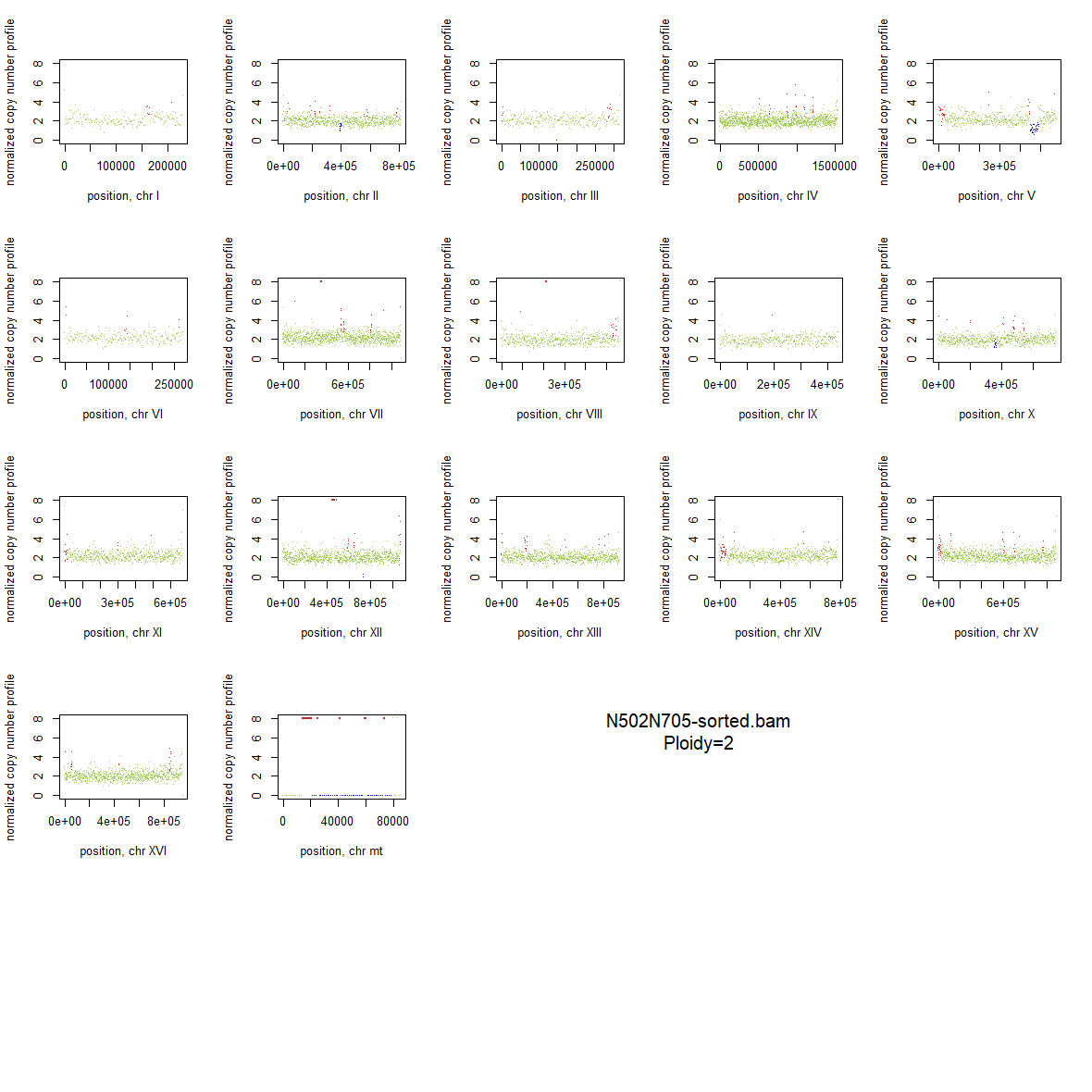

Supplement: Figure 2—source data 2. [file elife-79346-fig2-data2.zip › Figure2-source data 1/pACT1-sec53-V238M/2x_V238M_05.png]

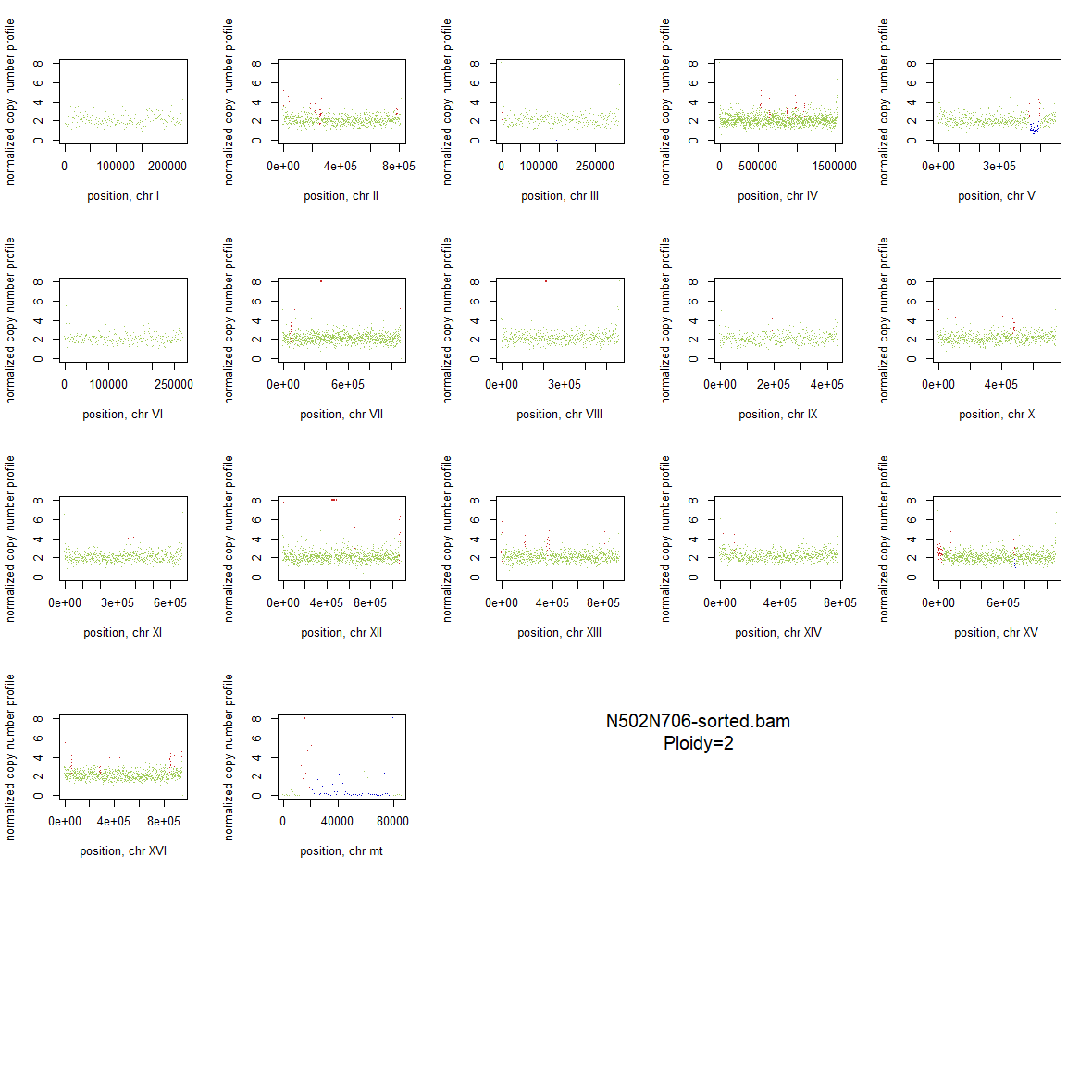

Supplement: Figure 2—source data 2. [file elife-79346-fig2-data2.zip › Figure2-source data 1/pACT1-sec53-V238M/2x_V238M_06.png]

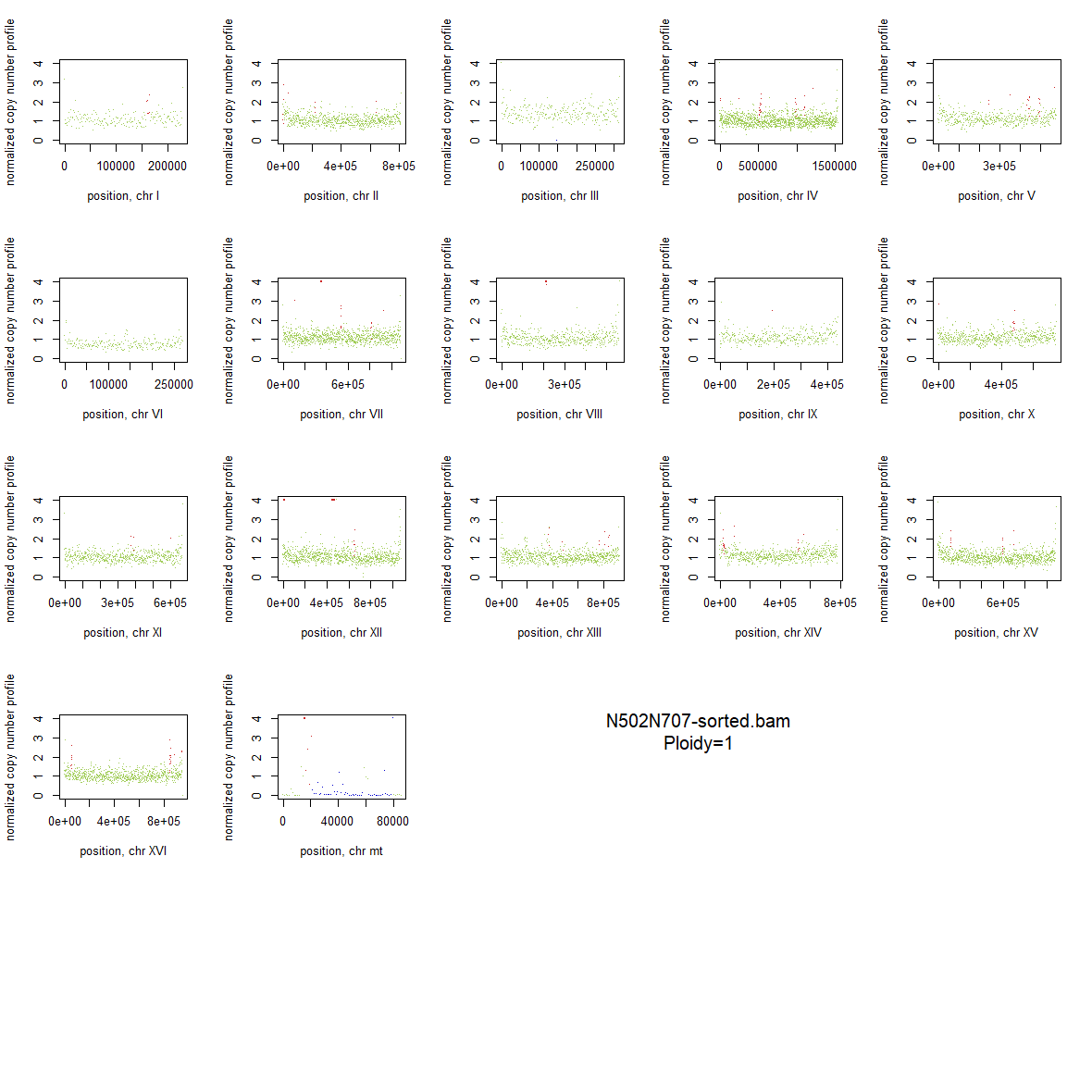

Supplement: Figure 2—source data 2. [file elife-79346-fig2-data2.zip › Figure2-source data 1/pACT1-sec53-V238M/2x_V238M_07.png]

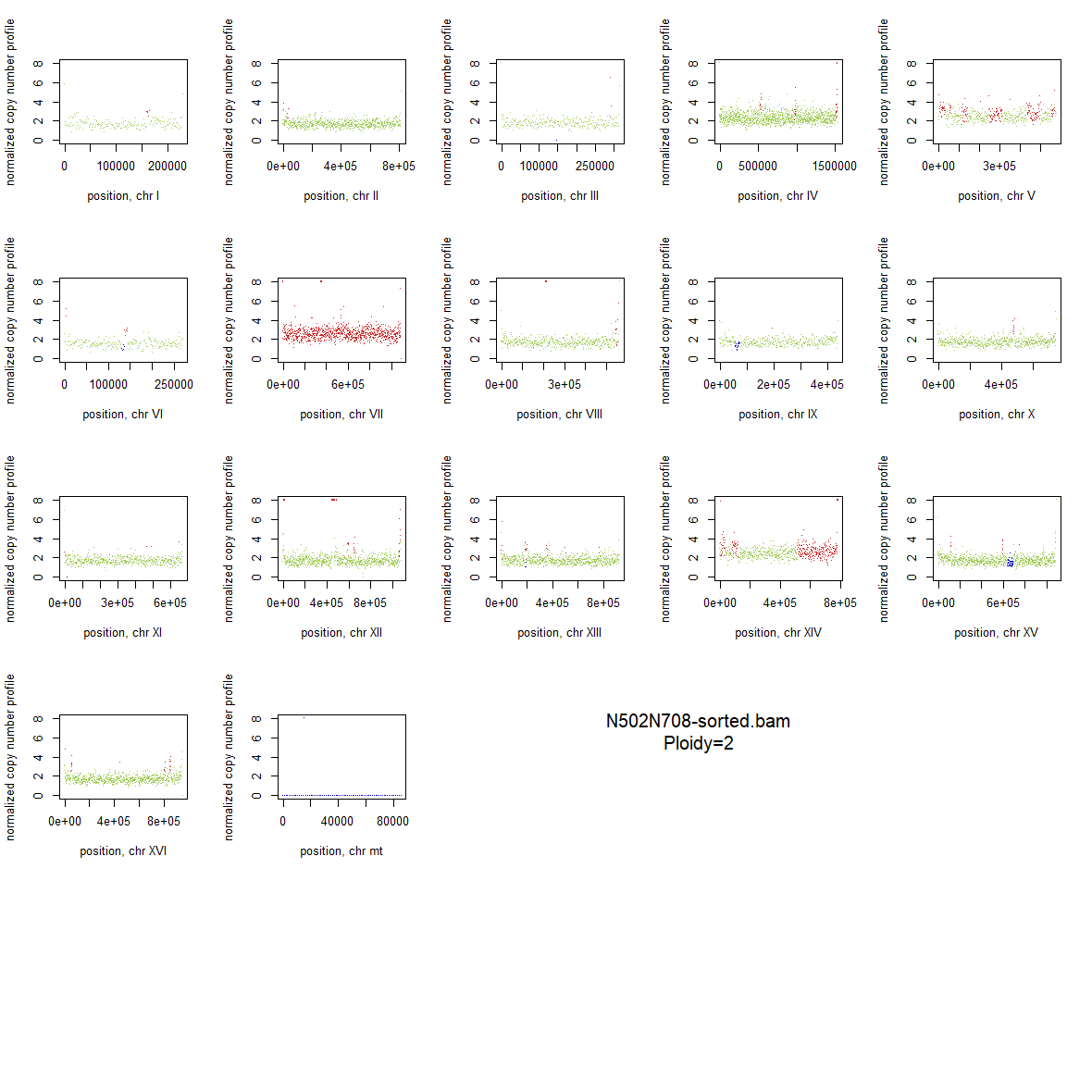

Supplement: Figure 2—source data 2. [file elife-79346-fig2-data2.zip › Figure2-source data 1/pACT1-sec53-V238M/2x_V238M_08.png]

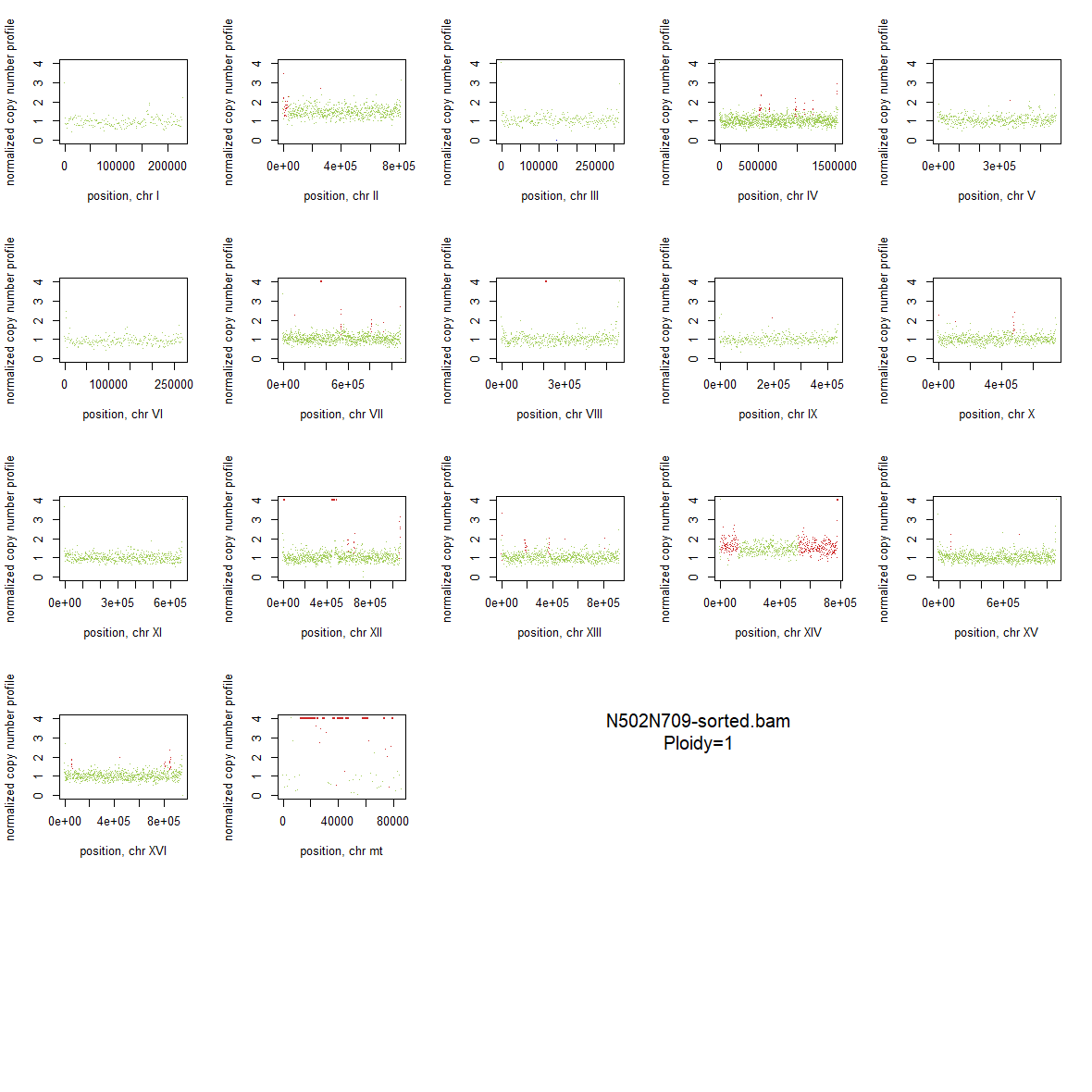

Supplement: Figure 2—source data 2. [file elife-79346-fig2-data2.zip › Figure2-source data 1/pACT1-sec53-V238M/2x_V238M_09.png]

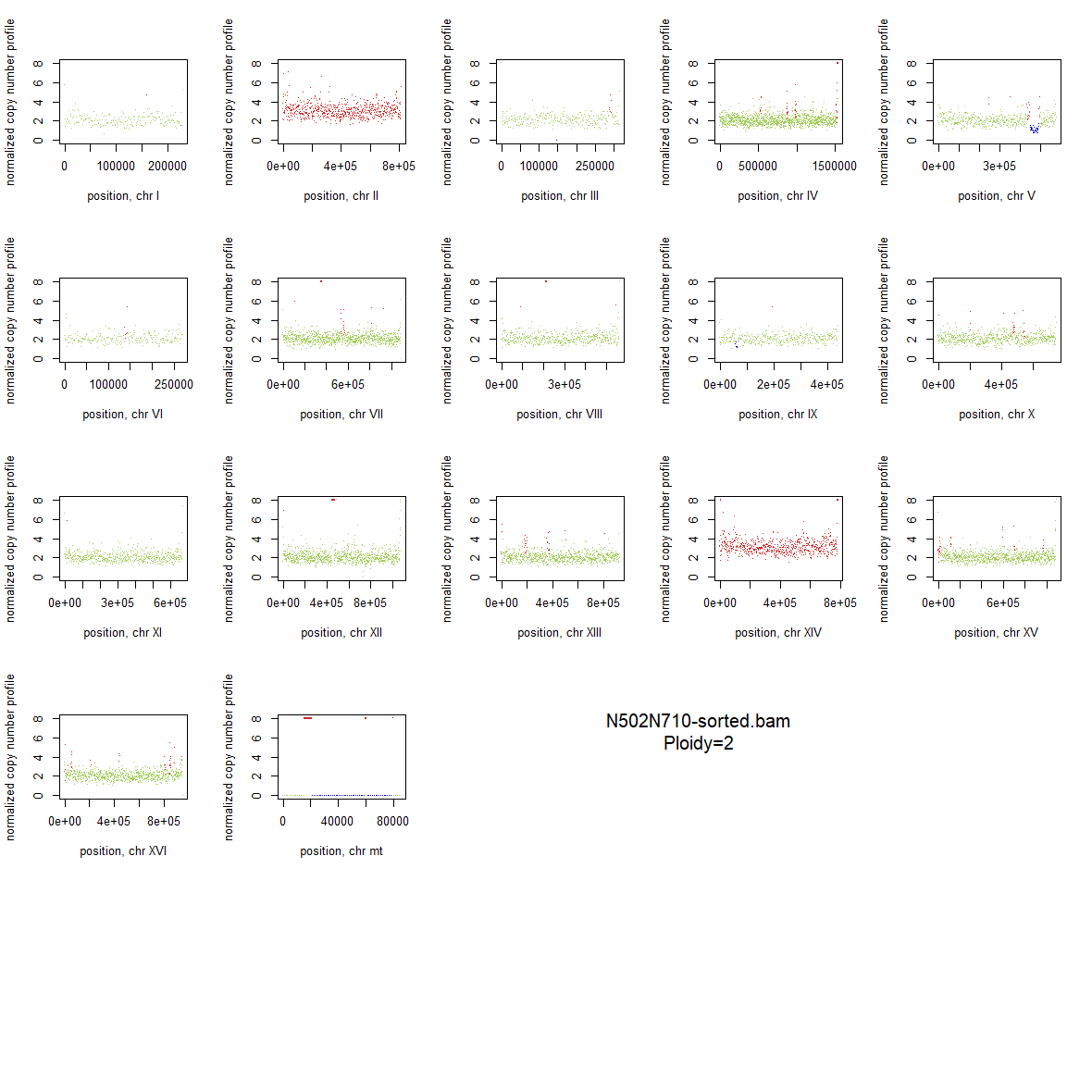

Supplement: Figure 2—source data 2. [file elife-79346-fig2-data2.zip › Figure2-source data 1/pACT1-sec53-V238M/2x_V238M_10.png]

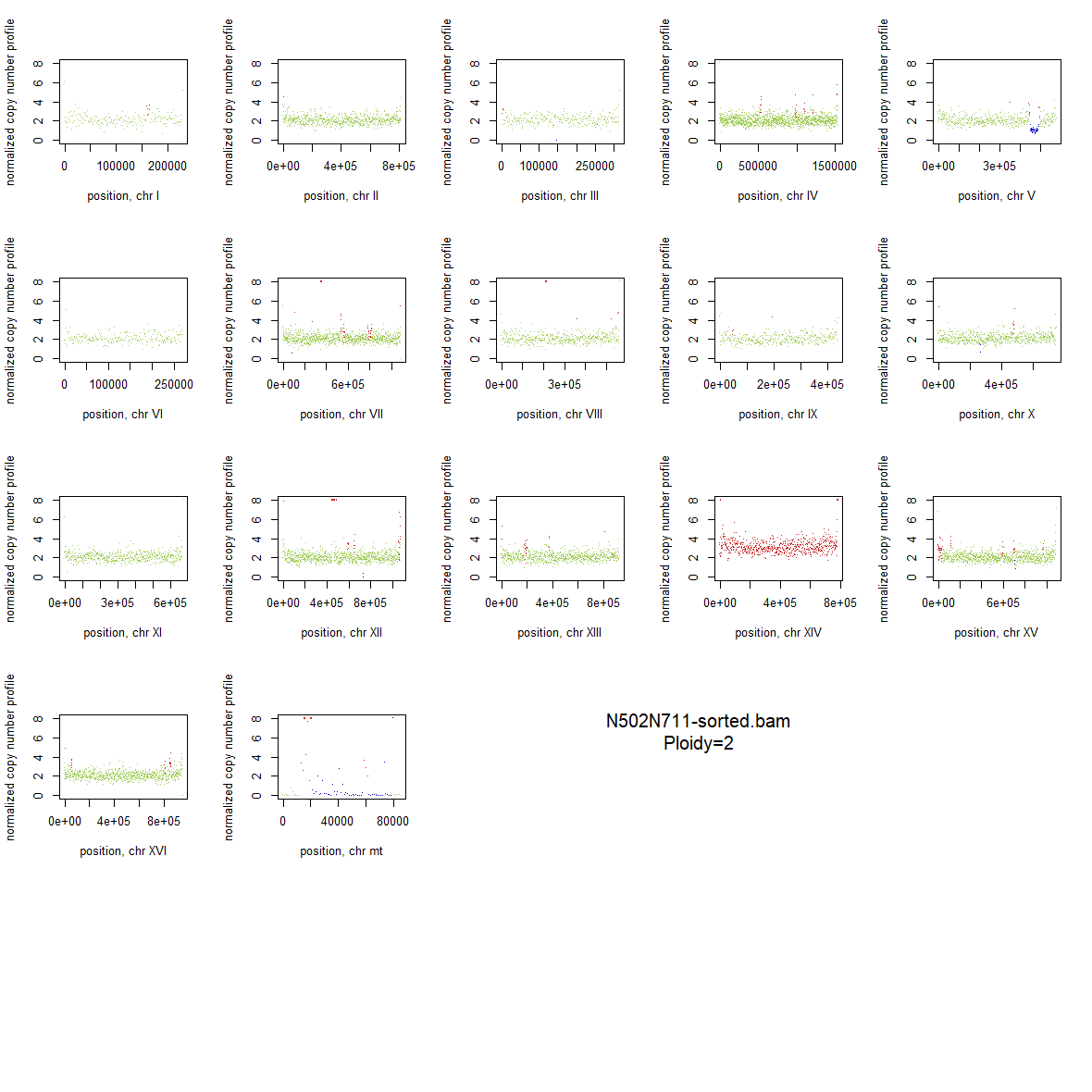

Supplement: Figure 2—source data 2. [file elife-79346-fig2-data2.zip › Figure2-source data 1/pACT1-sec53-V238M/2x_V238M_11.png]

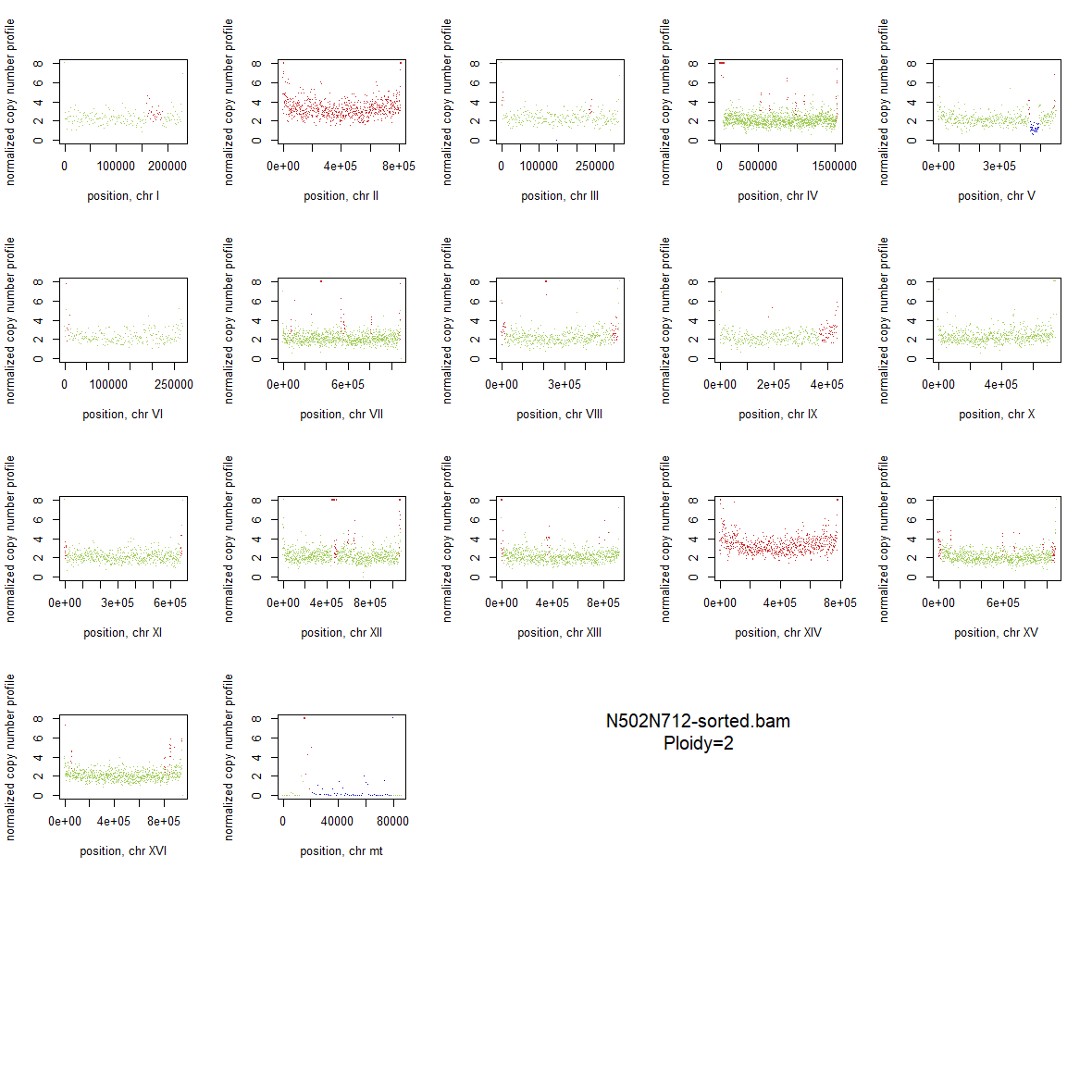

Supplement: Figure 2—source data 2. [file elife-79346-fig2-data2.zip › Figure2-source data 1/pACT1-sec53-V238M/2x_V238M_12.png]

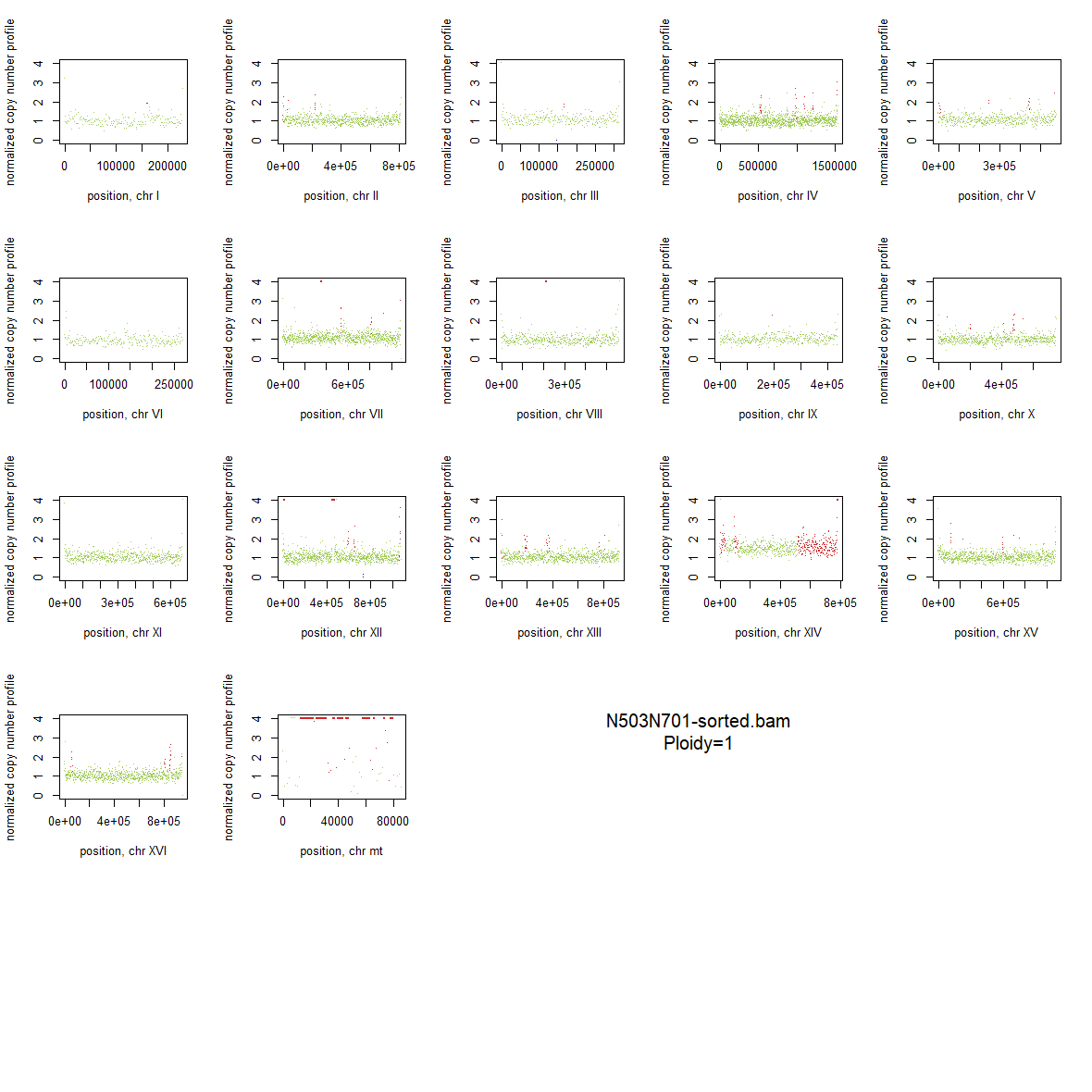

Supplement: Figure 2—source data 2. [file elife-79346-fig2-data2.zip › Figure2-source data 1/pACT1-sec53-V238M/2x_V238M_13.png]

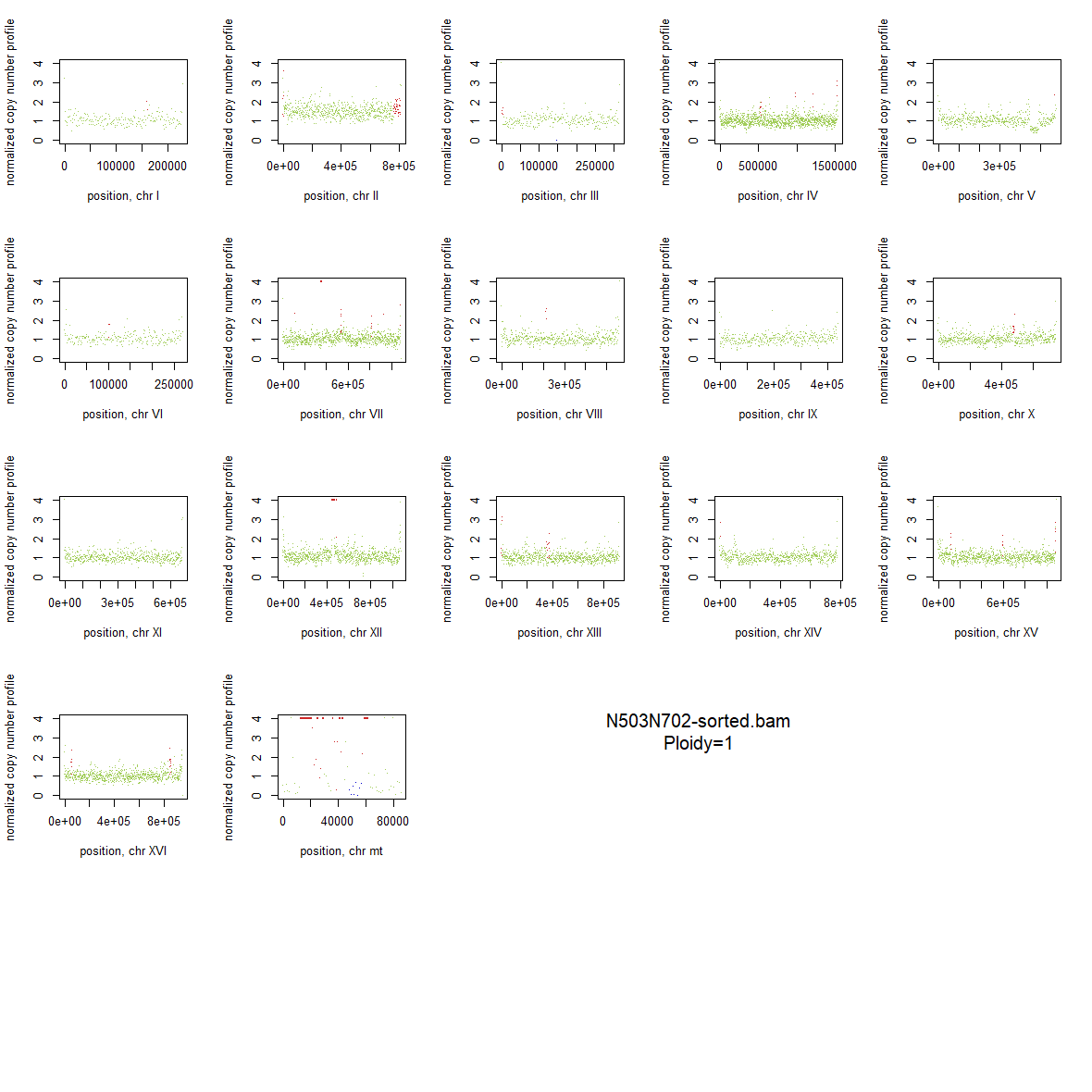

Supplement: Figure 2—source data 2. [file elife-79346-fig2-data2.zip › Figure2-source data 1/pACT1-sec53-V238M/2x_V238M_14.png]

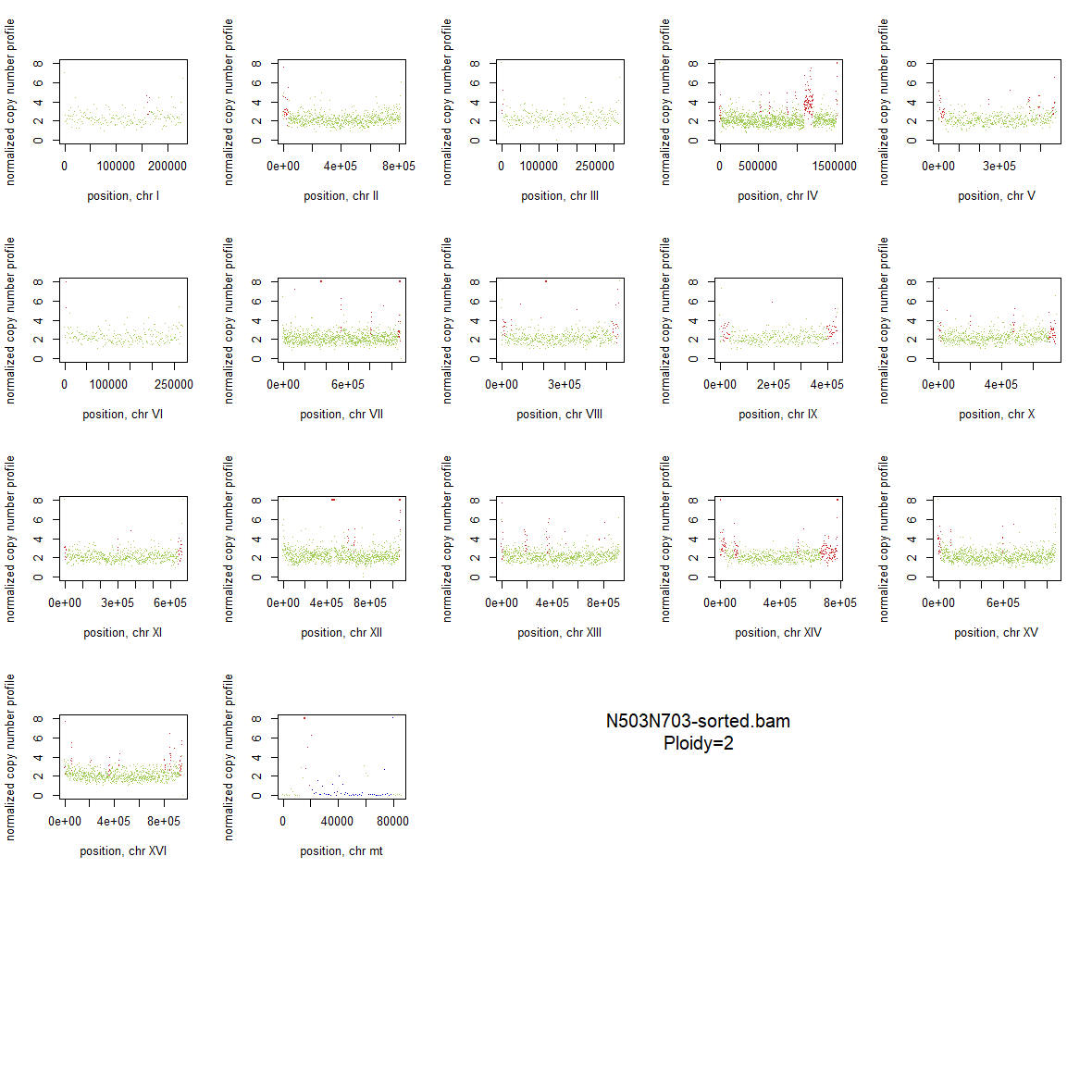

Supplement: Figure 2—source data 2. [file elife-79346-fig2-data2.zip › Figure2-source data 1/pACT1-sec53-V238M/2x_V238M_15.png]

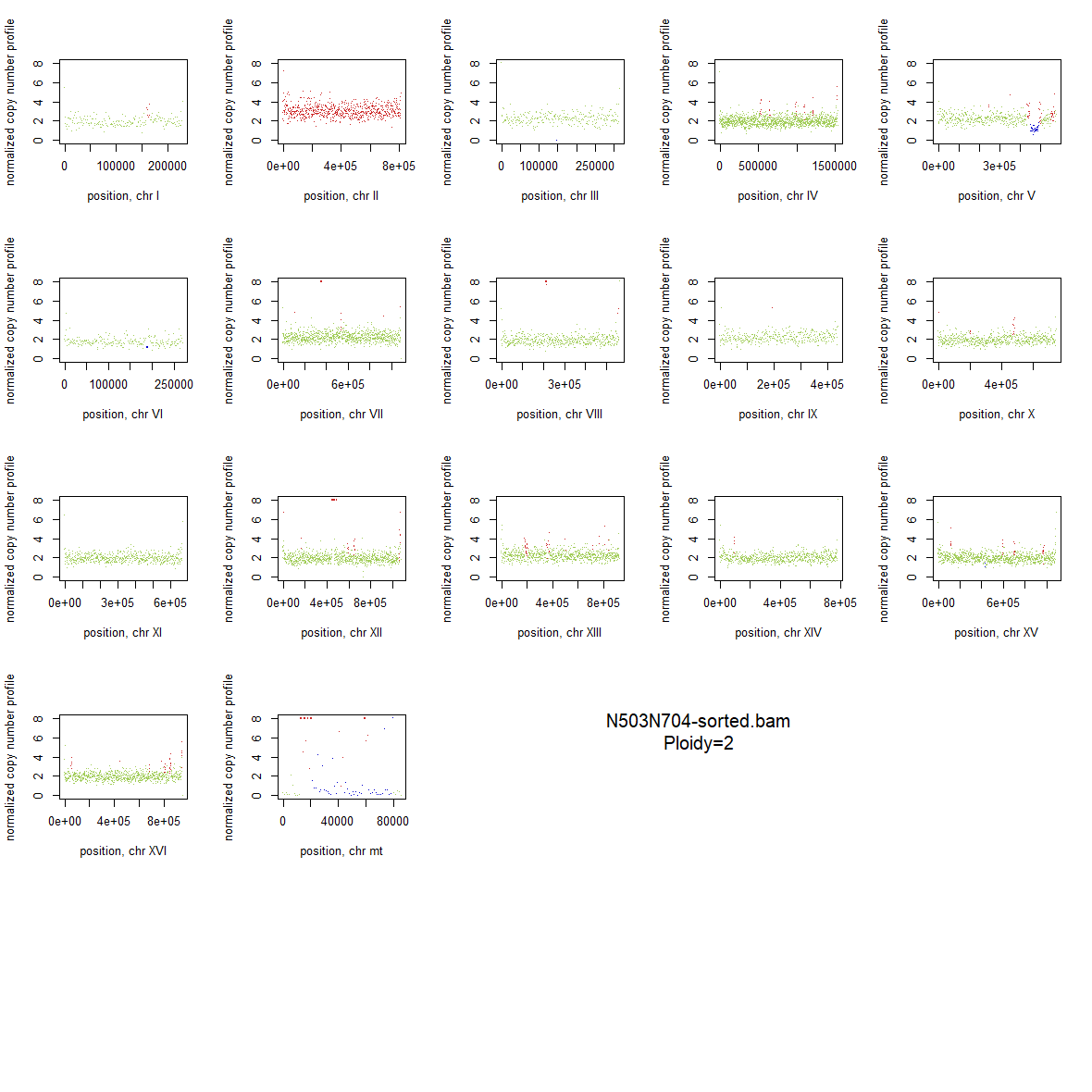

Supplement: Figure 2—source data 2. [file elife-79346-fig2-data2.zip › Figure2-source data 1/pACT1-sec53-V238M/2x_V238M_16.png]

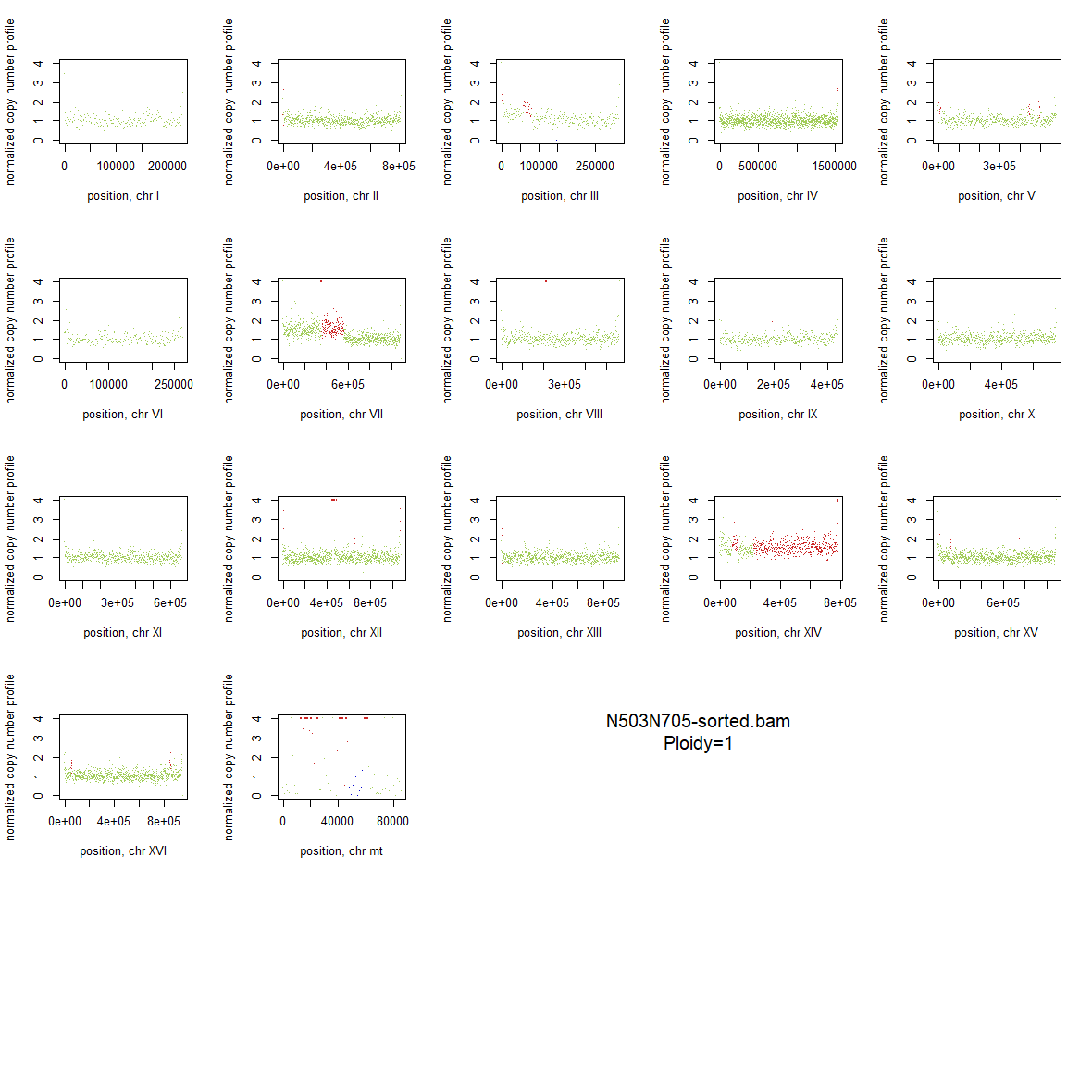

Supplement: Figure 2—source data 2. [file elife-79346-fig2-data2.zip › Figure2-source data 1/pACT1-sec53-V238M/2x_V238M_17.png]

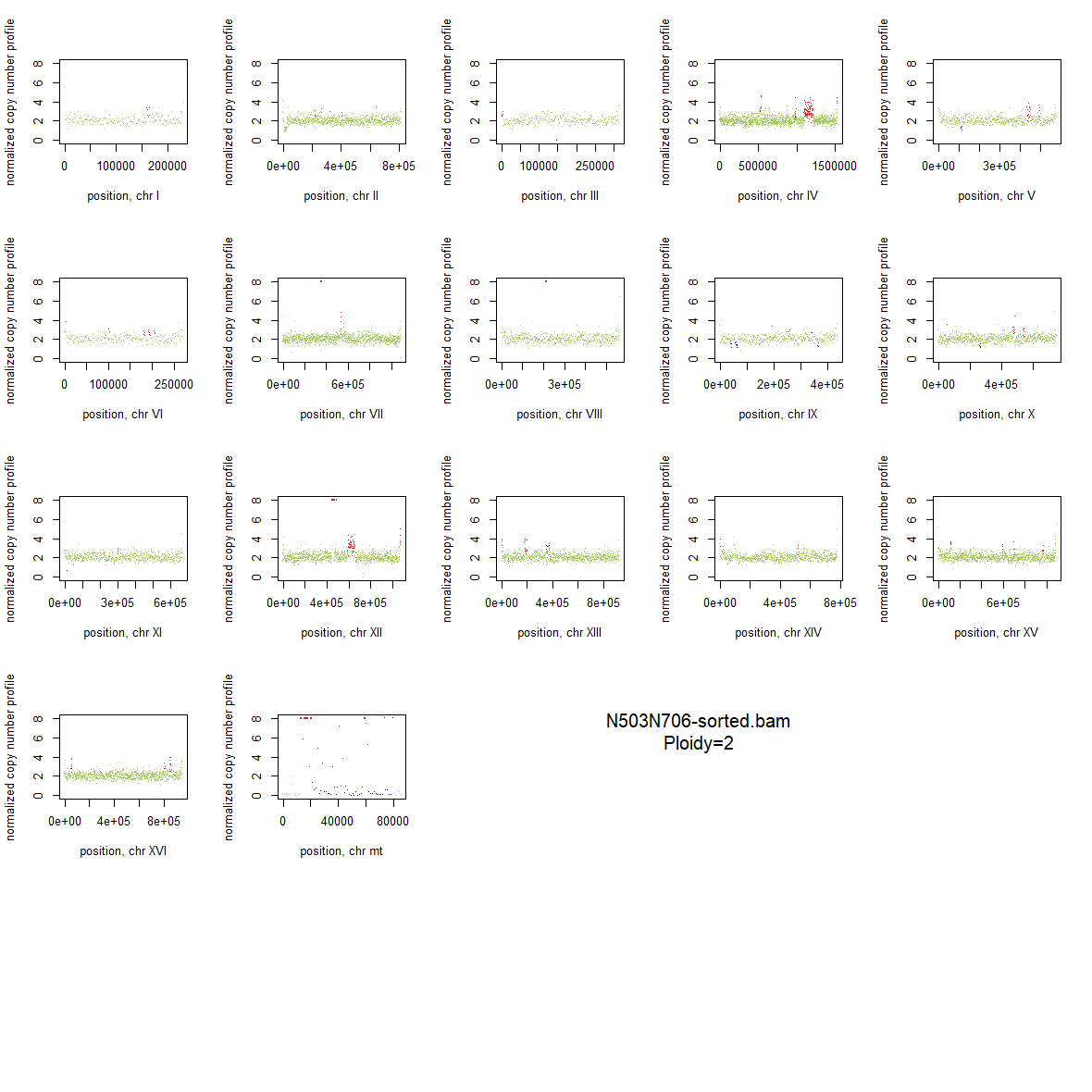

Supplement: Figure 2—source data 2. [file elife-79346-fig2-data2.zip › Figure2-source data 1/pACT1-sec53-V238M/2x_V238M_18.png]

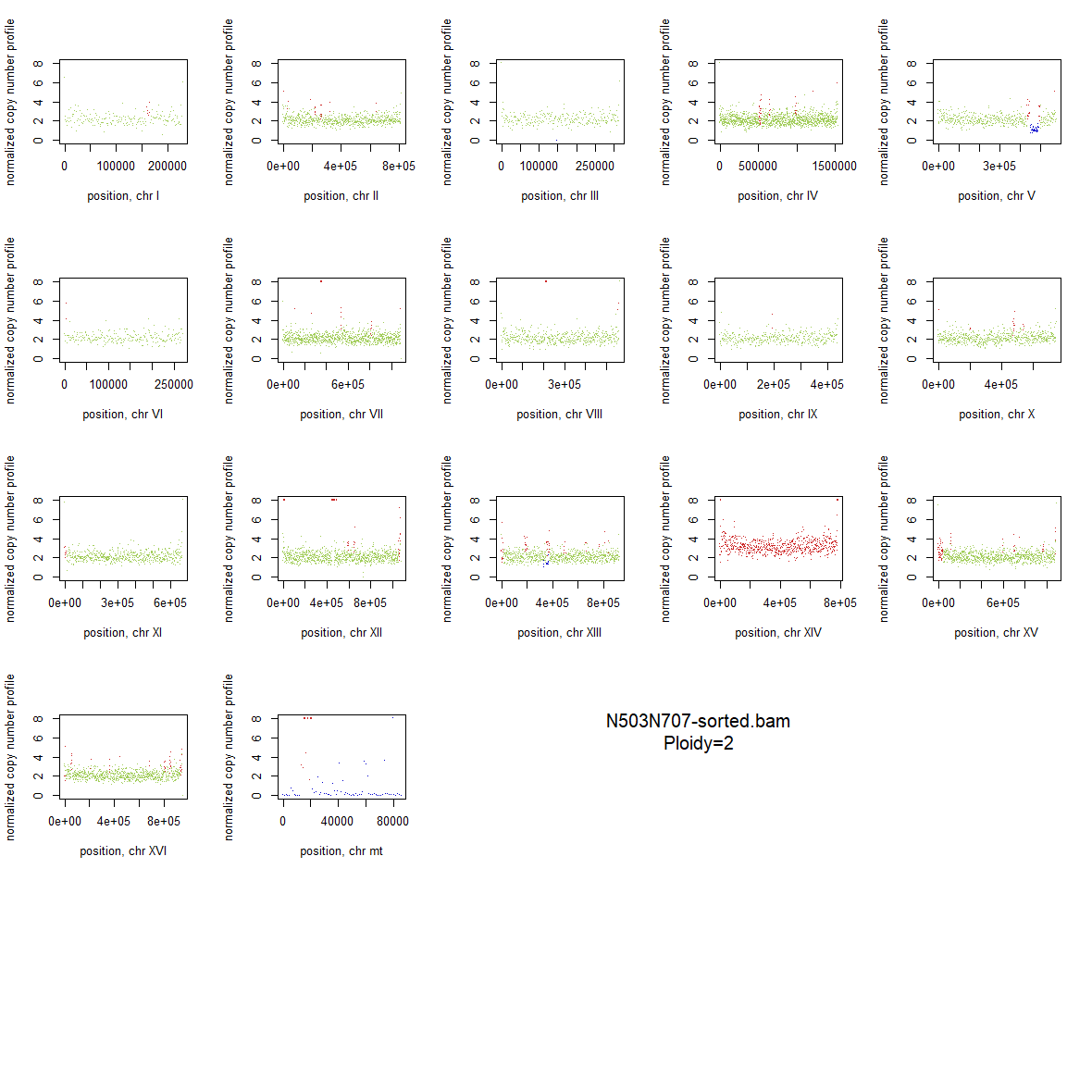

Supplement: Figure 2—source data 2. [file elife-79346-fig2-data2.zip › Figure2-source data 1/pACT1-sec53-V238M/2x_V238M_19.png]

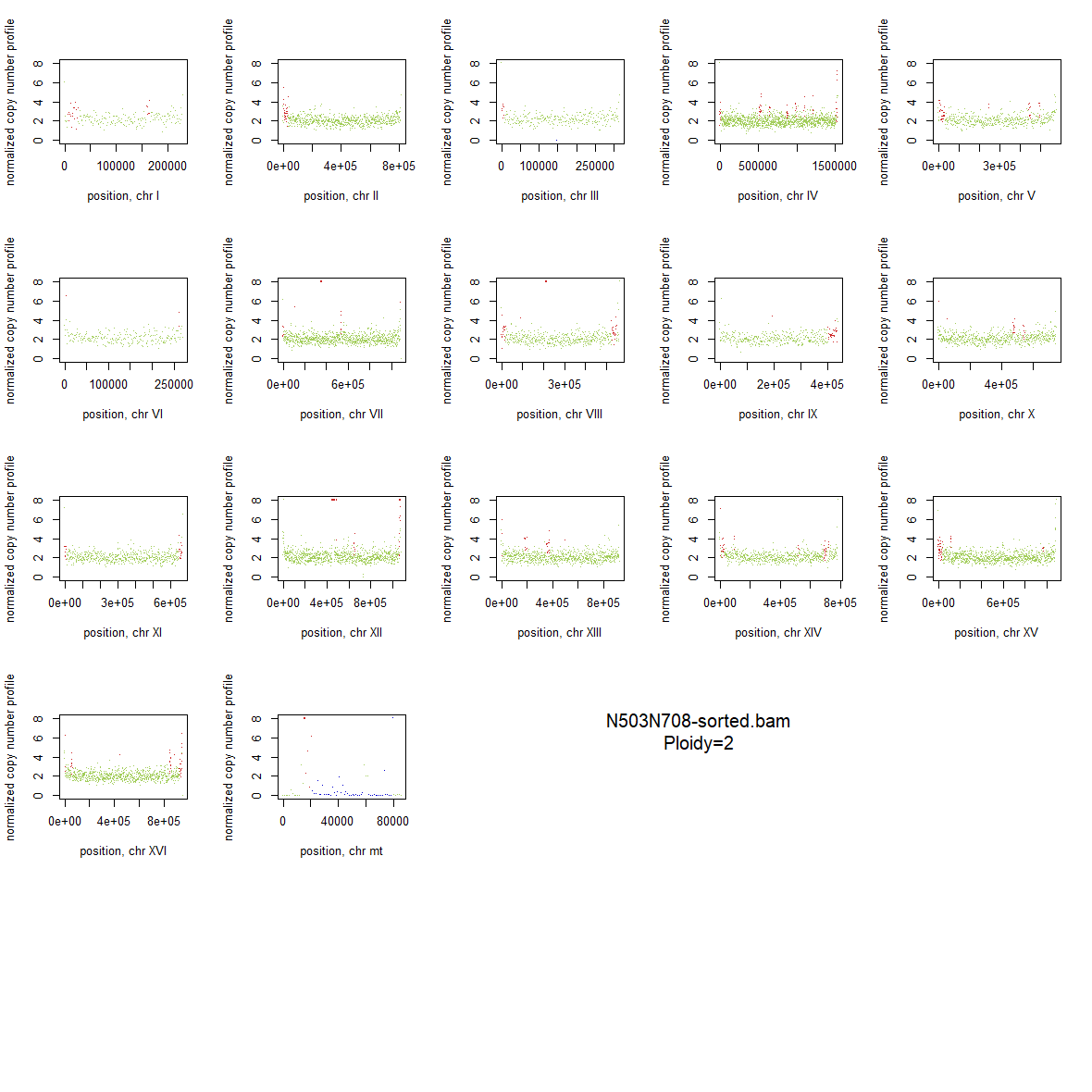

Supplement: Figure 2—source data 2. [file elife-79346-fig2-data2.zip › Figure2-source data 1/pACT1-sec53-V238M/2x_V238M_20.png]

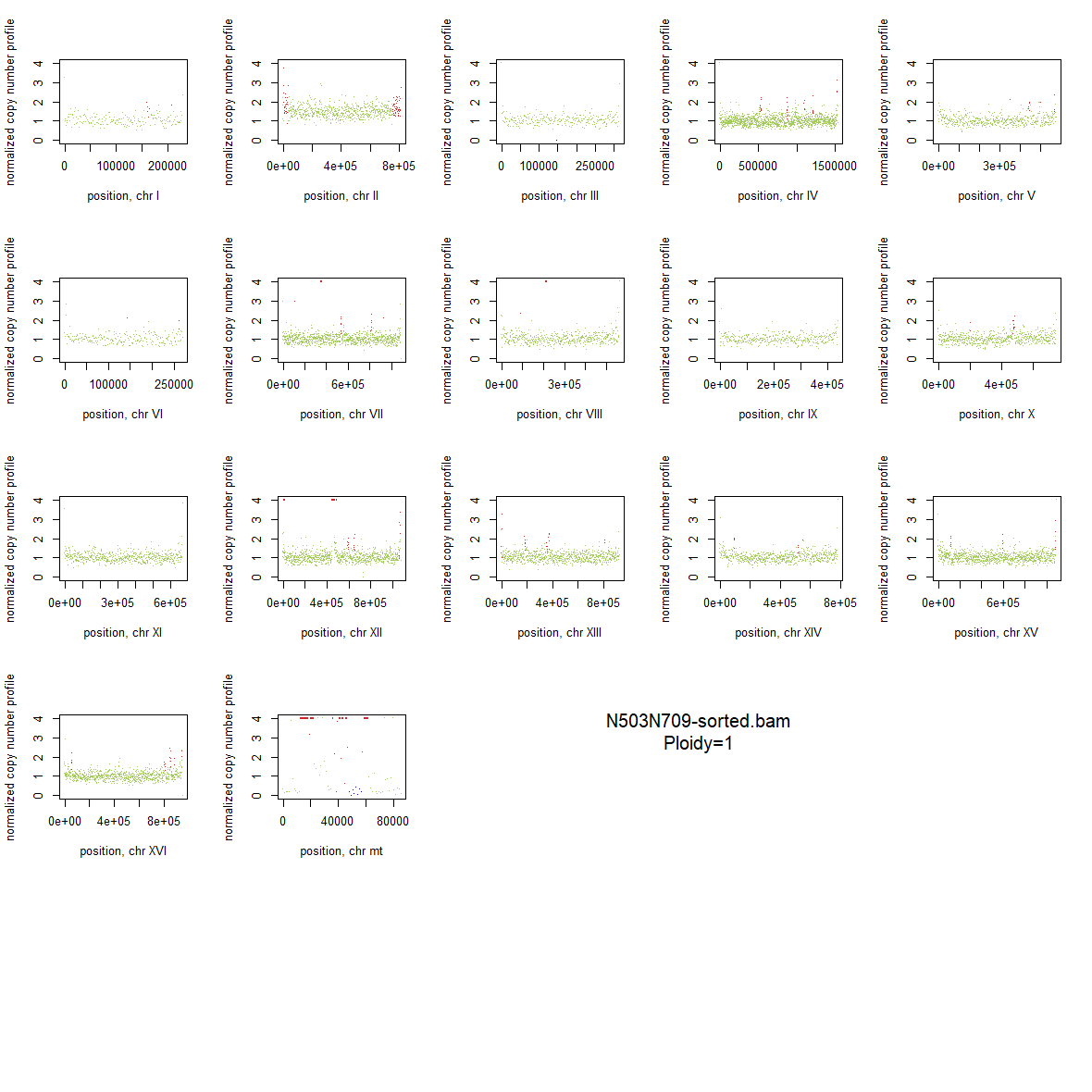

Supplement: Figure 2—source data 2. [file elife-79346-fig2-data2.zip › Figure2-source data 1/pACT1-sec53-V238M/2x_V238M_21.png]

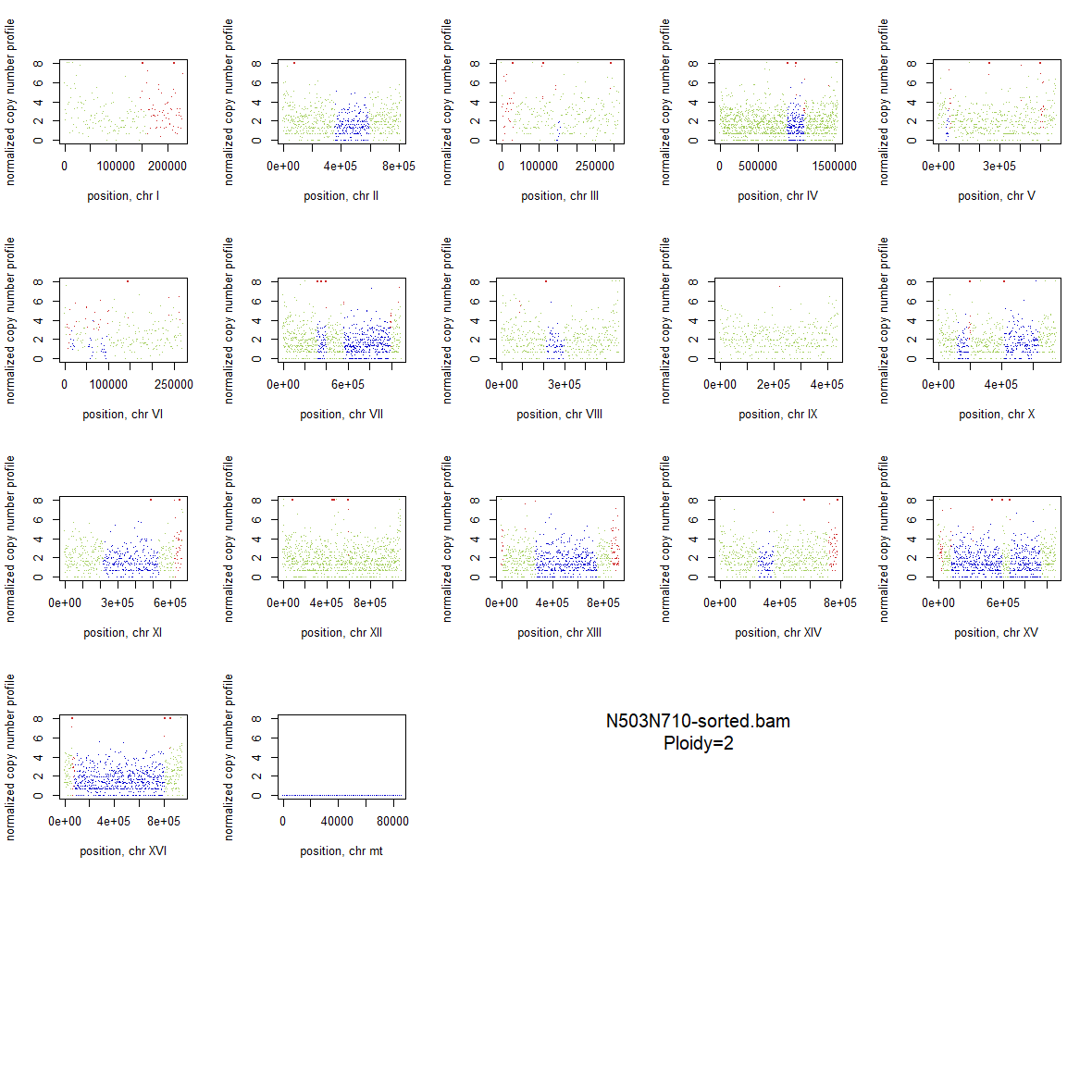

Supplement: Figure 2—source data 2. [file elife-79346-fig2-data2.zip › Figure2-source data 1/pACT1-sec53-V238M/2x_V238M_22.png]

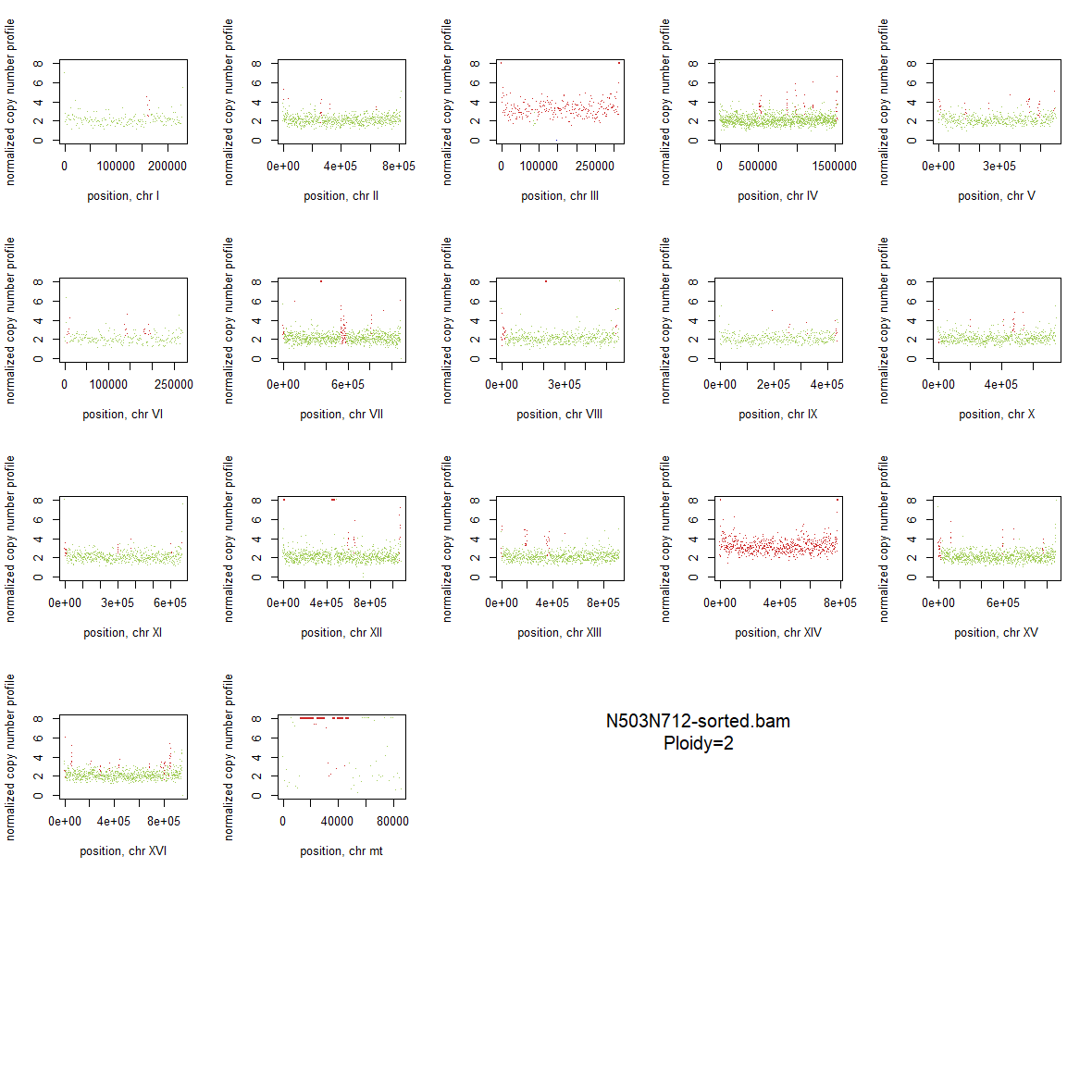

Supplement: Figure 2—source data 2. [file elife-79346-fig2-data2.zip › Figure2-source data 1/pACT1-sec53-V238M/2x_V238M_23.png]

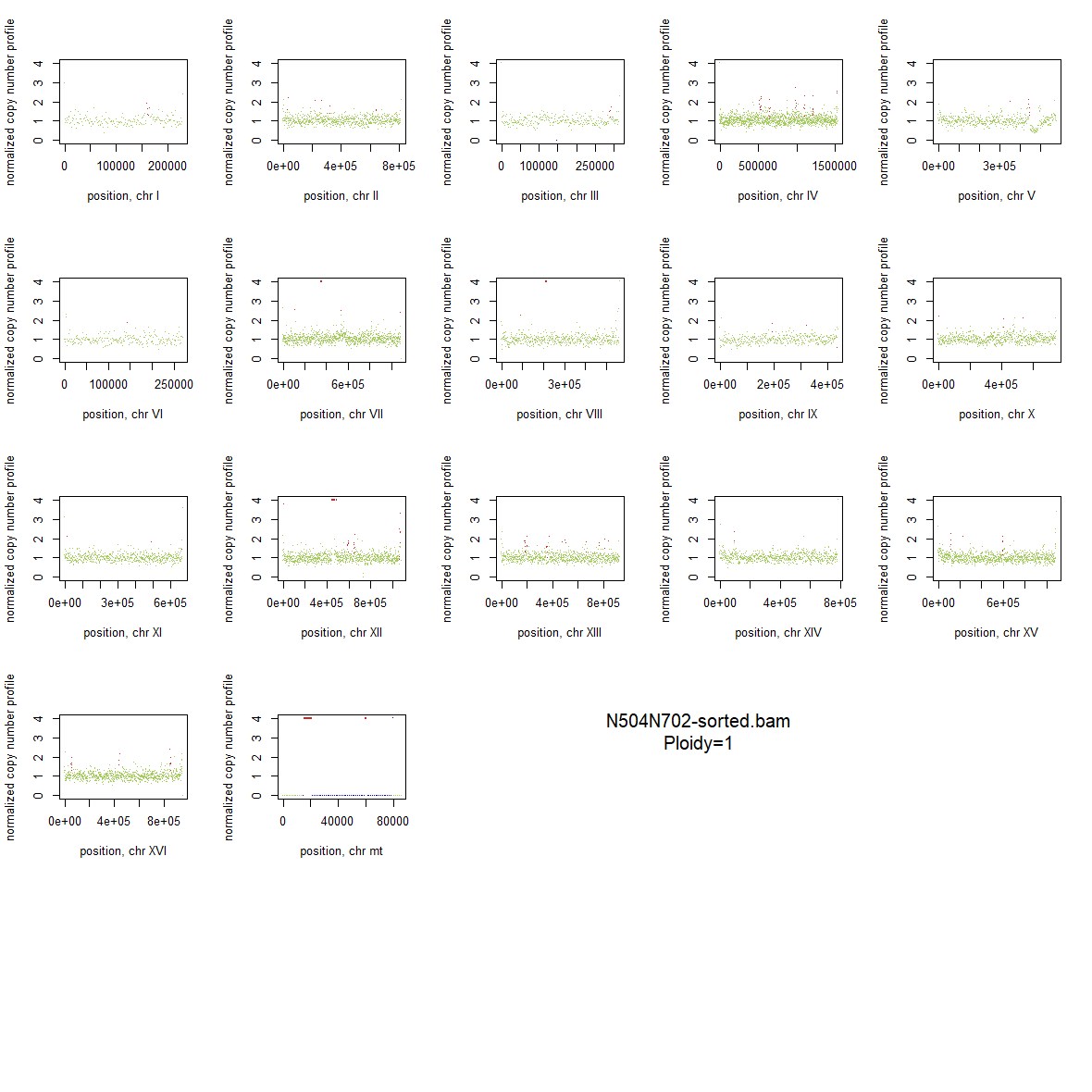

Supplement: Figure 2—source data 2. [file elife-79346-fig2-data2.zip › Figure2-source data 1/pACT1-sec53-V238M/2x_V238M_24.png]

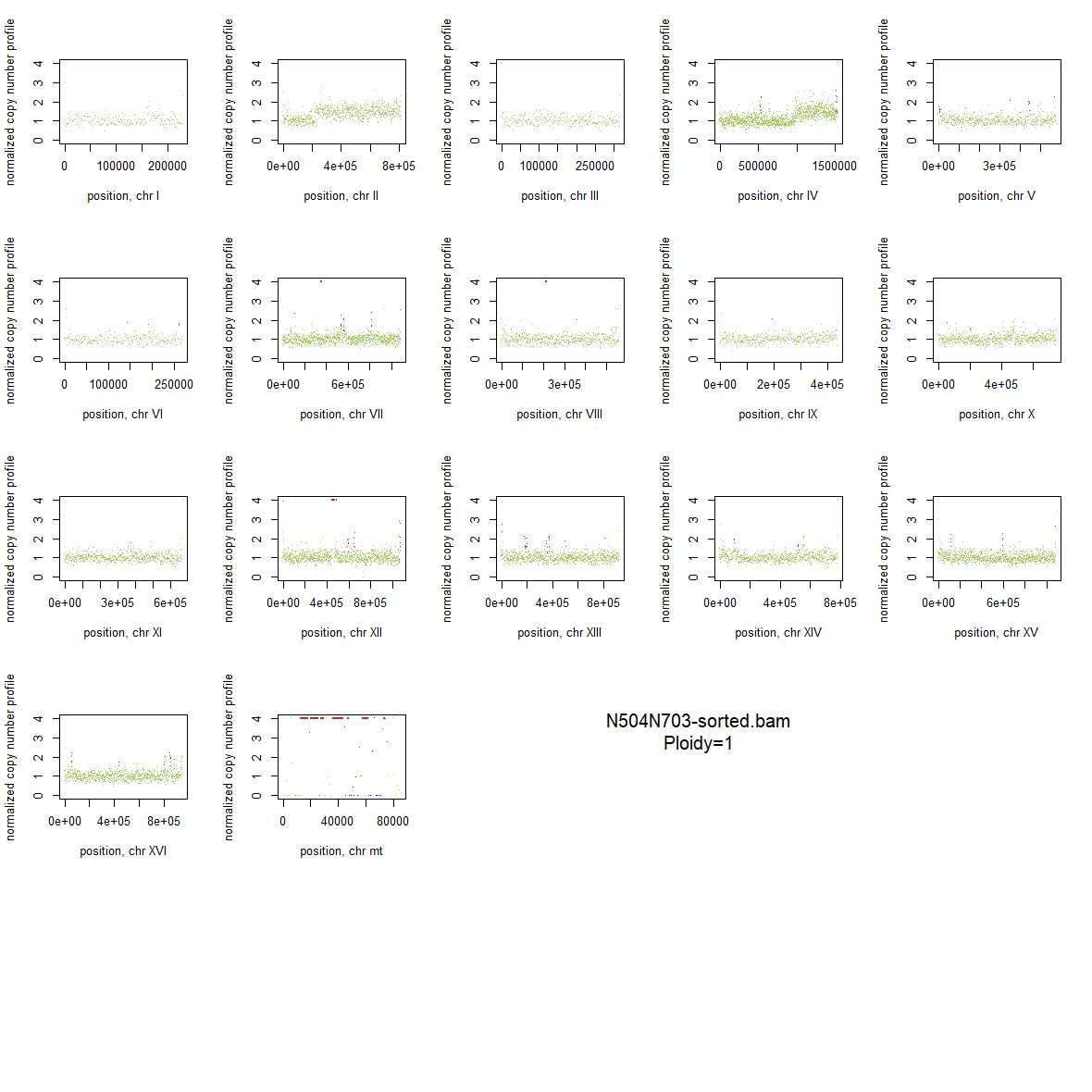

Supplement: Figure 2—source data 2. [file elife-79346-fig2-data2.zip › Figure2-source data 1/pACT1-sec53-V238M/2x_V238M_25.png]

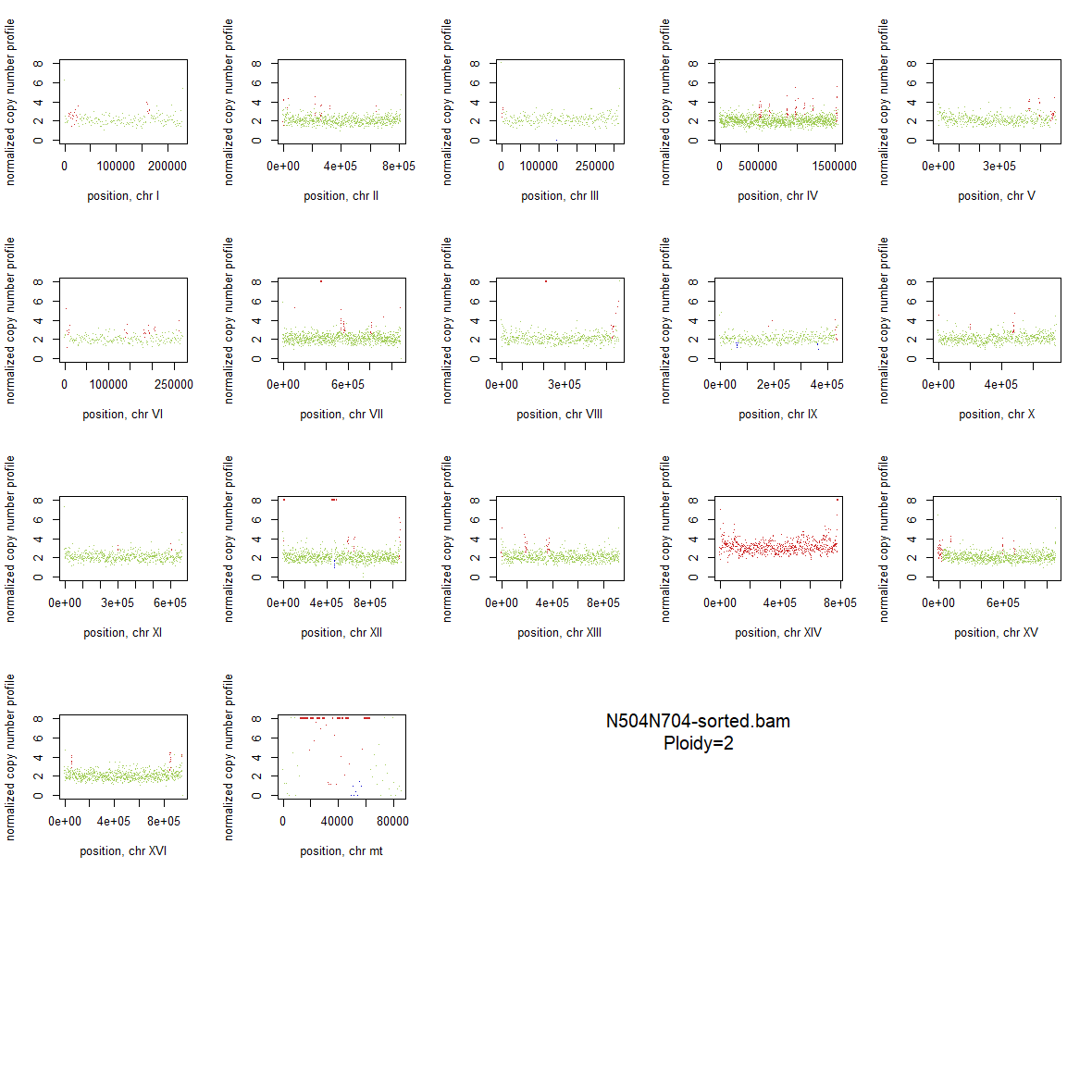

Supplement: Figure 2—source data 2. [file elife-79346-fig2-data2.zip › Figure2-source data 1/pACT1-sec53-V238M/2x_V238M_26.png]

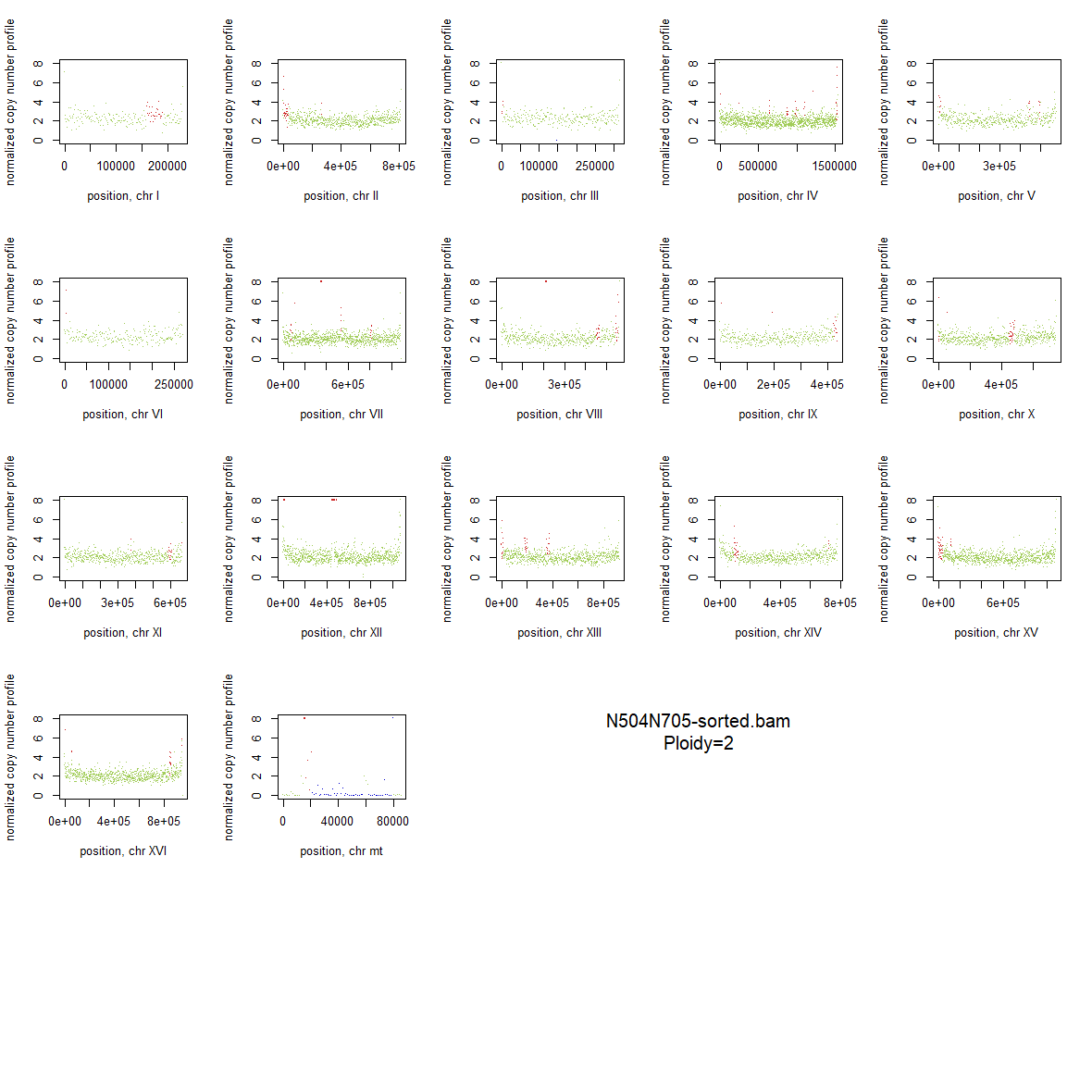

Supplement: Figure 2—source data 2. [file elife-79346-fig2-data2.zip › Figure2-source data 1/pACT1-sec53-V238M/2x_V238M_27.png]

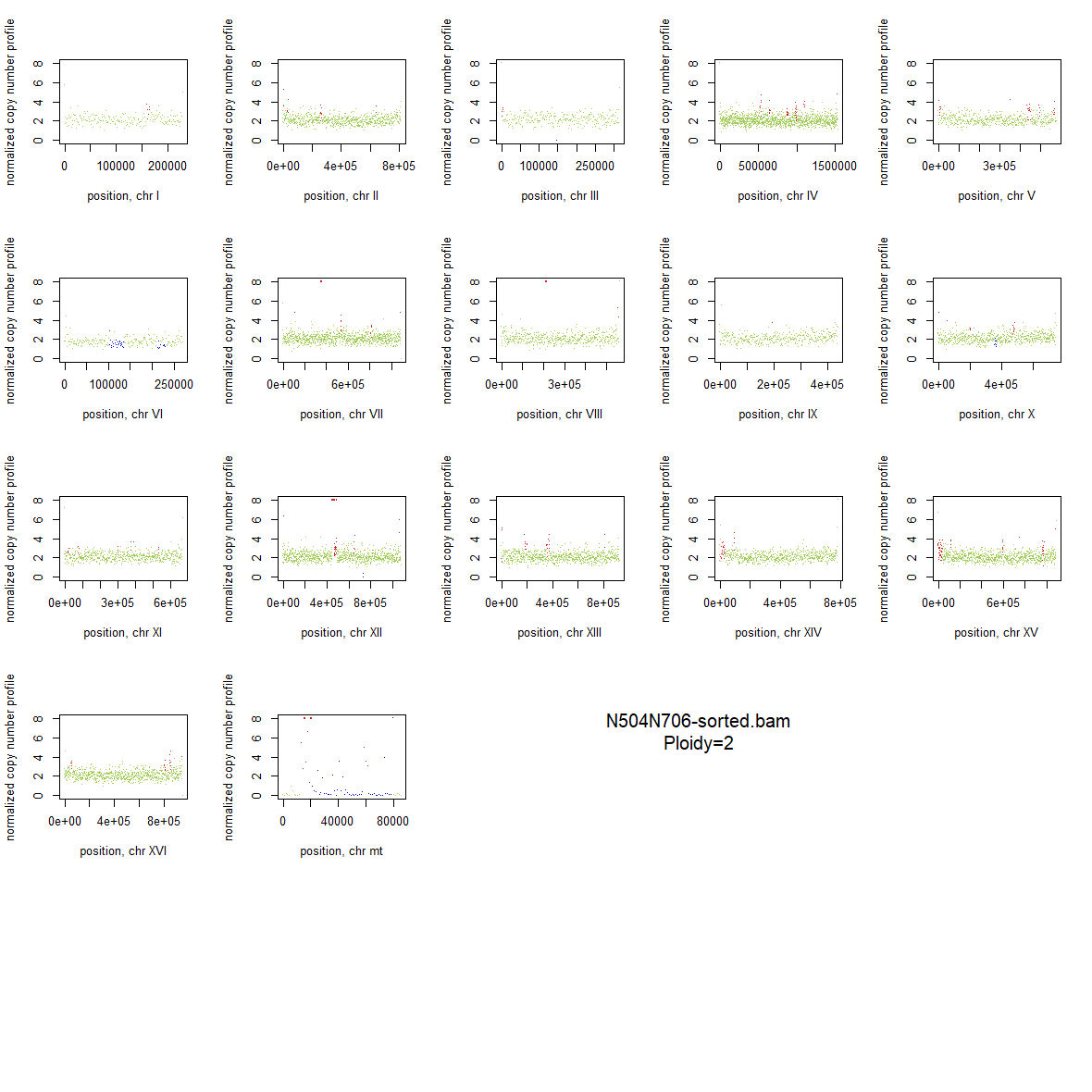

Supplement: Figure 2—source data 2. [file elife-79346-fig2-data2.zip › Figure2-source data 1/pACT1-sec53-V238M/2x_V238M_28.png]

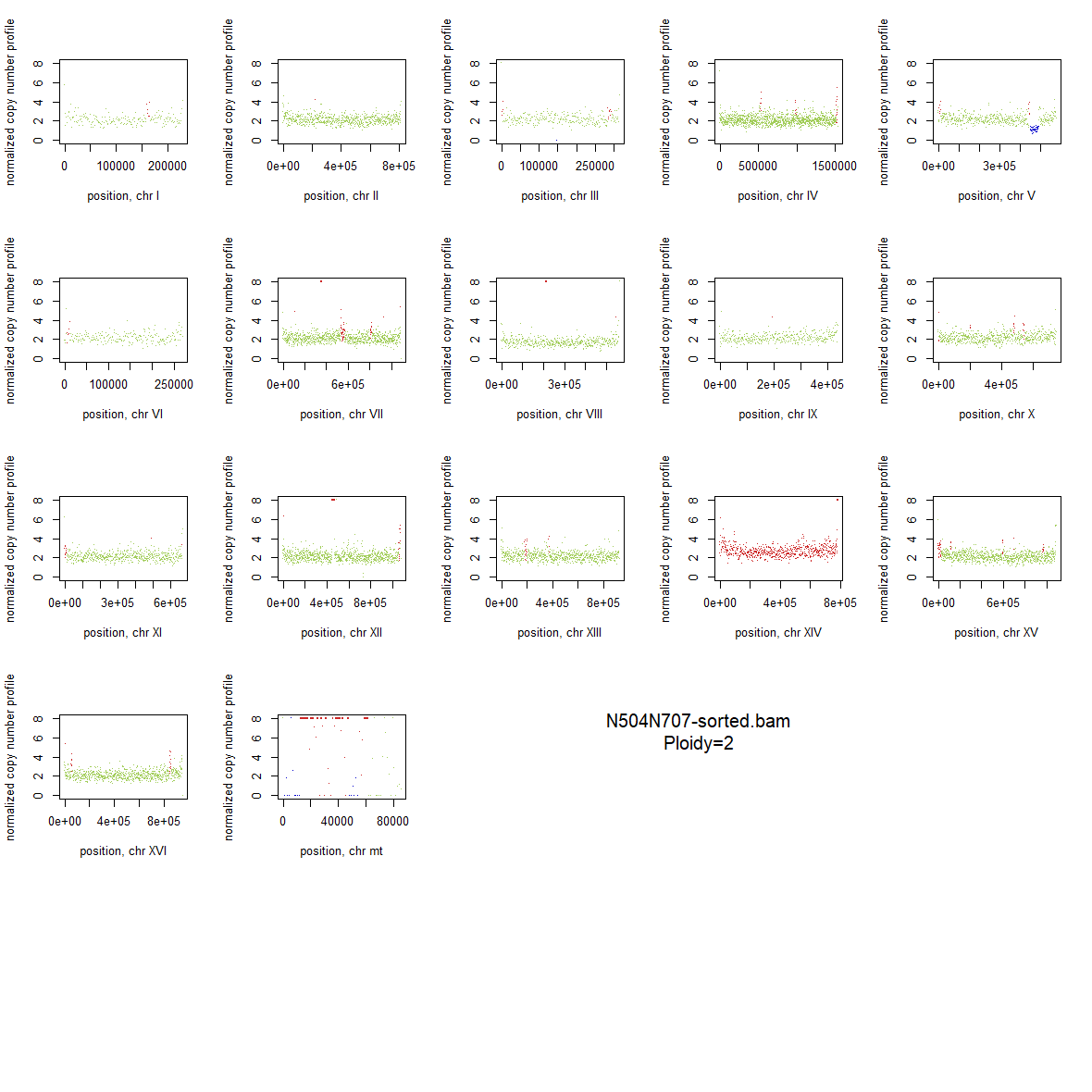

Supplement: Figure 2—source data 2. [file elife-79346-fig2-data2.zip › Figure2-source data 1/pACT1-sec53-V238M/2x_V238M_29.png]

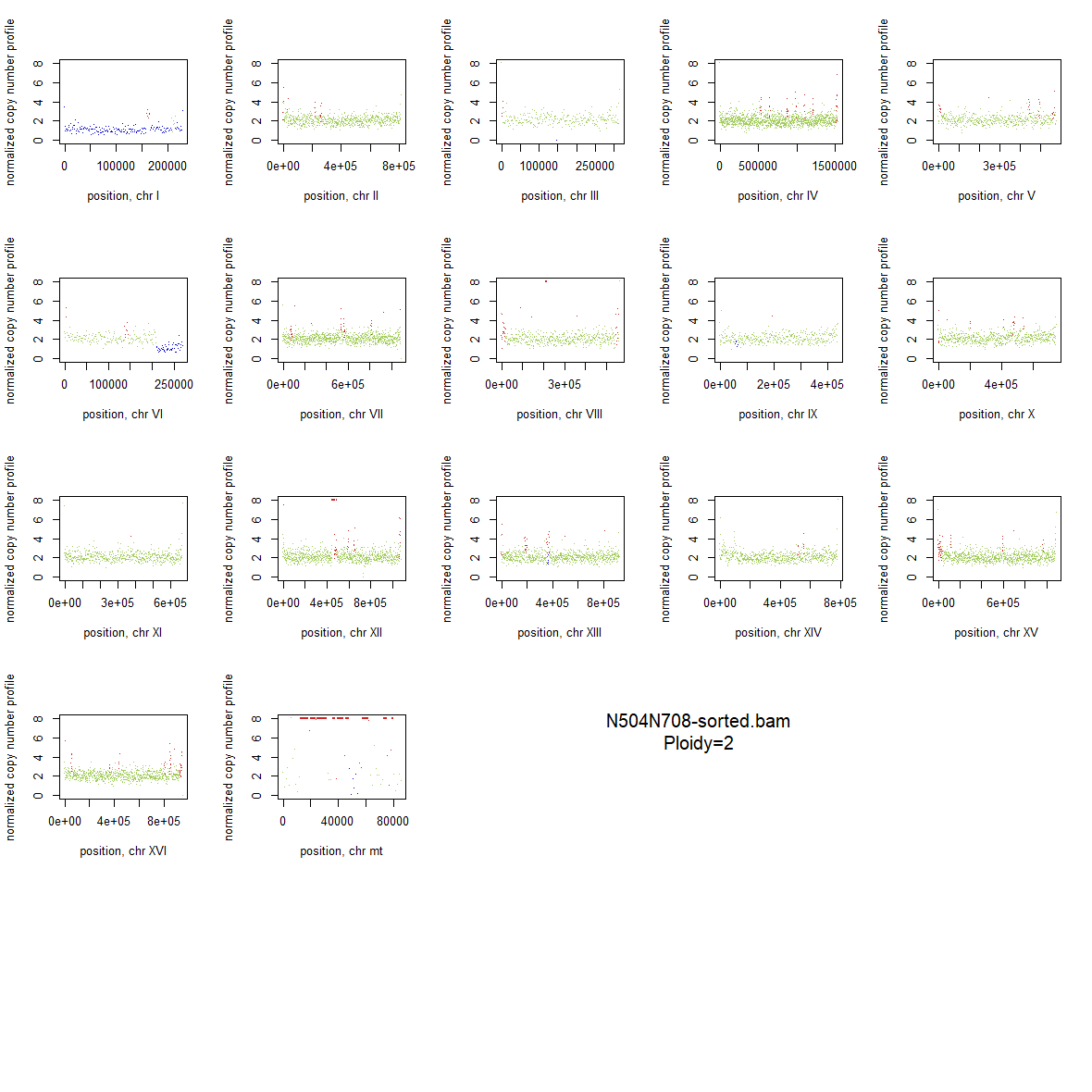

Supplement: Figure 2—source data 2. [file elife-79346-fig2-data2.zip › Figure2-source data 1/pACT1-sec53-V238M/2x_V238M_30.png]

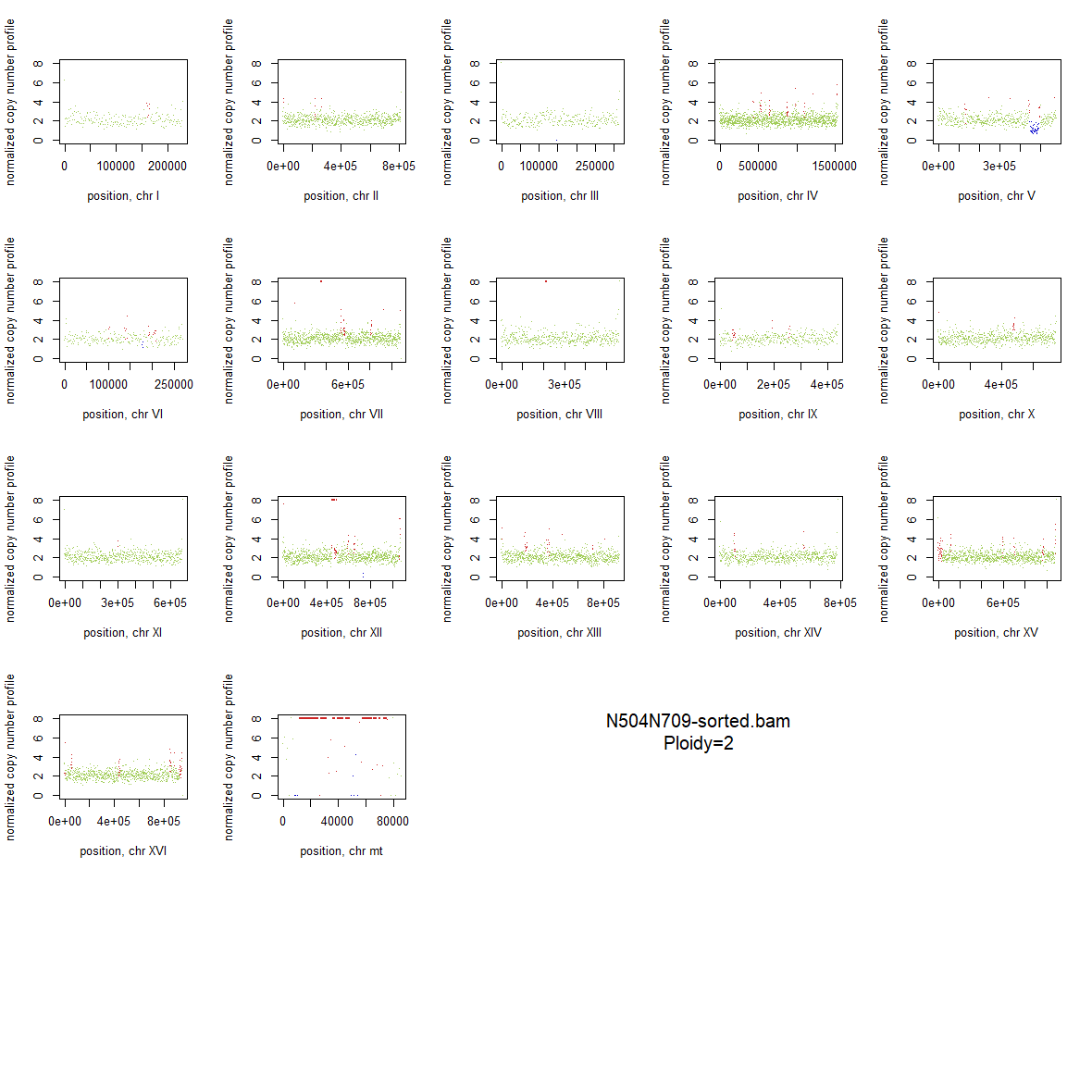

Supplement: Figure 2—source data 2. [file elife-79346-fig2-data2.zip › Figure2-source data 1/pACT1-sec53-V238M/2x_V238M_31.png]

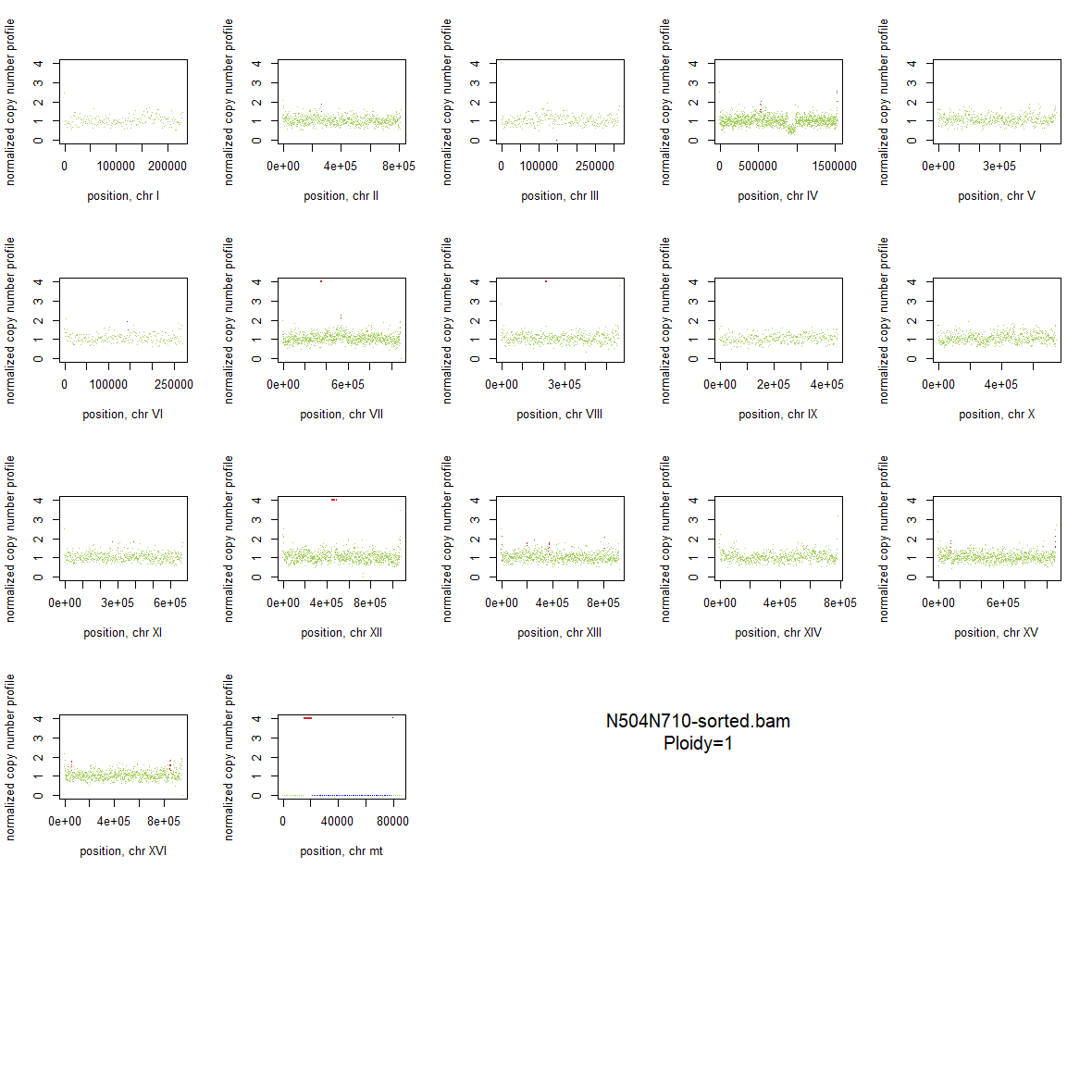

Supplement: Figure 2—source data 2. [file elife-79346-fig2-data2.zip › Figure2-source data 1/pACT1-sec53-V238M/2x_V238M_32.png]

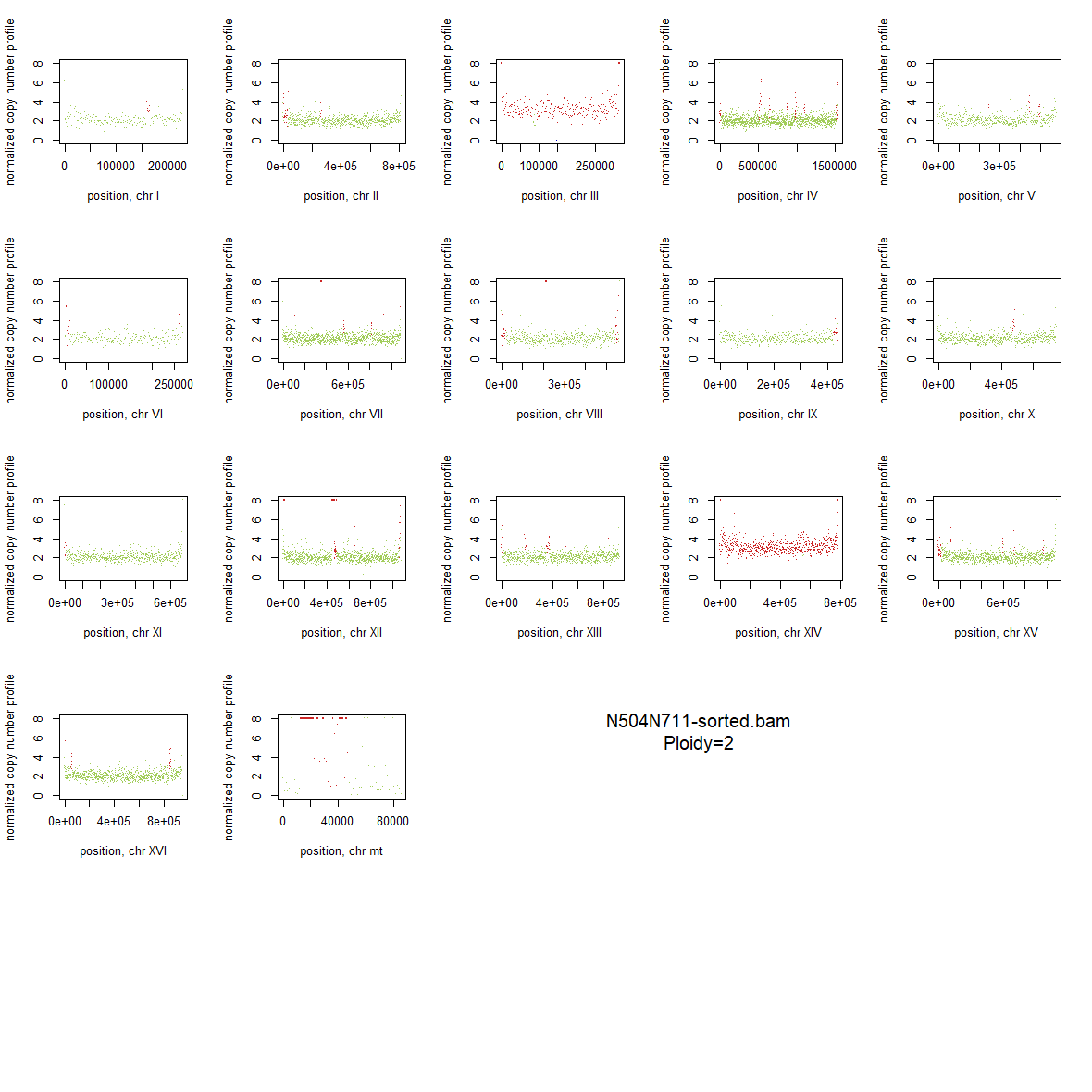

Supplement: Figure 2—source data 2. [file elife-79346-fig2-data2.zip › Figure2-source data 1/pACT1-sec53-V238M/2x_V238M_33.png]

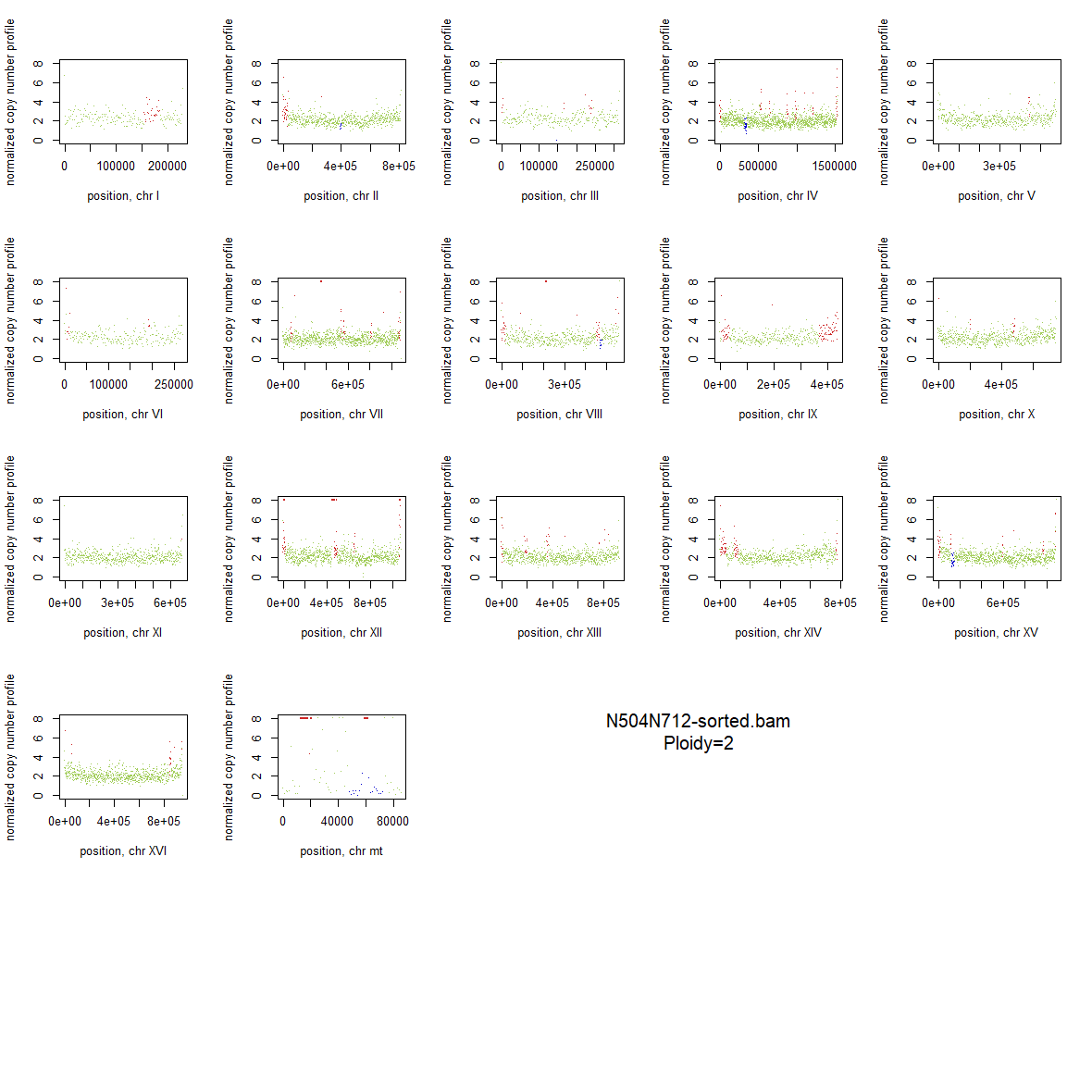

Supplement: Figure 2—source data 2. [file elife-79346-fig2-data2.zip › Figure2-source data 1/pACT1-sec53-V238M/2x_V238M_34.png]

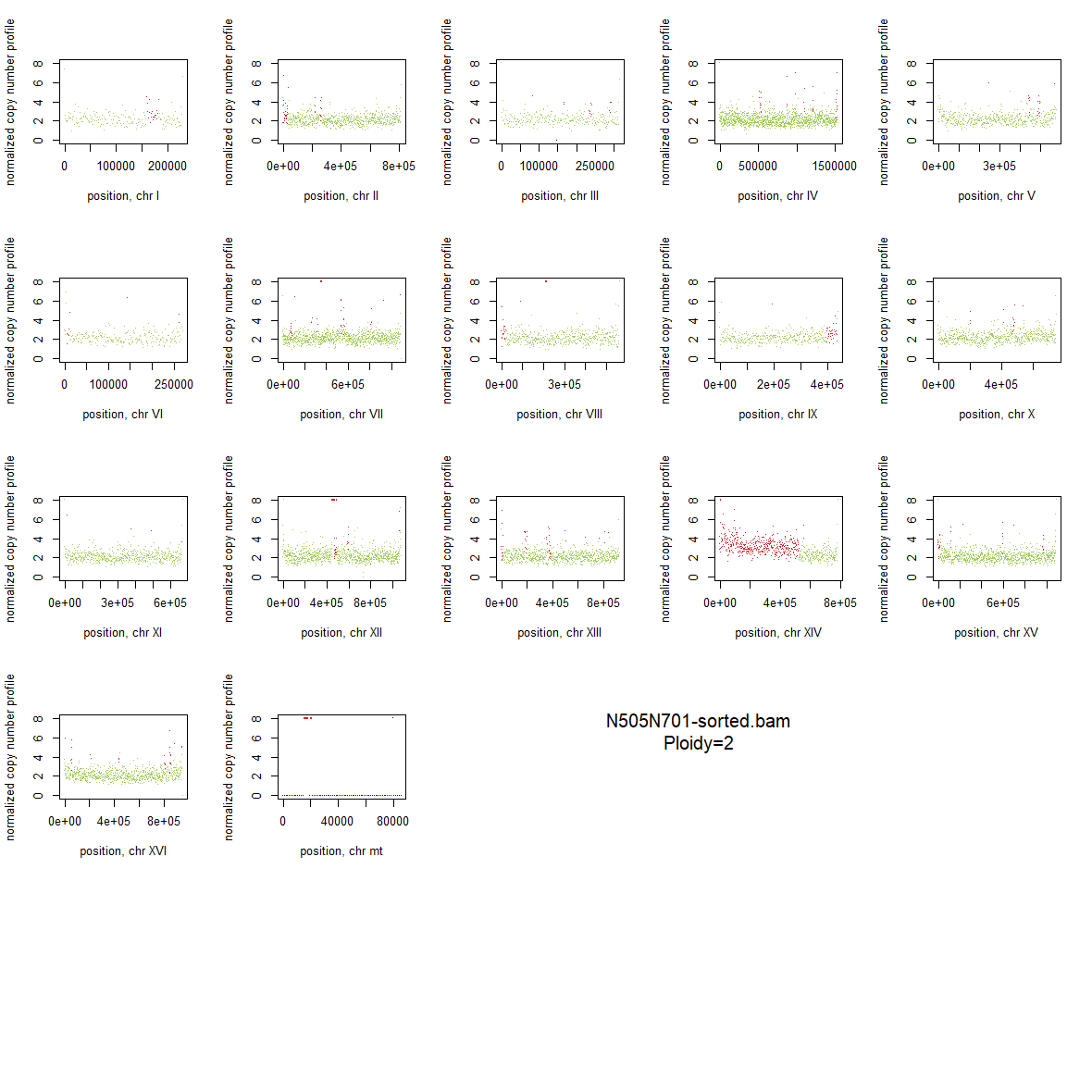

Supplement: Figure 2—source data 2. [file elife-79346-fig2-data2.zip › Figure2-source data 1/pACT1-sec53-V238M/2x_V238M_35.png]

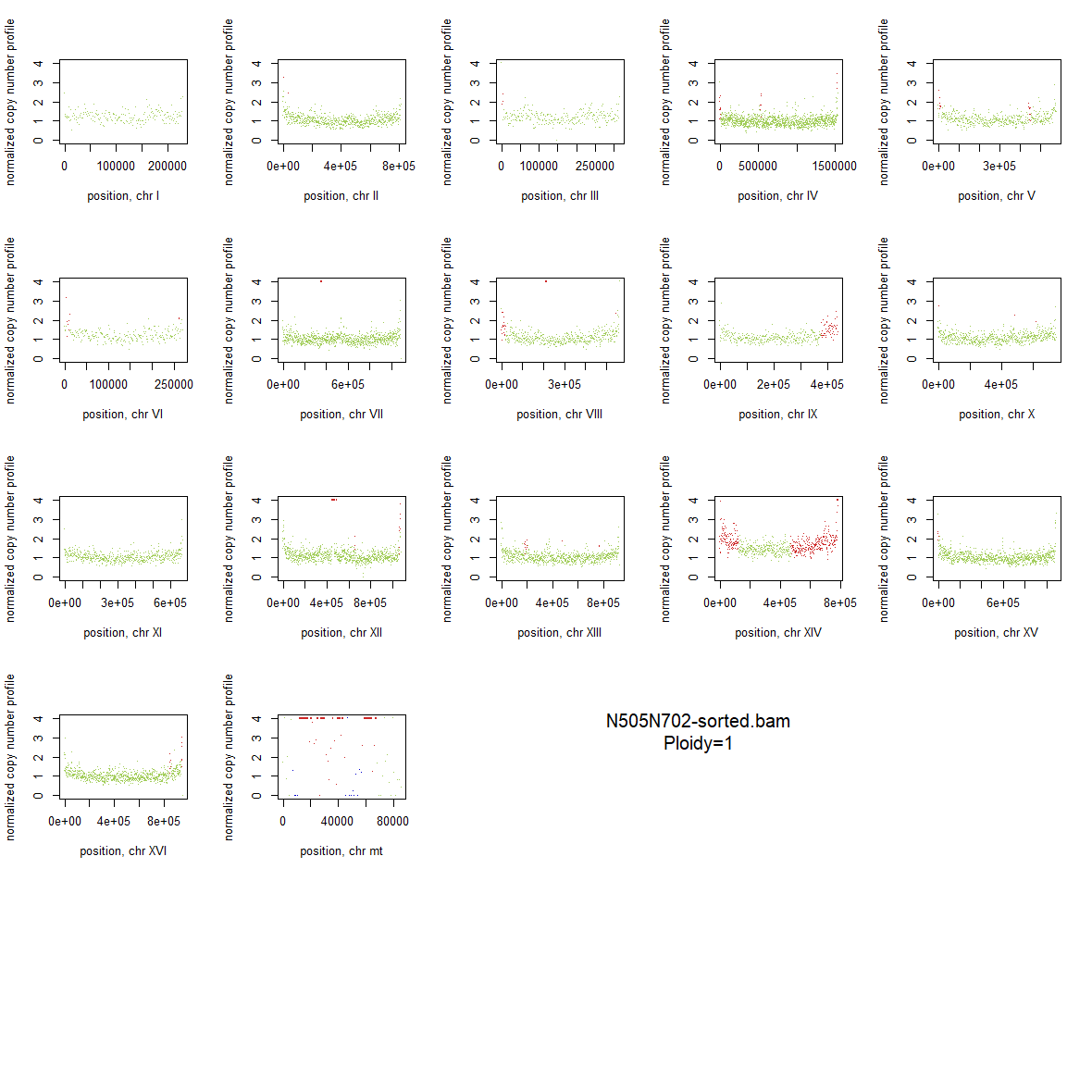

Supplement: Figure 2—source data 2. [file elife-79346-fig2-data2.zip › Figure2-source data 1/pACT1-sec53-V238M/2x_V238M_36.png]

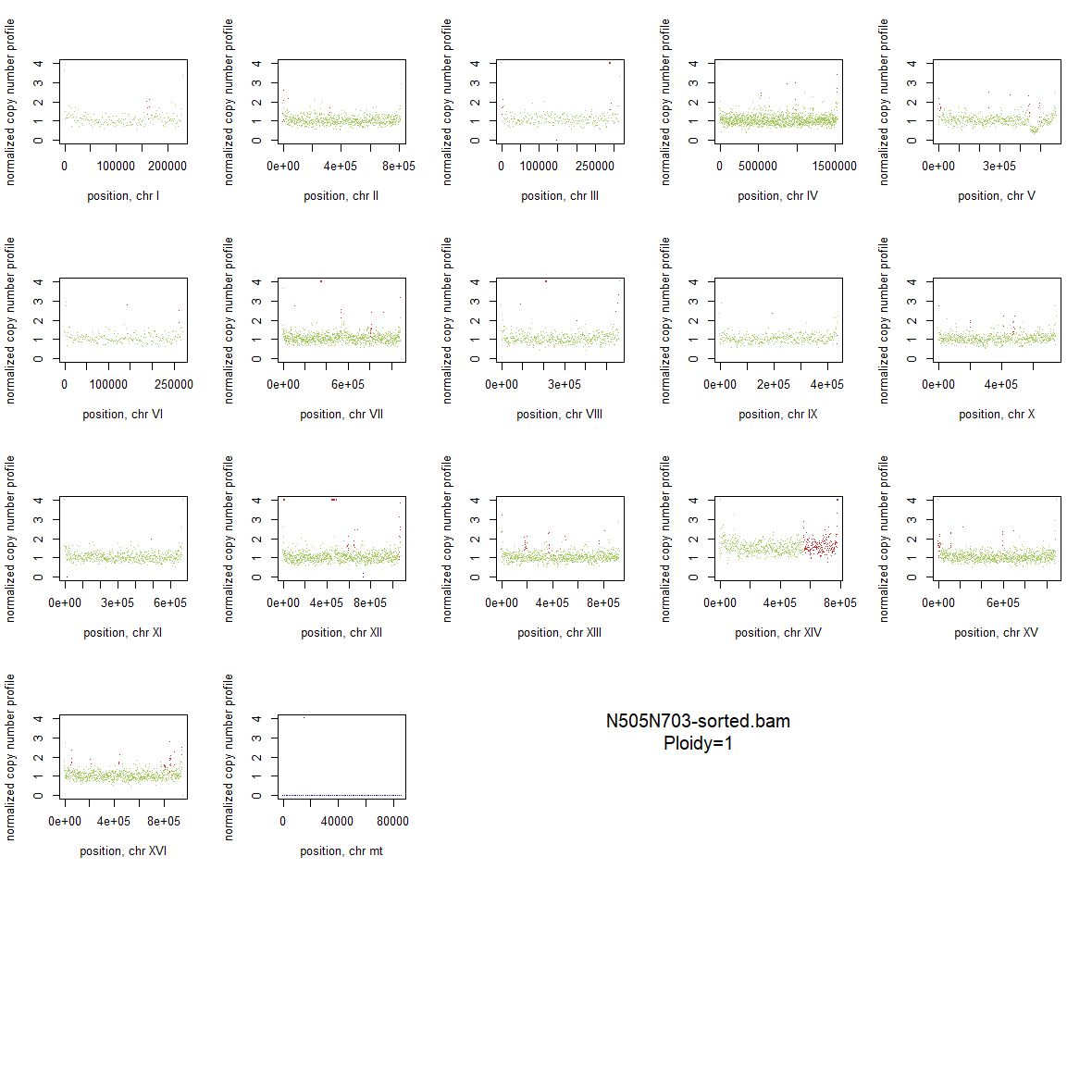

Supplement: Figure 2—source data 2. [file elife-79346-fig2-data2.zip › Figure2-source data 1/pACT1-sec53-V238M/2x_V238M_37.png]

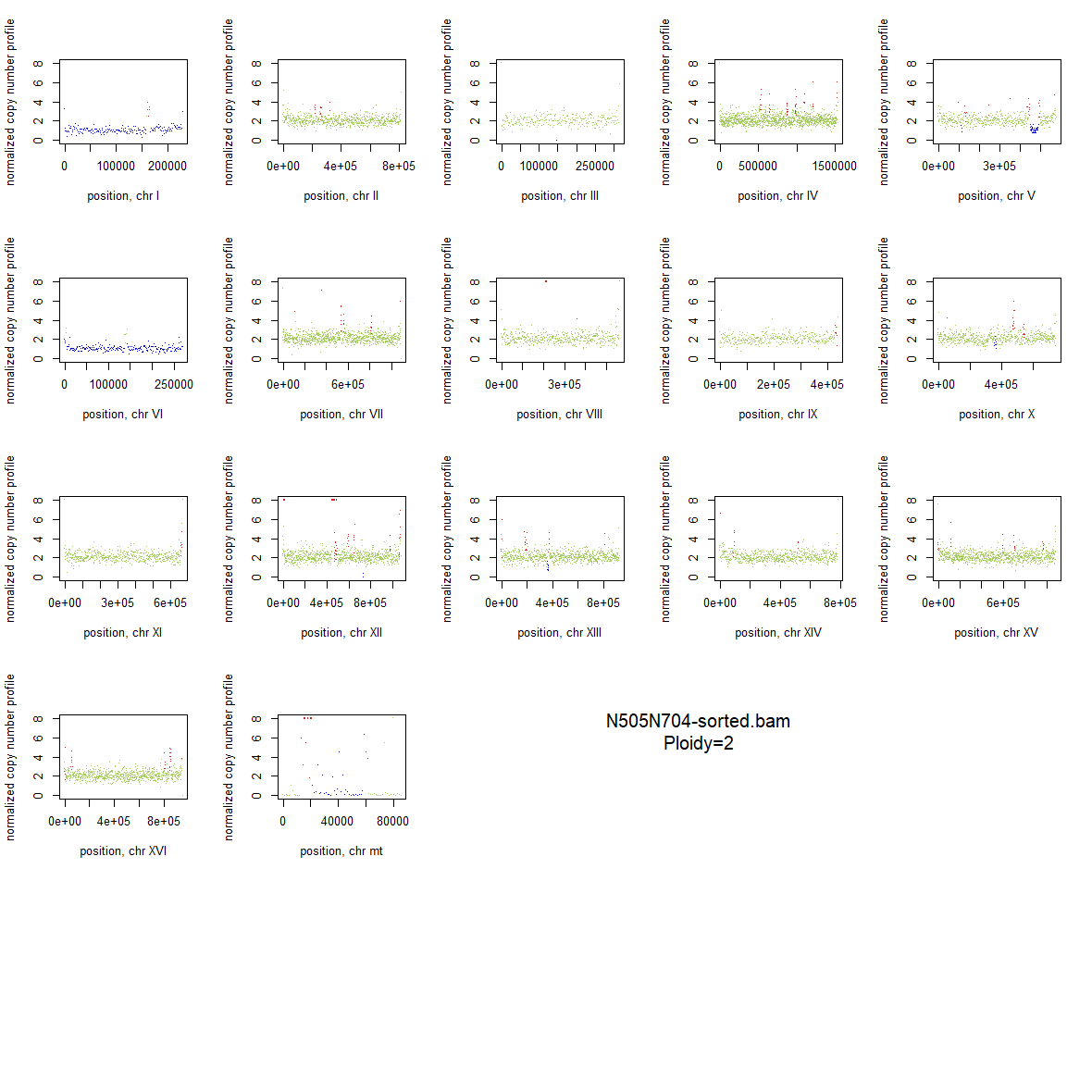

Supplement: Figure 2—source data 2. [file elife-79346-fig2-data2.zip › Figure2-source data 1/pACT1-sec53-V238M/2x_V238M_38.png]

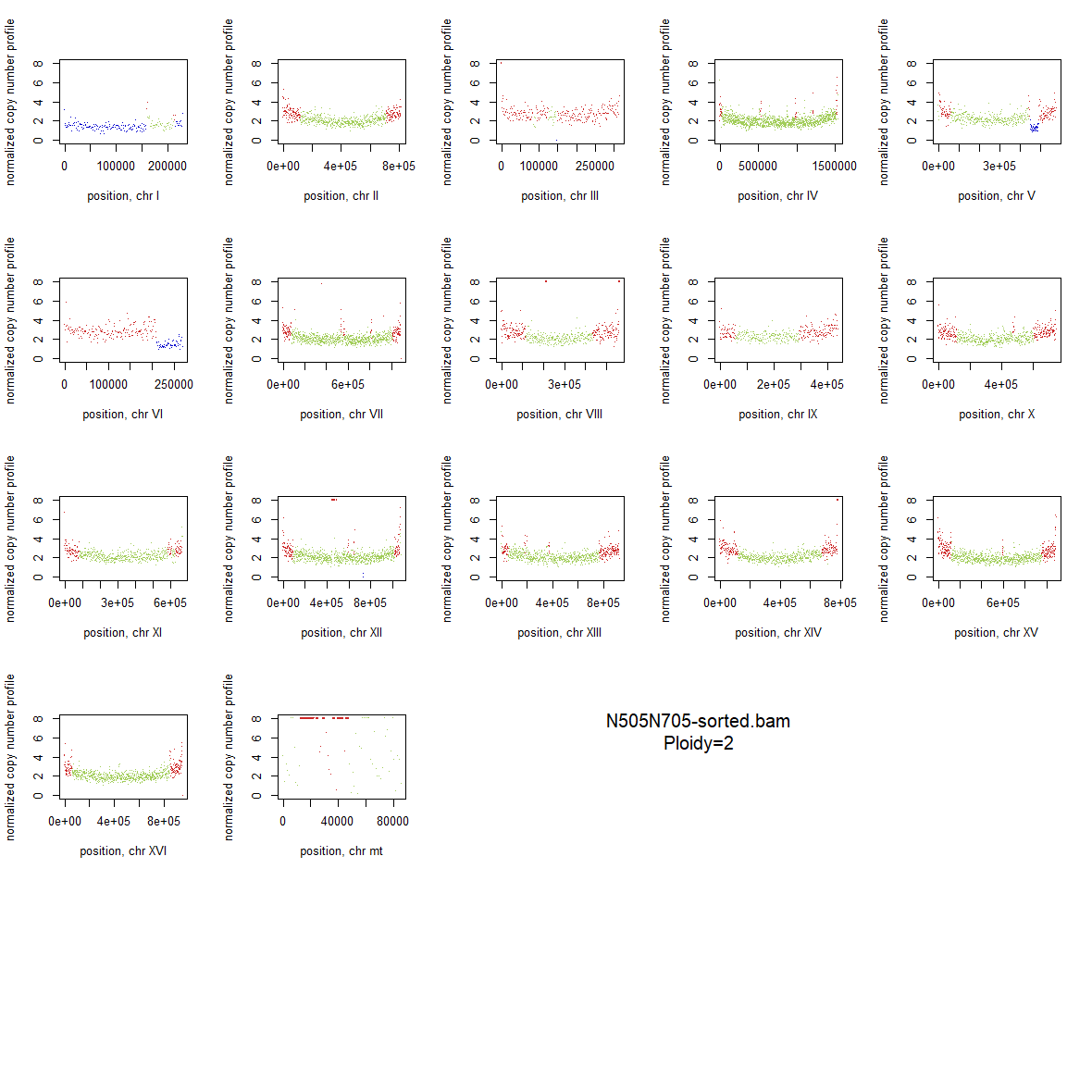

Supplement: Figure 2—source data 2. [file elife-79346-fig2-data2.zip › Figure2-source data 1/pACT1-sec53-V238M/2x_V238M_39.png]

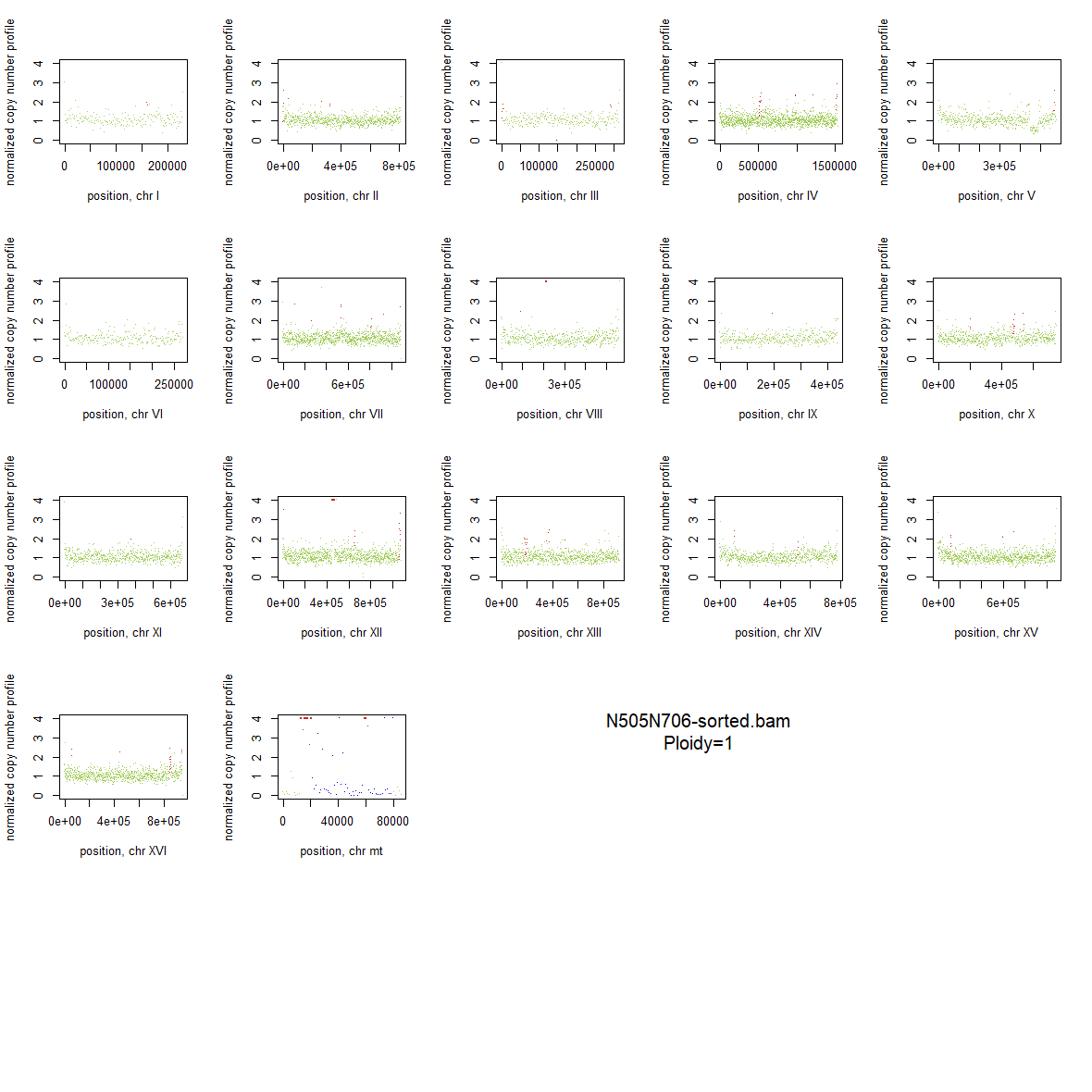

Supplement: Figure 2—source data 2. [file elife-79346-fig2-data2.zip › Figure2-source data 1/pACT1-sec53-V238M/2x_V238M_40.png]

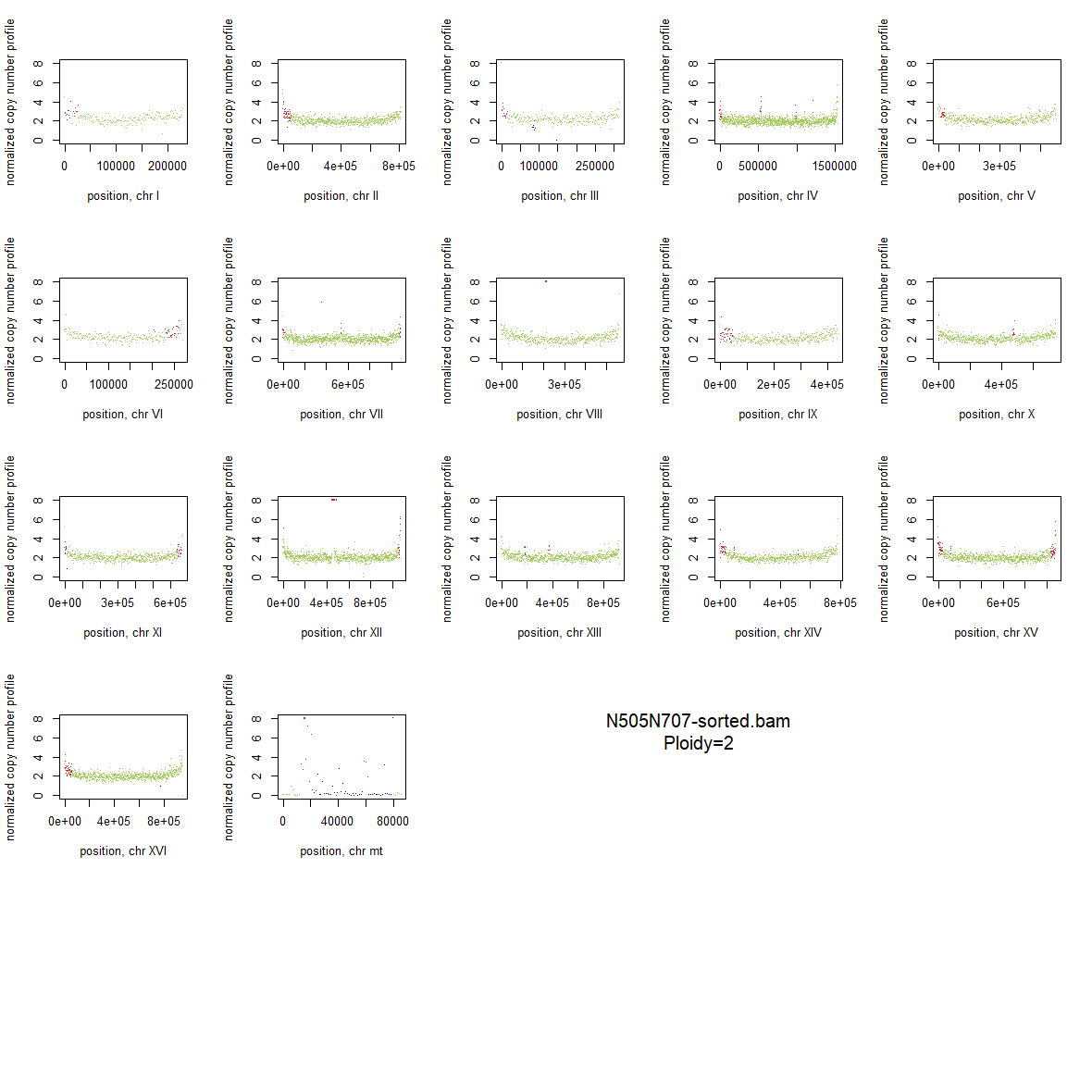

Supplement: Figure 2—source data 2. [file elife-79346-fig2-data2.zip › Figure2-source data 1/pACT1-sec53-V238M/2x_V238M_41.png]

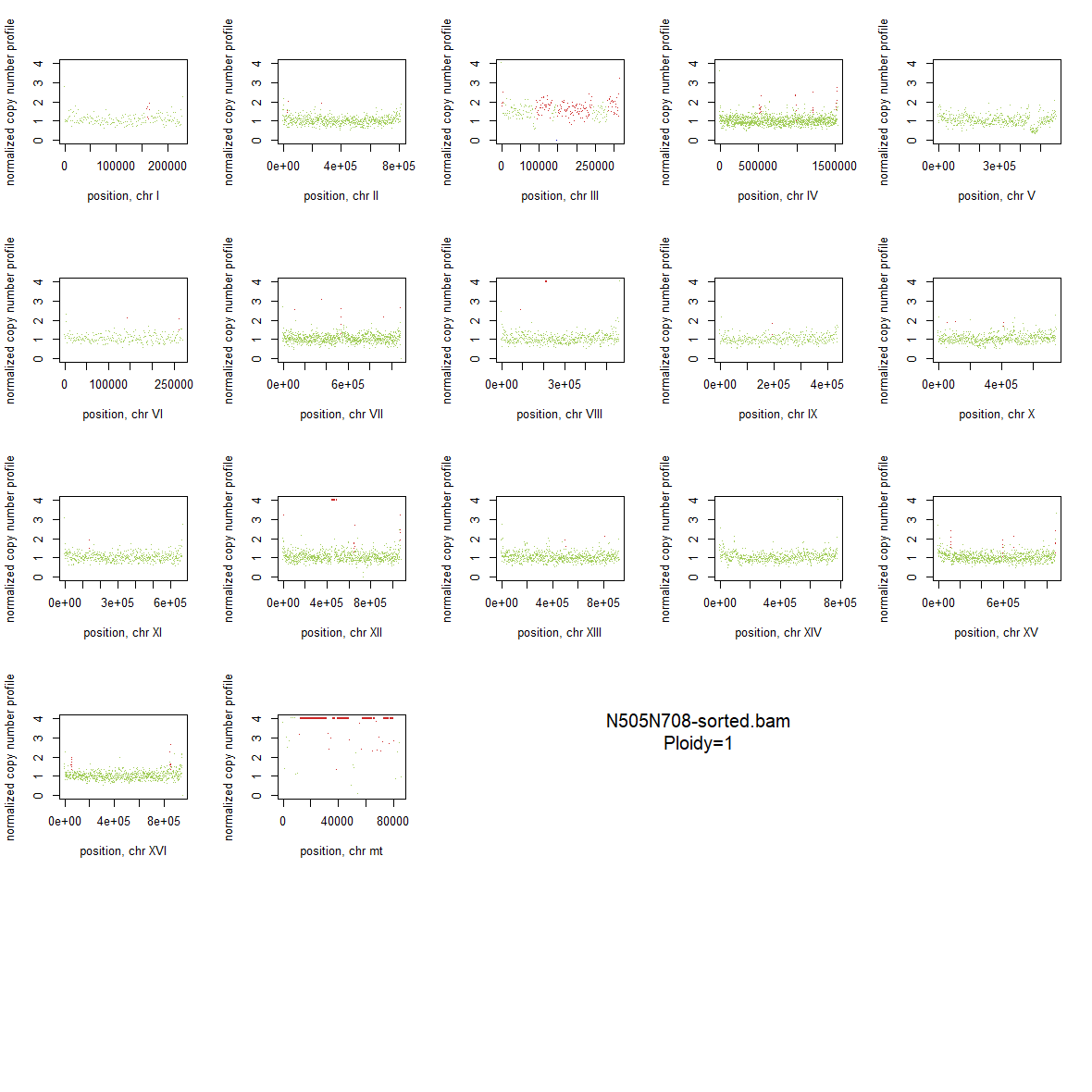

Supplement: Figure 2—source data 2. [file elife-79346-fig2-data2.zip › Figure2-source data 1/pACT1-sec53-V238M/2x_V238M_42.png]

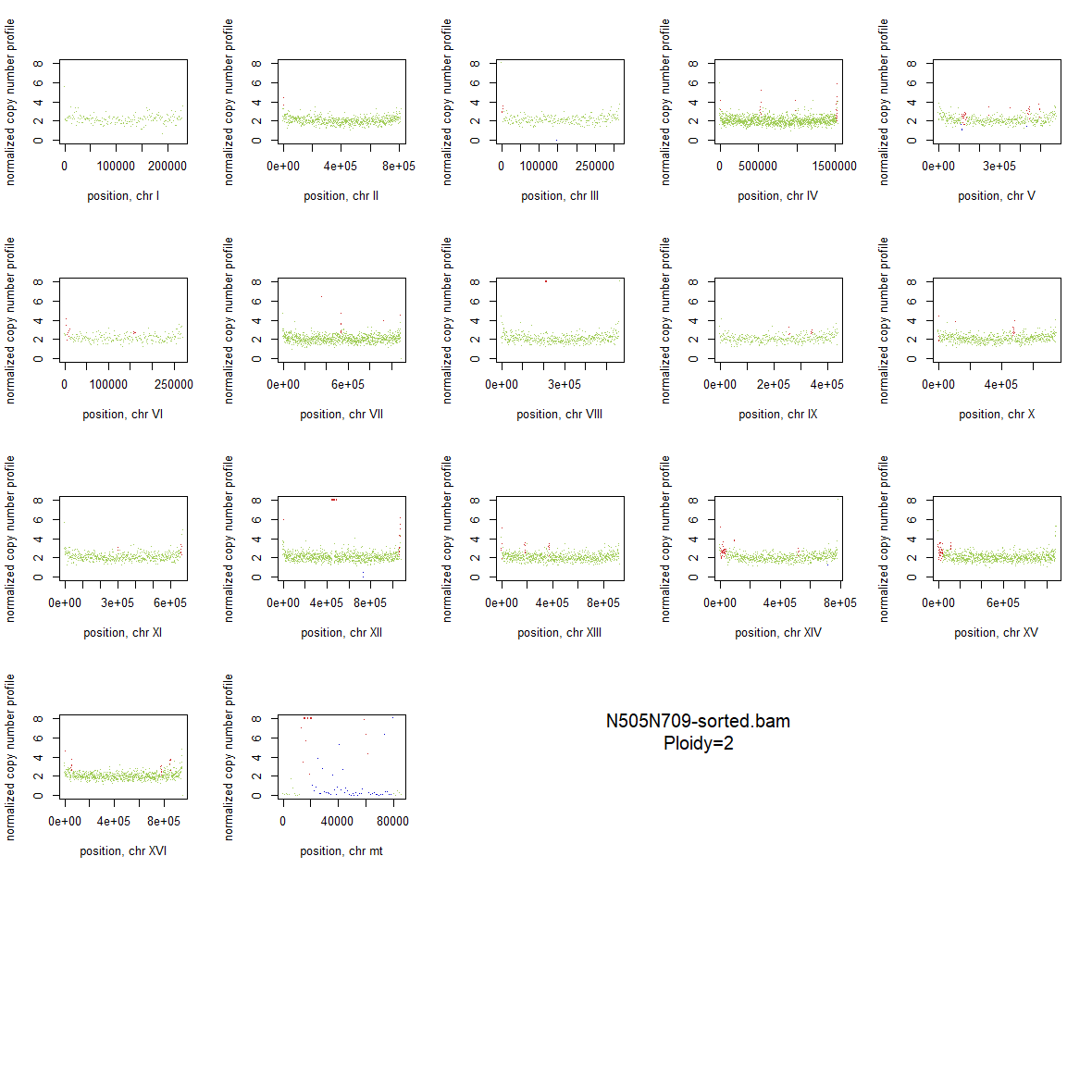

Supplement: Figure 2—source data 2. [file elife-79346-fig2-data2.zip › Figure2-source data 1/pACT1-sec53-V238M/2x_V238M_43.png]

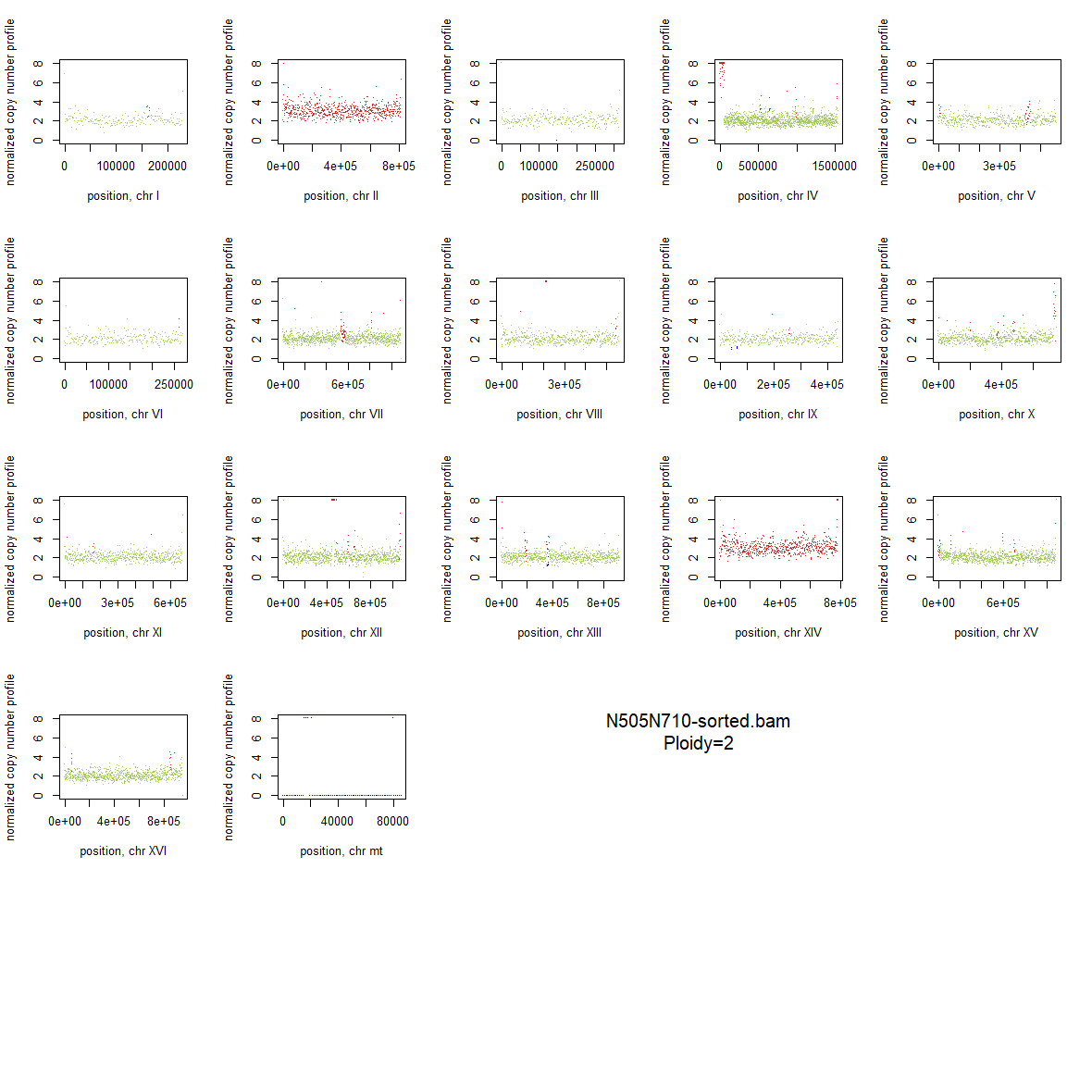

Supplement: Figure 2—source data 2. [file elife-79346-fig2-data2.zip › Figure2-source data 1/pACT1-sec53-V238M/2x_V238M_44.png]

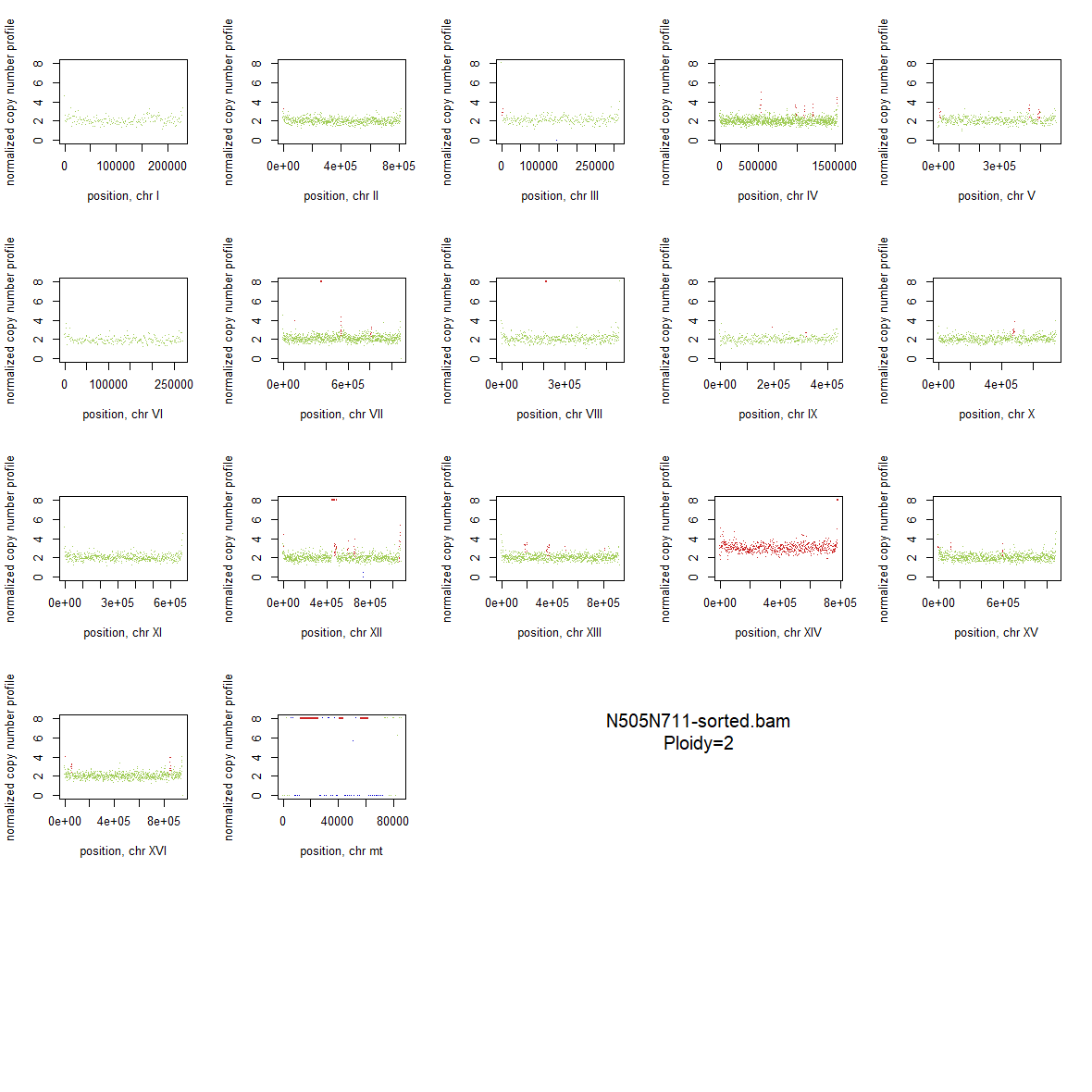

Supplement: Figure 2—source data 2. [file elife-79346-fig2-data2.zip › Figure2-source data 1/pACT1-sec53-V238M/2x_V238M_45.png]

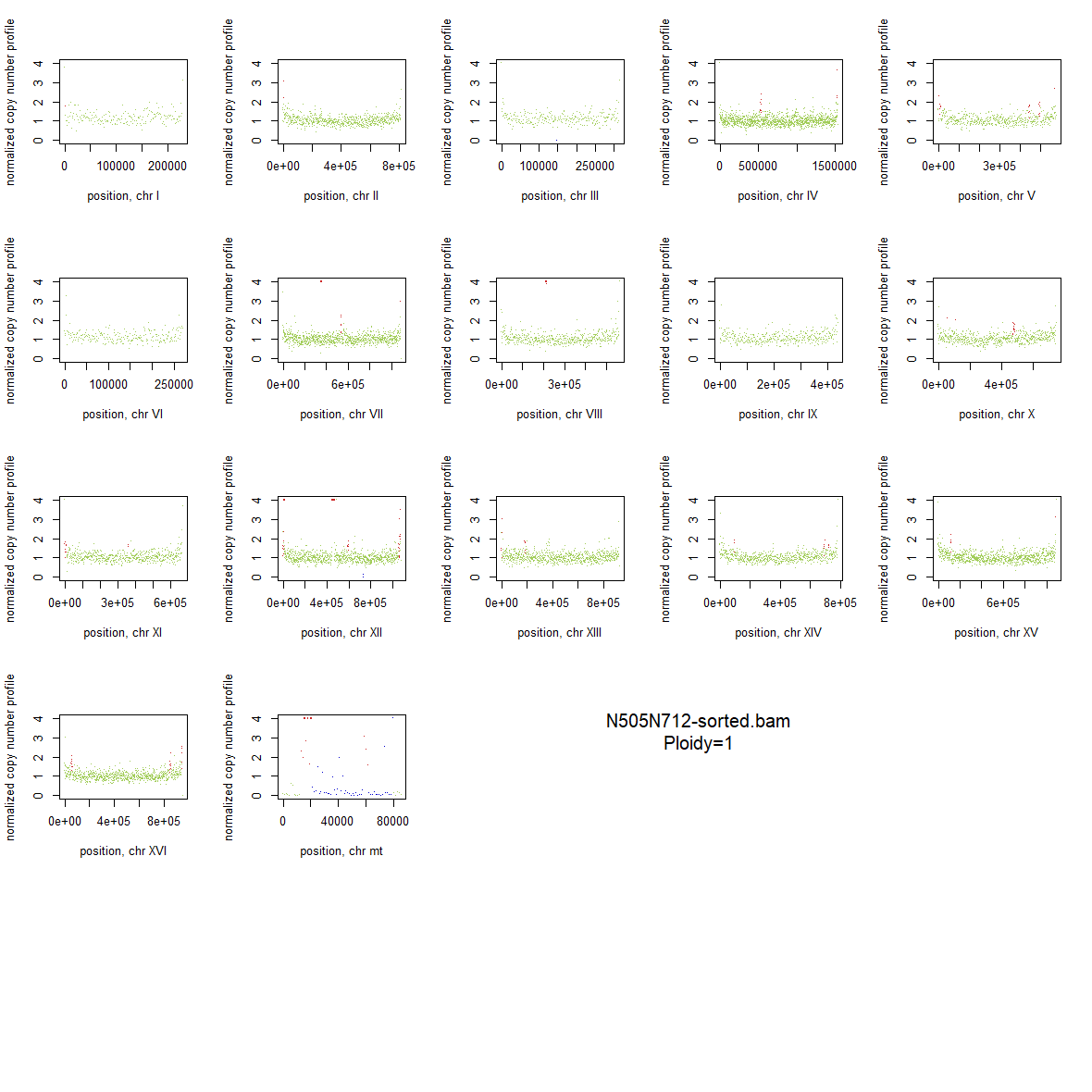

Supplement: Figure 2—source data 2. [file elife-79346-fig2-data2.zip › Figure2-source data 1/pACT1-sec53-V238M/2x_V238M_46.png]

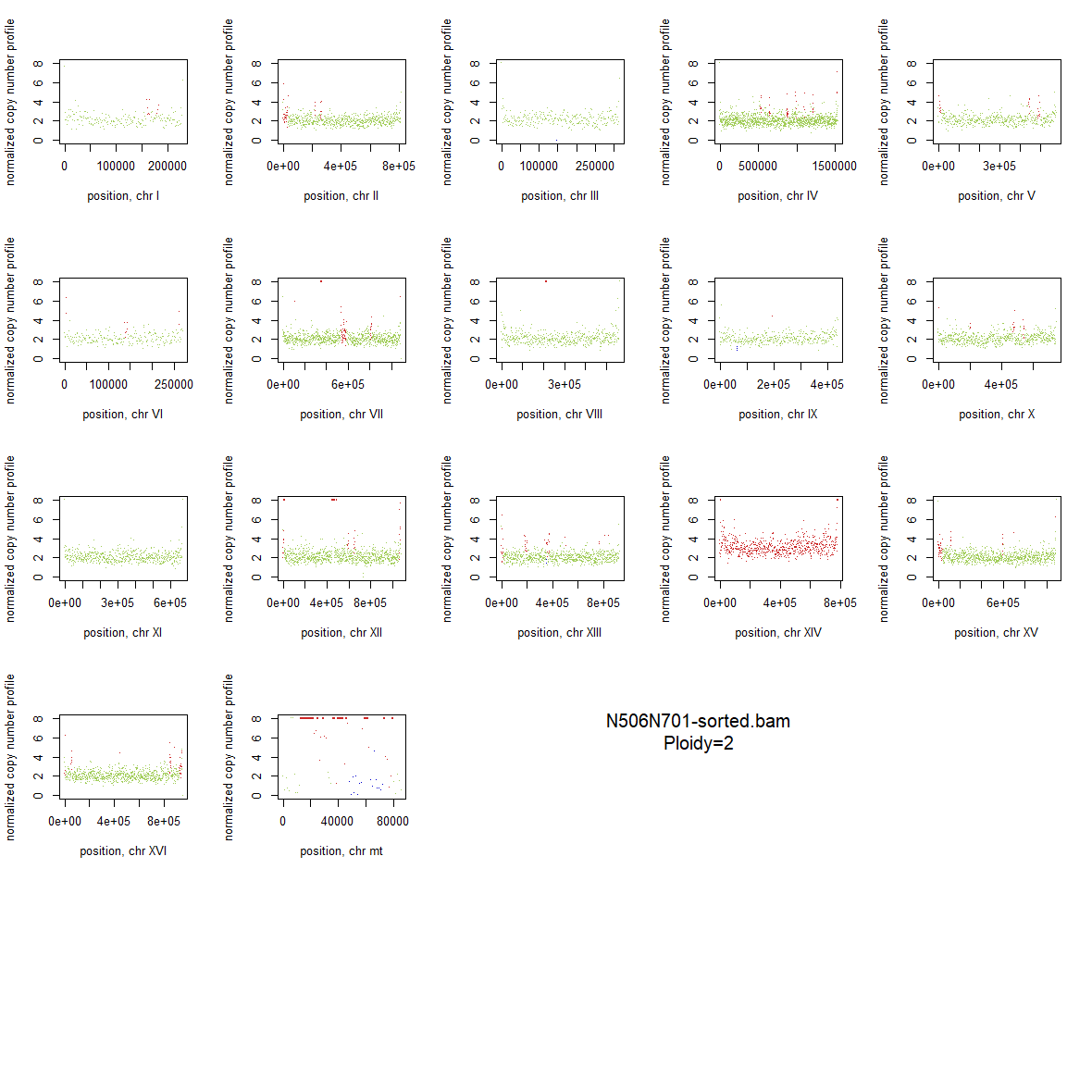

Supplement: Figure 2—source data 2. [file elife-79346-fig2-data2.zip › Figure2-source data 1/pACT1-sec53-V238M/2x_V238M_47.png]

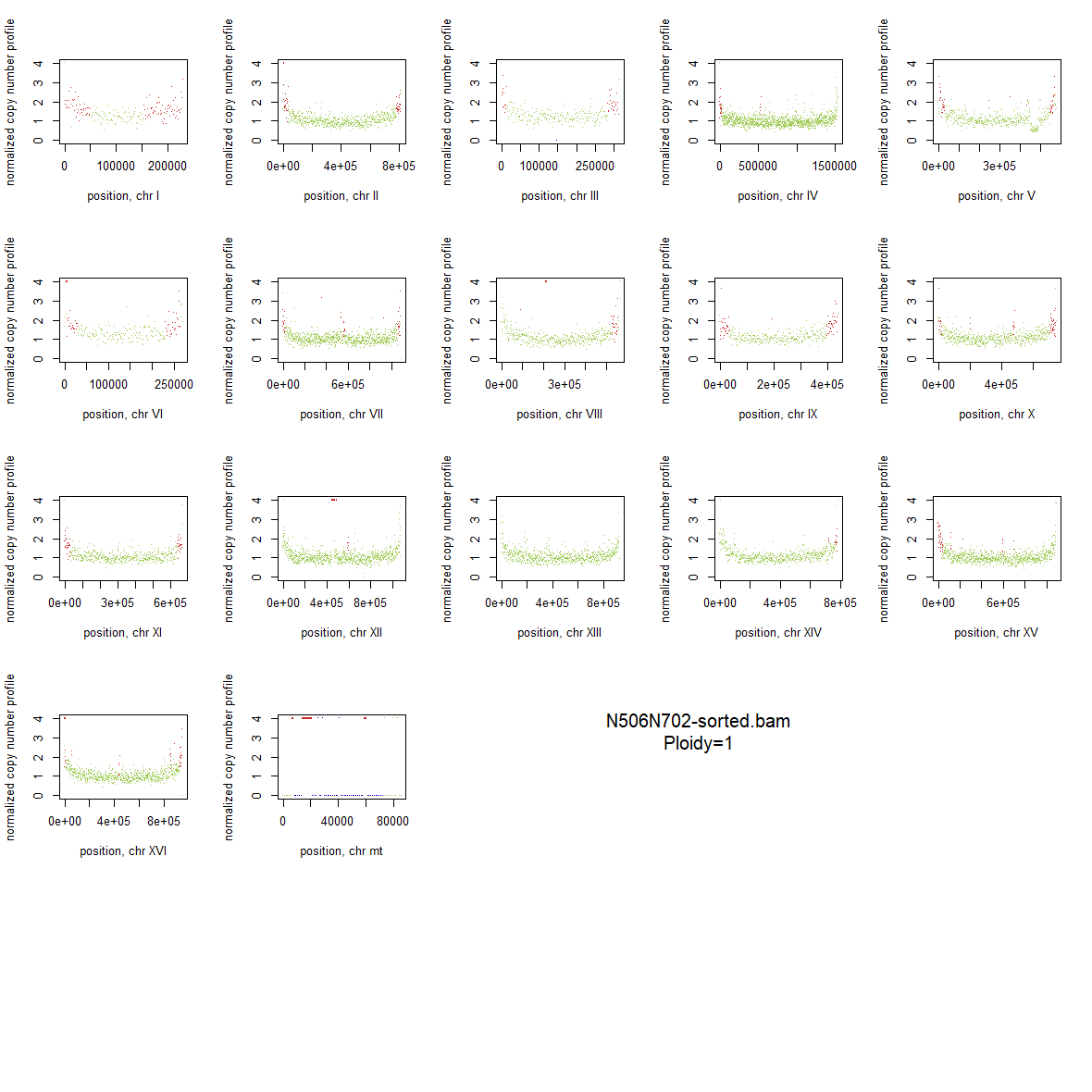

Supplement: Figure 2—source data 2. [file elife-79346-fig2-data2.zip › Figure2-source data 1/pACT1-sec53-V238M/2x_V238M_48.png]

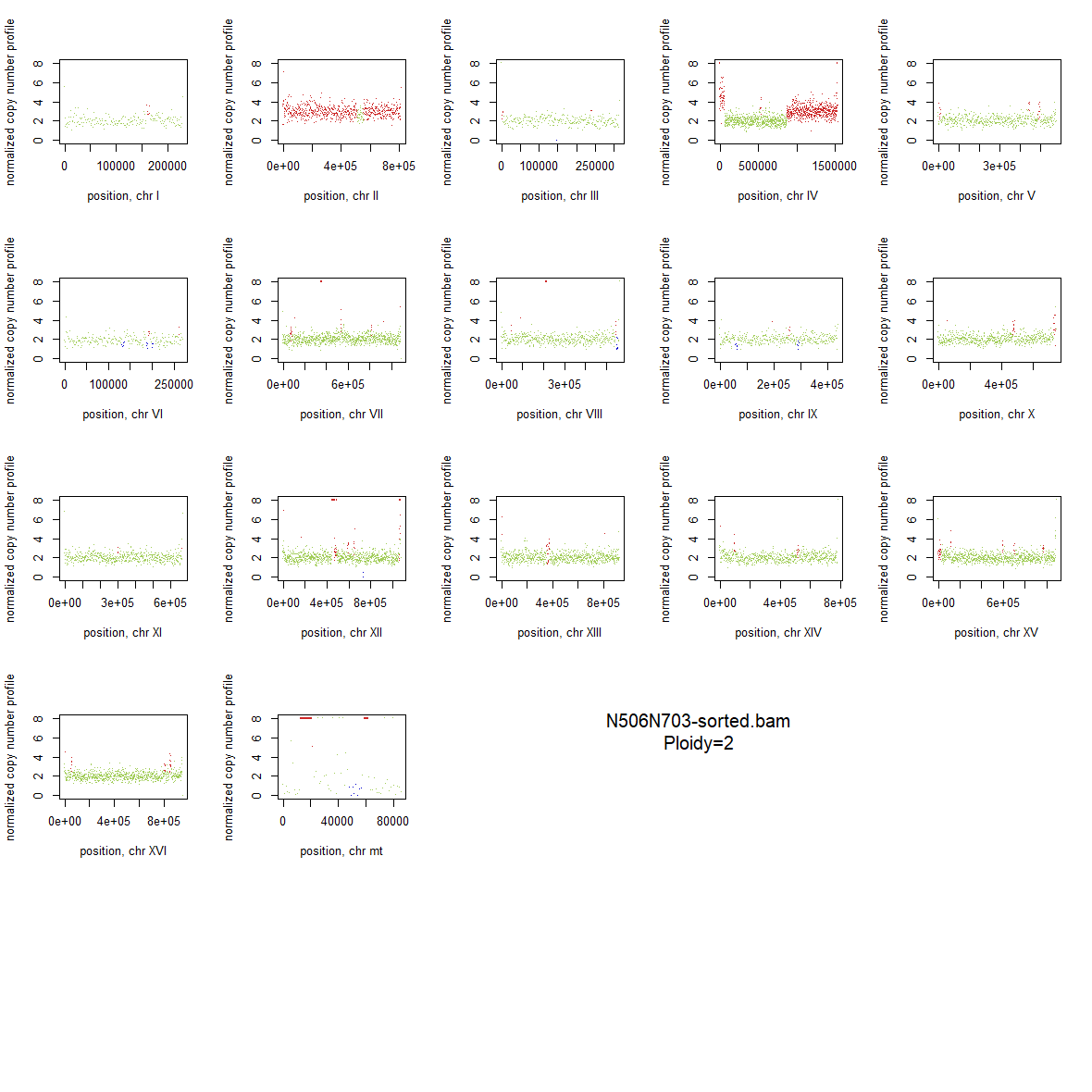

Supplement: Figure 2—source data 2. [file elife-79346-fig2-data2.zip › Figure2-source data 1/pACT1-sec53-V238M/2x_V238M_49.png]

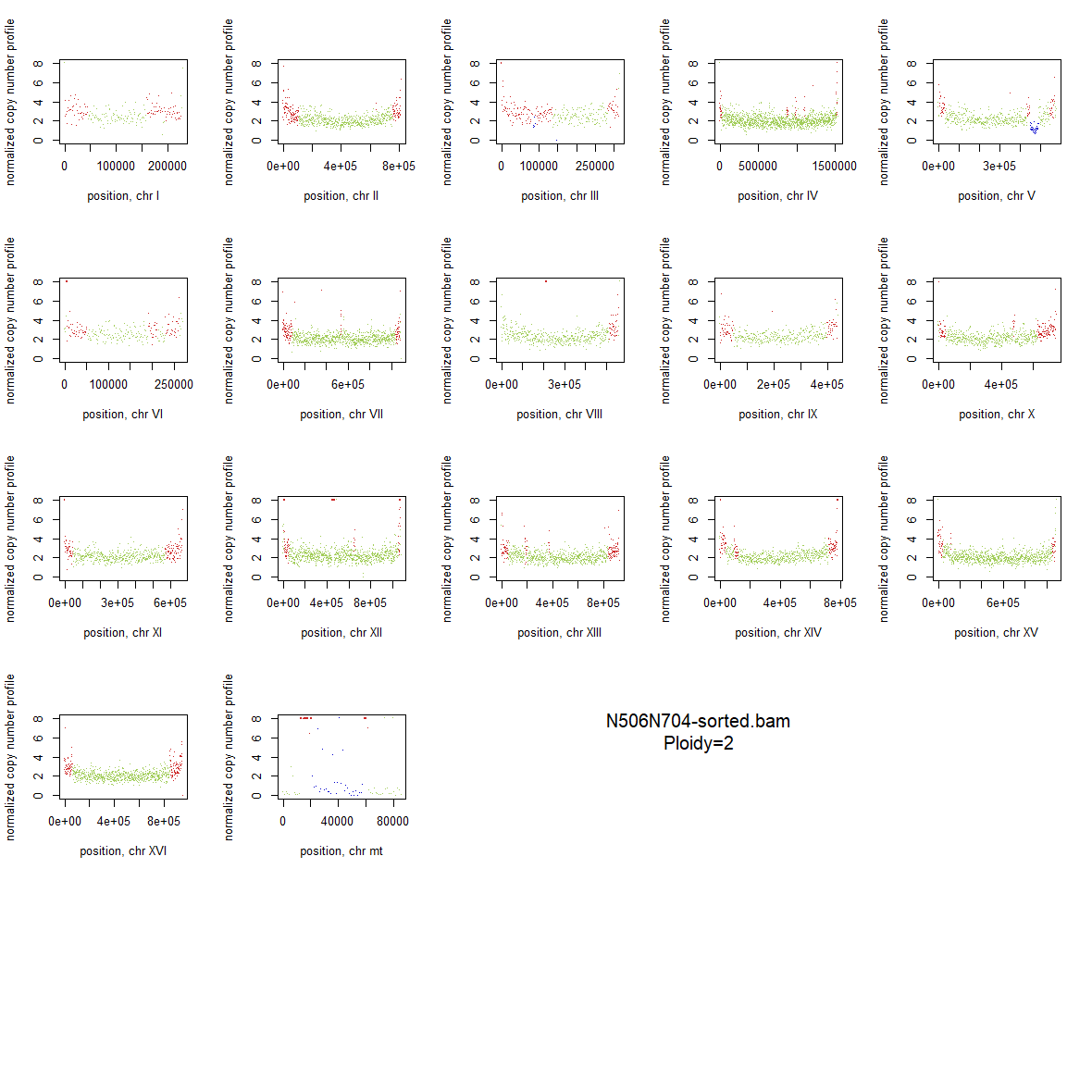

Supplement: Figure 2—source data 2. [file elife-79346-fig2-data2.zip › Figure2-source data 1/pACT1-sec53-V238M/2x_V238M_50.png]

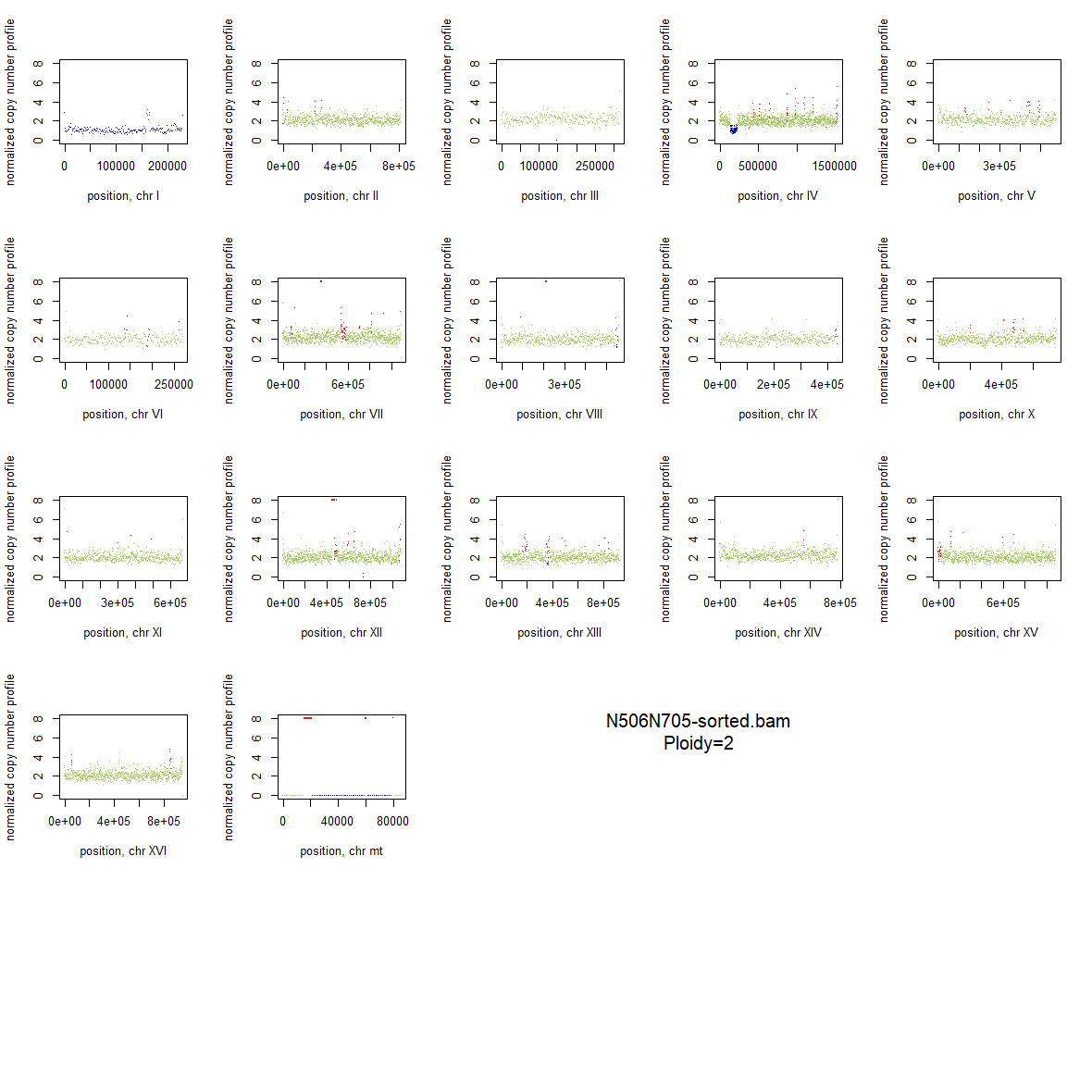

Supplement: Figure 2—source data 2. [file elife-79346-fig2-data2.zip › Figure2-source data 1/pACT1-sec53-V238M/2x_V238M_51.png]

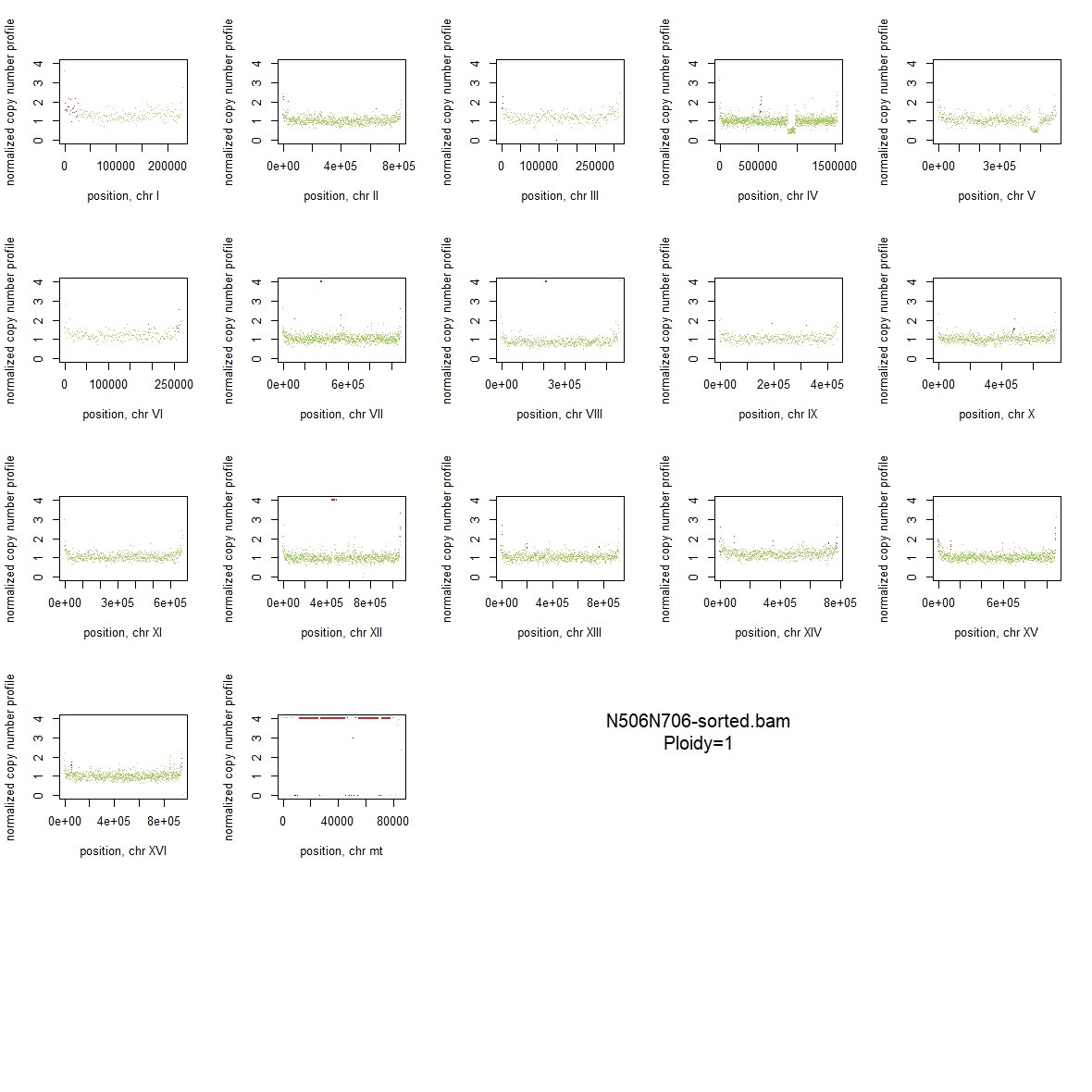

Supplement: Figure 2—source data 2. [file elife-79346-fig2-data2.zip › Figure2-source data 1/pACT1-sec53-V238M/2x_V238M_52.png]

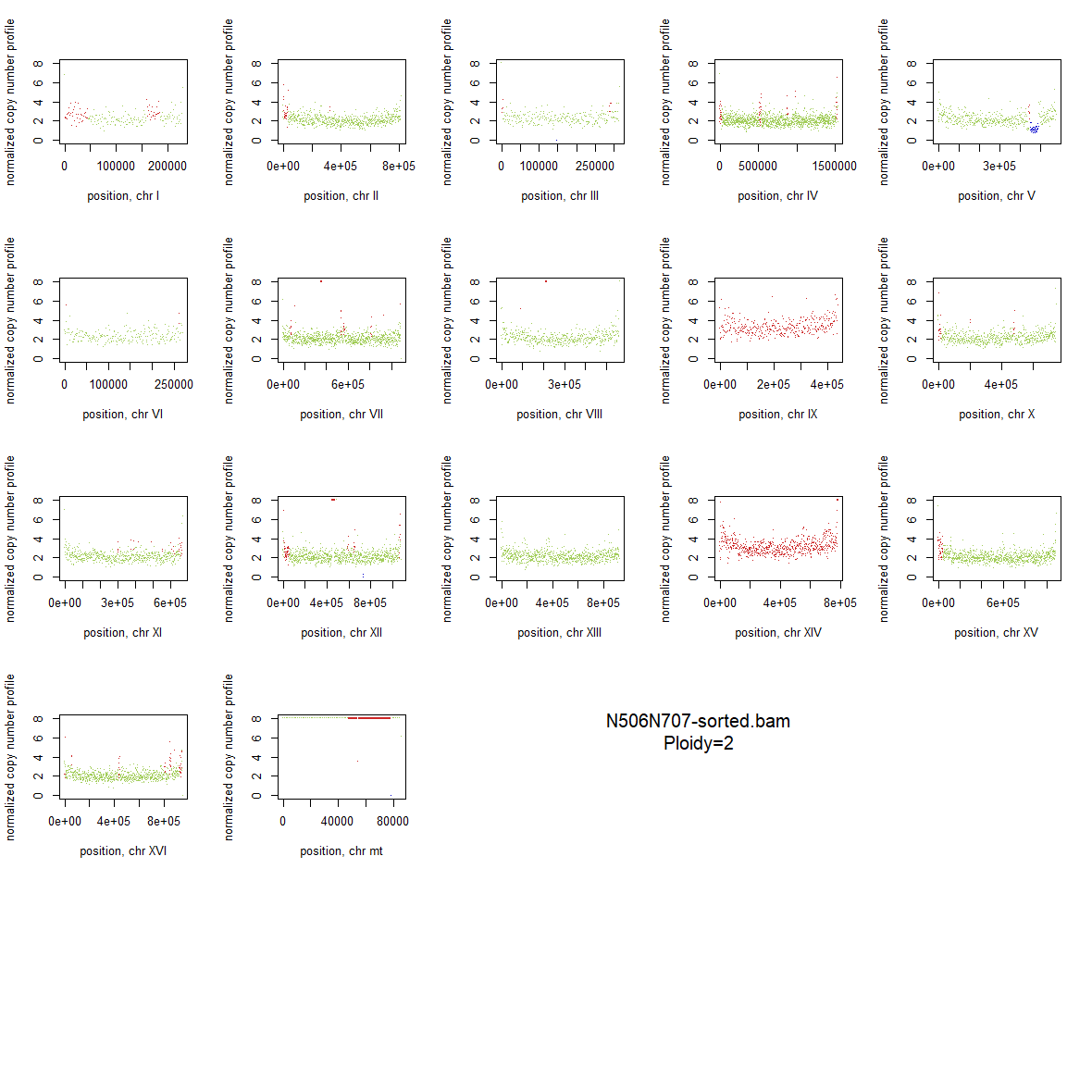

Supplement: Figure 2—source data 2. [file elife-79346-fig2-data2.zip › Figure2-source data 1/pACT1-sec53-V238M/2x_V238M_53.png]

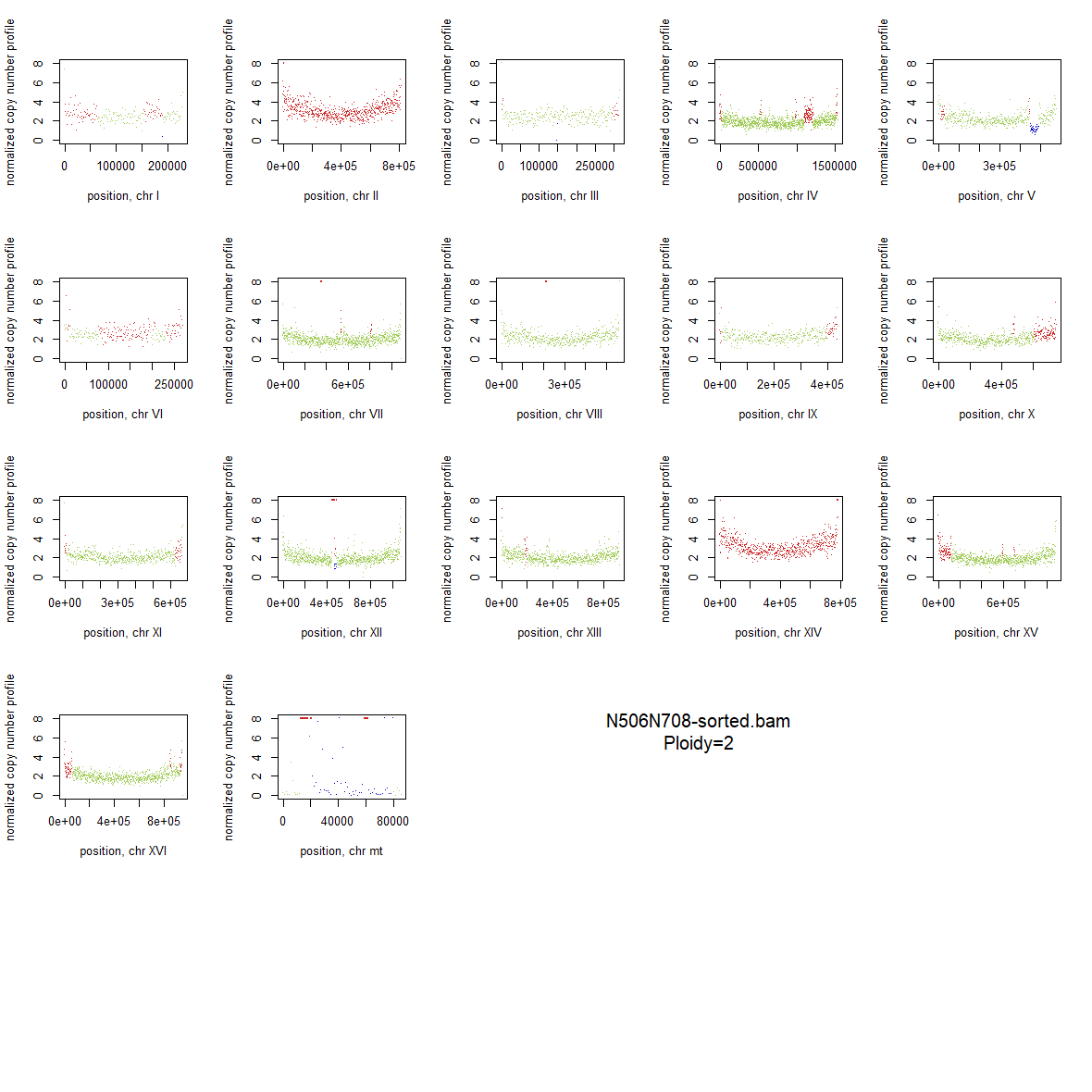

Supplement: Figure 2—source data 2. [file elife-79346-fig2-data2.zip › Figure2-source data 1/pACT1-sec53-V238M/2x_V238M_54.png]

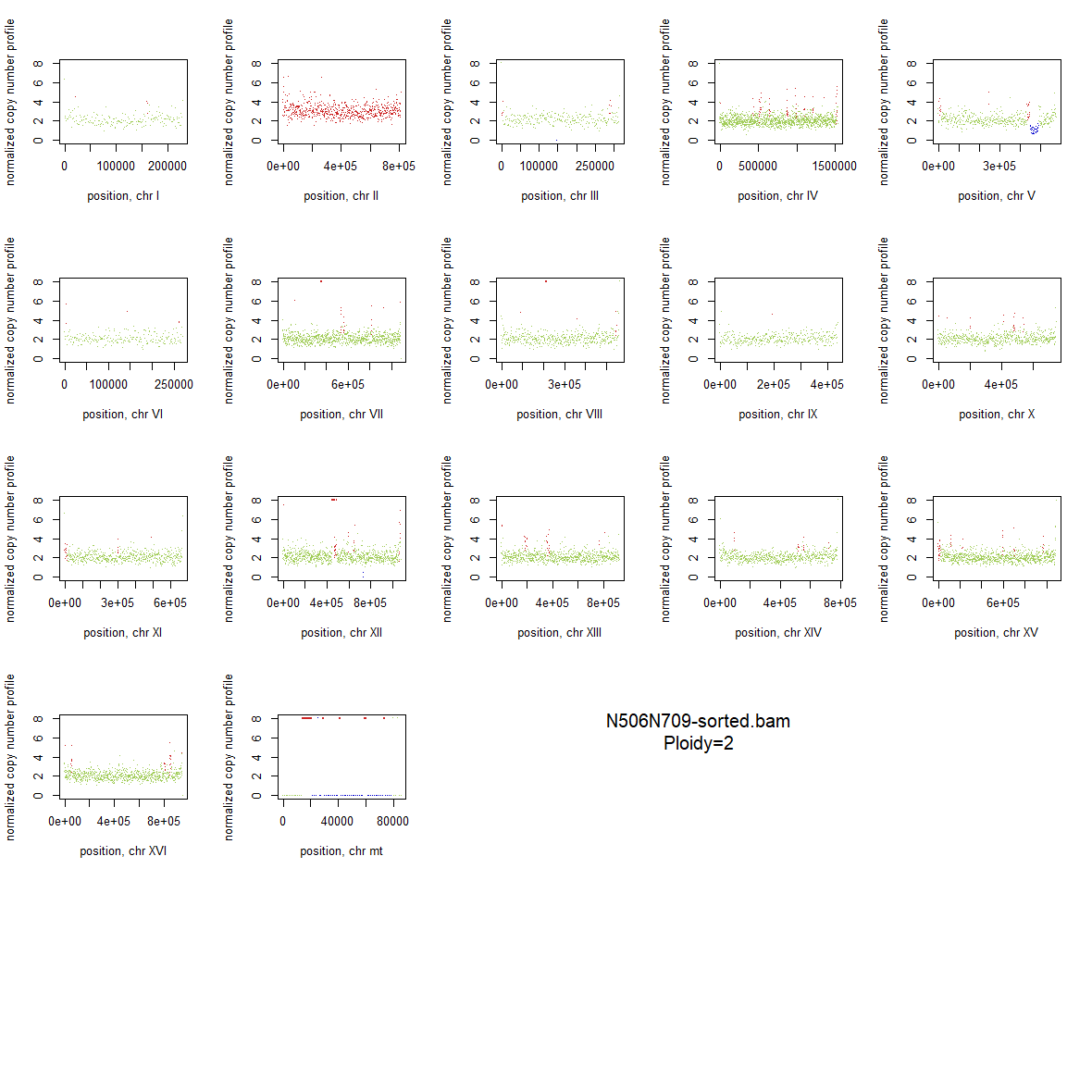

Supplement: Figure 2—source data 2. [file elife-79346-fig2-data2.zip › Figure2-source data 1/pACT1-sec53-V238M/2x_V238M_55.png]

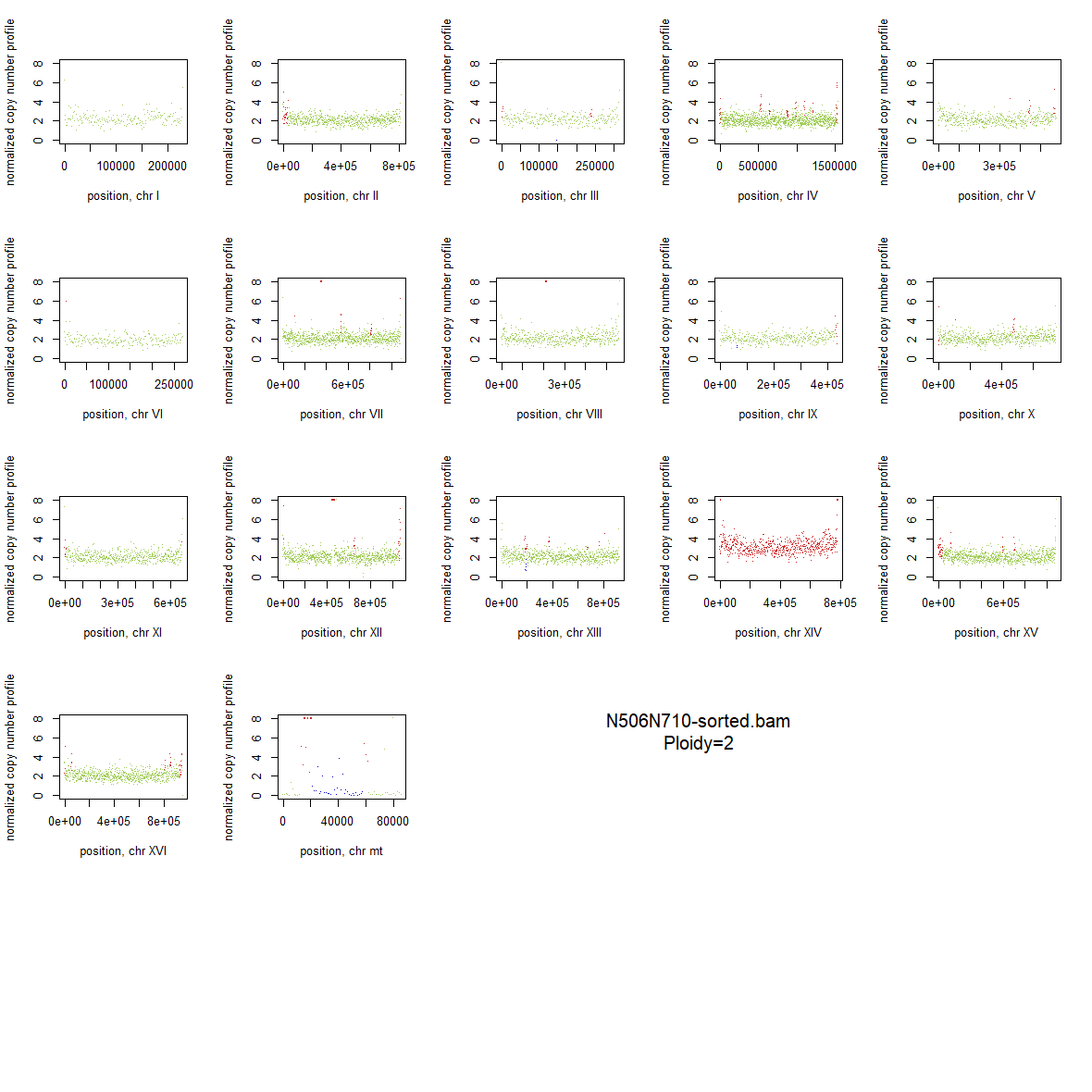

Supplement: Figure 2—source data 2. [file elife-79346-fig2-data2.zip › Figure2-source data 1/pACT1-sec53-V238M/2x_V238M_56.png]

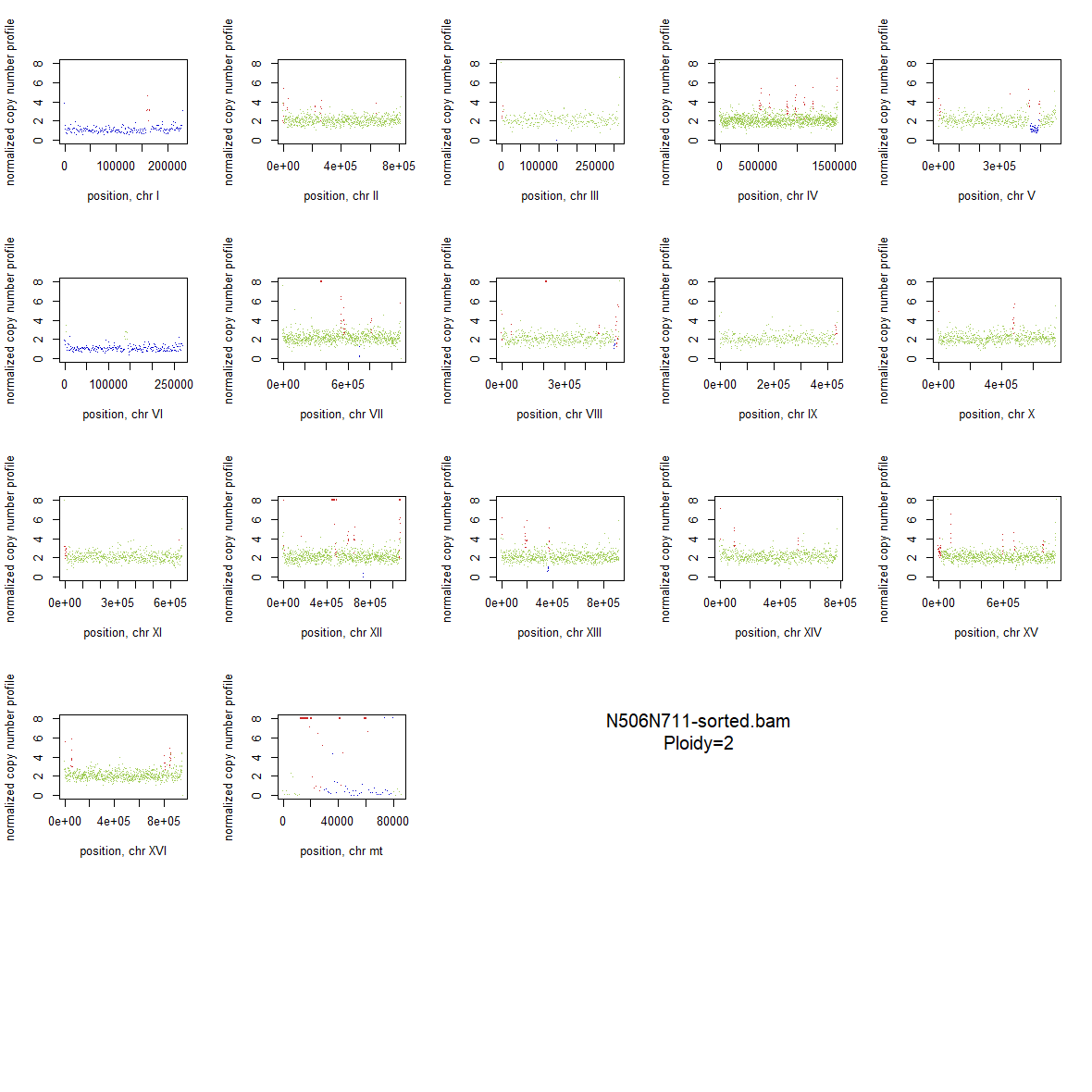

Supplement: Figure 2—source data 2. [file elife-79346-fig2-data2.zip › Figure2-source data 1/pACT1-sec53-V238M/2x_V238M_57.png]

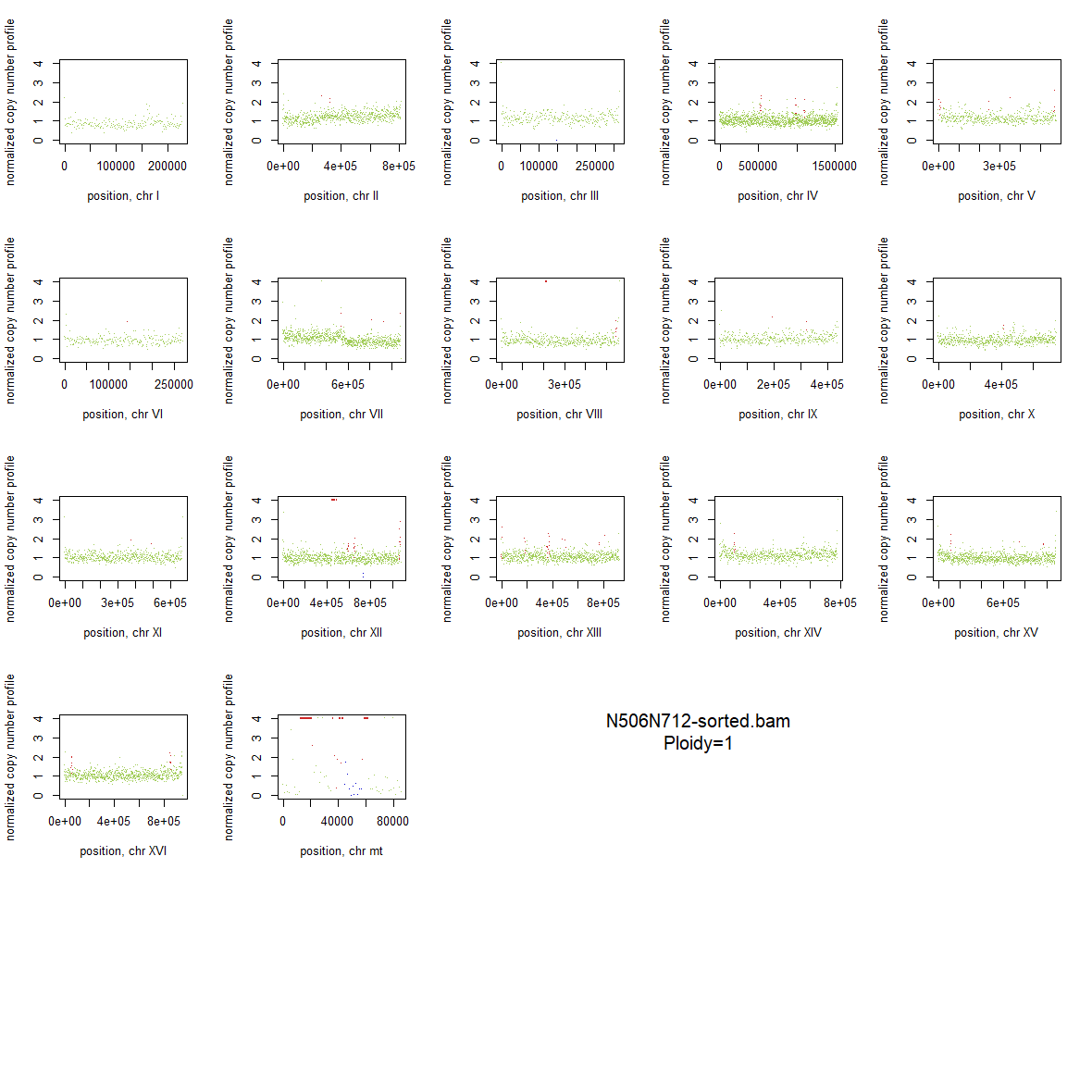

Supplement: Figure 2—source data 2. [file elife-79346-fig2-data2.zip › Figure2-source data 1/pACT1-sec53-V238M/2x_V238M_58.png]

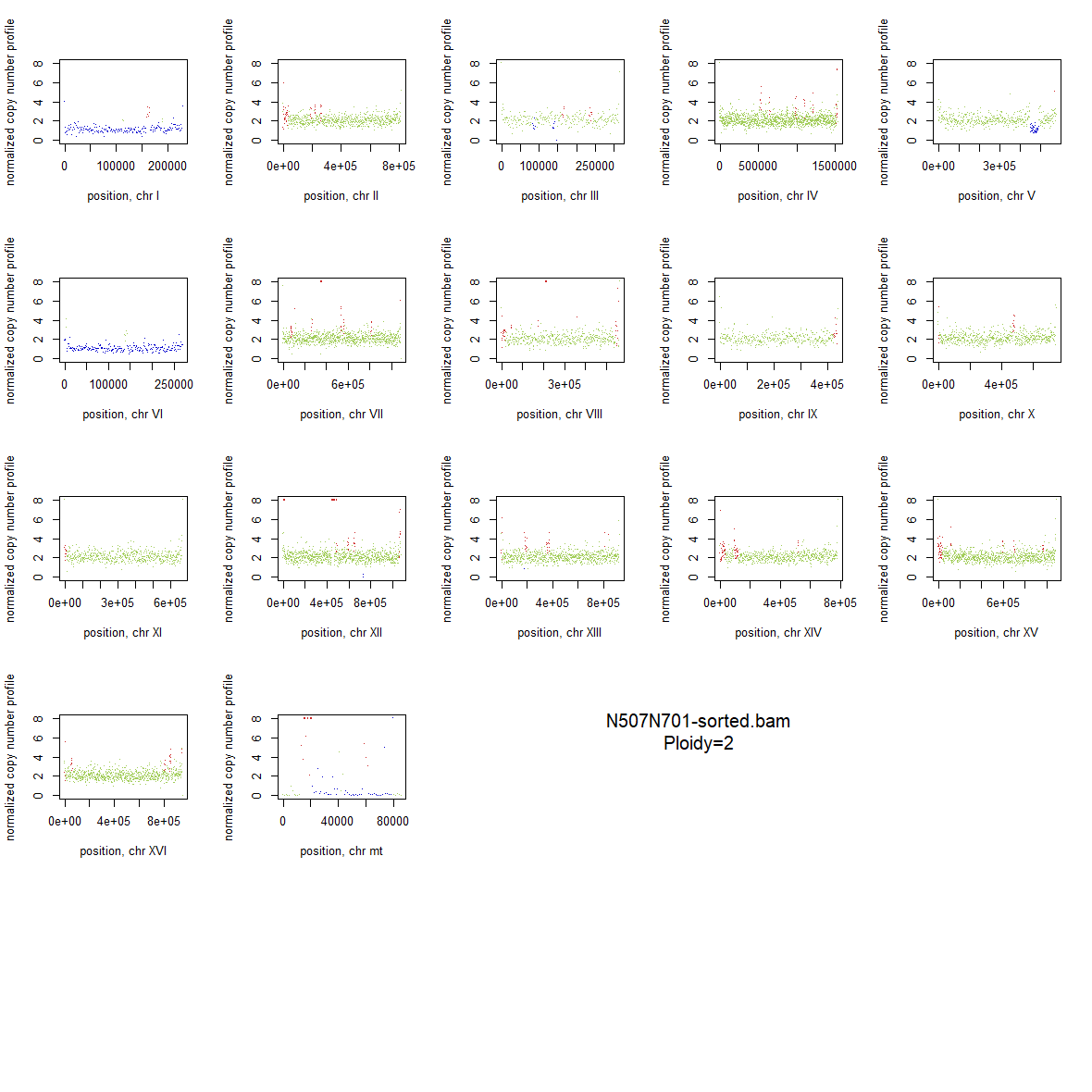

Supplement: Figure 2—source data 2. [file elife-79346-fig2-data2.zip › Figure2-source data 1/pACT1-sec53-V238M/2x_V238M_59.png]

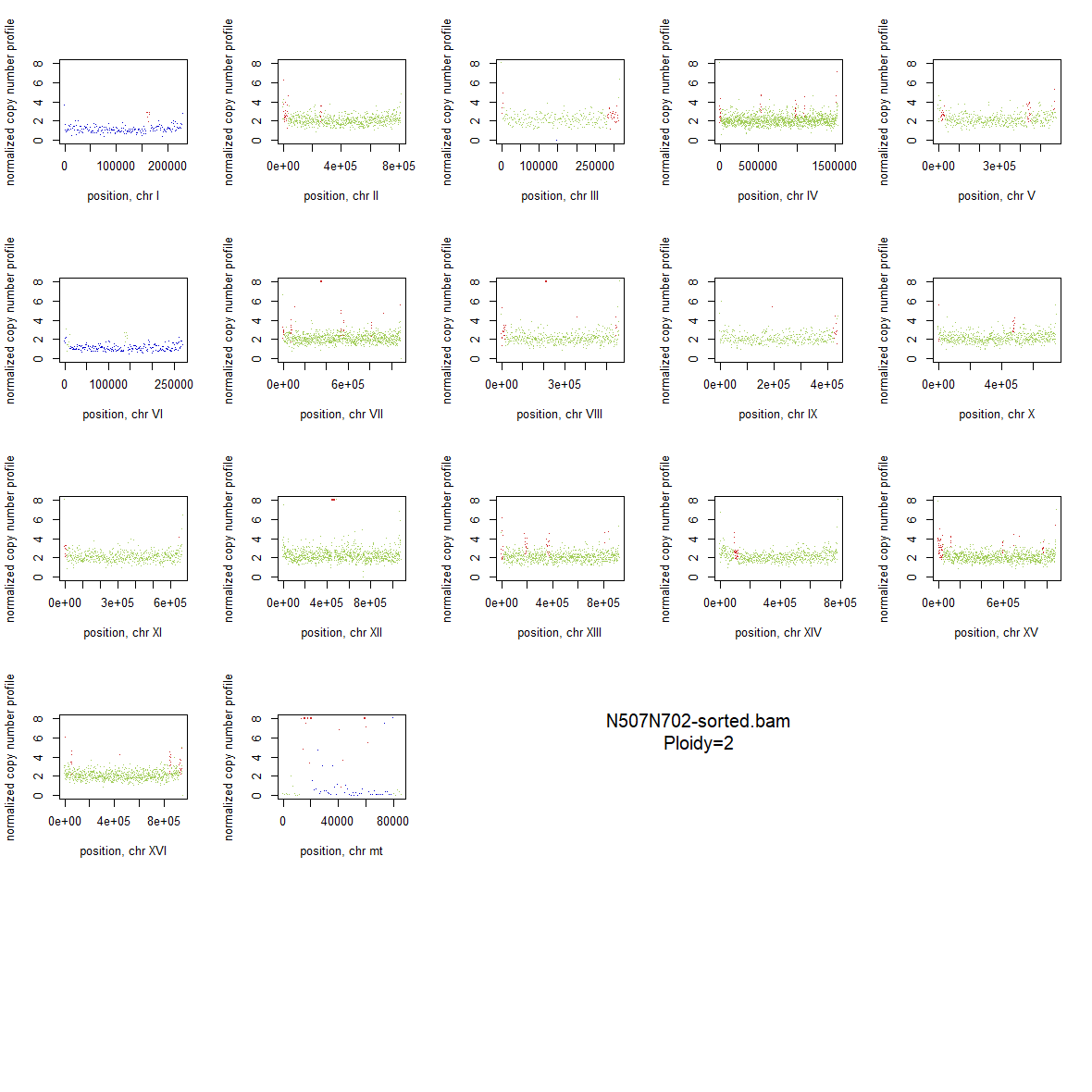

Supplement: Figure 2—source data 2. [file elife-79346-fig2-data2.zip › Figure2-source data 1/pACT1-sec53-V238M/2x_V238M_60.png]

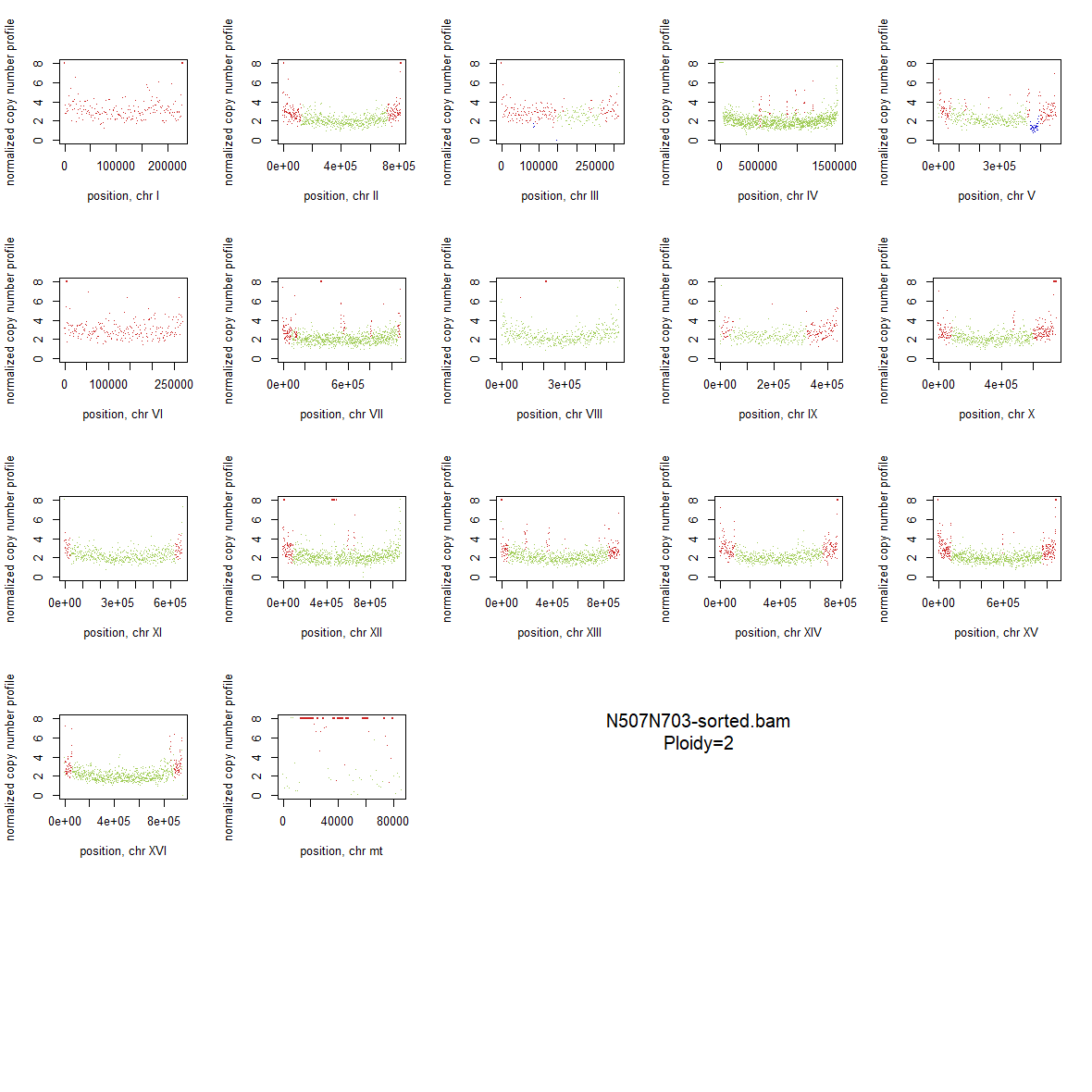

Supplement: Figure 2—source data 2. [file elife-79346-fig2-data2.zip › Figure2-source data 1/pACT1-sec53-V238M/2x_V238M_61.png]

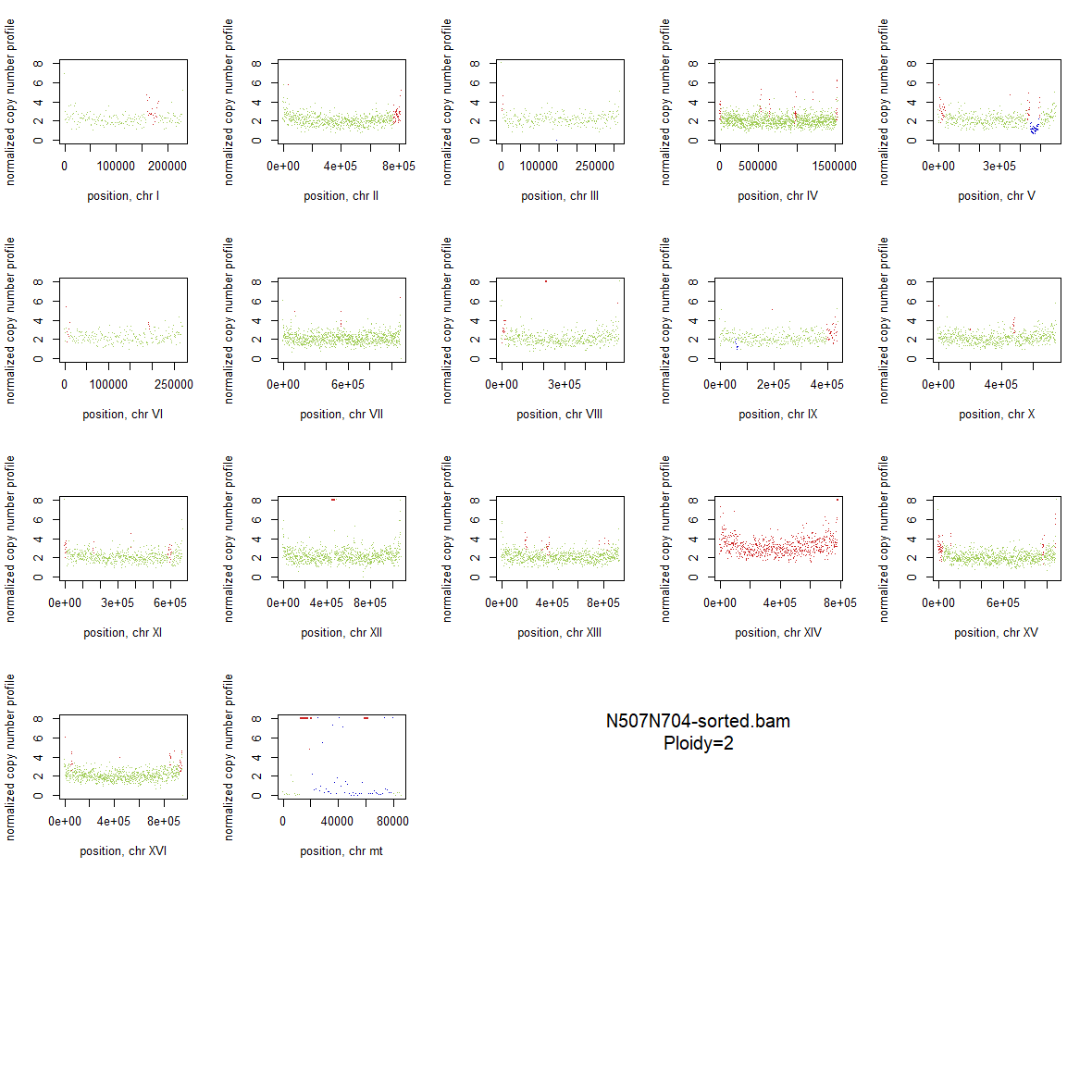

Supplement: Figure 2—source data 2. [file elife-79346-fig2-data2.zip › Figure2-source data 1/pACT1-sec53-V238M/2x_V238M_62.png]

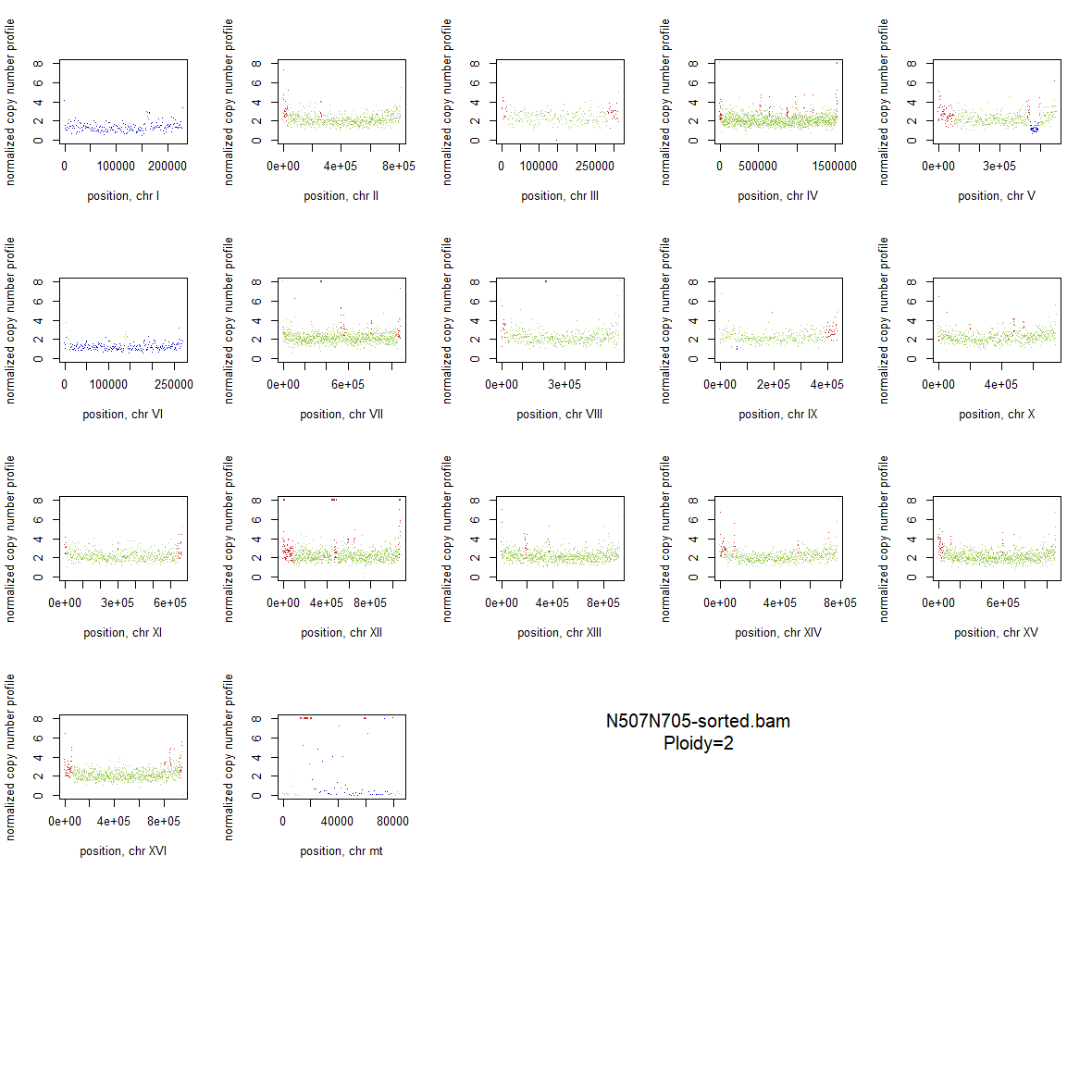

Supplement: Figure 2—source data 2. [file elife-79346-fig2-data2.zip › Figure2-source data 1/pACT1-sec53-V238M/2x_V238M_63.png]

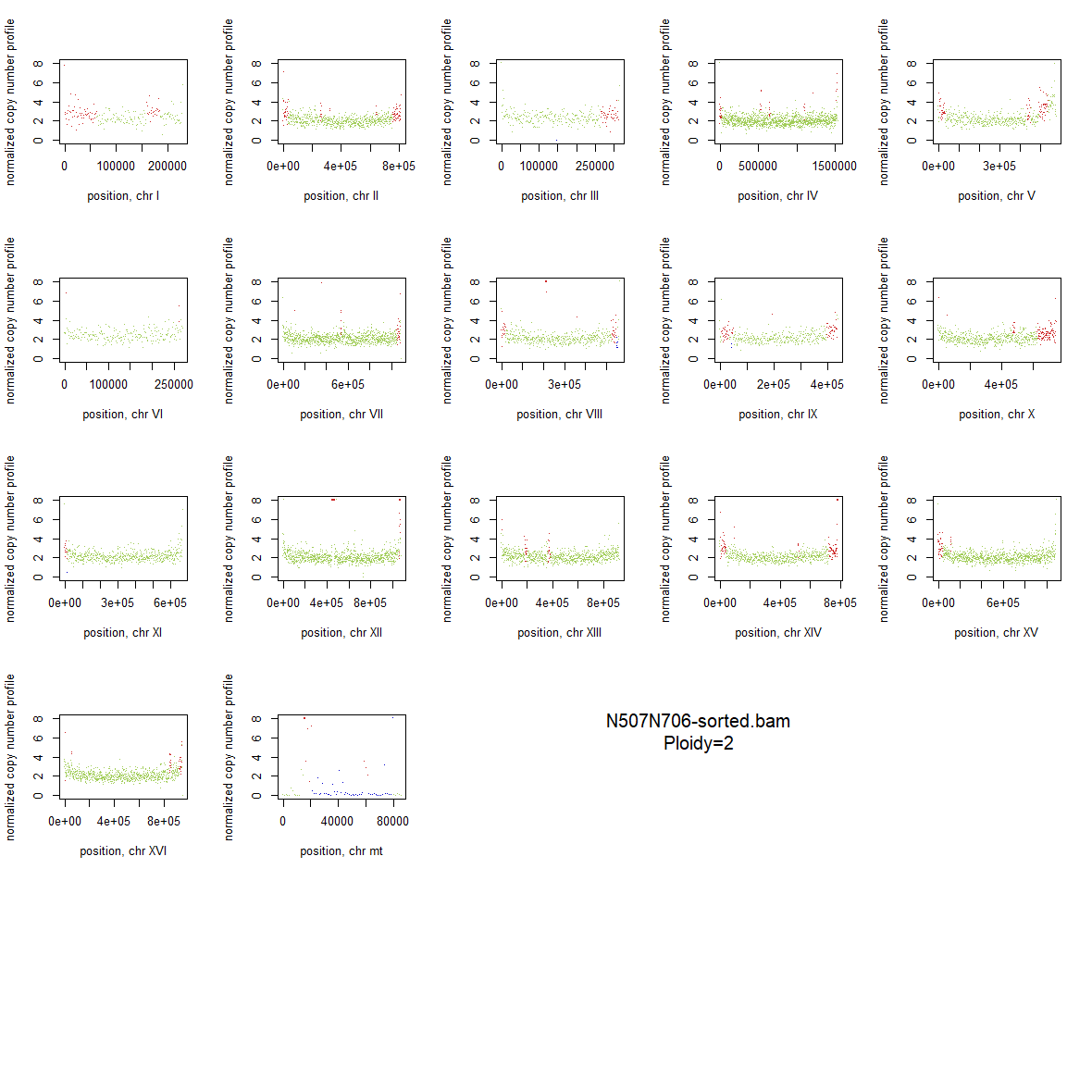

Supplement: Figure 2—source data 2. [file elife-79346-fig2-data2.zip › Figure2-source data 1/pACT1-sec53-V238M/2x_V238M_64.png]

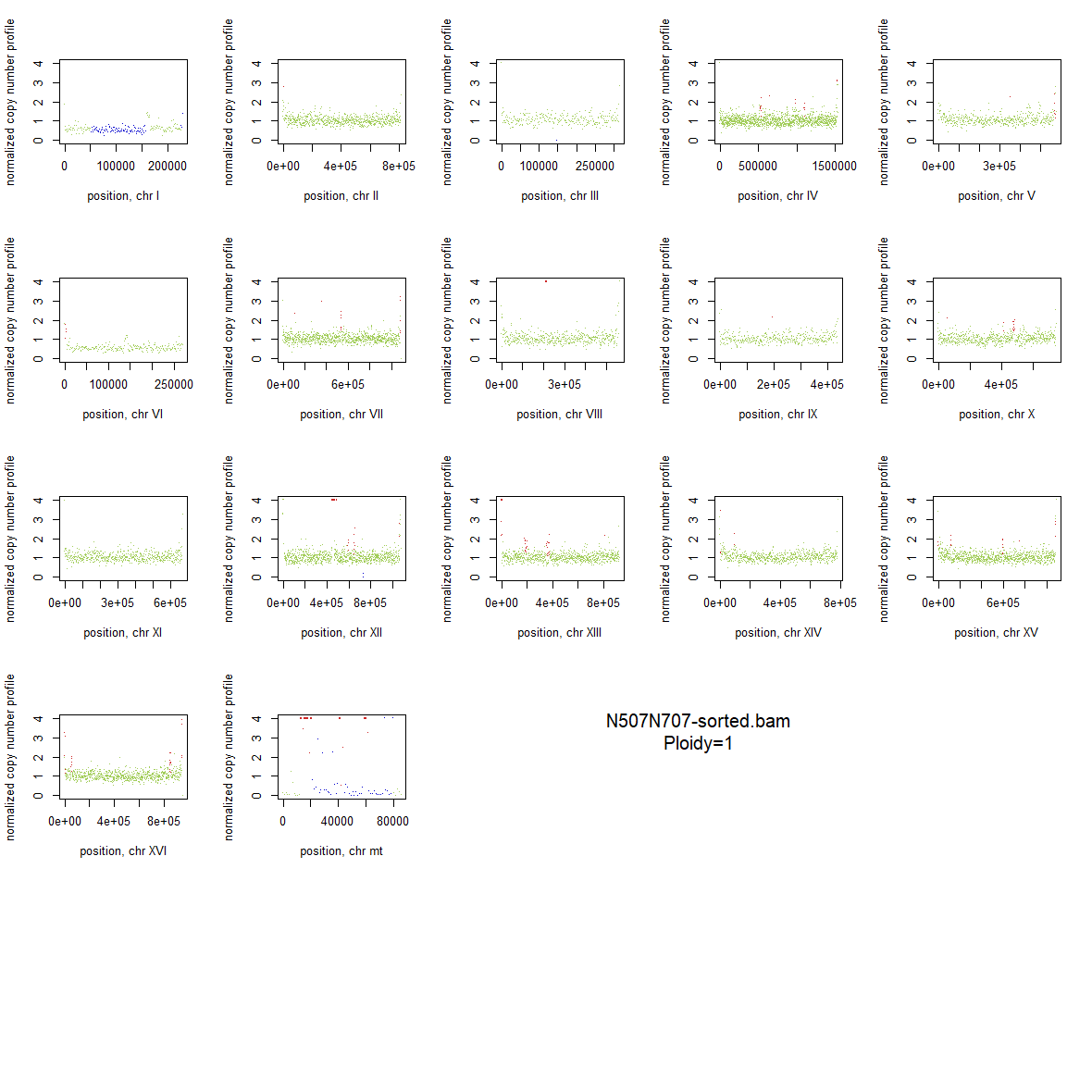

Supplement: Figure 2—source data 2. [file elife-79346-fig2-data2.zip › Figure2-source data 1/pACT1-sec53-V238M/2x_V238M_65.png]

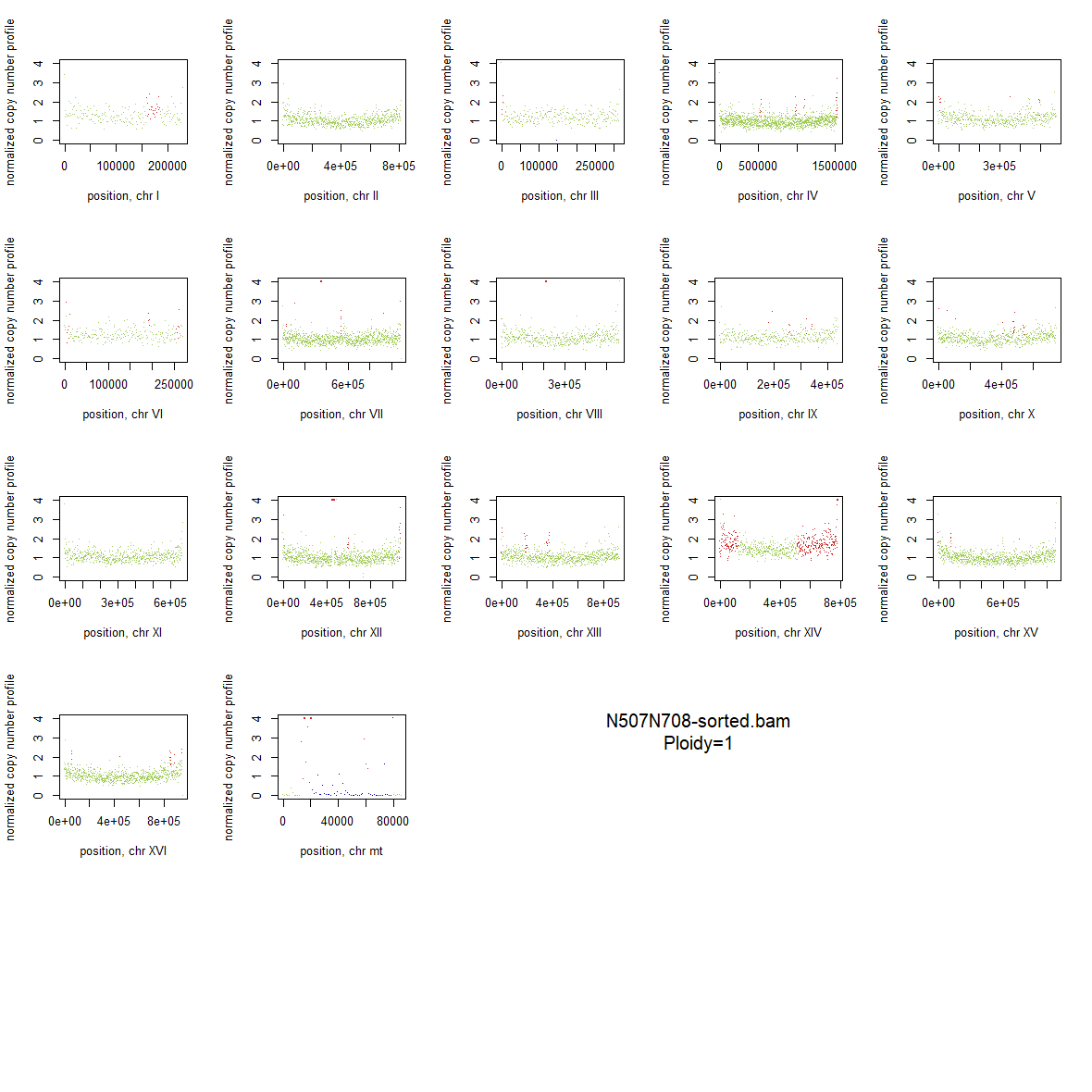

Supplement: Figure 2—source data 2. [file elife-79346-fig2-data2.zip › Figure2-source data 1/pACT1-sec53-V238M/2x_V238M_66.png]

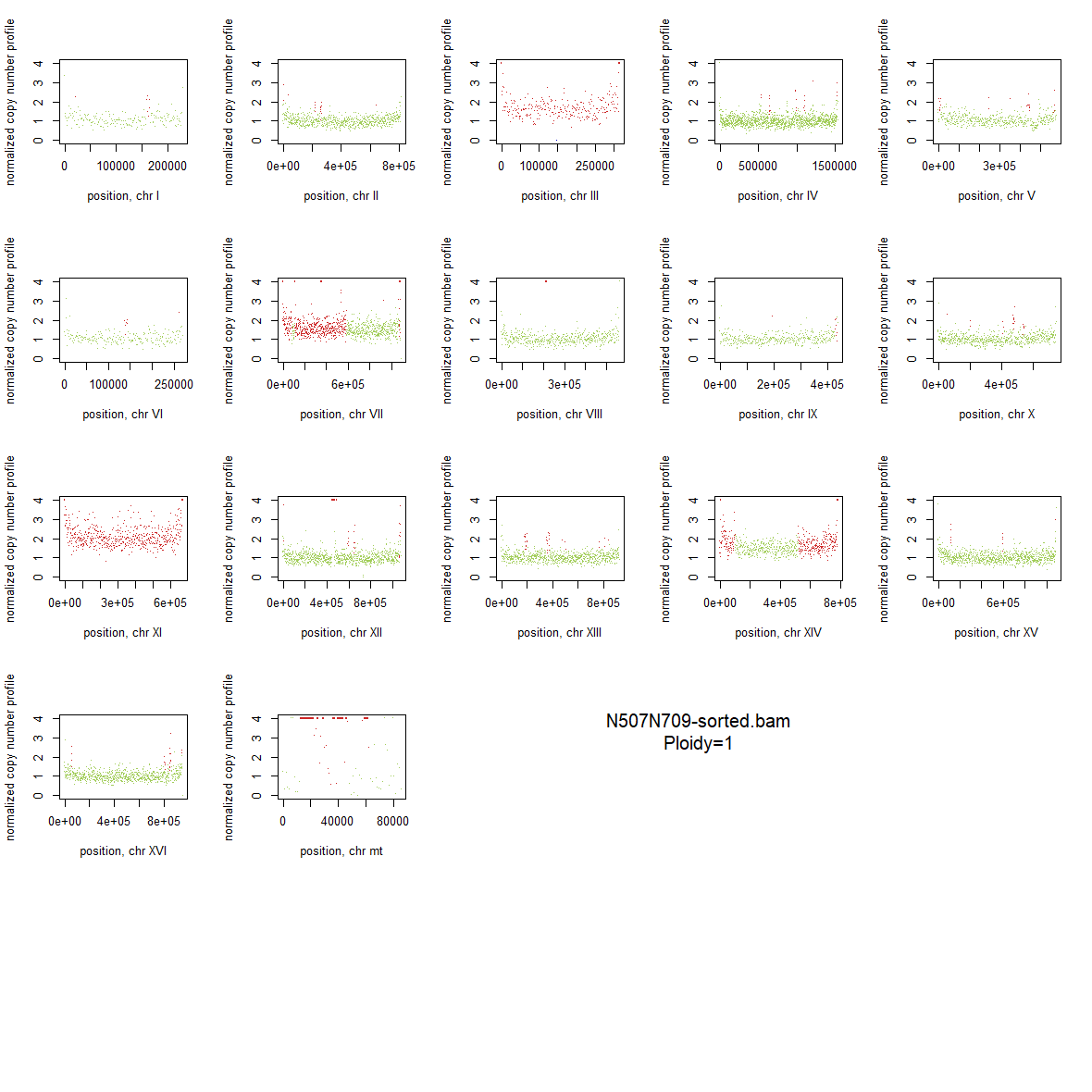

Supplement: Figure 2—source data 2. [file elife-79346-fig2-data2.zip › Figure2-source data 1/pACT1-sec53-V238M/2x_V238M_67.png]

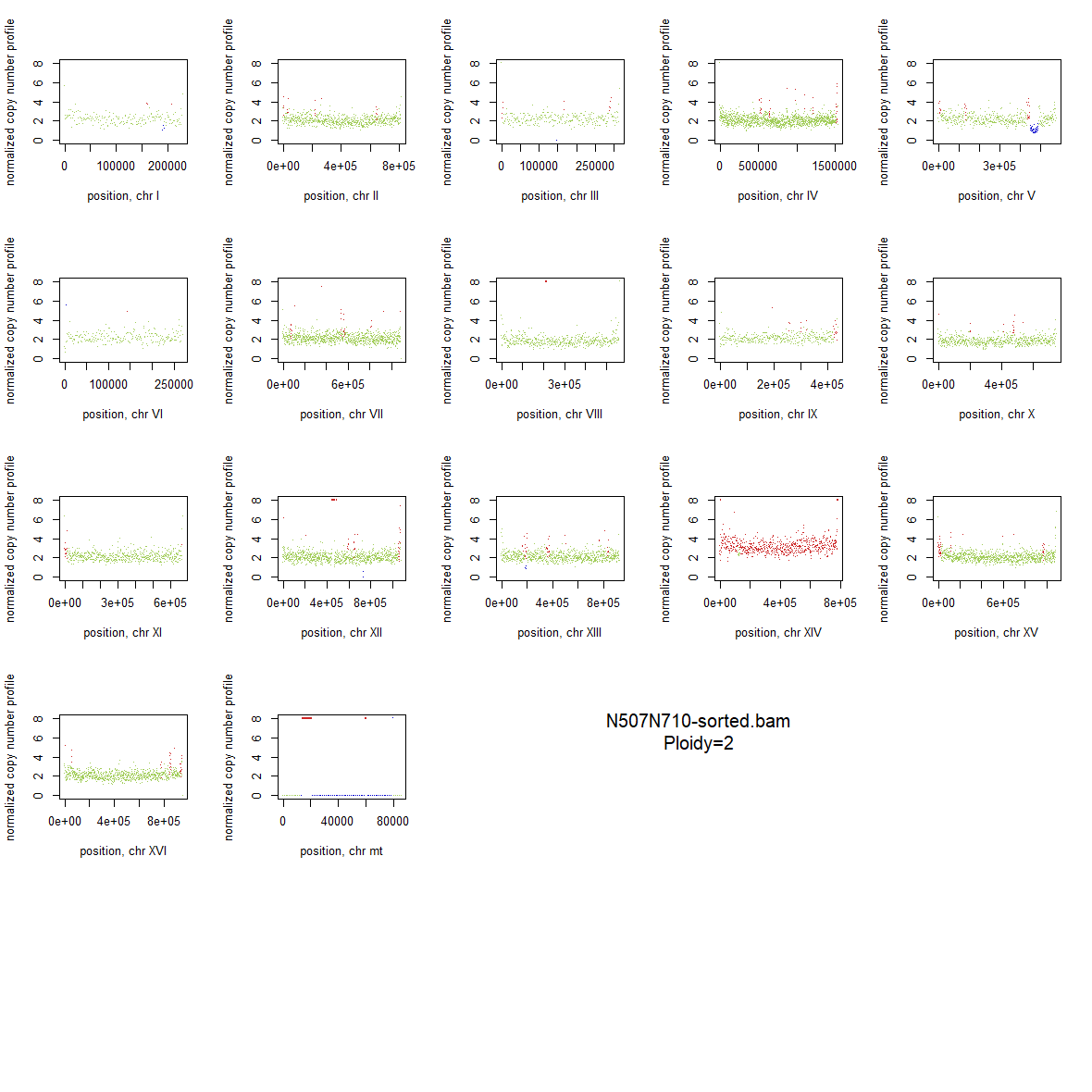

Supplement: Figure 2—source data 2. [file elife-79346-fig2-data2.zip › Figure2-source data 1/pACT1-sec53-V238M/2x_V238M_68.png]

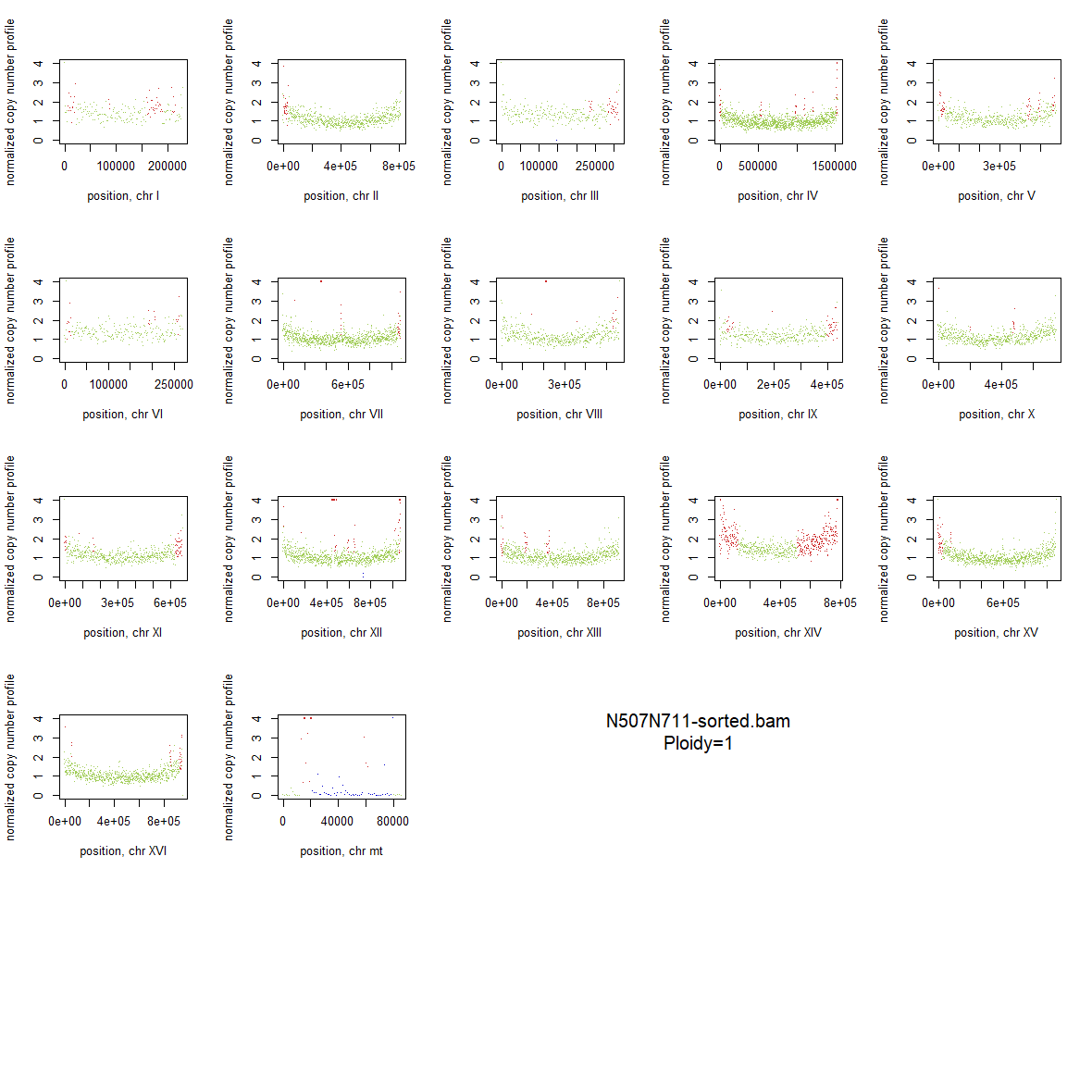

Supplement: Figure 2—source data 2. [file elife-79346-fig2-data2.zip › Figure2-source data 1/pACT1-sec53-V238M/2x_V238M_69.png]

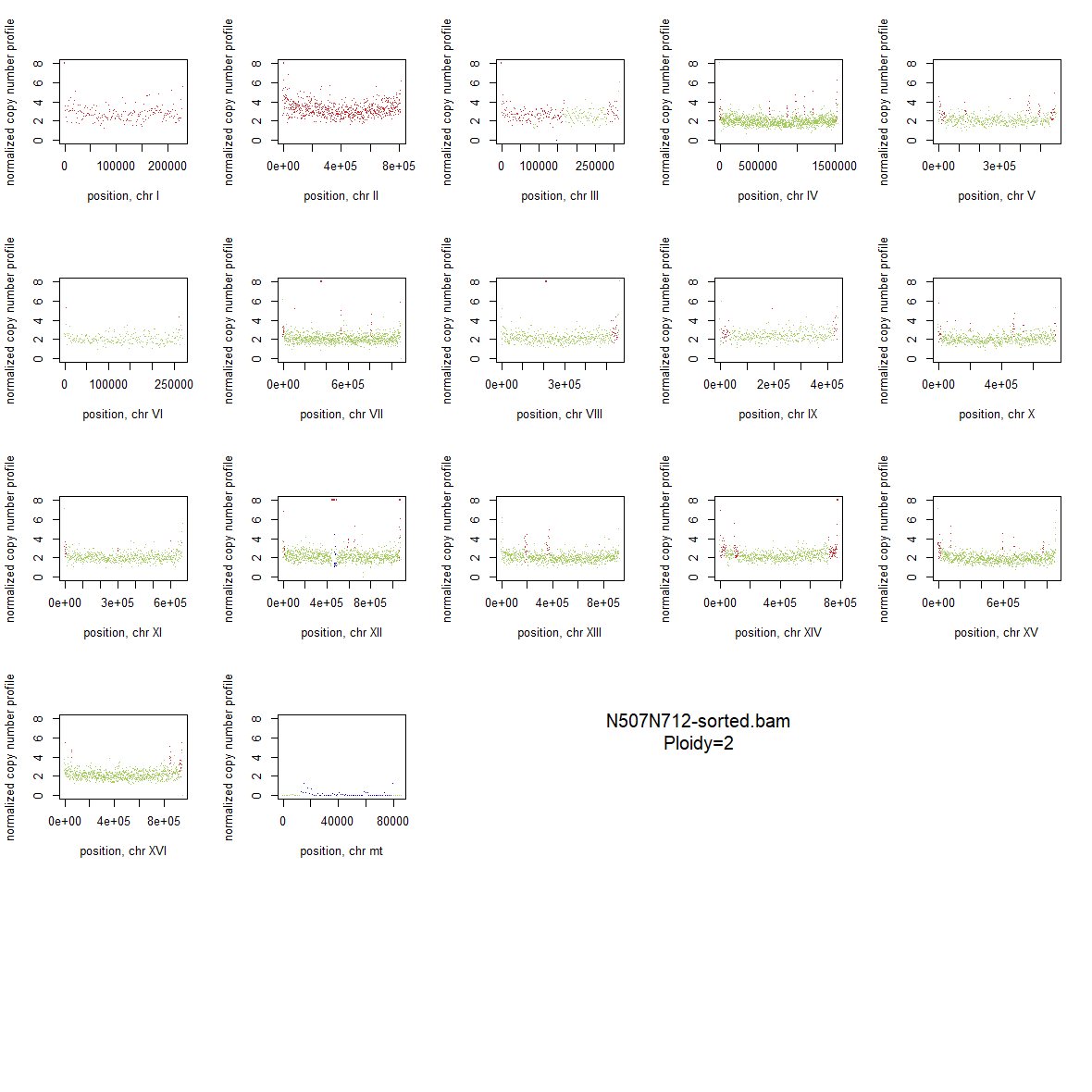

Supplement: Figure 2—source data 2. [file elife-79346-fig2-data2.zip › Figure2-source data 1/pACT1-sec53-V238M/2x_V238M_70.png]

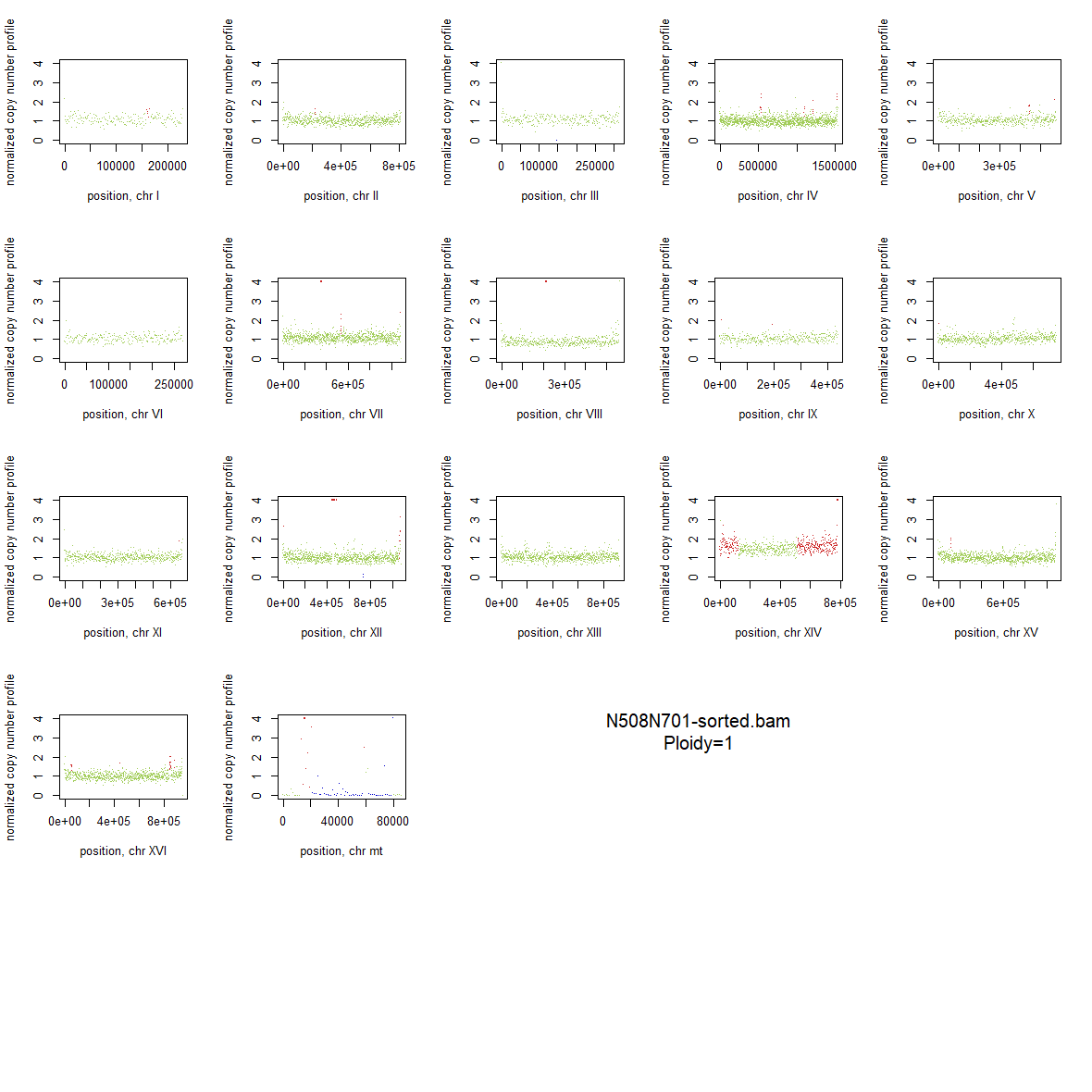

Supplement: Figure 2—source data 2. [file elife-79346-fig2-data2.zip › Figure2-source data 1/pACT1-sec53-V238M/2x_V238M_71.png]

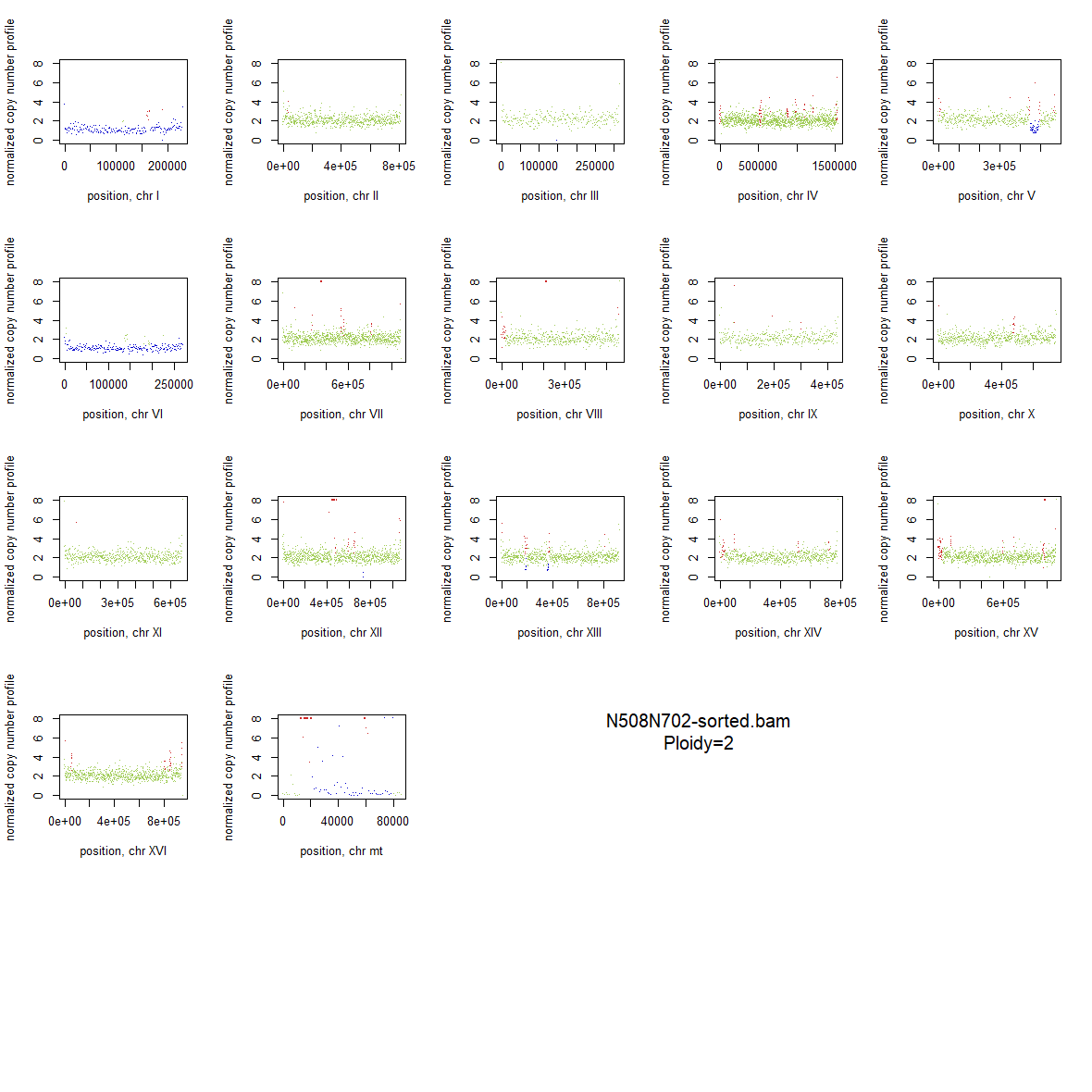

Supplement: Figure 2—source data 2. [file elife-79346-fig2-data2.zip › Figure2-source data 1/pACT1-sec53-V238M/2x_V238M_72.png]

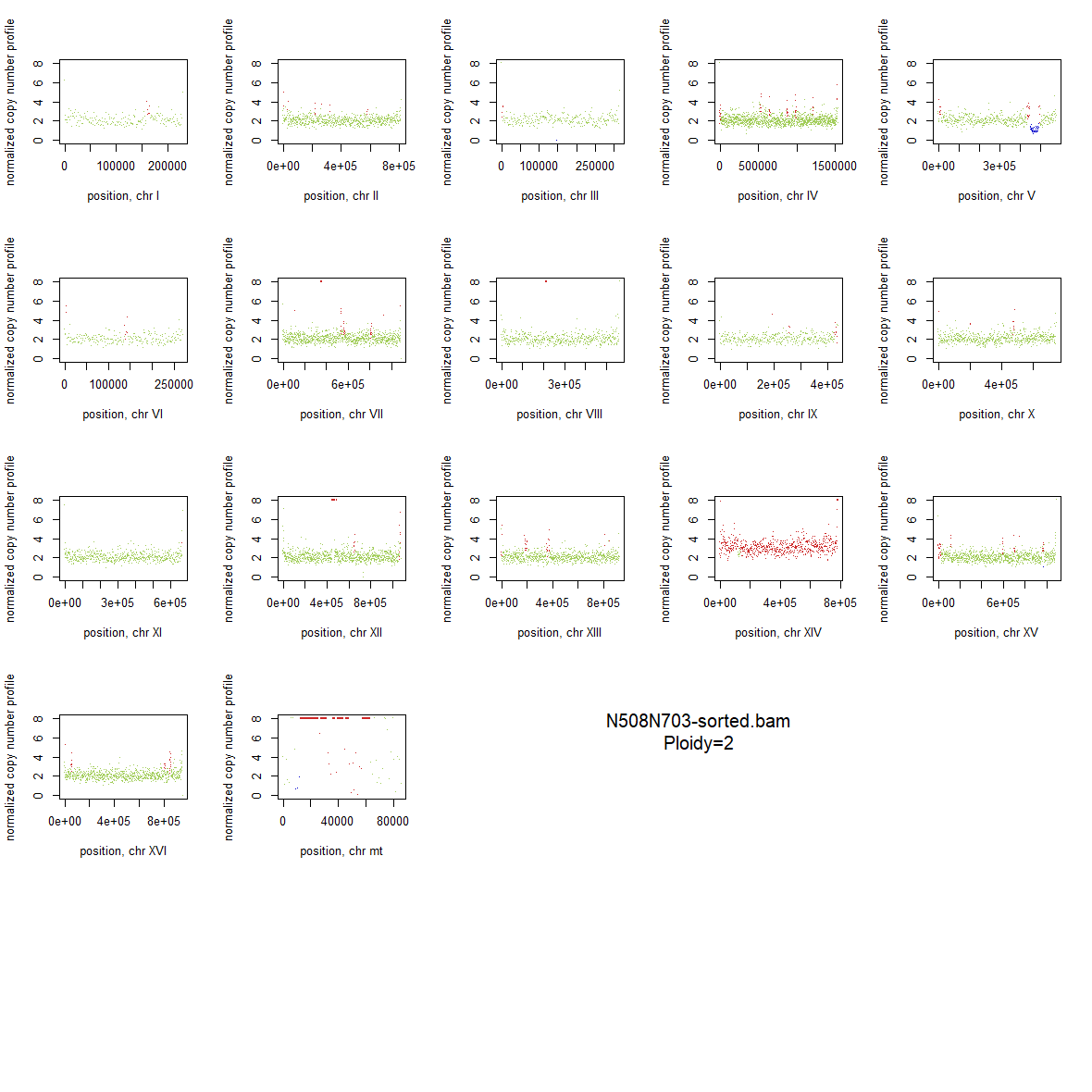

Supplement: Figure 2—source data 2. [file elife-79346-fig2-data2.zip › Figure2-source data 1/pACT1-sec53-V238M/2x_V238M_73.png]

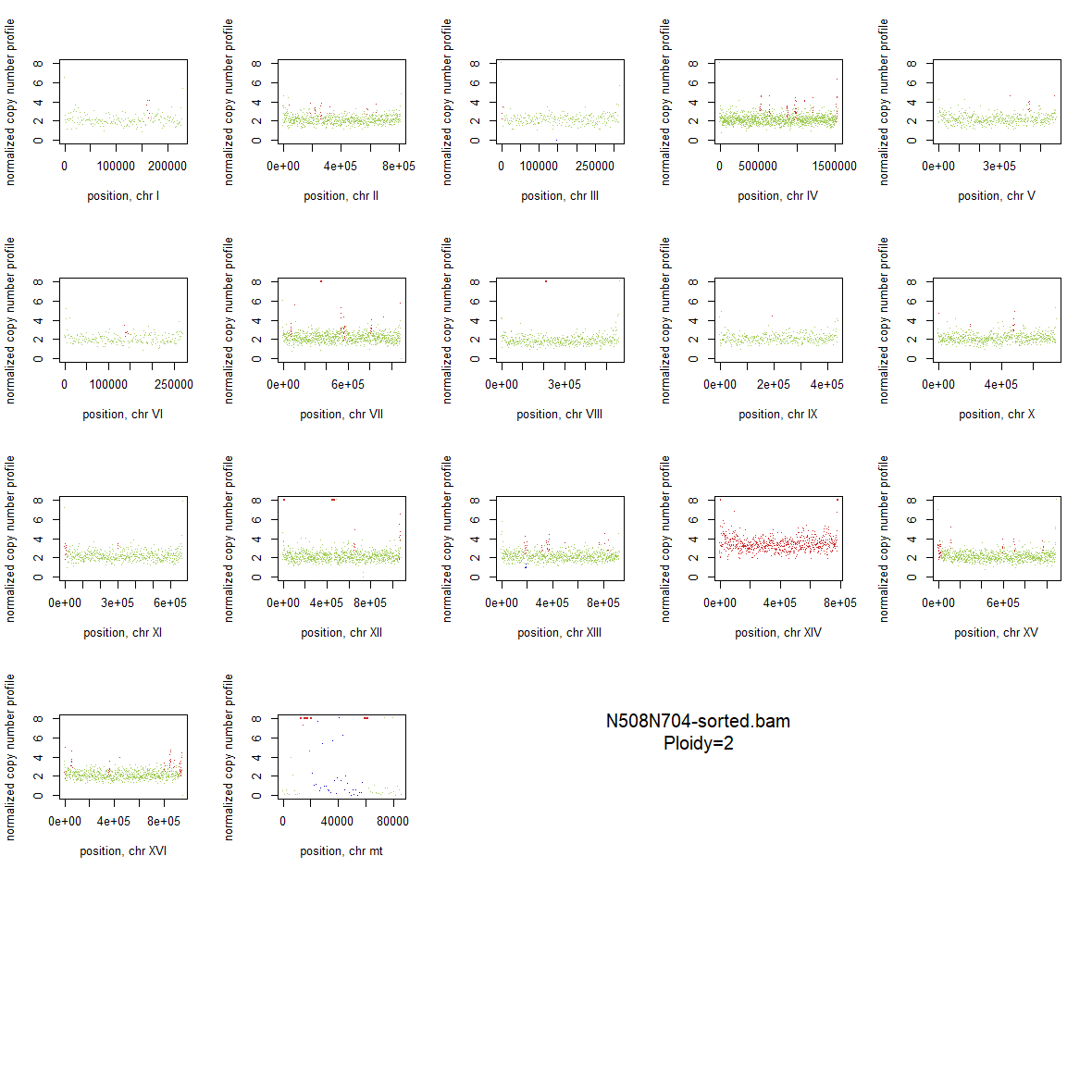

Supplement: Figure 2—source data 2. [file elife-79346-fig2-data2.zip › Figure2-source data 1/pACT1-sec53-V238M/2x_V238M_74.png]

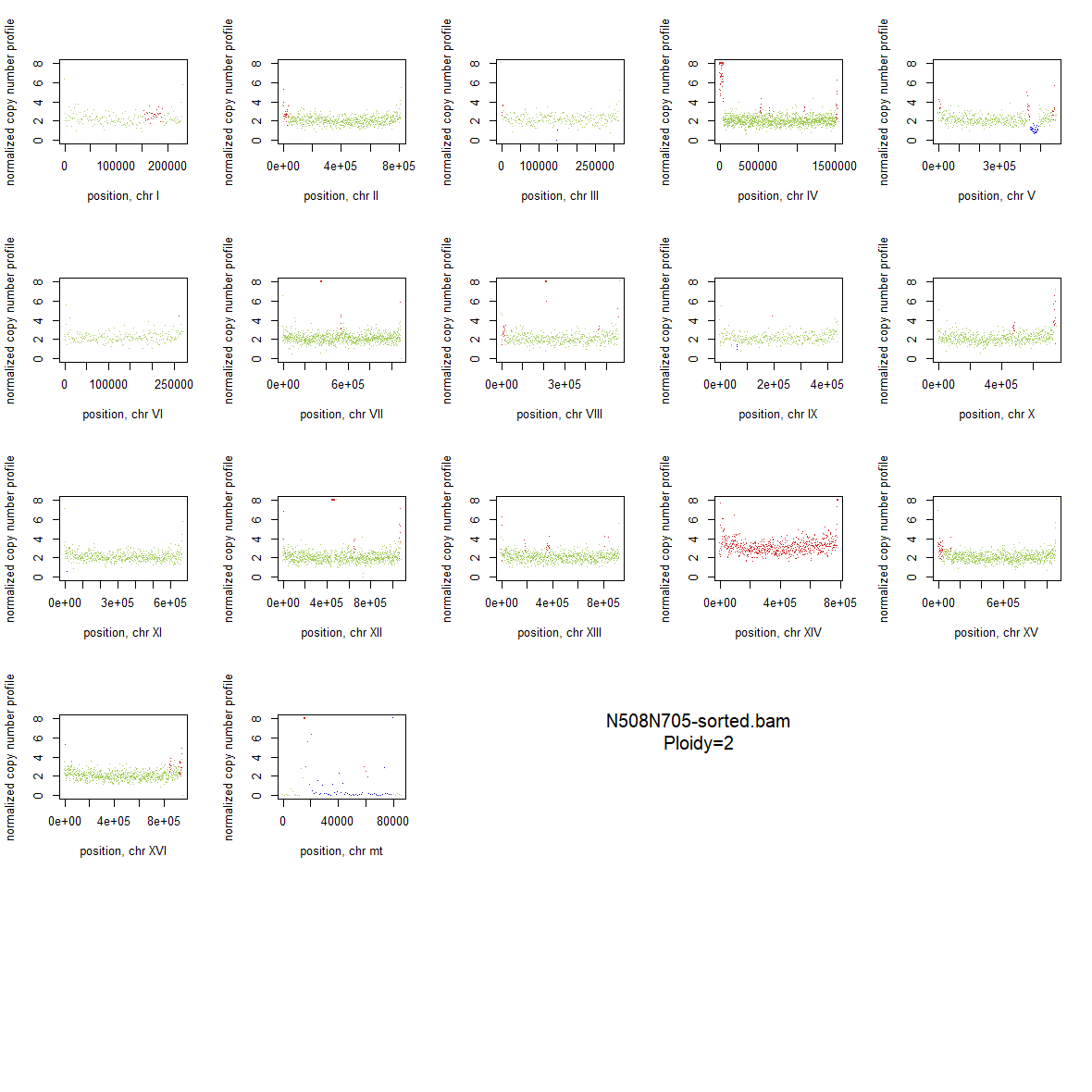

Supplement: Figure 2—source data 2. [file elife-79346-fig2-data2.zip › Figure2-source data 1/pACT1-sec53-V238M/2x_V238M_75.png]

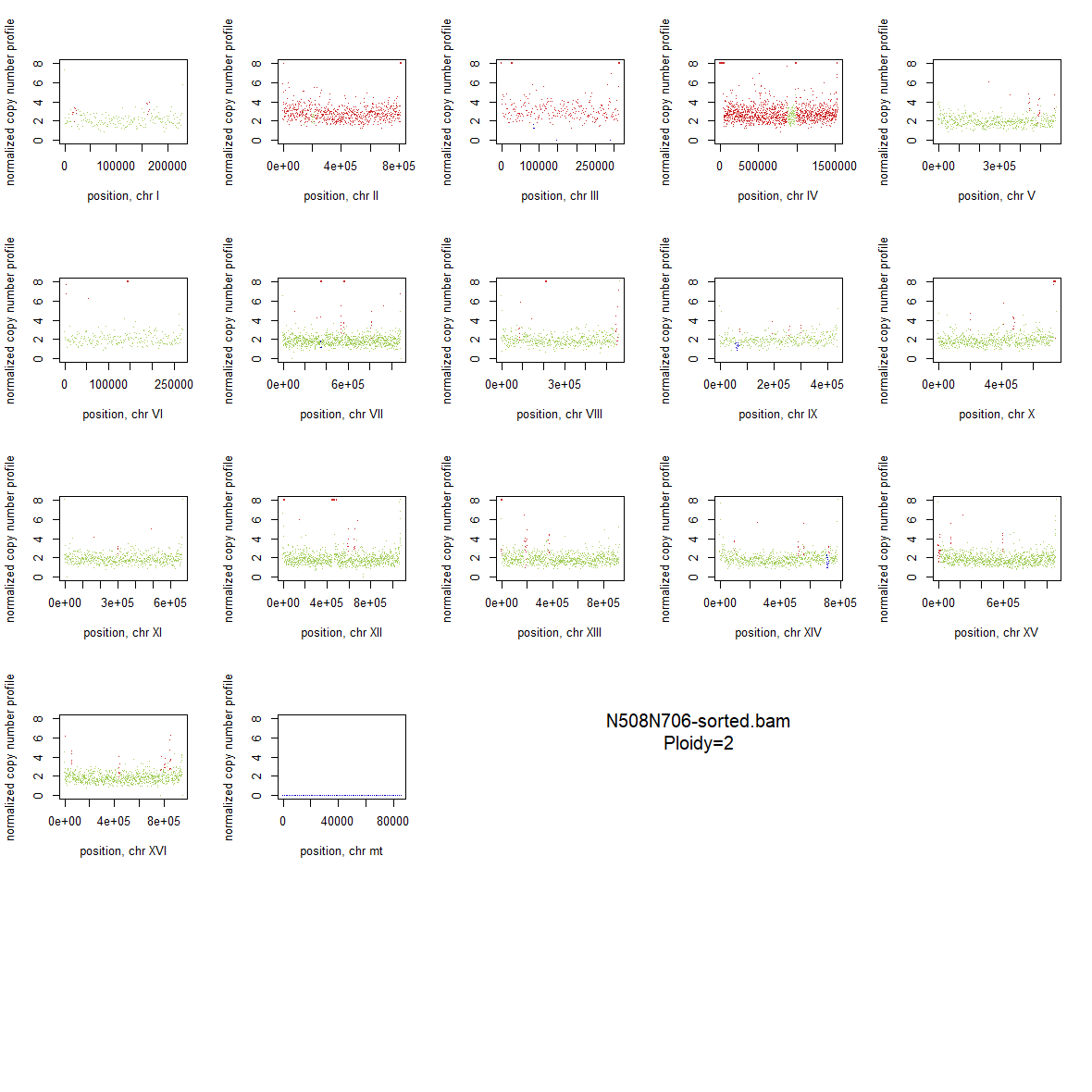

Supplement: Figure 2—source data 2. [file elife-79346-fig2-data2.zip › Figure2-source data 1/pACT1-sec53-V238M/2x_V238M_76.png]

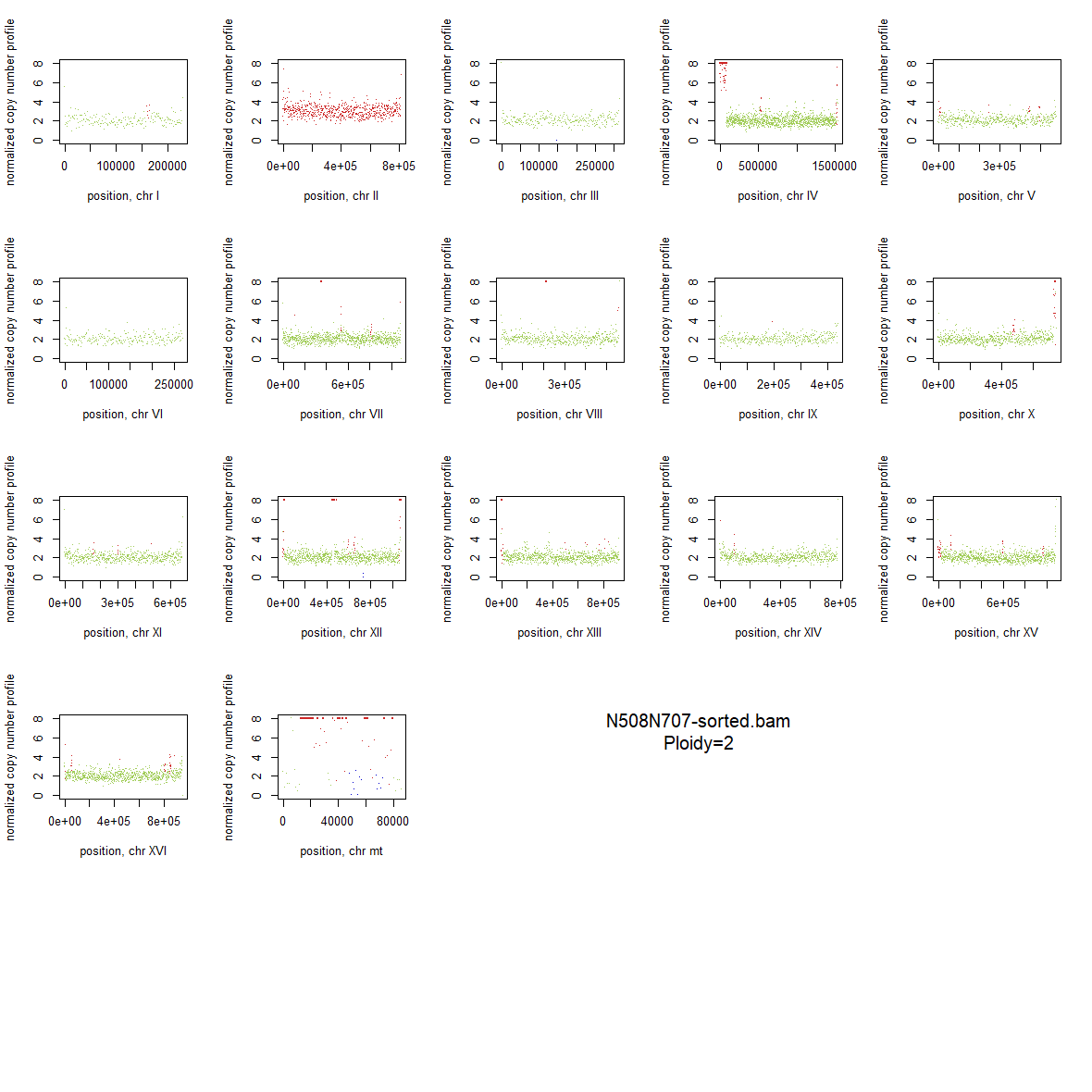

Supplement: Figure 2—source data 2. [file elife-79346-fig2-data2.zip › Figure2-source data 1/pACT1-sec53-V238M/2x_V238M_77.png]

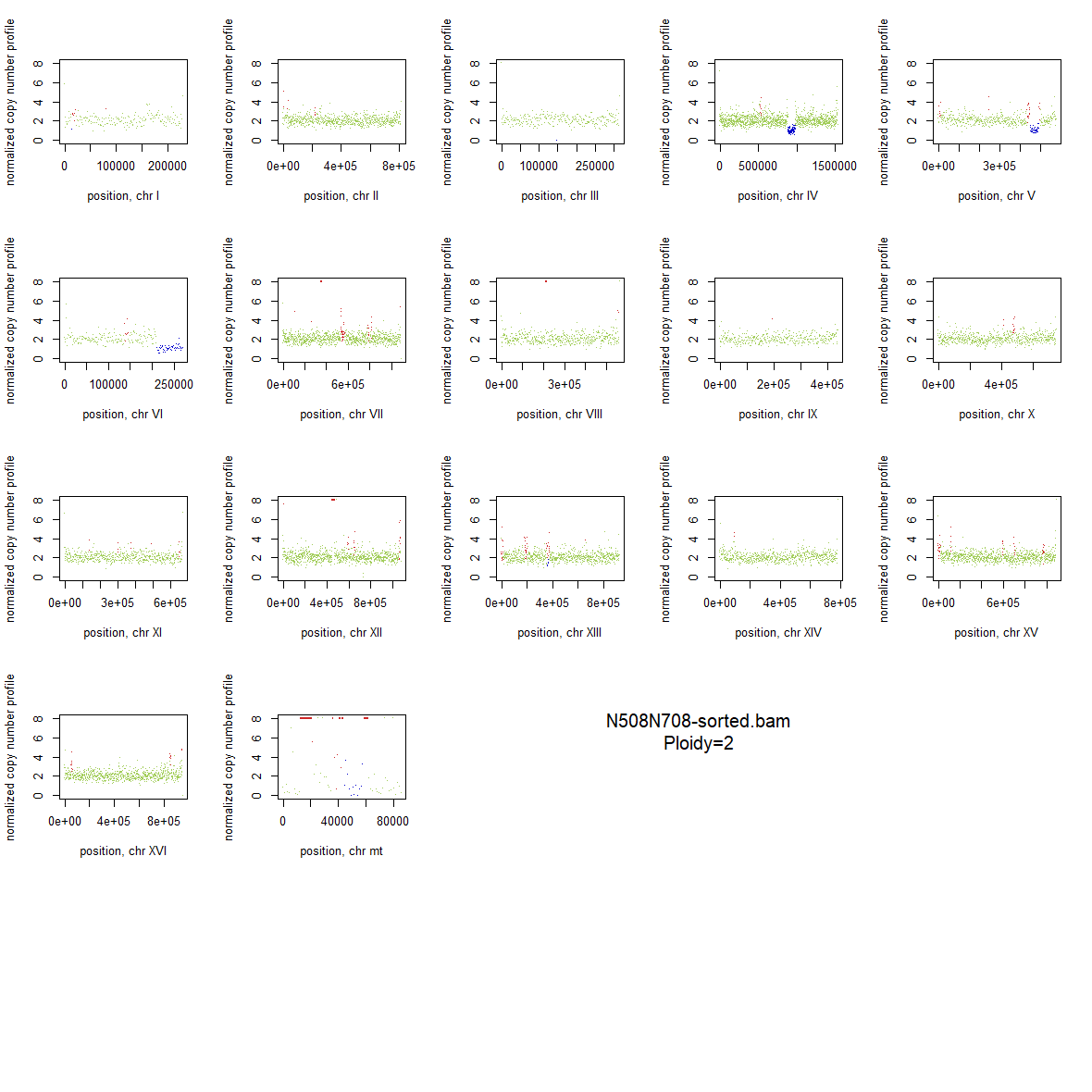

Supplement: Figure 2—source data 2. [file elife-79346-fig2-data2.zip › Figure2-source data 1/pACT1-sec53-V238M/2x_V238M_78.png]

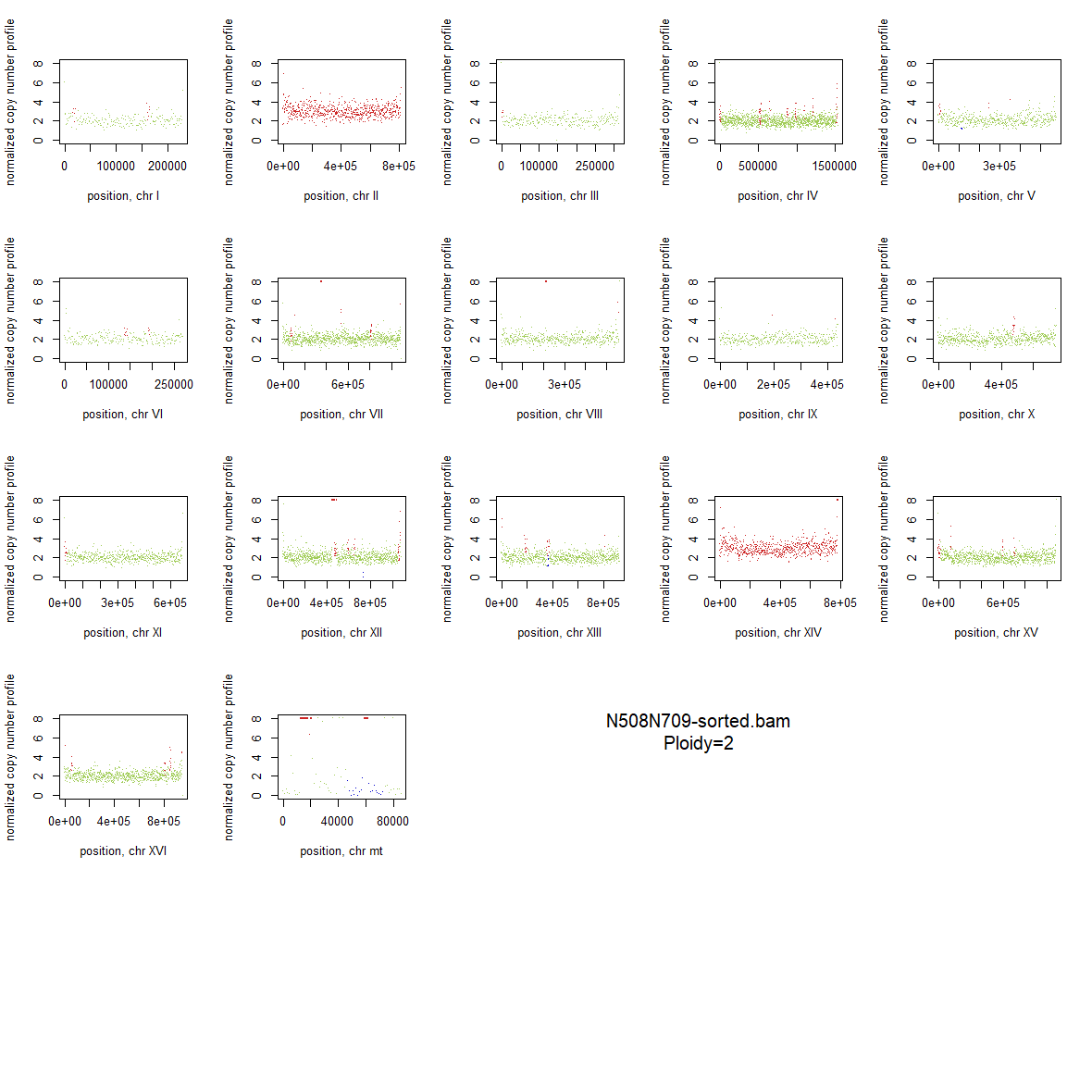

Supplement: Figure 2—source data 2. [file elife-79346-fig2-data2.zip › Figure2-source data 1/pACT1-sec53-V238M/2x_V238M_79.png]

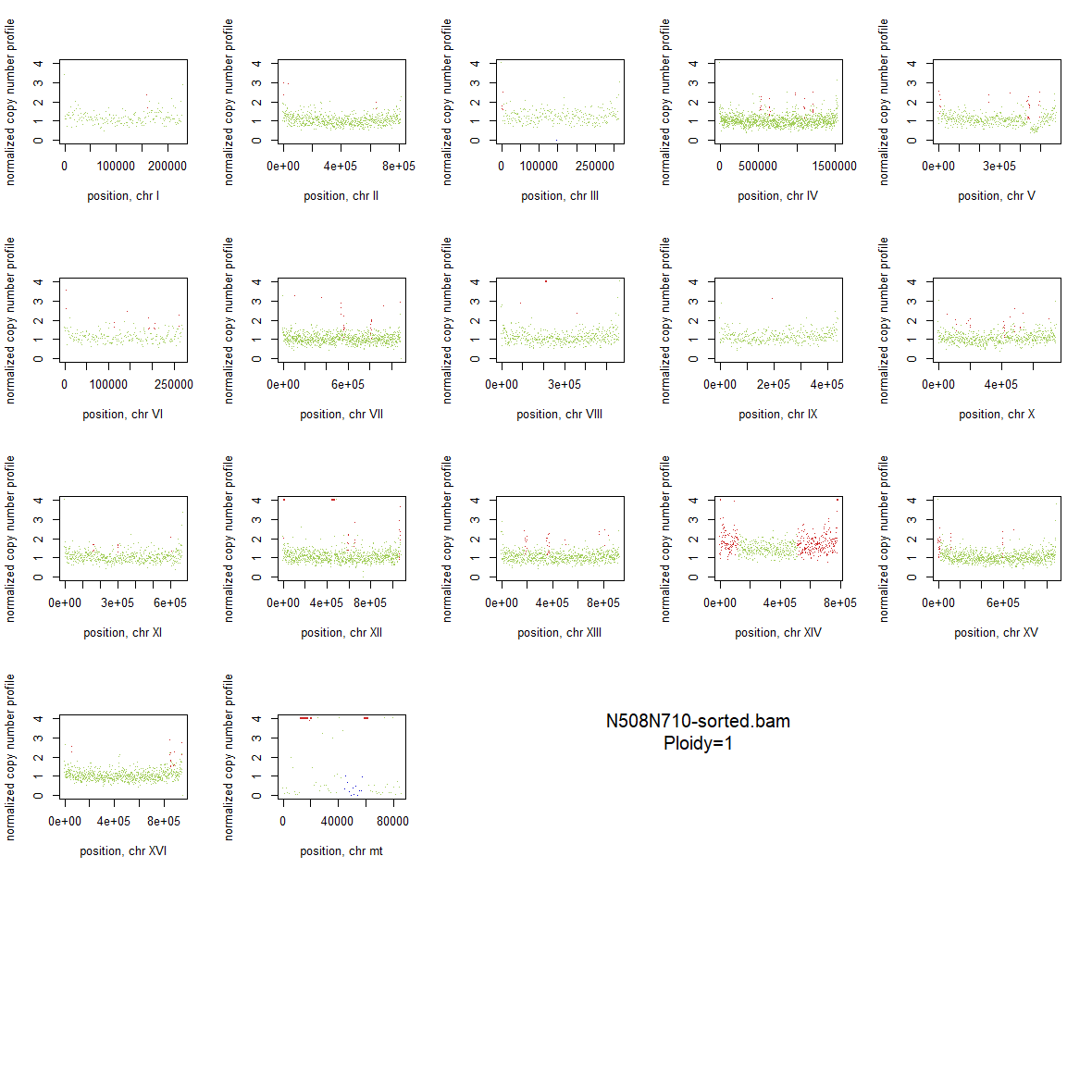

Supplement: Figure 2—source data 2. [file elife-79346-fig2-data2.zip › Figure2-source data 1/pACT1-sec53-V238M/2x_V238M_80.png]

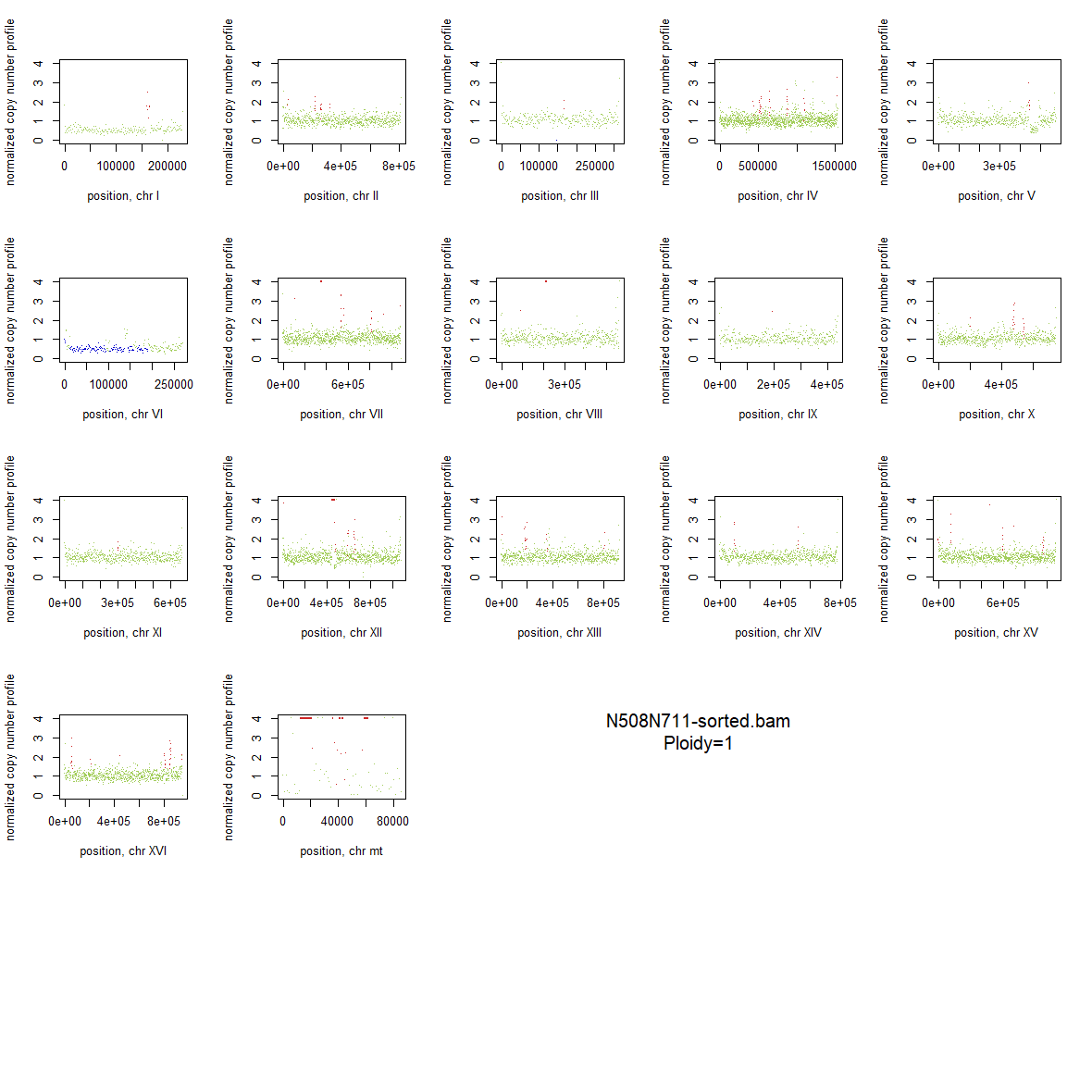

Supplement: Figure 2—source data 2. [file elife-79346-fig2-data2.zip › Figure2-source data 1/pACT1-sec53-V238M/2x_V238M_81.png]

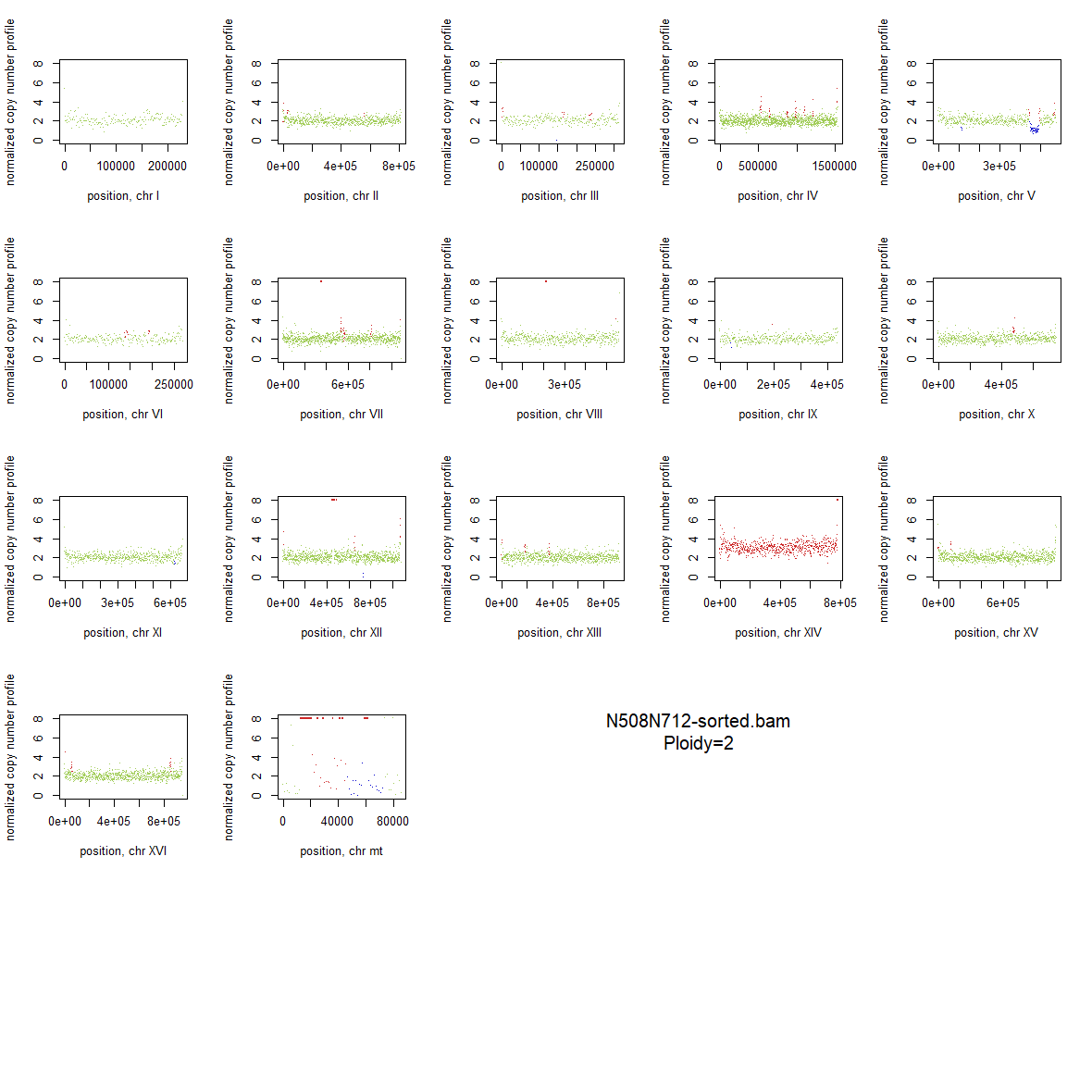

Supplement: Figure 2—source data 2. [file elife-79346-fig2-data2.zip › Figure2-source data 1/pACT1-sec53-V238M/2x_V238M_82.png]

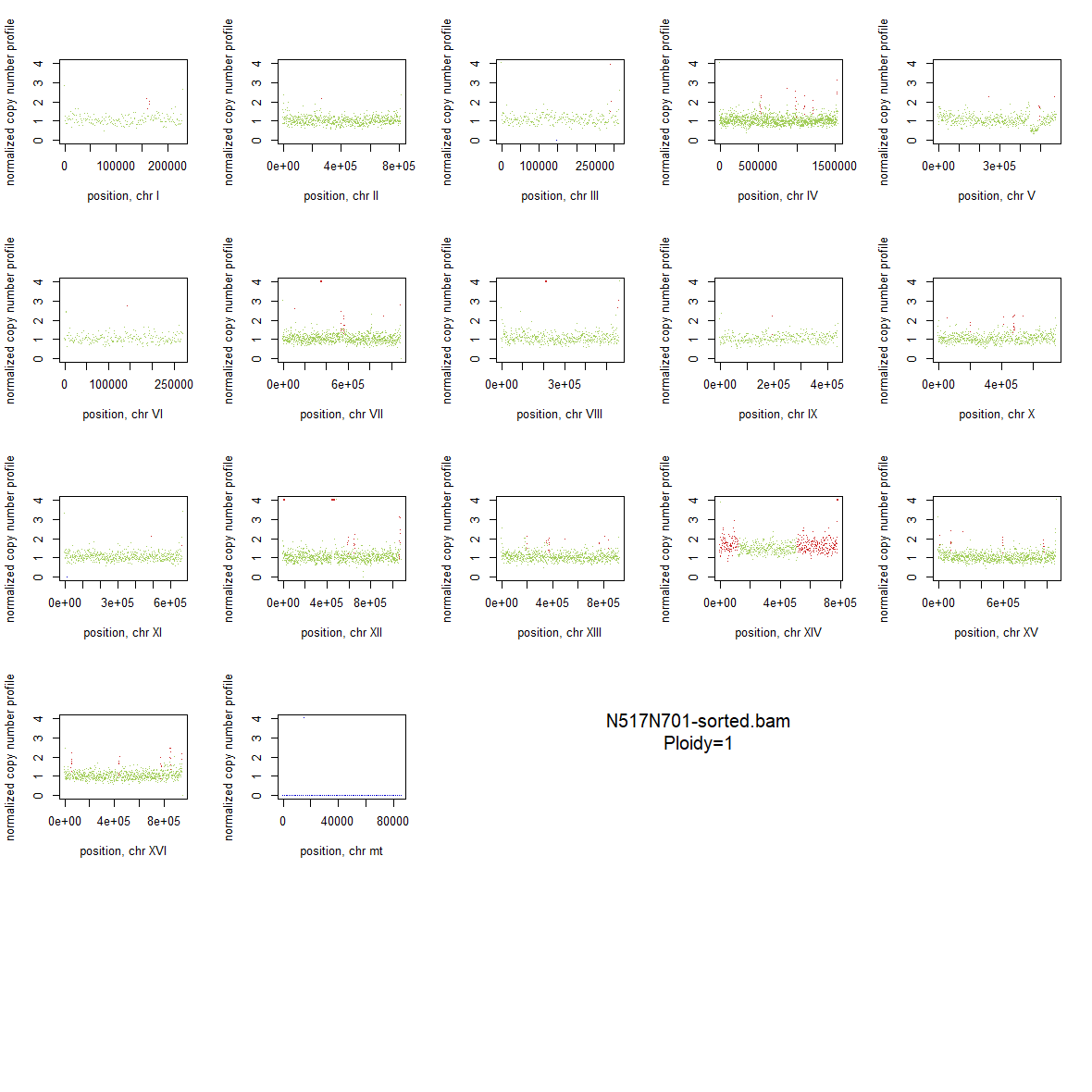

Supplement: Figure 2—source data 2. [file elife-79346-fig2-data2.zip › Figure2-source data 1/pACT1-sec53-V238M/2x_V238M_83.png]

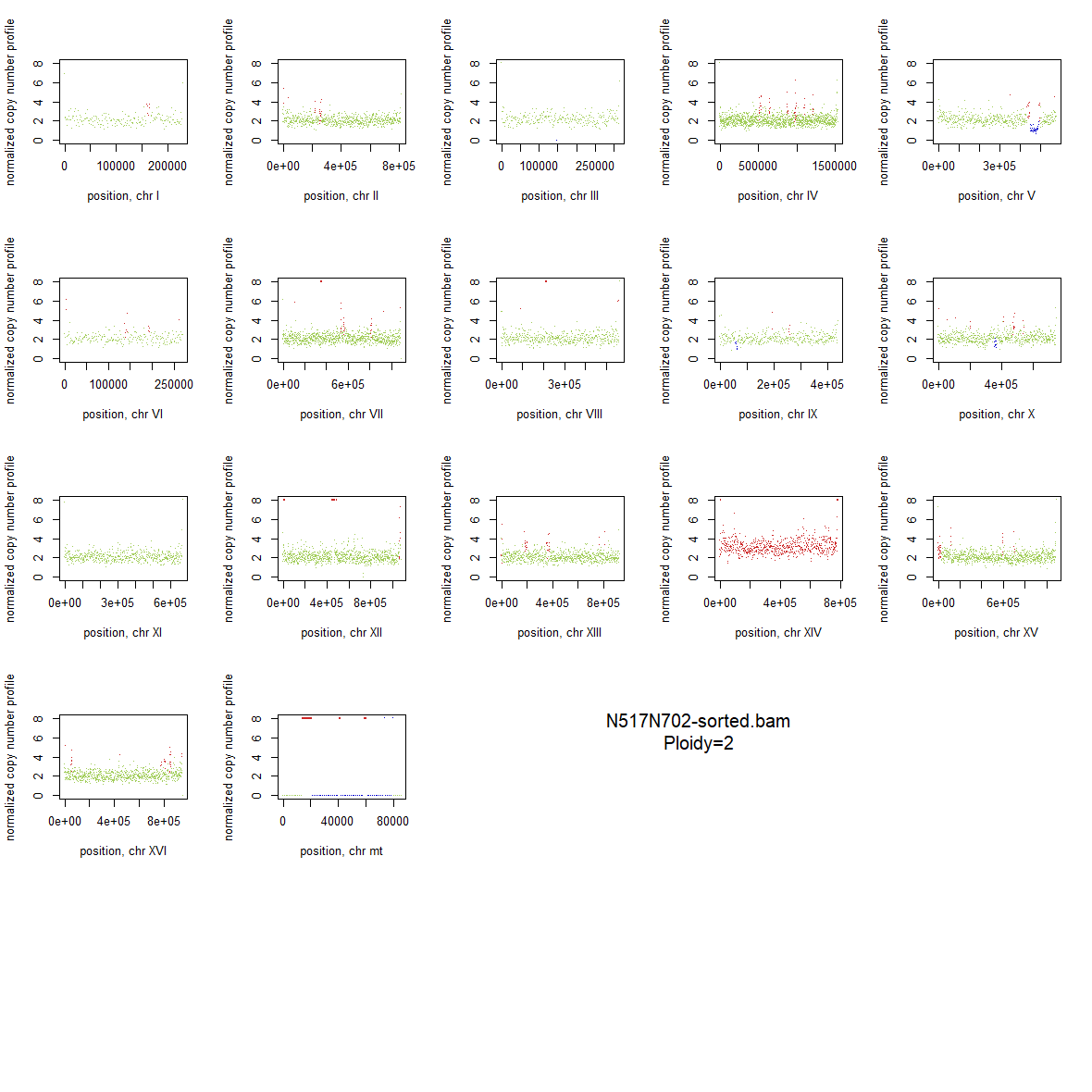

Supplement: Figure 2—source data 2. [file elife-79346-fig2-data2.zip › Figure2-source data 1/pACT1-sec53-V238M/2x_V238M_84.png]

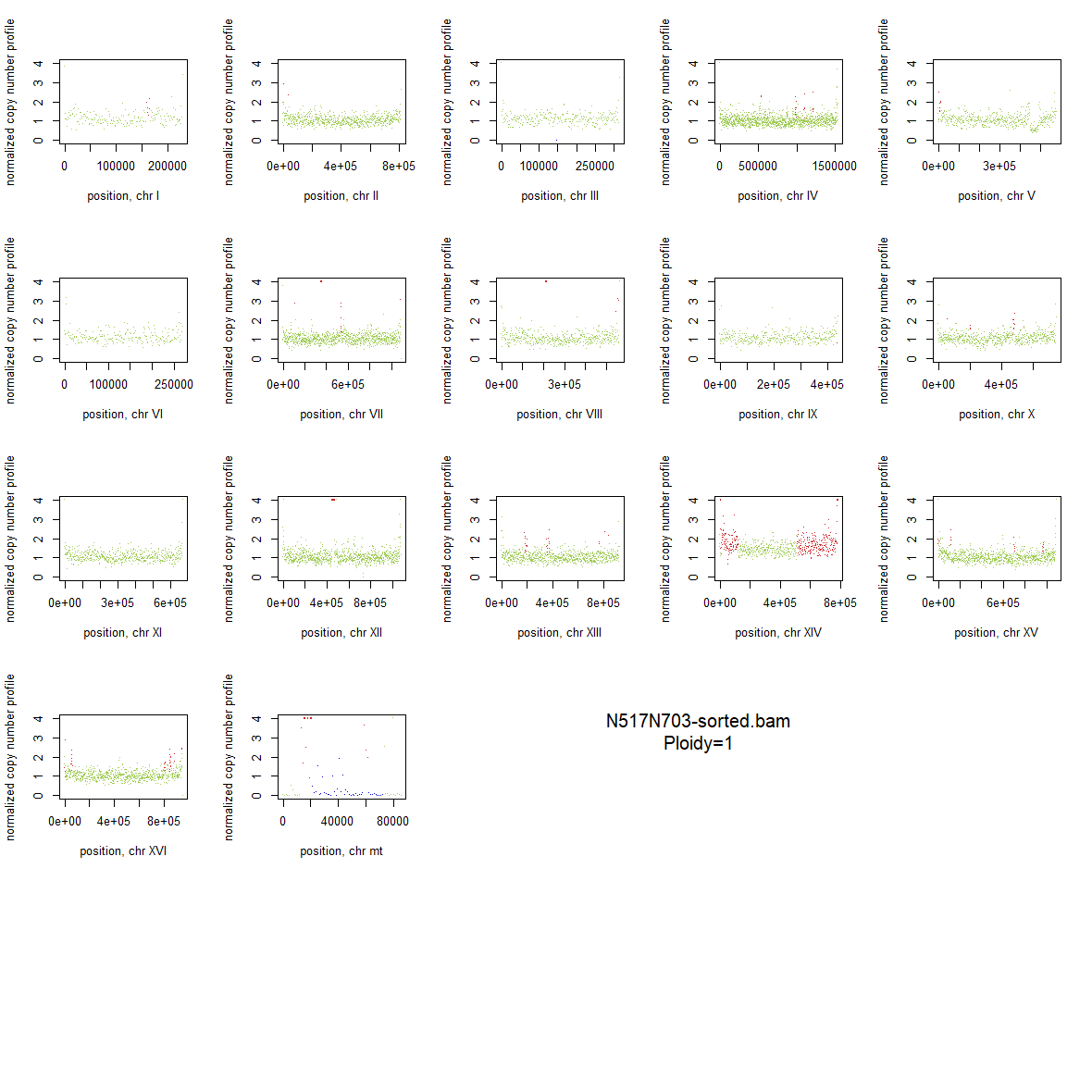

Supplement: Figure 2—source data 2. [file elife-79346-fig2-data2.zip › Figure2-source data 1/pACT1-sec53-V238M/2x_V238M_85.png]

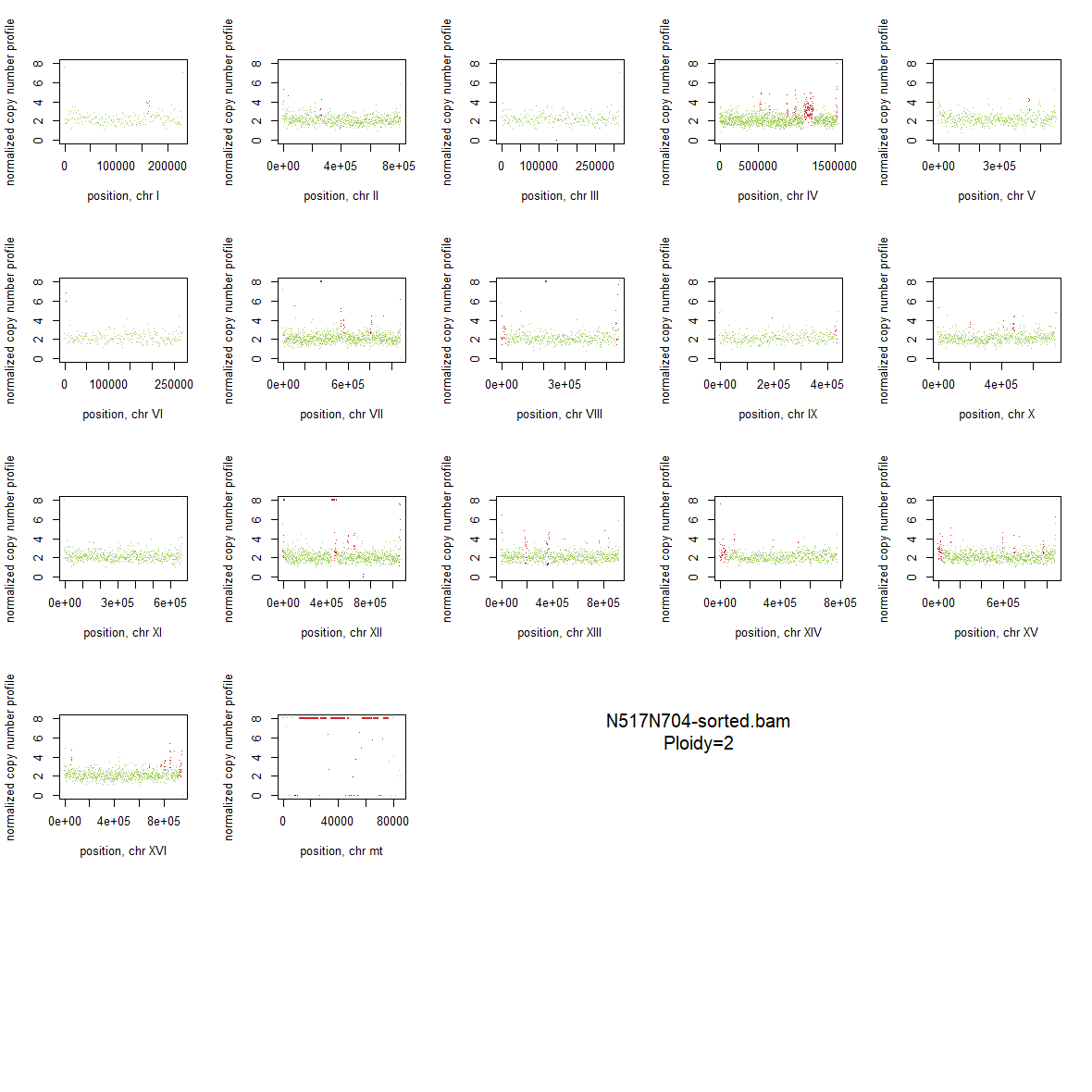

Supplement: Figure 2—source data 2. [file elife-79346-fig2-data2.zip › Figure2-source data 1/pACT1-sec53-V238M/2x_V238M_86.png]

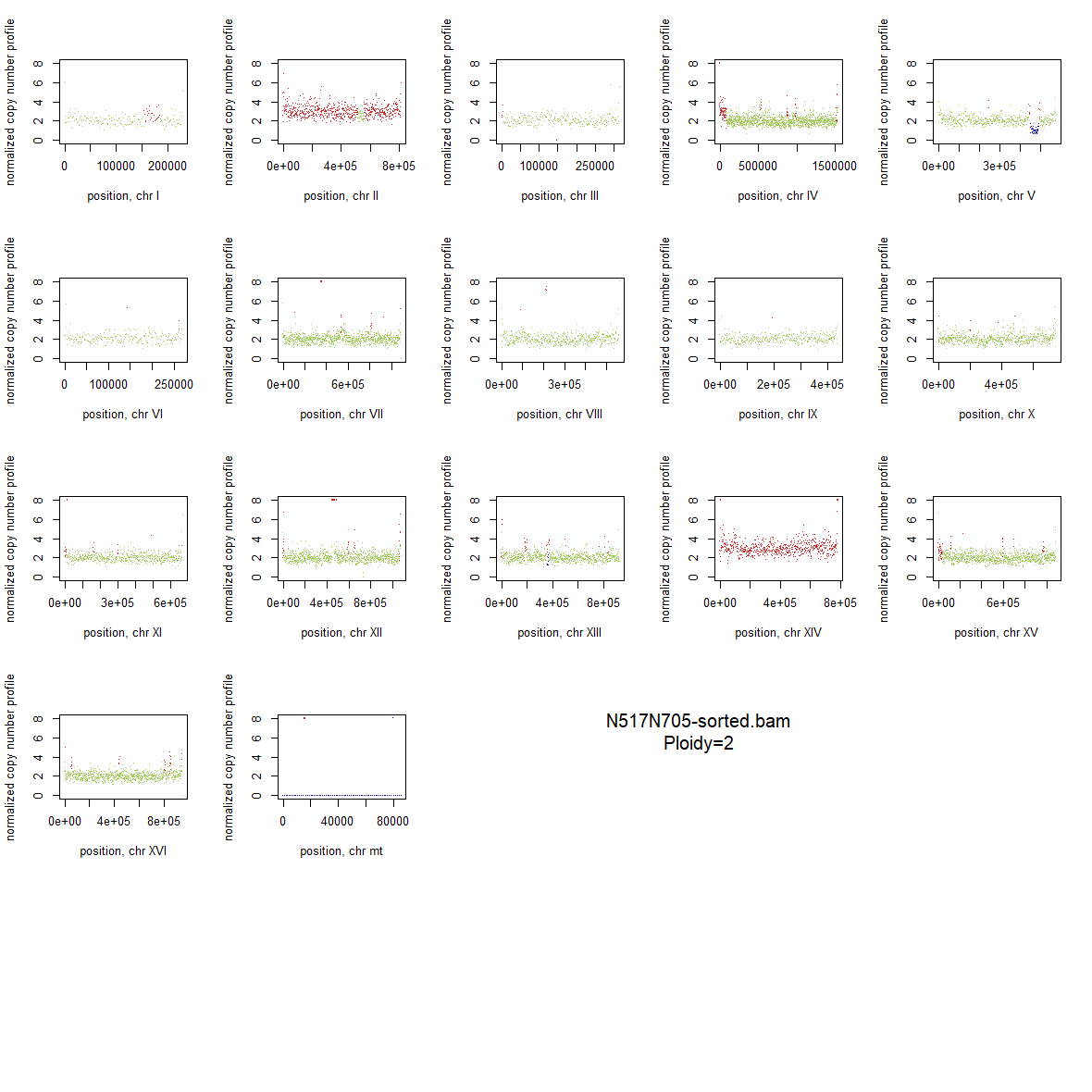

Supplement: Figure 2—source data 2. [file elife-79346-fig2-data2.zip › Figure2-source data 1/pACT1-sec53-V238M/2x_V238M_87.png]

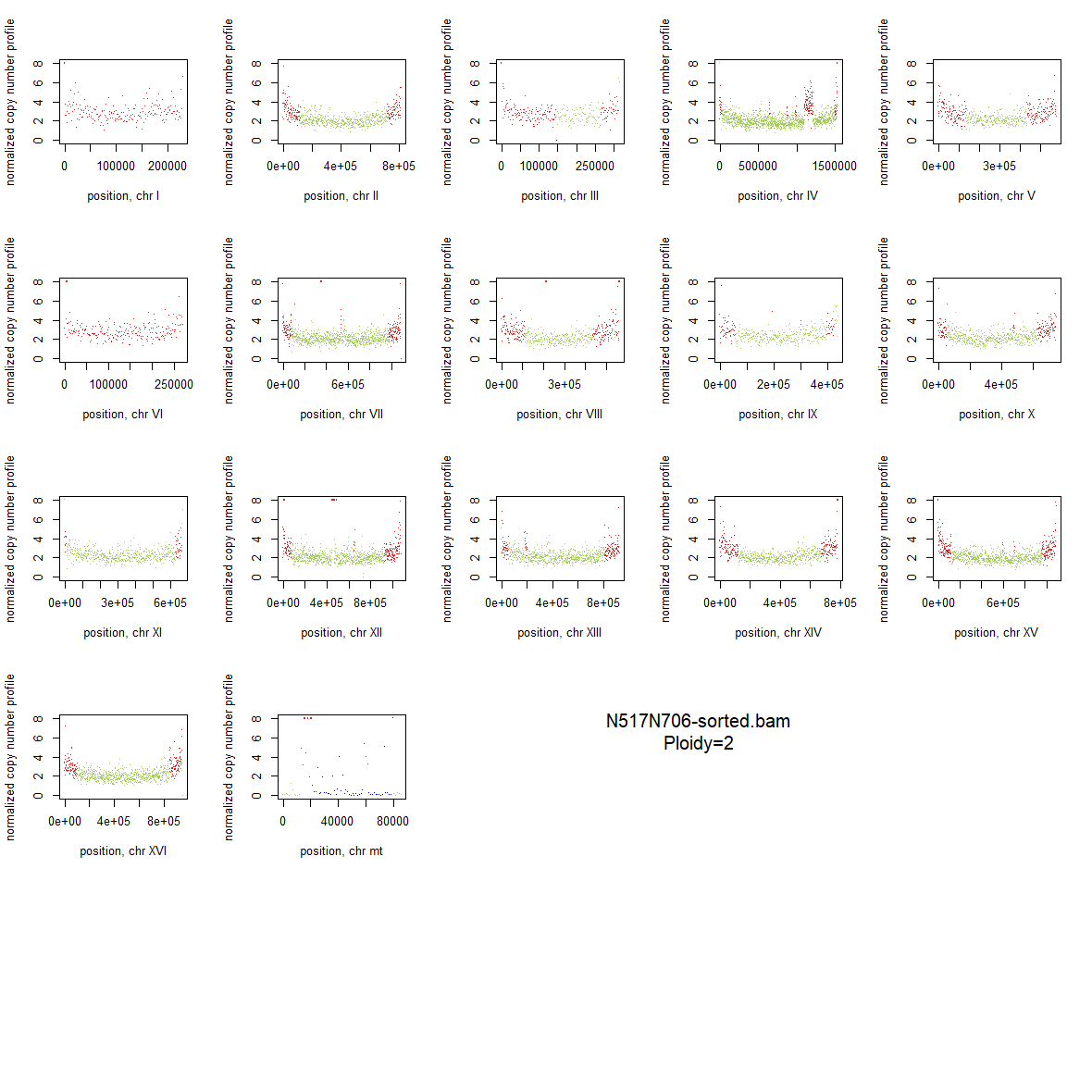

Supplement: Figure 2—source data 2. [file elife-79346-fig2-data2.zip › Figure2-source data 1/pACT1-sec53-V238M/2x_V238M_88.png]

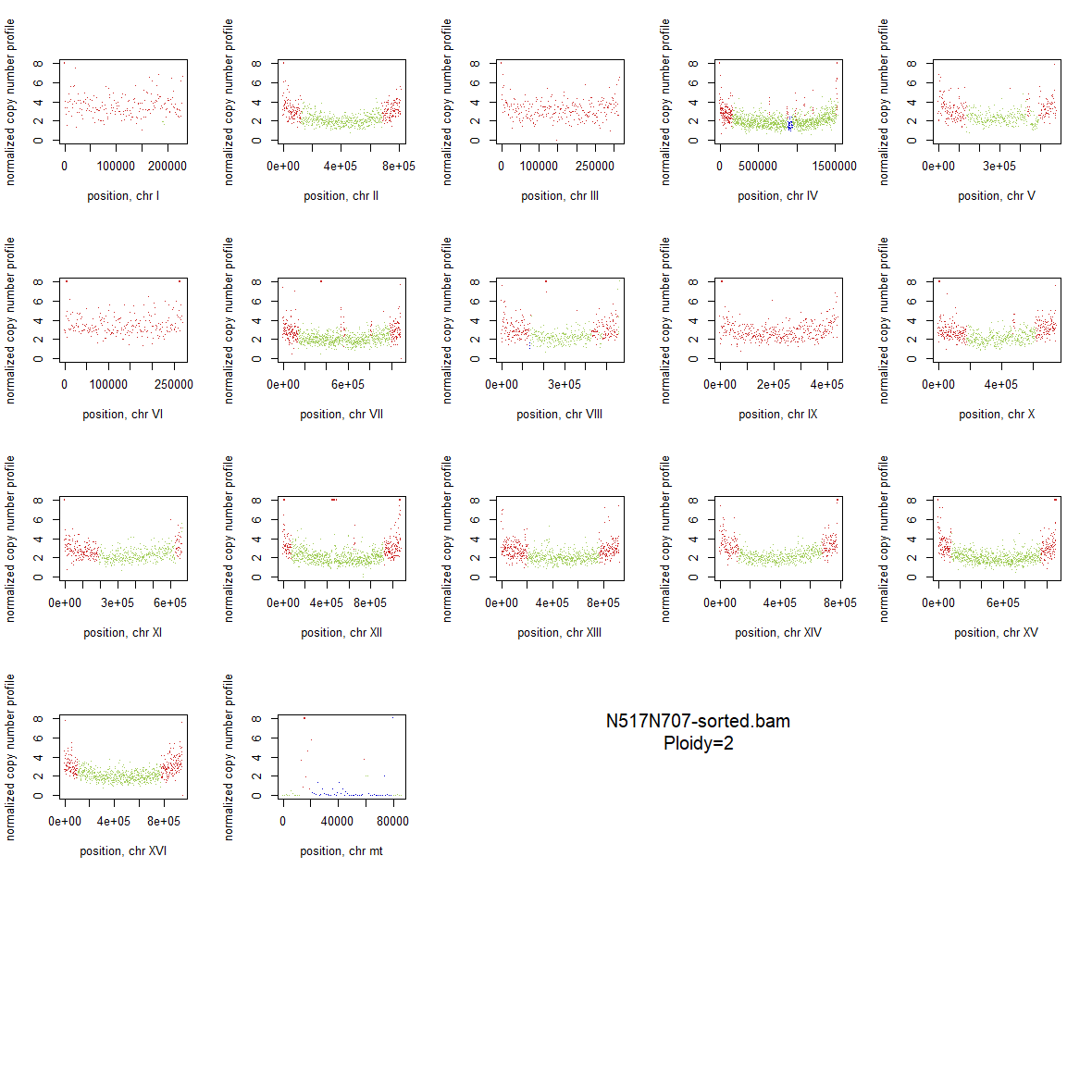

Supplement: Figure 2—source data 2. [file elife-79346-fig2-data2.zip › Figure2-source data 1/pACT1-sec53-V238M/2x_V238M_89.png]

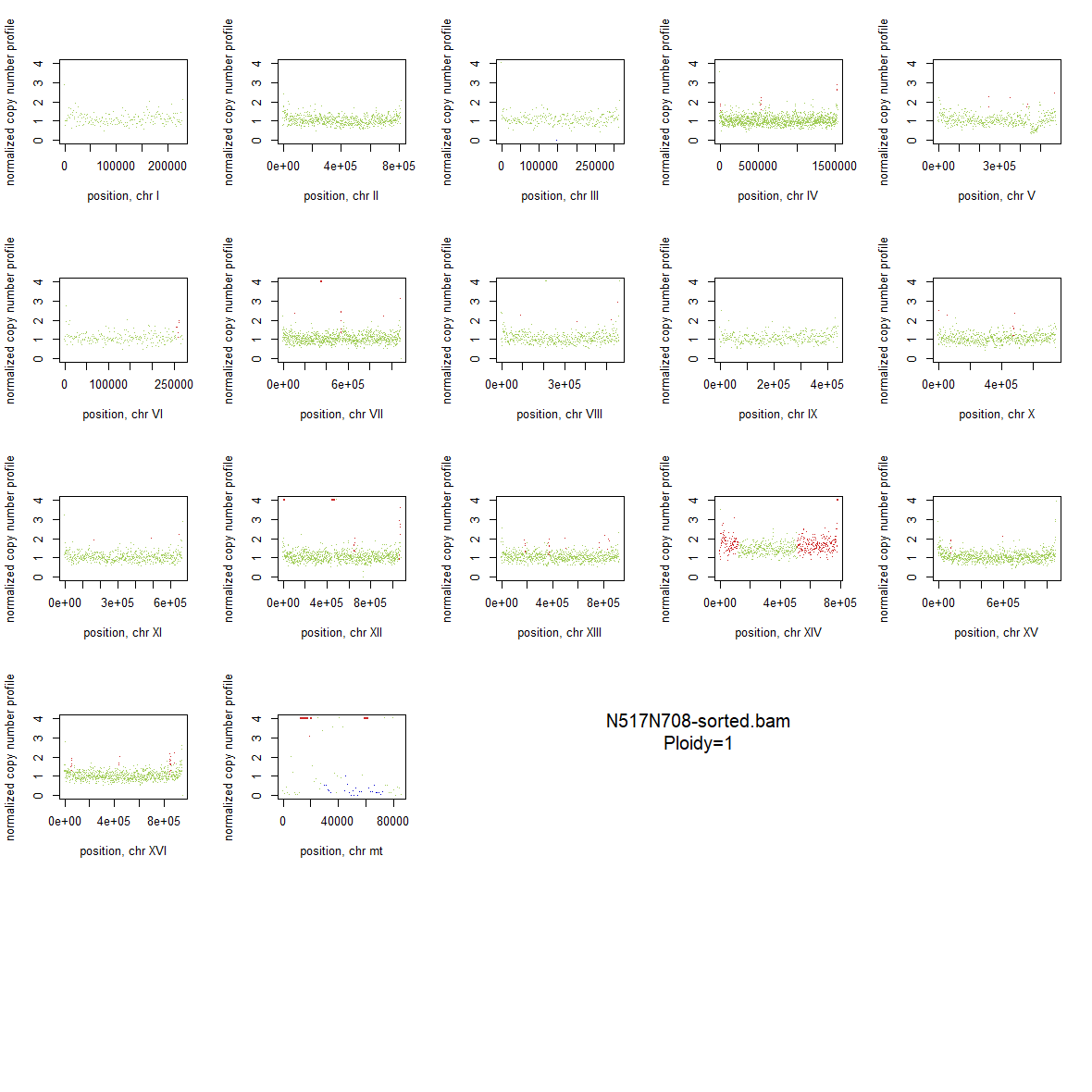

Supplement: Figure 2—source data 2. [file elife-79346-fig2-data2.zip › Figure2-source data 1/pACT1-sec53-V238M/2x_V238M_90.png]

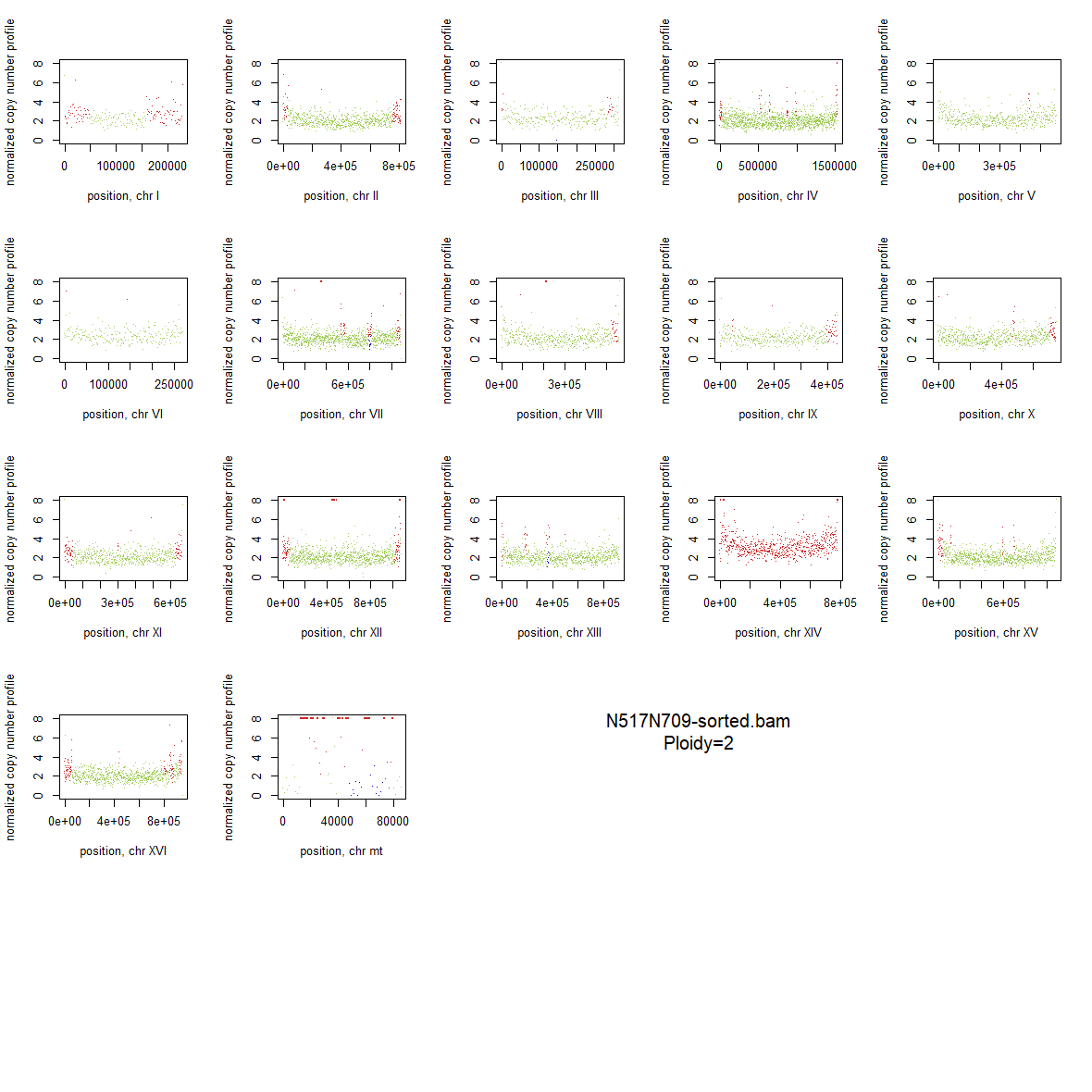

Supplement: Figure 2—source data 2. [file elife-79346-fig2-data2.zip › Figure2-source data 1/pACT1-sec53-V238M/2x_V238M_91.png]

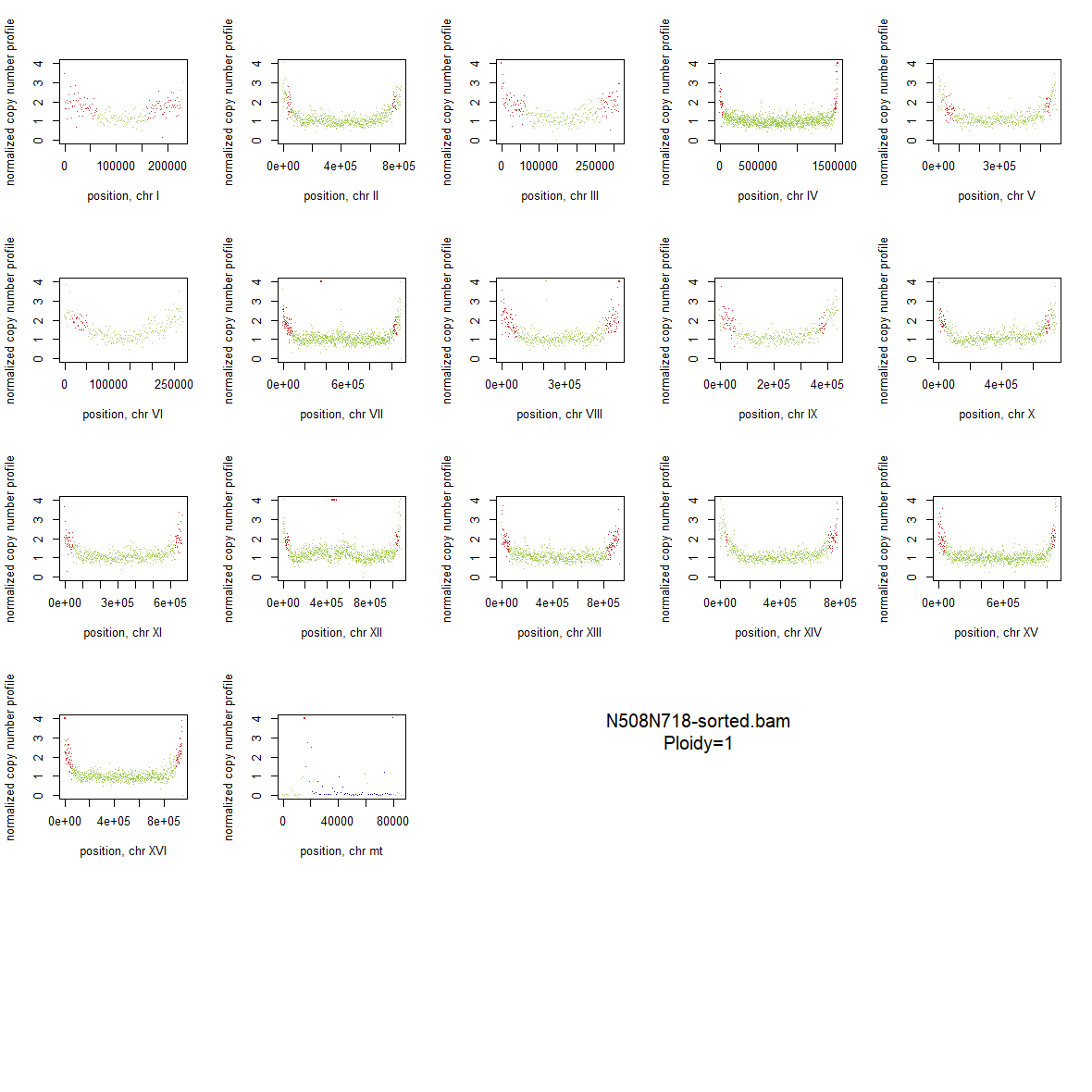

Supplement: Figure 2—source data 2. [file elife-79346-fig2-data2.zip › Figure2-source data 1/pACT1-SEC53-WT/2x_Wildtype_01.png]

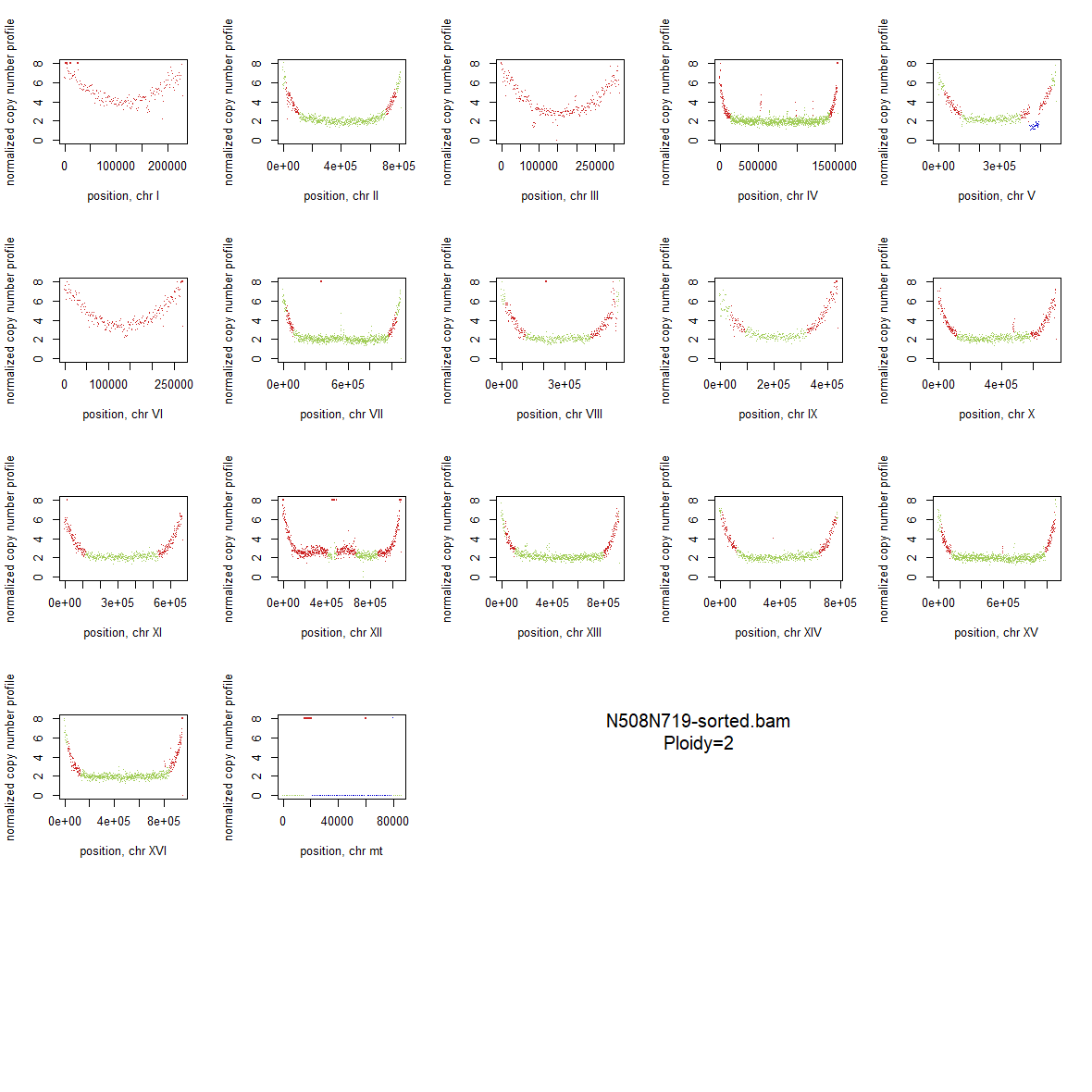

Supplement: Figure 2—source data 2. [file elife-79346-fig2-data2.zip › Figure2-source data 1/pACT1-SEC53-WT/2x_Wildtype_02.png]

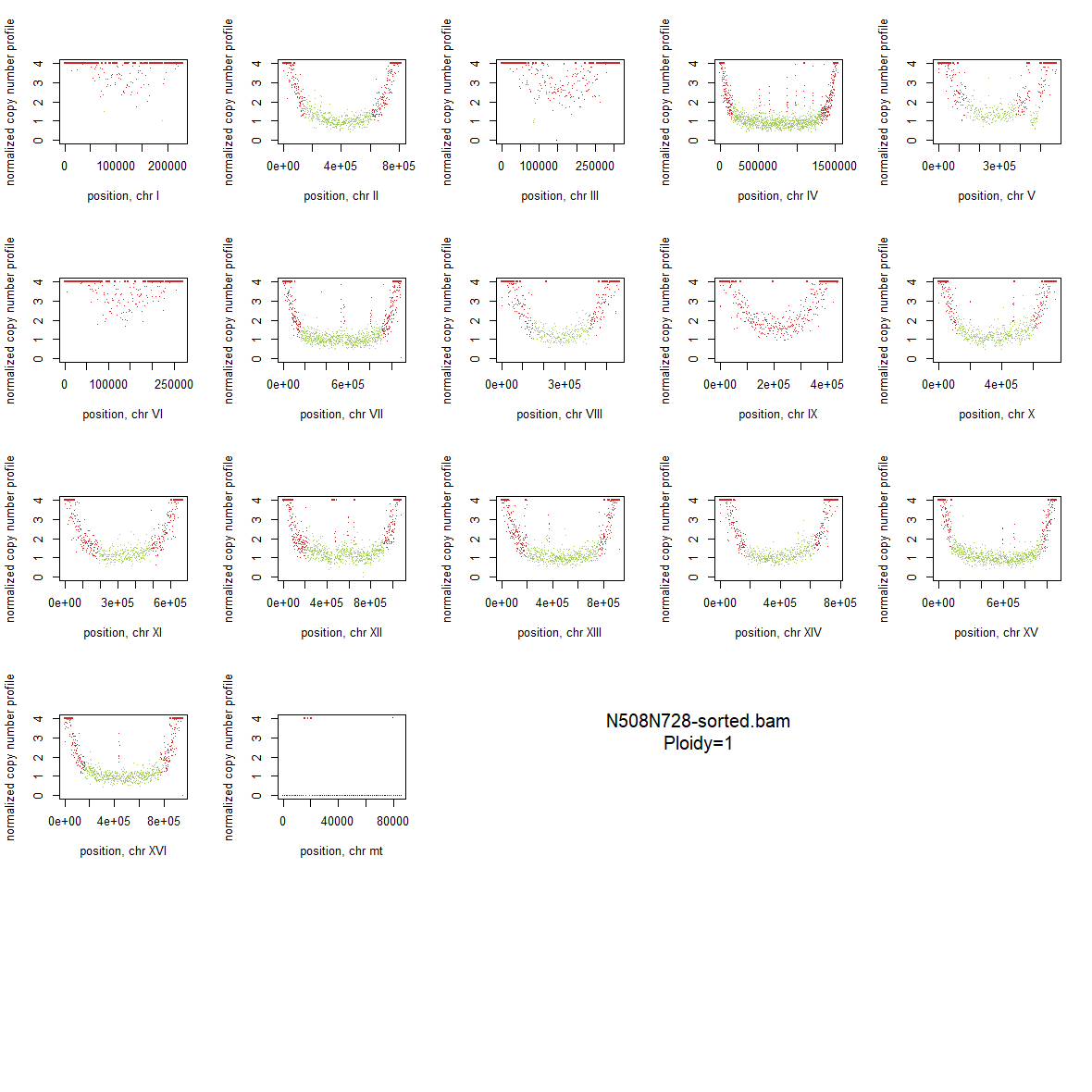

Supplement: Figure 2—source data 2. [file elife-79346-fig2-data2.zip › Figure2-source data 1/pACT1-SEC53-WT/2x_Wildtype_03.png]

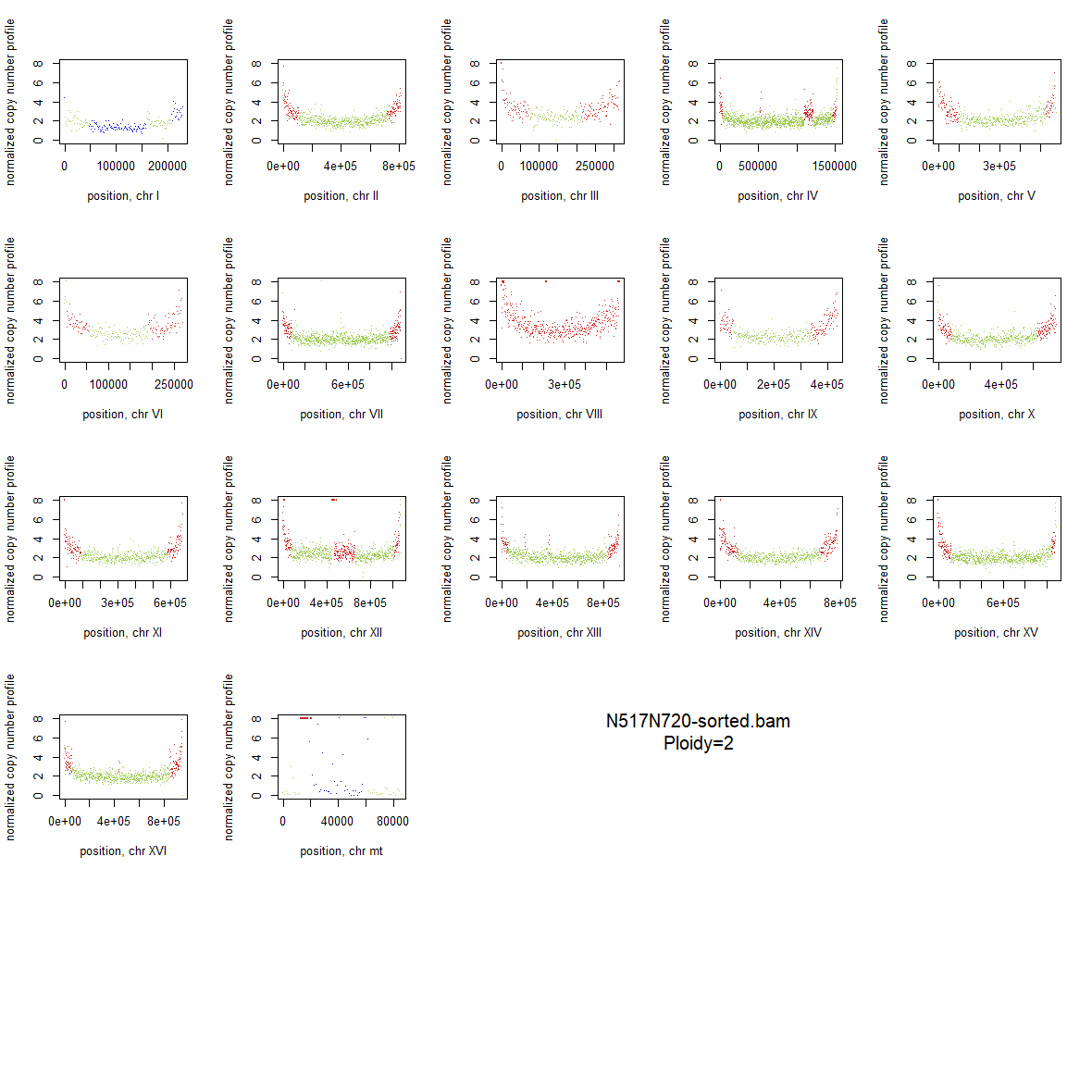

Supplement: Figure 2—source data 2. [file elife-79346-fig2-data2.zip › Figure2-source data 1/pACT1-SEC53-WT/2x_Wildtype_04.png]

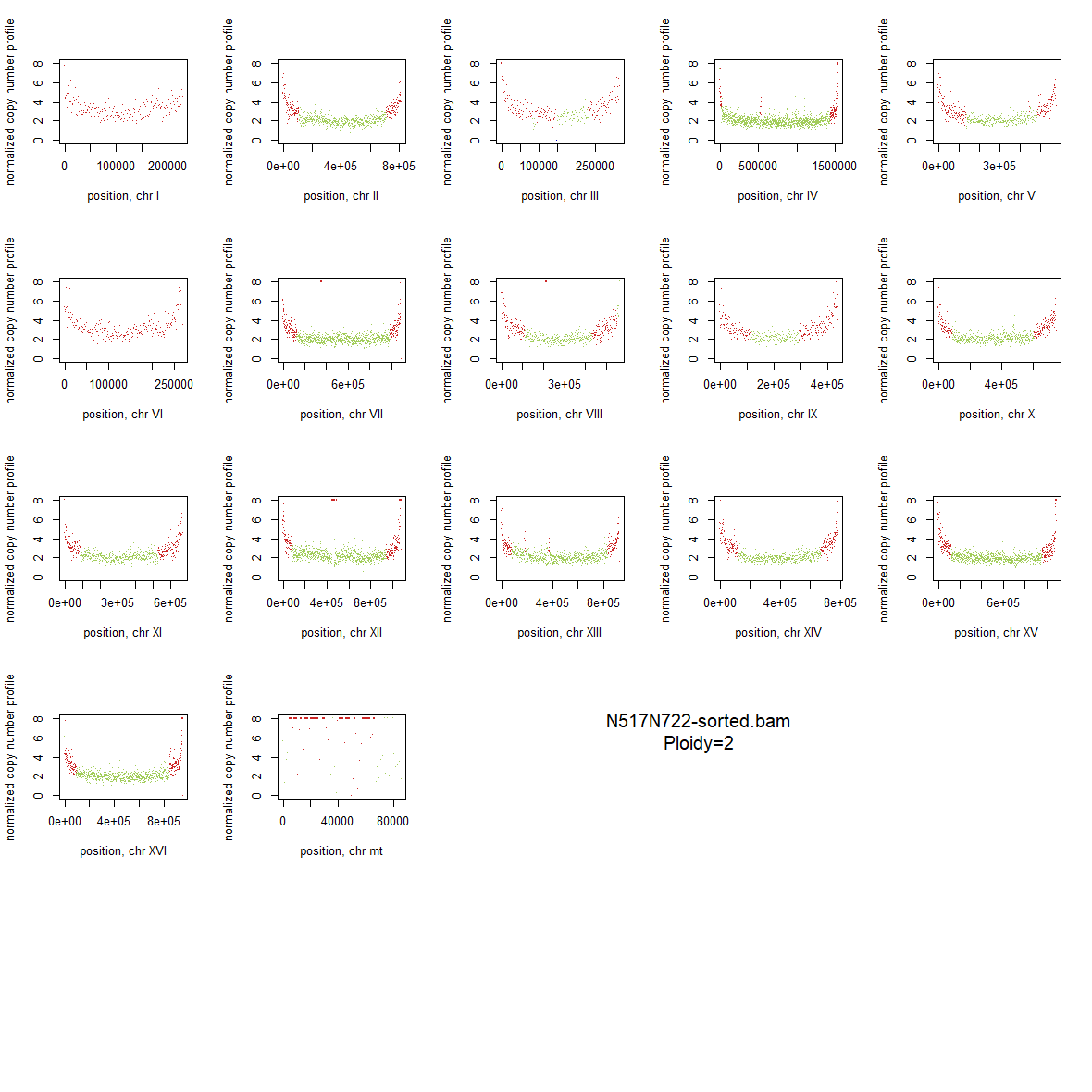

Supplement: Figure 2—source data 2. [file elife-79346-fig2-data2.zip › Figure2-source data 1/pACT1-SEC53-WT/2x_Wildtype_05.png]

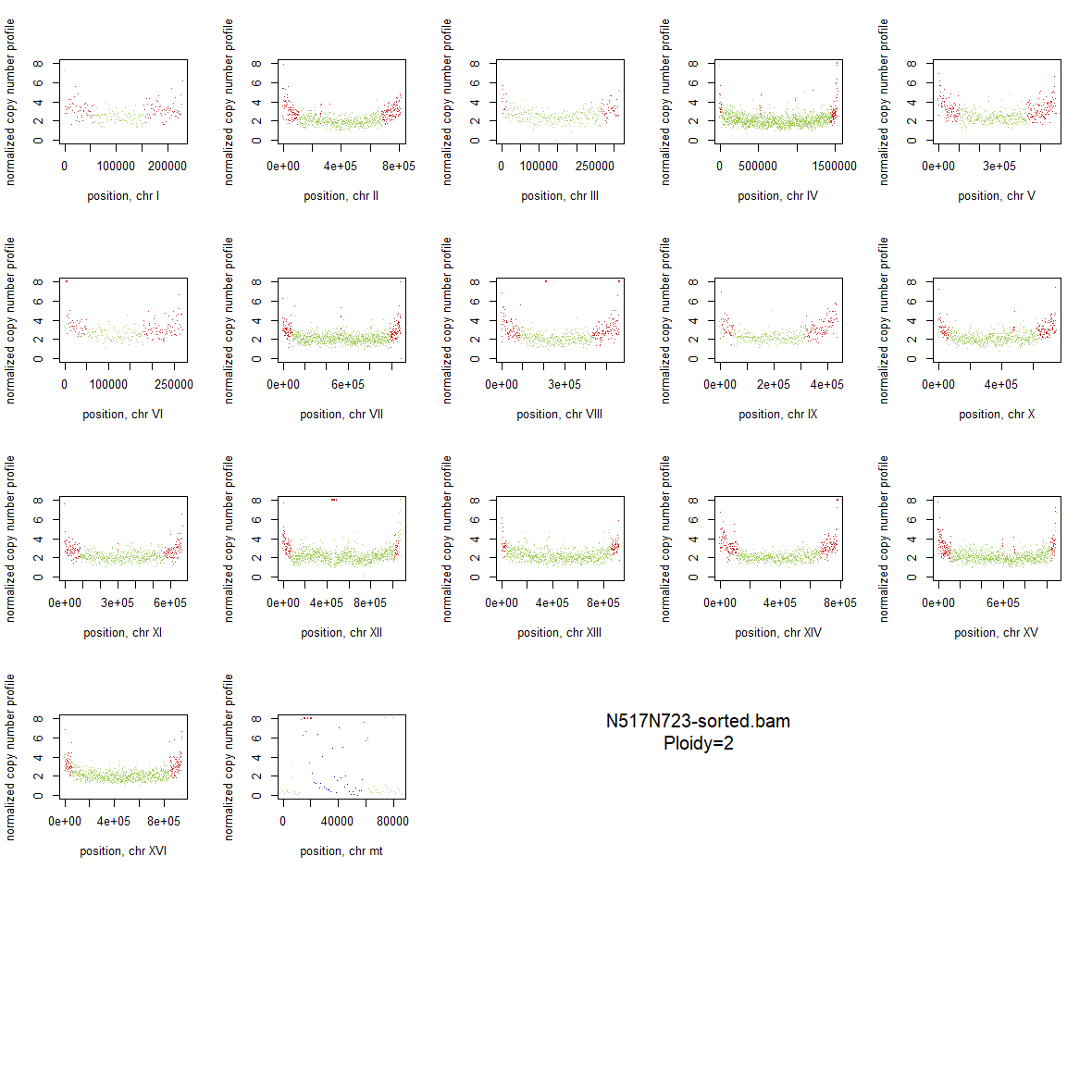

Supplement: Figure 2—source data 2. [file elife-79346-fig2-data2.zip › Figure2-source data 1/pACT1-SEC53-WT/2x_Wildtype_06.png]

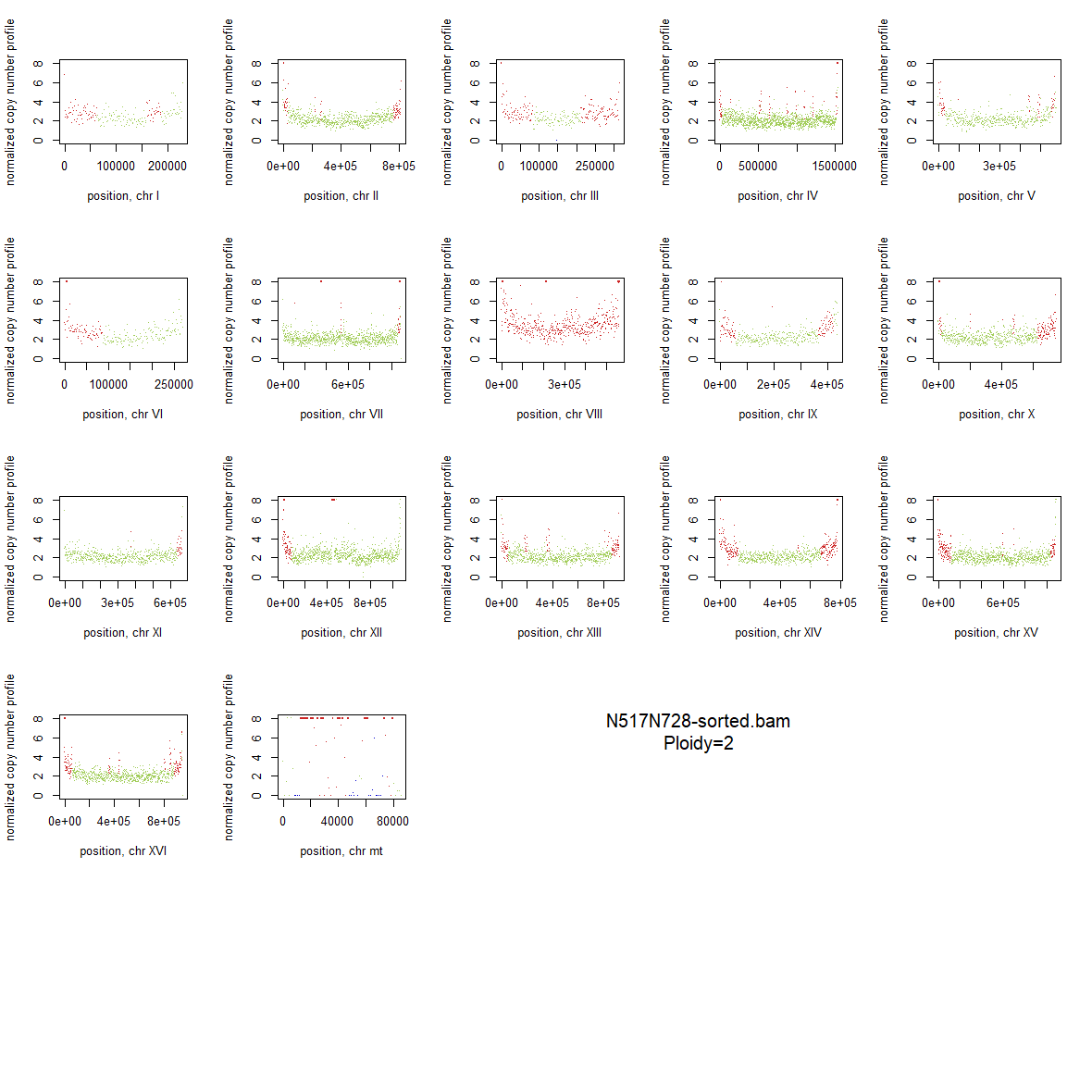

Supplement: Figure 2—source data 2. [file elife-79346-fig2-data2.zip › Figure2-source data 1/pACT1-SEC53-WT/2x_Wildtype_07.png]

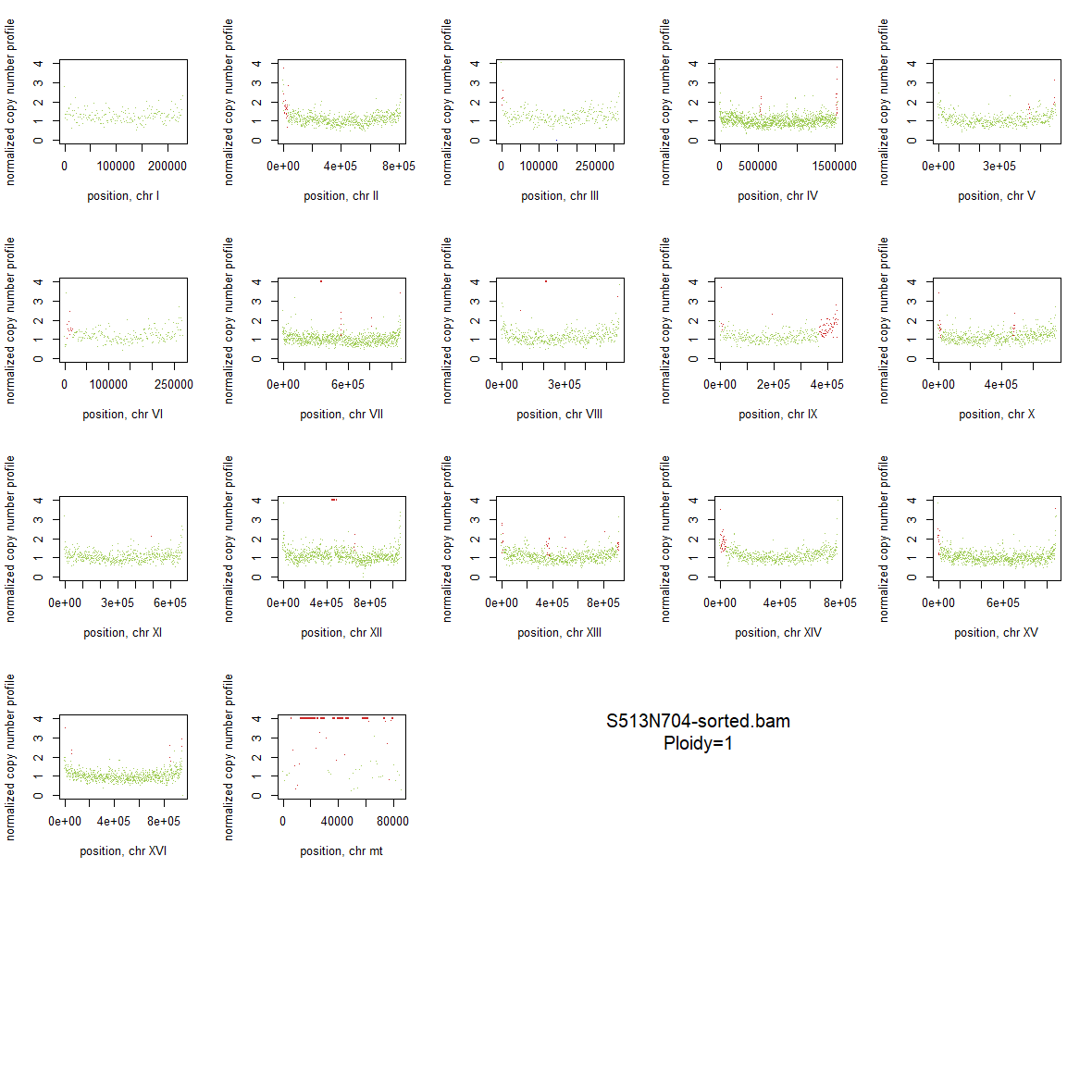

Supplement: Figure 2—source data 2. [file elife-79346-fig2-data2.zip › Figure2-source data 1/pACT1-SEC53-WT/2x_Wildtype_08.png]

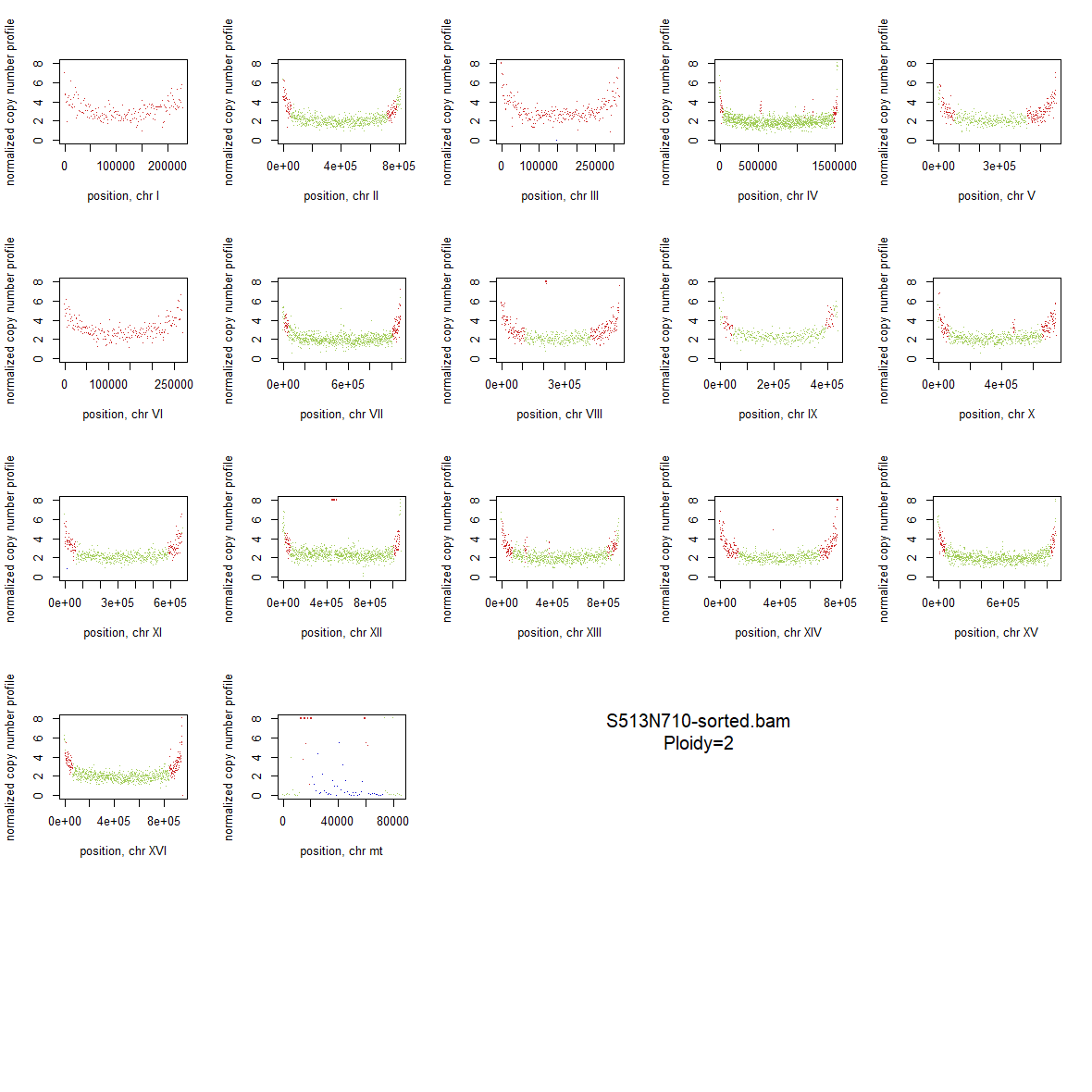

Supplement: Figure 2—source data 2. [file elife-79346-fig2-data2.zip › Figure2-source data 1/pACT1-SEC53-WT/2x_Wildtype_09.png]
